# Introducing the Catalytic Amination of Silanes via Nitrene Insertion

**DOI:** 10.1021/jacs.2c03739

**Published:** 2022-06-01

**Authors:** Anabel M. Rodríguez, Jorge Pérez-Ruíz, Francisco Molina, Ana Poveda, Raúl Pérez-Soto, Feliu Maseras, M. Mar Díaz-Requejo, Pedro J. Pérez

**Affiliations:** † Laboratorio de Catálisis Homogénea, Unidad Asociada al CSIC, CIQSO-Centro de Investigación en Química Sostenible and Departamento de Química, Universidad de Huelva, 21007 Huelva, Spain; § CICbioGUNE, Basque Research & Technology Alliance (BRTA), Bizkaia Technology Park, Building 800, 48160 Derio, Bizkaia, Spain; ‡ Institute of Chemical Research of Catalonia (ICIQ), The Barcelona Institute of Science and Technology, Avgda. Països Catalans, 16, 43007 Tarragona, Spain

## Abstract

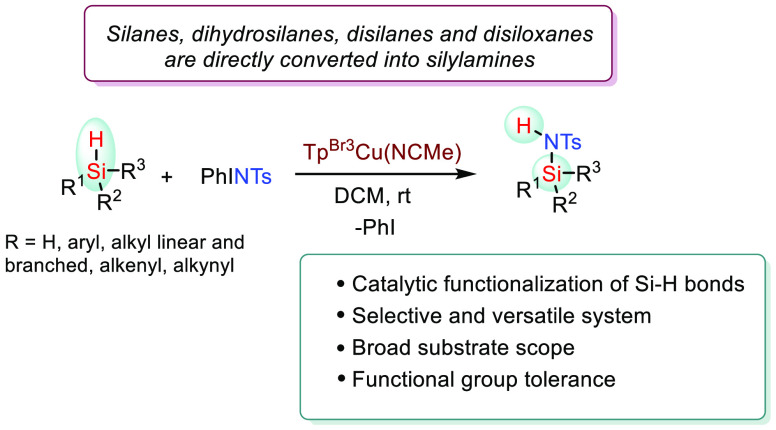

The direct functionalization of Si–H bonds by the nitrene insertion methodology is described. A copper(I) complex bearing a trispyrazolylborate ligand catalyzes the transfer of a nitrene group from PhI=NTs to the Si–H bond of silanes, disilanes, and siloxanes, leading to the exclusive formation of Si–NH moieties in the first example of this transformation. The process tolerates other functionalities in the substrate such as several C–H bonds and alkyne and alkene moieties directly bonded to the silicon center. Density functional theory (DFT) calculations provide a mechanistic interpretation consisting of a Si–H homolytic cleavage and subsequent rebound to the Si-centered radical.

## Introduction

**Scheme 1:**
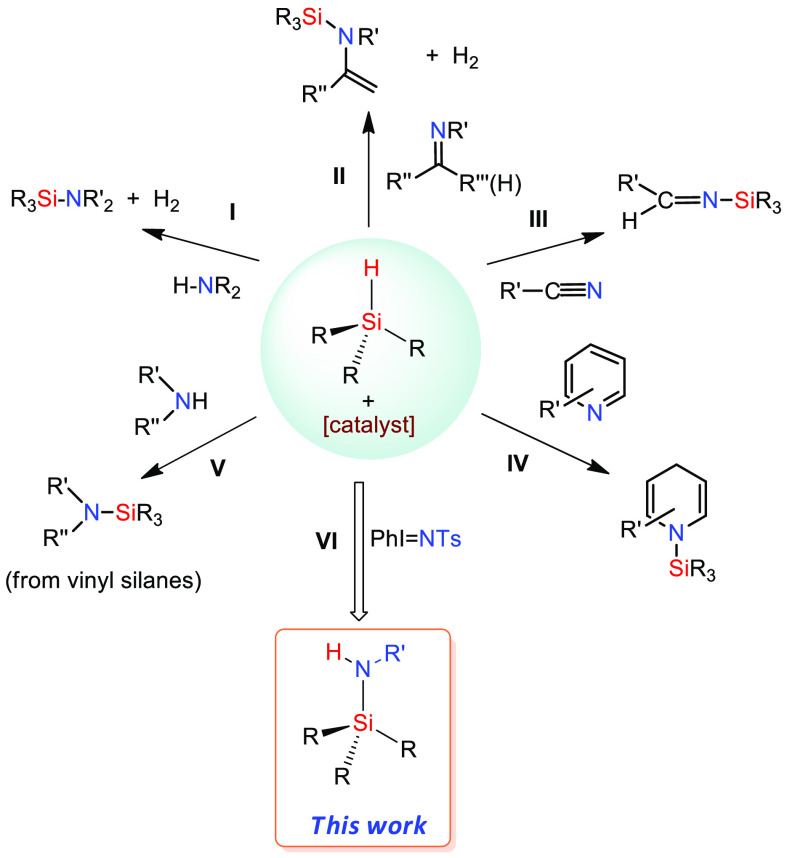
Strategies for Si–N Bond Formation

Silicon-based compounds bearing Si–N bonds constitute an important class within both organic/inorganic fields with applications ranging from ligands to protecting groups, bases, or functional materials.^1,2^ Several methods have been reported to date for the construction of Si–N bonds from silanes (Scheme 1). In addition to the stoichiometric reaction of chlorosilanes with amines (and subsequent elimination of HCl), the following catalytic processes are known for generating such groups: (a) the dehydrocoupling of amines and hydrosilanes (Scheme 1, I);^3^ (b) the hydrosilylation reaction of hydrolyzable imines^4^ (Scheme 1, II); (c) the hydrosilylation of nitriles (Scheme 1, III);^5^ (d) the use of pyridines as N sources (Scheme 1, IV);^6^ (e) the N-silylation employing vinyl-silanes (Scheme 1, V).^7^ In all cases, the hydrogen from the parent Si–H bond is lost during the transformation.^8^

**Scheme 2:**
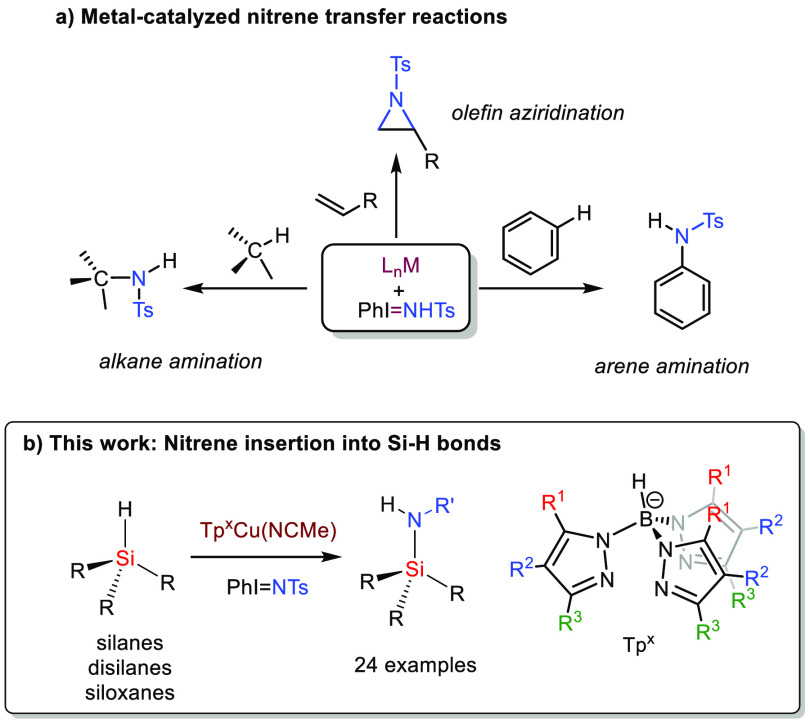
(a) Typical Examples of Known Nitrene Transfer Reactions; (b) Novel Procedure for Si–H Functionalization by Nitrene Insertion

The metal-catalyzed nitrene transfer employing hypervalent iodine compounds or organic azides as the nitrene source has been successfully employed to introduce the NR unit into an array of unsaturated and saturated bonds, including C–H bonds (Scheme 2a).^9^ The reaction occurs through metal–nitrene intermediates,^10^ which induce the homolytic C–H bond cleavage and subsequent C–N bond formation.^11^ Surprisingly, the related reaction onto a Si–H bond leading to the Si–N bond formation remains, to the best of our knowledge, yet unreported. In the last two decades, our research group has developed significant activity in the field of catalytic nitrene transfer reactions using complex Cu- and Ag-based catalysts bearing trispyrazolylborate ligands.^12^ In general, Tp^x^M complexes (M = Cu, Ag) are highly active and selective catalysts for the aziridination reactions of olefins and dienols^13^ as well as for nitrene insertion into C–H bonds of arenes and alkanes.^14^ Herein, we describe the first example of such a transformation in which the Si–H bonds of silanes, disilanes, and siloxanes are modified upon insertion of a nitrene group into such a moiety. The process takes place at room temperature and with high selectivity (Scheme 2b), and the maintenance of the hydrogen atom from the initial Si–H bond provides a certain degree of atom economy to the reaction.

## Results and Discussion

### Catalytic Reaction Model: Dimethyl(Phenyl)Silane with PhI=NTs

**Scheme 3:**
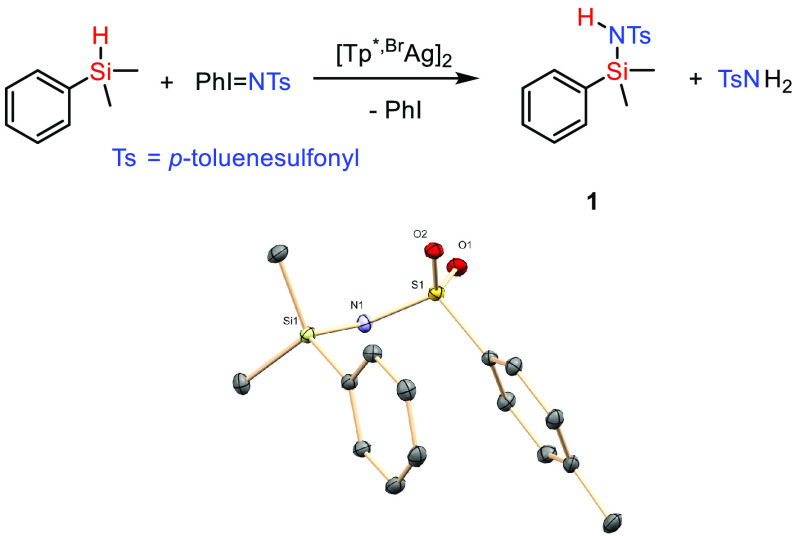
Catalytic Functionalization of Dimethylphenylsilane with PhI=NTs Using [Tp^*,Br^Ag]_2_ as the Catalyst

We first faced the functionalization of a model substrate such as dimethyl(phenyl)silane with PhI=NTs. As a catalyst, we chose the silver complex [Tp^*,Br^Ag]_2_ for which we reported the best catalytic activity for the alkane C–H bond amination reaction.^11a^ This dinuclear compound in solution delivers monomeric Tp^*,Br^Ag units, which react with PhI=NTs to give the silver–nitrene complex.^15^ The experimental methodology is quite simple: a solution of the catalyst and the silane in dichloromethane at room temperature is prepared before solid PhI=NTs is added, which slowly dissolves. Stirring at room temperature for 45 min led to complete consumption of the latter. Removal of volatiles and NMR studies of the reaction crude revealed the formation (Scheme 3) of new compound **1** in 65% yield as well as some TsNH_2_ from PhI=NTs decomposition. Compound **1** was purified by column chromatography with reverse phase C18 silica gel and isolated as an off-white solid. The ^1^H NMR spectrum shows a resonance at −0.55 ppm for the SiMe_2_ group as well as a broad singlet at 4.66 ppm assigned to the N–H moiety. No Si–H resonance is observed, the spectrum being completed with the expected resonances for the tosyl and phenyl groups. The ^13^C NMR data are consistent with the formulation proposed for **1**. The change in the chemical shifts in the ^29^Si NMR spectra from −17.1 ppm (starting silane) to 1.0 ppm for **1** is noted. Finally, single crystals of this compound were obtained upon cooling petroleum ether/hexane solutions, leading to the determination of the solid-state structure (Scheme 3),^16^ which confirmed that proposed from the spectroscopic data.

**Figure 1:**
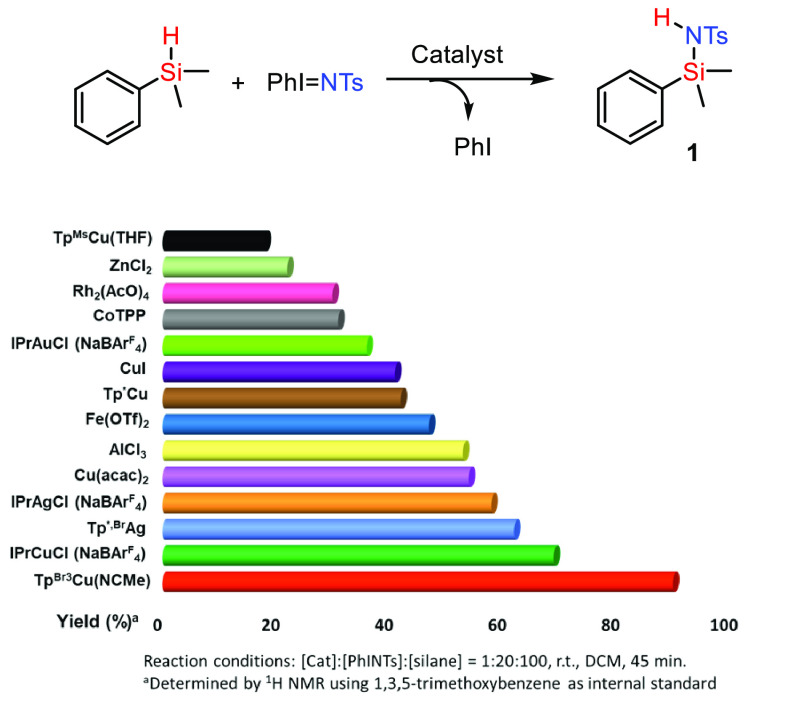
Catalyst screening for the nitrene transfer reaction onto dimethylphenylsilane.

Once the insertion of the NTs group into the Si–H bond was assessed, catalyst screening was carried out with the same probe reaction of PhI=NTs and dimethylphenylsilane. An array of several Cu-, Ag-, and Au-based complexes, either with Tp^x^ (hydrotrispyrazolyborate) or NHC (N-heterocyclic carbene) ligands, were employed as well as some representative examples of Cu, Rh, Co, or Lewis acids (Zn-, Fe-, or Al-based), given literature precedents for their competence in nitrene transfer.^9^ The results are shown in Figure 1 (see the Supporting Information for details). Most of the candidates showed catalytic activity within the 20–60% yield into **1**, with only three of them surpassing that value. In addition to the already mentioned [Tp^*,Br^Ag]_2_, IPrCuCl induced 70% yield whereas Tp^Br3^Cu(NCMe) led to the maximum value of 90%, being by far the best of the whole series. Reaction conditions were further optimized in terms of stoichiometry, solvent, and nitrene precursor (see the Supporting Information), leading to the use of PhI=NTs in dichloromethane and a 1:5 [PhI=NTs]/[silane] ratio as the most productive conditions.

### Scope of the Silanamination Reaction

**Scheme 4:**
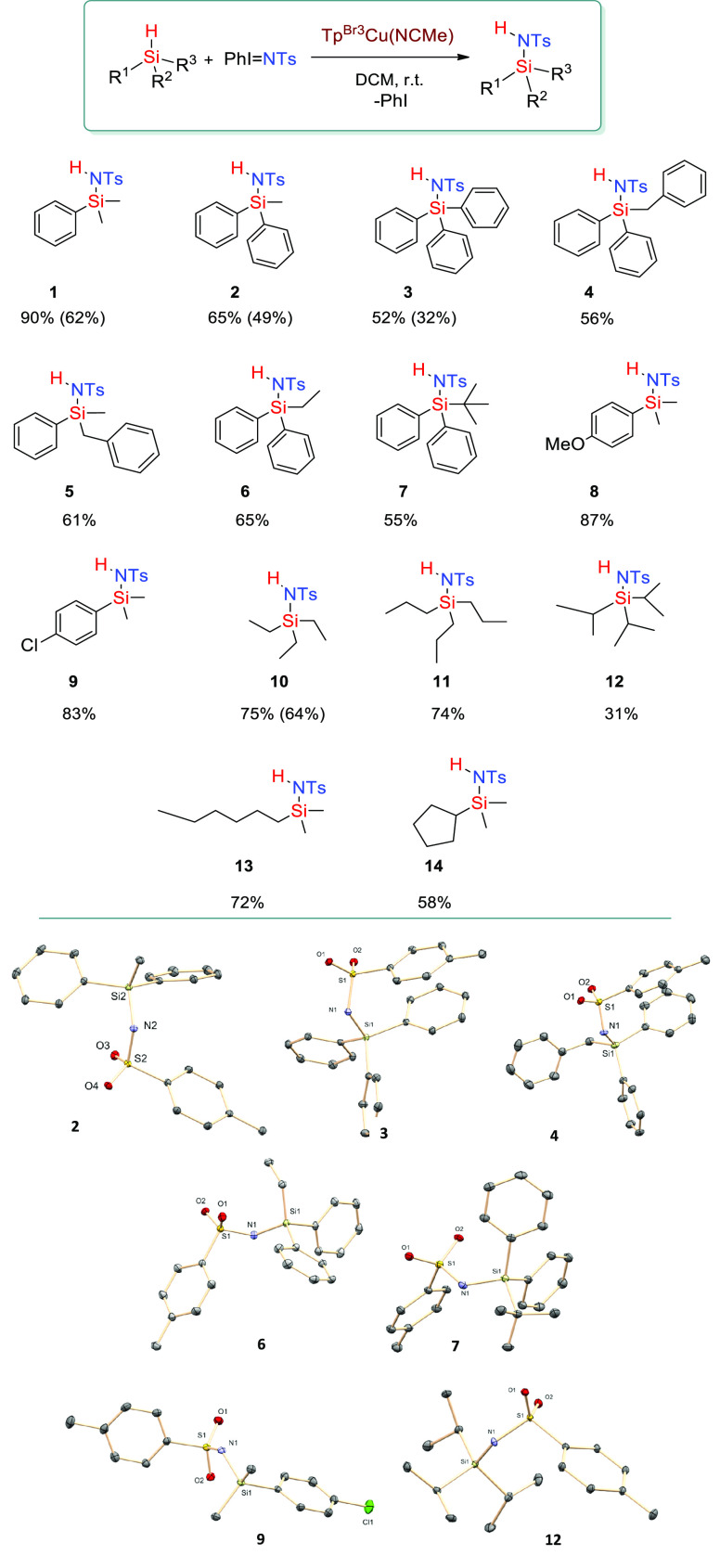
Scope of the Silane Functionalization by Nitrene Insertion Using Tp^Br3^Cu(NCMe) as Catalyst Yields determined with an internal standard; values in brackets correspond to isolated yields. Reaction conditions: [Cat]/[PhINTs]/[silane] = 1:20:100, r.t., DCM, 45 min. Yields determined by ^1^H NMR using 1,3,5-trimethoxybenzene as the internal standard. See the Supporting Information for experimental details.

**Scheme 5:**
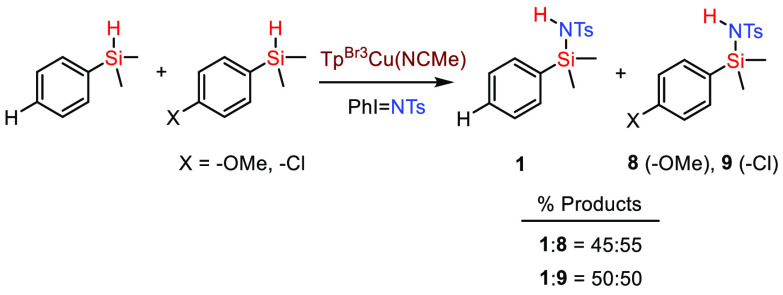
Competition Experiments with Aryldimethylsilanes Reaction conditions: [Cat]/[PhINTs]/[silane]/[silane-X] = 1:20:50:50, r.t., DCM, 45 min. Yields determined by ^1^H NMR using 1,3,5-trimethoxybenzene as the internal standard. See the Supporting Information for experimental details.

After the optimal reaction conditions were defined, the scope of this transformation was studied. Scheme 4 contains the 14 compounds obtained by employing this methodology in which hydrosilanes bearing aryl and/or alkyl substituents were generated in 31–90% yield (determined by internal standard on the reaction crude; see the Supporting Information) with TsNH_2_ accounting for all the initial PhI=NTs. Despite the previous reports on the capabilities of this copper catalyst inserting the nitrene units into arene or alkane C–H bonds,^14^ now, the nitrene transfer occurs in an exclusive manner onto the Si–H bond, while the aryl or alkyl groups bonded to Si remain unreacted. Electronic effects do not seem crucial for the reaction outcome, since the use of dimethylarylsilanes bearing OMe or Cl substituents in the aryl ring did not affect the yields into the functionalized silane (see Scheme 4, **1**, **8**, and **9**). When competition experiments were carried out between these three silanes, nearly equimolar mixtures were obtained (Scheme 5). With the caution of a reduced number of experiments, it seems that electronic effects are not crucial in this transformation.

**Figure 2:**
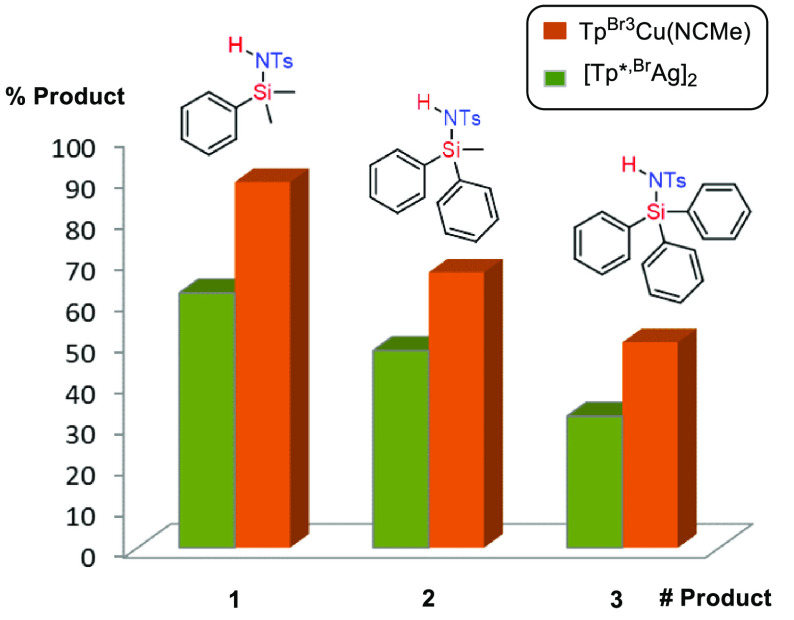
Effect of bulkiness of substituents on the nirene insertion reaction catalyzed by Tp^Br3^Cu(NCMe) and [Tp^*,Br^Ag]_2_ complexes.

The tris-alkyl substituted silanes delivered reasonable yields within the range of 72–75% (compounds **10**, **11**, **13**) for linear alkyl fragments, which turned into lower yields upon increasing the volume of the substituent (31% yield for **12**). The difference augments when the alkyl group displays a certain steric hindrance, as is the case of cyclopentyldimethylsilane (**14**, 58%). A comparison of phenyl- with alkyl-substituted silanes shows that the former is more reactive: it is the case of **1** (90%) and **13** (72%). The functionalization of the Si–H becomes more difficult when increasing the number of aromatic rings: the steric effect surpasses by far the augment of nucleophilicity at the Si–H bond by the action of the aryl groups. Such an effect is found for both catalysts; since the catalytic pocket defined by the Tp^Br3^ and Tp^*,Br^ ligands is quite similar,^15a^ the reactivity is defined by the geometry around the Si–H bond (Figure 2).

We also targeted the evaluation of isotopic effects. Toward that end, we prepared PhMe_2_Si–D and run the catalytic reaction with PhI=NTs. Unfortunately, in all cases, we obtained the protio-derivative, since we observe an N–D exchange with adventitious water (which also originates from the formation of TsNH_2_). Therefore, we could not evaluate the Si–H/Si–D competition experiments.

**Scheme 6:**
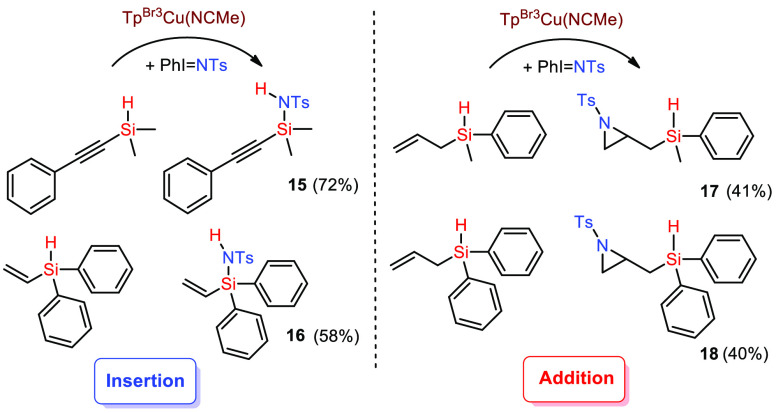
Study of the Tolerance of Other Functional Groups: Insertion vs Addition Reaction Reaction conditions: [Cat]/[PhINTs]/[silane] = 1:20:100, r.t., DCM, 45 min. Yields determined by ^1^H NMR using 1,3,5-trimethoxybenzene as the internal standard. See the Supporting Information for experimental details.

Once the tolerance toward C–H bonds was demonstrated, we studied the compatibility with other functional groups using silanes bearing alkyne or alkene functionalities as well as N-containing silanes. As shown in Scheme 6, when the C–C multiple bond is directly connected to the silicon center, the reaction occurs at the Si–H bond with an effective formation of the Si–N moiety. However, with allylic groups as substituents, the preferred transformation is the aziridination of the alkene. In the context of nitrene transfer chemistry, it is well established that the nucleophilicity of the substrate governs the reactivity. Therefore, the olefin must be more reactive than the Si–H bond. However, if the C=C bond is hindered, as is the case of the substrate leading to **16**, the reactivity is reversed.

Regarding the use of 2-(dimethylsilyl)pyridine and *N*,*N*-diethyl-1,1-dimethylsilanamine as representative examples of N-containing silanes, the reaction proceeded toward the formation of zwitterionic, N–N containing compounds, following the reactivity recently reported by our group^17^ with no functionalization of the Si–H bond being observed (eqs 1 and 2).
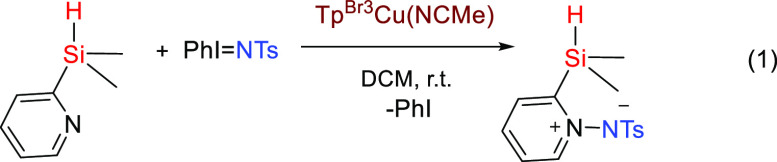
1
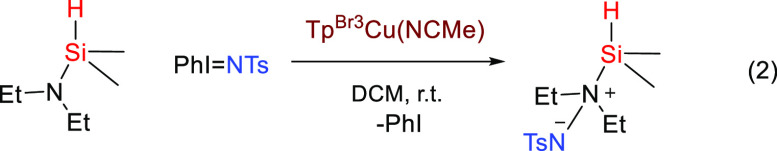
2

**Figure 3:**
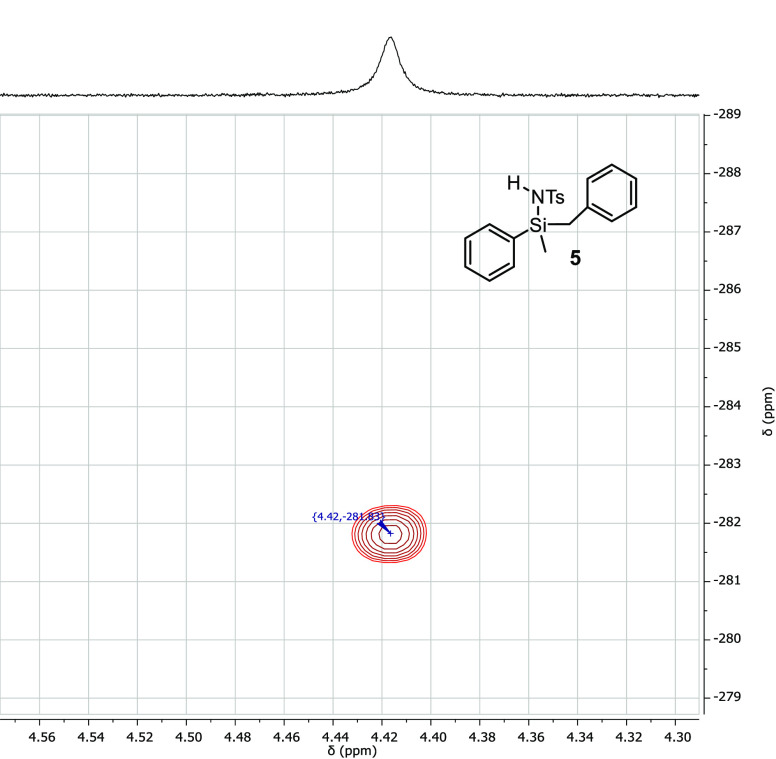
^1^H–^15^N HSQC NMR spectrum for **5** (CDCl_3_).

Given the novelty of this transformation and the lack of ^15^N NMR data for compounds bearing Si–N bonds, we have carried out NMR experiments toward that end. Thus, the resonance for the Si–N nucleus has been detected through INEPT and/or HSQC experiments in the vicinity of −285 ppm, referred to as nitromethane, for representative compounds (**1**, **4**–**6**, **8**, **10**, and **11**). Figure 3 displays the 2D spectrum of compound **5** with δ = −281.8 ppm. ^29^Si NMR data has also been collected, and the chemical shift of the new compounds is in the interval of +15 ppm (trisalkyl substituted) to ca. 0 to −10 ppm when incorporating the aryl substituents (see the Supporting Information).

**Scheme 7:**
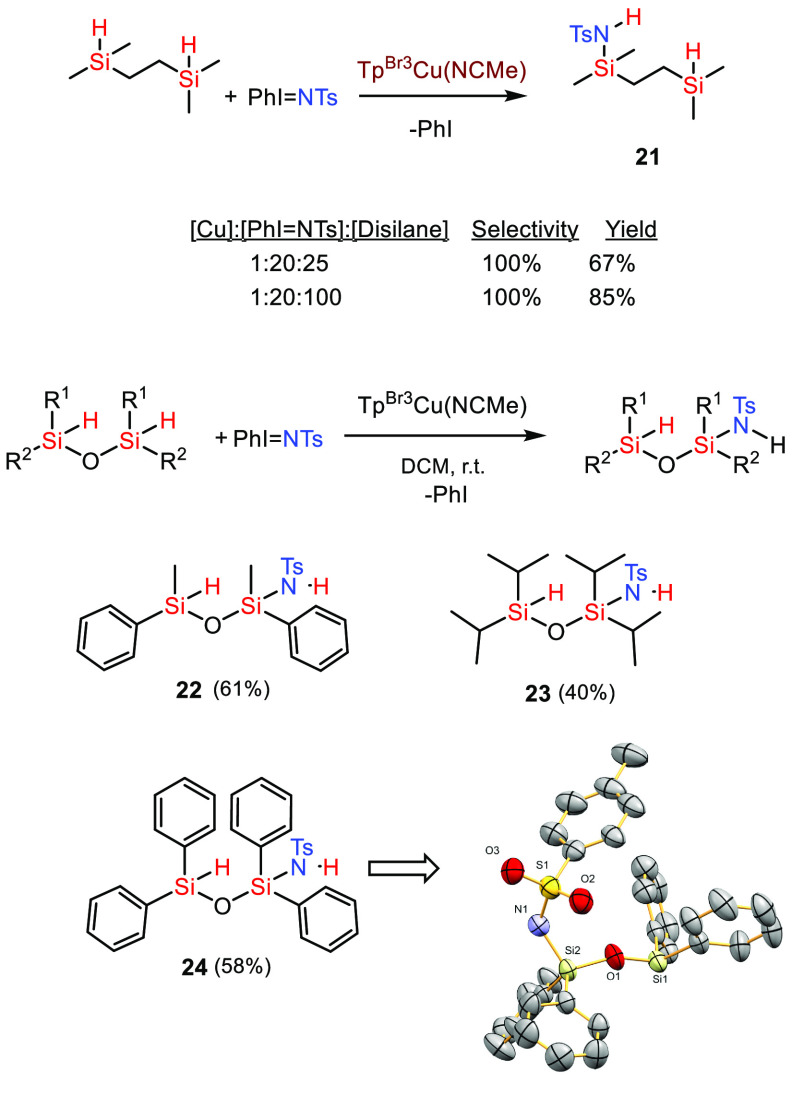
Functionalization of Disilane and Siloxane Compounds by Nitrene Insertion into the Si–H Bond Reaction conditions: [Cat]/[PhINTs]/[silane] = 1:20:100, r.t., DCM, 45 min. Yields determined by ^1^H NMR using 1,3,5-trimethoxybenzene as the internal standard. See the Supporting Information for experimental details.

The formation of Si–N bonds by this new methodology is not restricted to silanes of type HSiR_3_ but also works for dihydrosilanes H_2_SiR_2_. Despite the availability of two Si–H bonds, we have only observed the products derived from the monoinsertion of the nitrene group, no matter the ratio of reactants employed (Table 1). To complete the array of silanes capable of being functionalized with this tactic, we have employed disilane and siloxane compounds, which are also unreported toward that end (Scheme 7). The presence of two Si–H bonds per molecule in these substrates does not influence the reaction outcome with one unique nitrene unit being incorporated in each case. A similar observation has been reported for a rhodium-catalyzed C–H amination process.^18^ Attempts to force a second incorporation upon adding more PhI=NTs did not give the targeted product. We believe that the competitive formation of TsNH_2_ from adventitious water is a favored pathway. In agreement with this assumption, the yield into **21** is diminished when a 1:20:25 ratio of catalyst, PhI=NTs, and disilane is employed, compared with the same experiment carried out with a 1:20:100 molar ratio of catalyst and reactants.

**Table 1:**
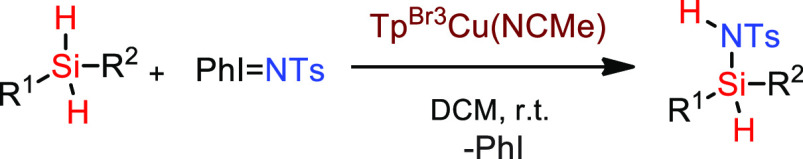

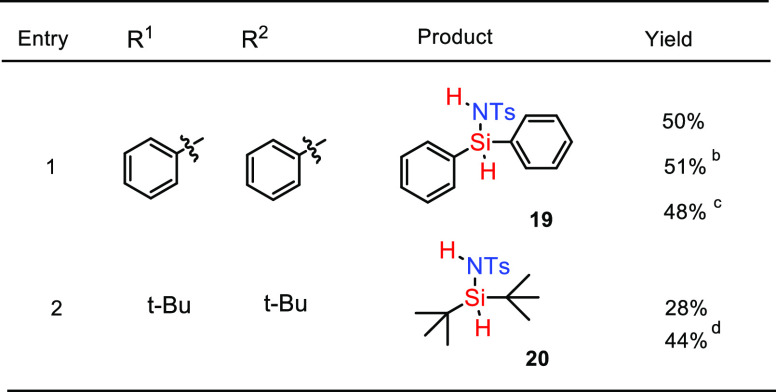
Reaction of Dihydrosilanes and PhI=NTs Using Tp^Br3^Cu(NCMe) as Catalyst^a^

a See the Supporting Information for experimental details.

b [PhI=NTs]/[silane] = 1:2.5.

c [PhI=NTs]/[silane] = 1:1.25.

d [Tp^*,Br^Ag]_2_ complex as catalyst.

Yields are moderate to high, and the experiments are performed at room temperature. Compound **24** was characterized by X-ray diffraction to completely assess the formation of the Si–N bond in this siloxane skeleton. The functionalization of siloxanes with this tactic is remarkable since these molecules are unit models for biocompatible polymers.

### Density Functional Theory (DFT) Studies

**Scheme 8:**
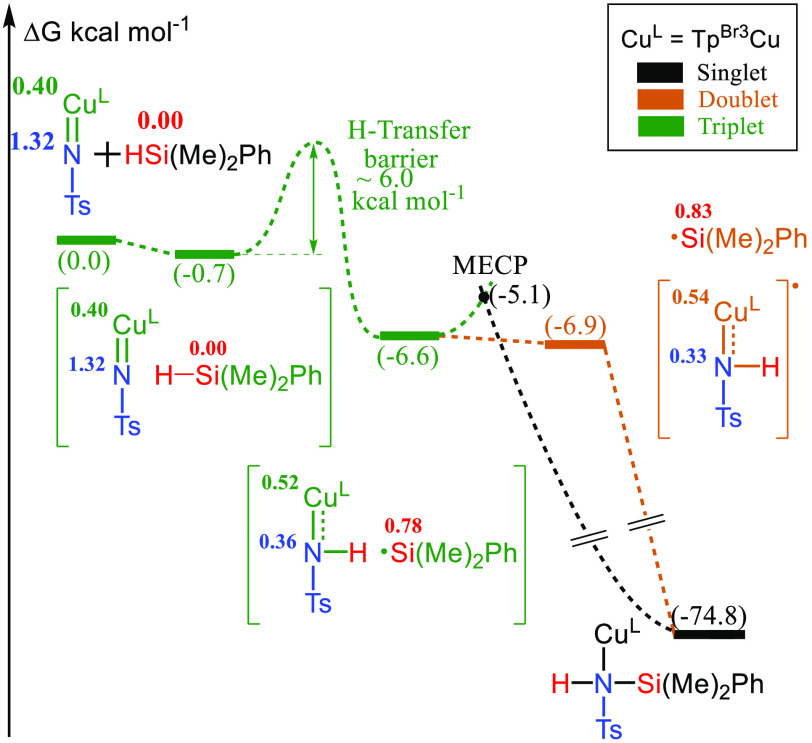
Computed Mechanism for the Nitrene Transfer to the Silane Numbers within the parentheses correspond to the computed relative free energies in kcal/mol. Numbers near the Cu, N, and Si atoms correspond to their computed Mulliken spin density.

Given the lack of precedents in this nitrene transfer to Si–H bonds, we carried out DFT calculations (B3LYP-D3, in DCM solvent; full details in the Supporting Information) in order to clarify the mechanism. A data set collection of the computational results is available in the ioChem-BD repository^19^ and can be accessed through https://doi.org/10.19061/iochem-bd-1-233. It is well-known that the interaction of the Tp^Br3^Cu core and PhI=NTs leads to nitrene intermediates Tp^Br3^Cu(NTs)^15b,17^ with the triplet state being more favorable. The reaction between Tp^Br3^Cu(NTs) and dimethylphenylsilane was therefore chosen, and the computed mechanism is shown in Scheme 8. The reaction proceeds through a homolytic cleavage of the Si–H bond in a process similar to the rebound mechanism reported by Cundari, Stavropoulous, and co-workers for C–H amination processes.^11b^ The Tp^Br3^Cu(NTs) starting species is in a triplet ground state with one unpaired electron fully on nitrogen and the second one shared between nitrogen and copper. This complex forms an adduct, −0.7 kcal mol^–1^ below, with the silane. This adduct can undergo the homolytic cleavage of the Si–H bond with a barrier below 6 kcal mol^–1^ in the key step of the mechanism. The resulting intermediate has one unpaired electron on the silyl and another one on the Cu–N moiety. It can rearrange to products either by separation and recombination (rebound mechanism) or through a triplet/singlet minimum energy crossing point (MECP). Both alternatives have very low barriers.

## Conclusions

A novel strategy for the formation of silicon–nitrogen bonds has been developed, employing copper catalysis for the insertion of a nitrene group into the Si–H bond of mono- and dihydrosilanes, disilanes, and siloxanes. At variance with previous methods, the hydrogen atom of the parent Si–H bond is maintained. DFT studies have shown that the process takes place through Si–H homolytic cleavage and rebound with the Si-centered radical. This is the first example of the formation of Si–N bonds by this methodology, which takes place under very mild conditions. This strategy provides a new window for the functionalization of silicon-based structures, including macromolecules of the silicone type.

## Experimental Section

### General Catalytic Experiment

In a Schlenk tube, under an inert atmosphere, the catalyst (0.01 mmol) was dissolved in deoxygenated solvent (6 mL) and the silane was added (1 mmol). PhI=NTs (0.2 mmol) was added in one portion, and the mixture was stirred at room temperature for 1.5 h. The solvent was removed under reduced pressure, and the reaction crude was analyzed by NMR spectroscopy. The residue was purified through a column of C18-reversed phase silica gel (eluent MeCN). Single crystals were obtained by crystallization in Et_2_O/hexane (2:1).

## Supporting Information Available

All procedures and characterization data, computational data, and Cartesian coordinates of the optimized structures (PDF)

## Accession Codes

CCDC 2124779, 2124782, 2124783, 2124786–2124791, 2124794, and 2125281 contain the supplementary crystallographic data for this paper. These data can be obtained free of charge via www.ccdc.cam.ac.uk/data_request/cif, or by emailing data_request@ccdc.cam.ac.uk, or by contacting The Cambridge Crystallographic Data Centre, 12 Union Road, Cambridge CB2 1EZ, UK; fax: +44 1223 336033.

## Dedication

Dedicated to Maurice “Brook” Brookhart on occasion of his 80th birthday.
