# Supplementary material for: Introducing the Catalytic Amination of Silanes via Nitrene Insertion
Source: J Am Chem Soc. 2022 Jun 1;144(23):10608–14. doi: 10.1021/jacs.2c03739 (PMC9490852; doi:10.1021/jacs.2c03739)
Supplement: Supplementary file 1 — ja2c03739_si_001.pdf [file ja2c03739_si_001.pdf]

Introducing the Catalytic Amination of Silanes via Nitrene Insertion

Anabel M. Rodríguez,† Jorge Pérez-Ruíz,† Francisco Molina,† Ana Poveda,§ Raúl Pérez-Soto,‡ Feliu Maseras,‡,* M. Mar Díaz-Requejo,†,* Pedro J. Pérez.†,*

Table of contents:

1. General Information S2

2. Synthesis of substrates S3

3. General procedure for catalytic experiments S8

4. Products characterization S11

5. NMR spectra of products S23

6. References for experimental section S54

7. Computational data S56

8. References for computational data S279

1. General information

All air– and moisture–sensitive manipulations were carried out with standard Schlenk techniques under nitrogen atmosphere or in a glovebox (MBRAUN UNILAB). Solvents were purchased from commercial sources, dried by distillation under nitrogen atmosphere using the suitable drying agent and deoxygenated immediately before their use. Reagents were acquired from Aldrich, Alfa Aesar, Acros Organics and Fluorochem, and used without any further purification. Commercially unavailable silanes were obtained following the detailed procedures in this section. The metallic complexes (Tp^xM),[1,2] IPrMCl[3,4]) and the nitrene precursor (PhINTs[5]) were synthesized by literature procedures.

NMR spectra were recorded on the Agilent 400MR and Agilent 500DD2 spectrometers as solutions at 298 K and Bruker Avance III HD 400 MHz or 500 MHz spectrometer at ambient temperature. Chemical shifts (δ) were referenced to internal solvent resonances and reported relative to TMS (tetramethylsilane) for ^{1}H and ^{13}C NMR.

^{15}N NMR experiments (^{15}N INEPT and ^{1}H-^{15}N HSQC) were carried out at 298 K on a Bruker NEO-400 spectrometer, equipped with a Z-axis gradient iProbe (BBF/H/D). ^{15}N chemical shifts are referred to CH_3NO_2 as IUPAC secondary standard.[6] The $^{1}J_{NH}$ value employed for both INEPT and HSQC experiments was determined from a previous non-decoupled HSQC on compound **11**, and was set to 77 Hz for all experiments. ^{15}N INEPT experiments were run only when enough sample was available. For all the samples, the direct ^{1}H-^{15}N HSQC were recorded using the standard BRUKER pulse program hsqcetgpsi2. A matrix of 1024 (F2) x 128 (F1) data points was acquired for every sample for a total window of 10 (^{1}H) x 100 (^{15}N) ppm. The number of scans was set from 8 to 128, according to the sample concentration. These acquired data were transformed to a 2096 x 1024 matrix, using cuadratic sinebell window function with a sine bell shift SSB=2.

High resolution mass spectroscopy experiments were carried out at the Centre of Research Technology and Innovation of the University of Seville (CITIUS) and at the Centre for Research in Sustainable Chemistry (CIQSO) of the University of Huelva. X-Ray diffraction studies were performed at the Centre for Research in Sustainable Chemistry (CIQSO) of the University of Huelva.

2. Synthesis of substrates

2.1. Synthesis of tert-butyldiphenylsilane[7]

In a Schlenk tube under inert atmosphere tert-butyl(chloro)diphenylsilane (6 mmol, 1.65 g, 1.5 mL) was dissolved in dry Et_2O (20 mL) and the mixture cooled at 0 °C. The lithium aluminium hydride solution (4M, 6 mmol) was added dropwise. The reaction mixture was stirred at room temperature overnight. Then, H_2O (20 mL) was added dropwise to quench the reaction. Concentrated hydrochloric acid was added dropwise until two layers separated. The organic phases were washed with saturated $NaHCO_3$ until they gave basic pH, followed by washing with brine (20 mL). The organic phase was dried over Na_2SO_4, filtered and the residue was evaporated by reduced pressure. The product was identified with NMR spectroscopy by comparison with reported data.

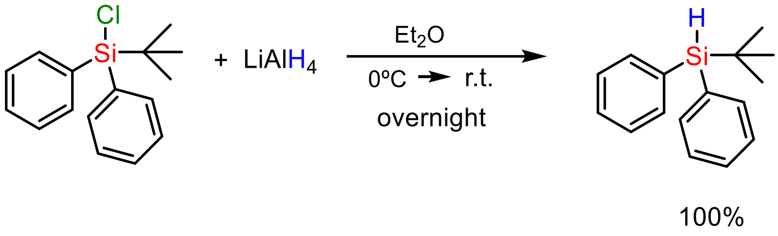

2.2. Synthesis of Silane A[7]

A Schlenk tube under inert atmosphere was charged with dry THF (10 mL) and vinylmagnesium bromide solution in Et_2O (10 mmol, 10 mL), and cooled at 0 °C. Then, diphenylchlorosilane (10 mmol, 2.2 g, 2.1 mL) was added dropwise. The mixture was stirred overnight at room temperature and poured carefully into ice water. The mixture was extracted with Et_2O (2 x 15 mL). The organic phases were washed with brine (15 mL), dried over Na_2SO_4, filtered and evaporated under reduced pressure. Finally, the product was purified through a column of silica gel (eluent with 1:6 EtOAc/hexane). This procedure was applied to the silanes shown below.

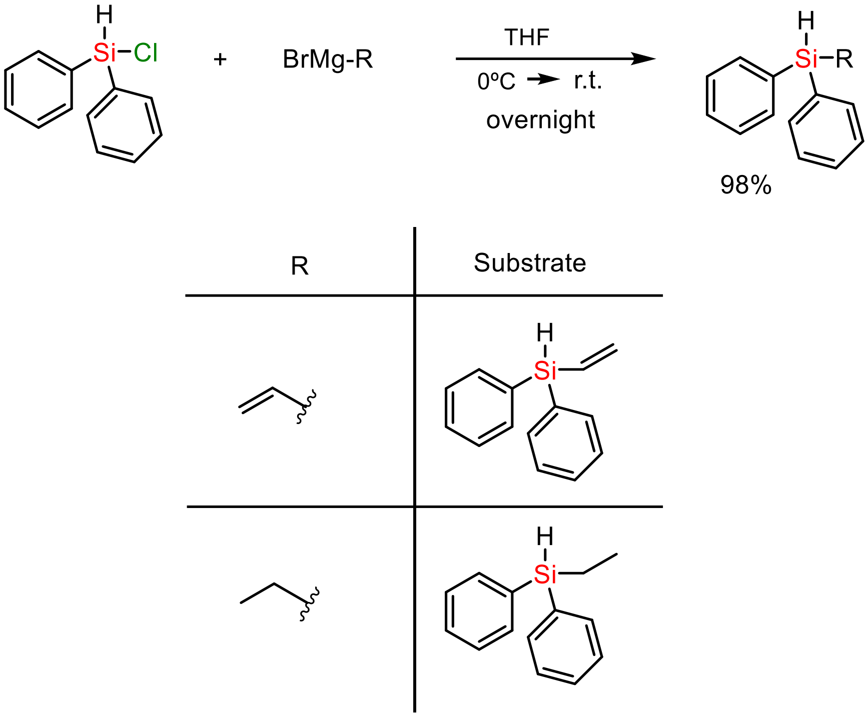

2.3. Synthesis of allyl(diphenyl)silane[8]

At -78 °C, in a Schlenk tube under inert atmosphere, to a suspension LiCl (5 mmol, 212 mg) in THF (10 mL) was added an allylmagnesium bromide solution in Et_2O (1M, 5 mmol, 5 mL), followed by diphenylsilane (5 mmol, 920 µL). The reaction mixture was stirred at the same temperature for 4 h. A solution of NH_4Cl (5 mL) was added to quench the reaction. The resulting mixture was filtered through Celite, adding Et_2O. The solution was dried over Na_2SO_4, filtered and evaporated under reduced pressure. Finally, the product was purified through a column of silica gel (eluent hexane).

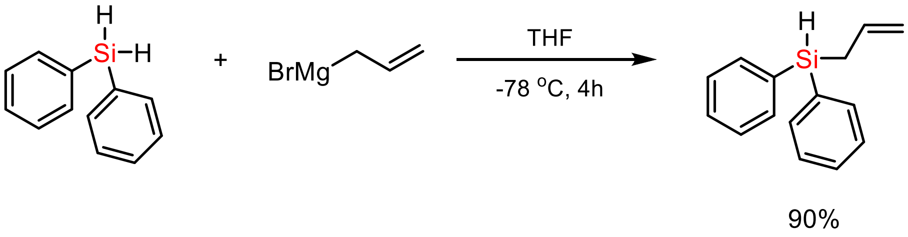

2.4. Synthesis allyl(phenyl)(methyl)silane[8]

To a suspension of LiCl (5 mmol, 212 mg) in THF (10 mL) was added an allylmagnesium bromide solution in Et_2O (1M, 5 mmol, 5 mL), followed by methylphenylsilane (5 mmol, 920 µL) under inert atmosphere. The reaction mixture was

stirred at room temperature for 1 h, before a solution of NH_4Cl (5 mL) was added to finish the reaction. The mixture was filtered through Celite, adding Et_2O. The residue was dried over Na_2SO_4, filtered and evaporated under reduced pressure. Finally, the product was purified through a column of silica gel (eluent hexane).

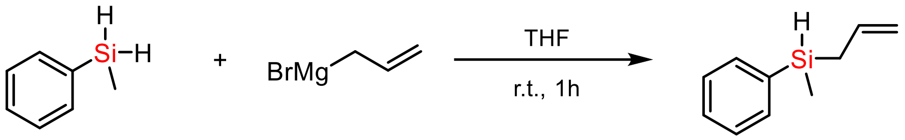

98%

2.5. Synthesis of benzyl(diphenyl)silane and benzyl(methyl)(phenyl)silane[8]

Following the previous procedure, at -30 °C, LiCl (6 mmol, 254.4 mg) and benzylmagnesium bromide (2M in diethyl ether, 6 mmol, 3 mL) were reacted with the corresponding silane (6 mmol) under inert atmosphere. Products were purified by silica gel chromatography with hexane as eluent.

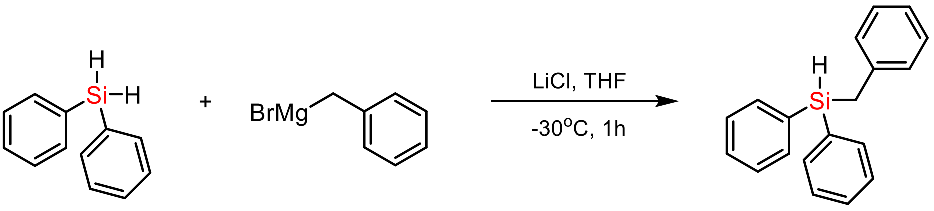

98%

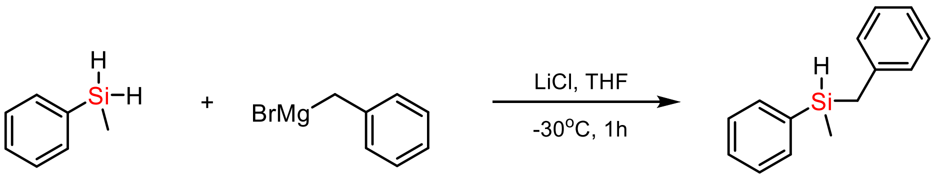

80%

2.6. Synthesis of silane B[8]

A Schlenk tube under inert atmosphere was charged with dry THF (10 mL) and the Grignard reagent (10 mL, 1M Et_2O), and cooled at 0 °C. Then, dimethylchlorosilane (8 mmol, 890 μL) was added dropwise over 15 min. The mixture was stirred at room temperature overnight. A solution of NH_4Cl (10 mL) was added to quench before extracting with Et_2O (3 x 15 mL). The organic phases were collected and washed with

H_2O (20 mL) and brine (20 mL). The organic solution was dried over Na_2SO_4, filtered and evaporated under reduced pressure, and the product was purified through a column of silica gel (eluent hexane). This procedure was applied to the silanes shown below.

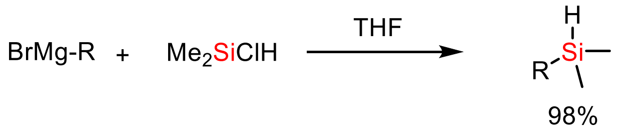

R	Substrate	Ref.
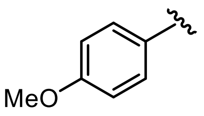	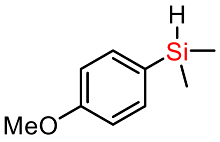	9
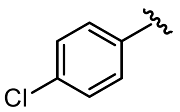	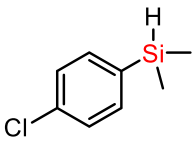	9
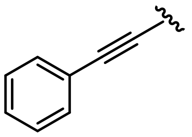	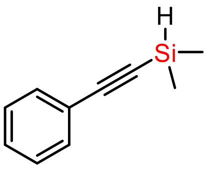	9
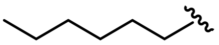	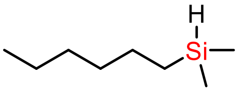	10
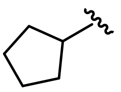	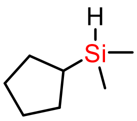	This work

2.7. Synthesis of disiloxanes

A) $Ph_2HSiOSiHPh_2$[11,12]

In a 25 mL round-bottomed flask was added $InBr_3$ (0.33 mmol), tetrahydrofuran (10 mL) and silane (6.7 mmol). The mixture was stirred for 4 h at room temperature keeping the flask opened to air, since gas evolution (hydrogen) takes place. Once the reaction was finished, the solvent was removed under reduced pressure. Then, the product was purified through a column of silica gel (100-200 mesh, eluent hexane).

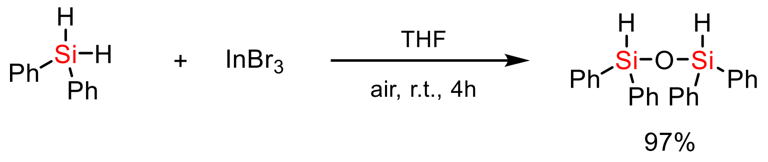

B) PhMeHSiOSiHMePh[13, 14]

To a solution of PhMeSiHCl (5.1 mmol) in acetonitrile (10 mL) was added H_2O (2.8 mmol, 50 μL), and the solution was stirred for 1 h at room temperature. All volatiles were then removed under reduced pressure, and the resulting residue was extracted with pentane (20 mL).

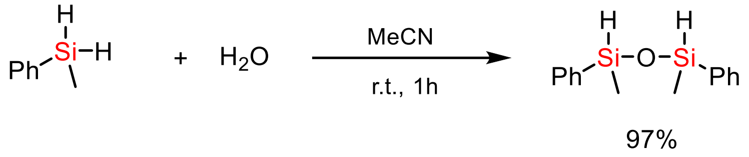

3. General procedure for catalytic experiments

3.1. General transfer reaction of PhINTs to silanes catalyzed by $Tp^{Br3}Cu(NCMe)$

In a Schlenk tube, under inert atmosphere, the catalyst (0.01 mmol) was dissolved in deoxygenated solvent (6 mL) and the silane was added (1 mmol). PhINTs (0.2 mmol) was added in one portion, and the mixture was stirred at room temperature for 1.5 h. The solvent was removed under reduced pressure and the reaction crude was analyzed by NMR spectroscopy. The residue was purified through a column of C_{18}-reversed phase silica gel (eluent MeCN). Single crystals were obtained by crystallization in Et_2O:Hexane (2:1).

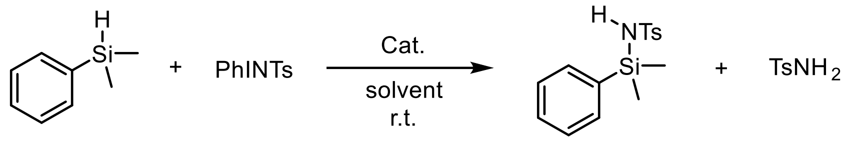

3.2. Catalyst screening

The scope of catalytic precursors was carried out following the above general procedure.

Figure S1: Catalyst screening[a]

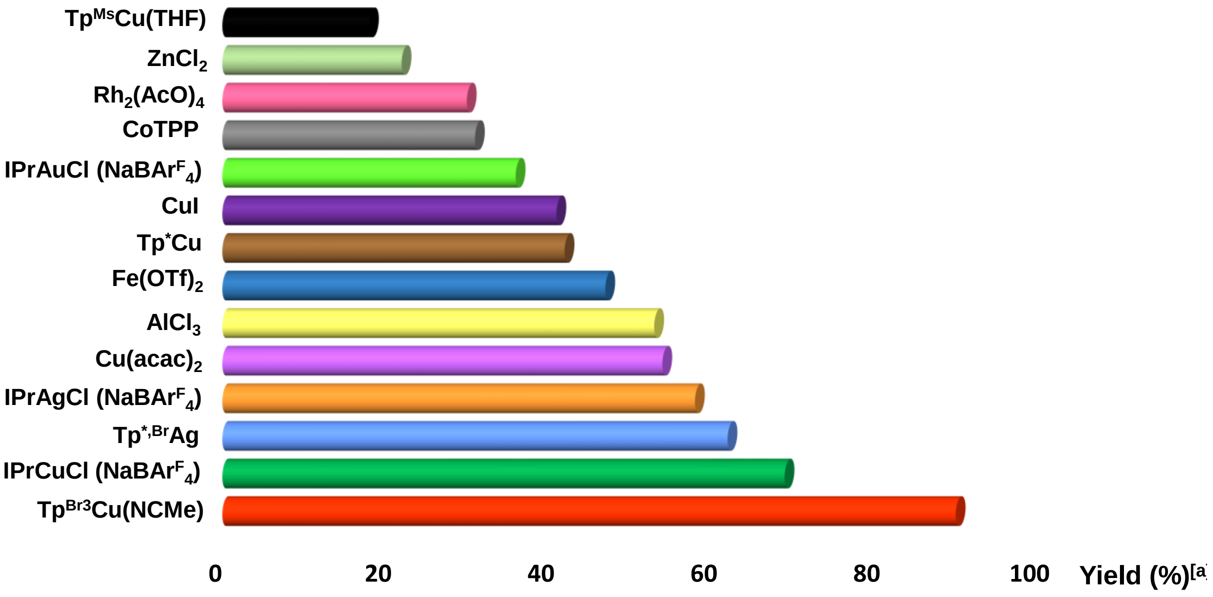

Reaction conditions: [Cat]:[PhINTs]:[silane] = 1:20:100, r.t., DCM, 45 min.

[a] Dertemined by ^{1}H NMR using 1,3,5-trimethoxybenzene as internal standard

3.3. Solvent effect

The effect of the reaction solvent was studied following the general procedure, with the results shown below:

Table S1: Solvent effect[a]

Catalyst	Solvent	Yield (%)[b]
$Tp^{Br3}Cu(NCMe)$	DCM	90
$Tp^{Br3}Cu(NCMe)$	MeCN	44
$Tp^{Br3}Cu(NCMe)$	DCE	39
$Tp^{Br3}Cu(NCMe)$	Acetone	<1

[a]Reaction conditions: [Cat.]:[PhINTs]:[silane]=1:20:100, r.t., 45 min.
[b]Determined by ^{1}H NMR using 1,3,5-trimethoxybenzene as internal standard

3.4. Study of proportion [Cat.]:[PhINTs]:[silane]

The effect of [Cat]:[PhINTs]:[silane] was studied following the general procedure, using different amounts of silane and PhINTs.

Table S2: [Cat]:[PhINTs]:[silane][a]

Catalyst	Cat:PhINTs:silane	Yield (%)[b]
$Tp^{Br3}Cu(NCMe)$	1:20:100	90
$Tp^{Br3}Cu(NCMe)$	1:20:40	59
$Tp^{Br3}Cu(NCMe)$	1:20:200	69
$Tp^{Br3}Cu(NCMe)$	1:50:100	21

[a]Reaction conditions: DCM, r.t., 45 min.
[b]Determined by ^{1}H NMR using 1,3,5-trimethoxybenzene as internal standard

3.5. Nitrene source

This effect was studied following the general procedure using different nitrene sources and addition ratios:

Table S3: Nitrene source[a]

Nitrene source	Procedure	Yield (%)[b]
PhINTs	"in situ"	6
PhINTs	4 portion	44
PhINTs	one portion	90
Cloramine-T	one portion	17

[a]Reaction conditions: [$Tp^{Br3}Cu(NCMe)$]:[PhINTs]:[silane]=1:20:100, DCM, r.t., 45 min.
[b]Determined by ^{1}H NMR using 1,3,5-trimethoxybenzene as internal standard

4. Products characterization.

Product 1[15]

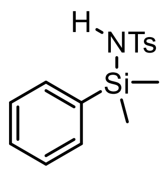

^{1}H NMR (400 MHz, $CDCl_3$): δ = 7.58 (d, *J* = 8.0 Hz, 2H), 7.52 (m, 2H), 7.42 (m, 1H), 7.35 (m, 2H), 7.18 (d, *J* = 8.0 Hz, 2H), 4.68 (s, 1H), 2.39 (s, 3H), 0.57 (s, 6H).

^{13}C{^{1}H} NMR (100 MHz, $CDCl_3$): δ = 142.8, 140.5, 135.1, 133.8,133.1, 130.4, 129.5, 129.4, 128.2, 127.8, 126.3, 21.6, -1.2.

^{29}Si NMR (100 MHz, $CDCl_3$): δ = 1.02.

^{15}N NMR (81 MHz, $CDCl_3$): δ = -280.56.

HRMS ESI [M+Na$^+$] Calculated for $C_{15}H_{19}O_2NNaSSi$ = 328.0798. Found = 328.0800.

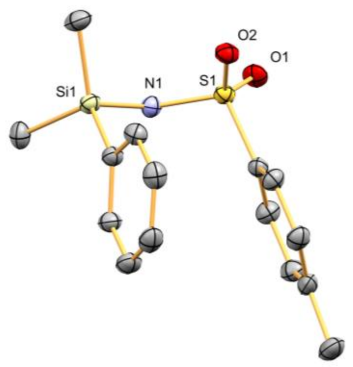

ORTEP for **1.**

Product 2

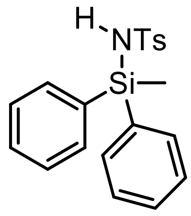

^{1}H NMR (400 MHz, $CDCl_3$): δ = 7.54 (m, 4H), 7.47 (d, *J* = 8.3 Hz, 2H), 7.43 (m, 2H), 7.36 (m, 4H), 7.13 (d, *J* = 8.1 Hz, 2H), 4.72 (s, 1H), 2.38 (s, 3H), 0.88 (s, 3H).

^{13}C{^{1}H} NMR (100 MHz, $CDCl_3$): δ = 142.9, 140.2, 134.7, 133.4, 130.5, 129.4, 128.2, 126.4, 21.6, -2.3.

^{29}Si NMR (100 MHz, $CDCl_3$): δ = -7.92.

HRMS ESI [M+Na$^+$] Calculated for $C_{20}H_{21}O_2NNaSSi$ = 390.0954. Found = 390.0952.

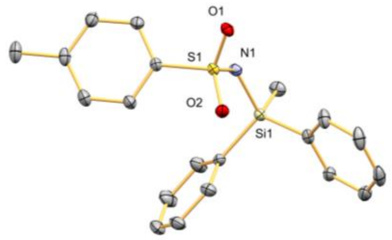

ORTEP for **2.**

Product 3

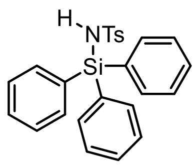

^{1}H NMR (400 MHz, $CDCl_3$): δ = 7.62 (m, 6H), 7.47 (m, 3H), 7.38 (m, 6H), 7.27 (m, 2H), 7.05 (m, 2H), 4.92 (s, 1H), 2.35 (s, 3H).

^{13}C{^{1}H} NMR (100 MHz, $CDCl_3$): δ = 142.8, 140.1, 136.0, 131.7, 130.7, 129.3, 128.2, 126.5, 21.6.

^{29}Si NMR (100 MHz, $CDCl_3$): δ = 1.8.

HRMS ESI [M+Na$^+$] Calculated for $C_{25}H_{23}O_2NNaSSi$ = 452.1111. Found = 452.1105.

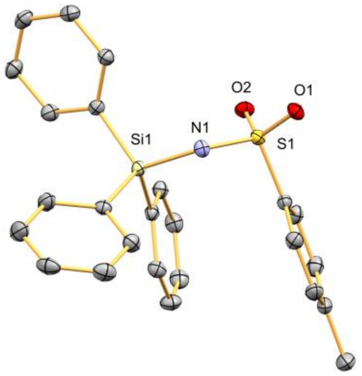

ORTEP for **3.**

Product 4

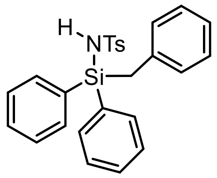

^{1}H NMR (400 MHz, $CDCl_3$): δ = 7.42 (m, 6H), 7.30 (m, 6H), 7.13 (m, 3H), 7.06 (m, 2H), 6.95 (m, 2H), 4.58 (s, 1H), 2.94 (s, 2H), 2.36 (s, 3H).

^{13}C{^{1}H} NMR (100 MHz, $CDCl_3$): δ = 142.8, 140.1, 136.5, 135.5, 131.4, 130.7, 129.3, 129.3, 128.5, 128.1, 126.4, 125.2, 23.7, 21.6.

^{29}Si NMR (100 MHz, $CDCl_3$): δ = -8.5.

^{15}N NMR (81 MHz, $CDCl_3$): δ = -282.81.

HRMS ESI [M+Na$^+$] Calculated for $C_{26}H_{25}O_2NNaSSi$ = 466.1267. Found = 466.1260.

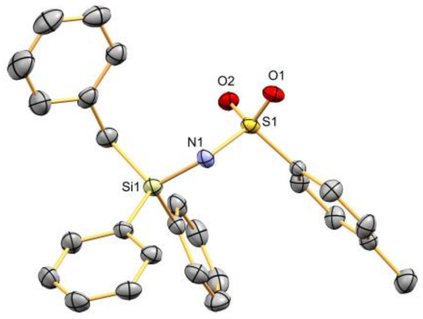

ORTEP for **4.**

Product 5

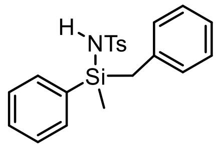

^{1}H NMR (400 MHz, $CDCl_3$): δ = 7.49 (m, 2H), 7.46 (m, 2H), 7.33 (m, 2H), 7.16 (m, 4H), 7.09 (m, 1H), 7.02 (m, 1H), 6.99 (m, 2H), 4.37 (s, 1H), 2.70 (d, J = 14 Hz, 1H), 2.58 (d, J = 14 Hz, 1H), 2.38 (s, 3H), 0.49 (s, 3H).

^{13}C{^{1}H} NMR (100 MHz, $CDCl_3$): δ = 142.9, 140.4, 137.1, 134.3, 133.5, 130.6, 129.5, 128.7, 128.1, 128.0, 126.3, 125.1, 24.9, 21.6, -3.6.

^{29}Si NMR (100 MHz, $CDCl_3$): δ = -2.5.

^{15}N NMR (81 MHz, $CDCl_3$): δ = -281.83.

HRMS ESI [M+Na$^+$] Calculated for $C_{21}H_{23}O_2NNaSSi$ = 404.1111. Found = 404.1109.

Product 6

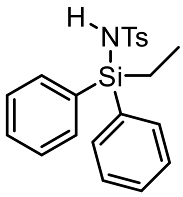

^{1}H NMR (400 MHz, $CDCl_3$): δ = 7.54 (m, 4H), 7.44 (m, 2H), 7.36 (m, 6H), 7.07 (m, 2H), 4.74 (s, 1H), 2.36 (s, 3H), 1.40 (q, J = 7.9 Hz, 2H), 1.07 (t, J = 8 Hz, 3H).

^{13}C{^{1}H} NMR (100 MHz, $CDCl_3$): δ = 142.7, 140.2, 135.2, 134.4, 132.3, 130.5, 130.0, 129.9, 129.3, 128.2, 128.0, 126.4, 21.6, 7.0, 5.7.

^{29}Si NMR (100 MHz, $CDCl_3$): δ = -2.6.

^{15}N NMR (81 MHz, $CDCl_3$): δ = -282.41.

HRMS ESI [M+Na$^+$] Calculated for $C_{21}H_{23}O_2NNaSSi$ = 404.1111. Found = 404.1106.

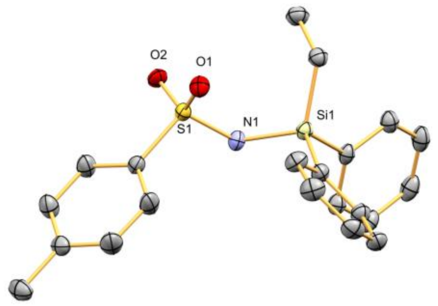

ORTEP for **6.**

Product 7

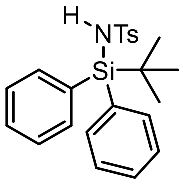

^{1}H NMR (400 MHz, $CDCl_3$): δ = 7.64 (m, 4H), 7.44 (m, 2H), 7.34 (m, 4H), 7.22 (d, J = 8.2 Hz, 2H), 7.03 (d, J = 8.1 Hz, 2H), 4.78 (s, 1H), 2.35 (s, 3H), 1.04 (s, 9H).

^{13}C{^{1}H} NMR (100 MHz, $CDCl_3$): δ = 142.5, 140.2, 136.3, 134.9, 131.1, 130.3, 129.8, 129.2, 127.9, 127.8, 126.4, 27.4, 21.6, 18.7.

^{29}Si NMR (100 MHz, $CDCl_3$): δ = 12.9.

HRMS ESI [M+Na$^+$] Calculated for $C_{23}H_{27}O_2NNaSSi$= 432.1424. Found = 432.1417.

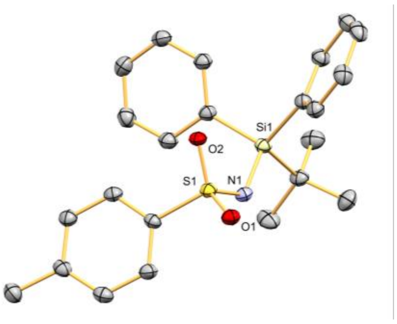

ORTEP for **7.**

Product 8

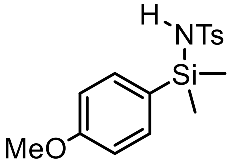

^{1}H NMR (400 MHz, $CDCl_3$): δ = 7.80 (d, *J* = 8.3 Hz, 2H), 7.52 (m, 2H), 7.30 (d, *J* = 8.1 Hz, 2H), 6.93 (m, 2H), 4.95 (s, 1H), 3.82 (s, 3H), 2.43 (s, 3H), 0.38 (s, 6H).

^{13}C{^{1}H} NMR (100 MHz, $CDCl_3$): δ = 161.0, 143.7, 139.2, 134.8, 130.3, 129.8, 126.6, 113.7, 55.2, 21.7, 0.2.

^{29}Si NMR (100 MHz, $CDCl_3$): δ = 0.8.

^{15}N NMR (81 MHz, $CDCl_3$): δ = -286.02.

HRMS ESI [M+Na$^+$] Calculated for $C_{16}H_{21}O_2NNaSSi$ = 358.0904. Found = 358.0900.

Product 9

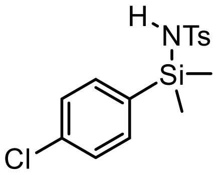

^{1}H NMR (400 MHz, $CDCl_3$): δ = 7.52 (d, *J* = 8.3 Hz, 2H), 7.40 (m, 2H), 7.26 (m, 2H), 7.14 (d, *J* = 8.0 Hz, 2H), 5.05 (s, 1H), 2.37 (s, 3H), 0.50 (s, 6H).

^{13}C{^{1}H} NMR (100 MHz, $CDCl_3$): δ = 142.9, 140.4, 136.7, 135.3, 134.6, 133.5, 129.4, 128.3, 126.2, 21.5, -1.4.

^{29}Si NMR (80 MHz, $CDCl_3$): δ = 0.8.

HRMS ESI [M+Na$^+$] Calculated for $C_{15}H_{18}O_2NClNaSSi$= 362.0408. Found = 362.0405.

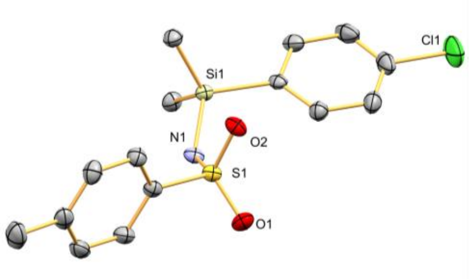

ORTEP for **9.**

Product 10

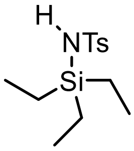

^{1}H NMR (400 MHz, $CDCl_3$): δ = 7.76 (d, *J* = 8.3 Hz, 2H), 7.25 (d, *J* = 8.3 Hz, 2H), 4.81 (s, 1H), 2.40 (s, 3H), 0.89 (m, 9H), 0.71 (m, 6H).

^{13}C{^{1}H} NMR (100 MHz, $CDCl_3$): δ = 142.7, 141.1, 129.5, 126.2, 21.6, 6.6, 4.4.

^{29}Si NMR (100 MHz, $CDCl_3$): δ = 14.69.

^{15}N NMR (81 MHz, $CDCl_3$): δ = -286.92.

HRMS ESI [M+1] Calculated for $C_{13}H_{24}O_2NSSi$= 286.1292. Found = 286.1293.

Product 11

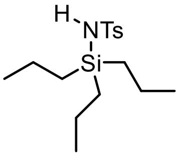

^{1}H NMR (400 MHz, $CDCl_3$): δ = 7.75 (d, *J* = 8.3 Hz, 2H), 7.24 (d, *J* = 8.3 Hz, 2H), 4.76 (s, 1H), 2.40 (s, 3H), 1.29 (m, 6H), 0.90 (m, 9H), 0.69 (m, 6H).

^{13}C{^{1}H} NMR (100 MHz, $CDCl_3$): δ = 142.6, 141.2, 129.4, 126.2, 21.5, 18.2, 16.7, 16.1.

^{29}Si NMR (100 MHz, $CDCl_3$): δ = 10.5.

^{15}N NMR (81 MHz, $CDCl_3$): δ =-285.97.

HRMS ESI [M+Na^{+}] Calculated for $C_{16}H_{29}O_2NNaSSi$= 350.1580. Found = 350.1576.

Product 12

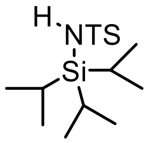

^{1}H NMR (400 MHz, $CDCl_3$): δ = 7.78 (d, *J* = 8.4 Hz, 2H), 7.27 (d, *J* = 7.8 Hz, 2H), 4.12 (s, 1H), 2.41 (s, 3H), 1.28 (h, *J* = 8 Hz, 3H), 1.05 (d, *J* = 7.4 Hz, 18H).

^{13}C{^{1}H} NMR (100 MHz, $CDCl_3$): δ = 142.7, 141.0, 129.5, 126.3, 21.6, 18.1, 12.0.

^{29}Si NMR (100 MHz, $CDCl_3$): δ = 13.3.

HRMS ESI [M+Na^{+}] Calculated for $C_{16}H_{29}O_2NNaSSi$ = 350.1580. Found = 350.1575.

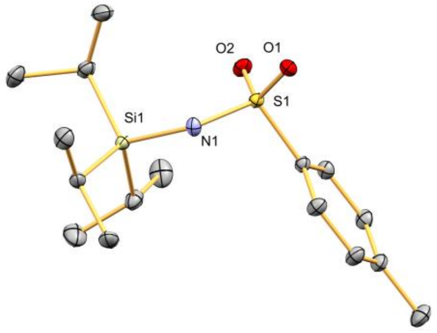

ORTEP for **12.**

Product 13

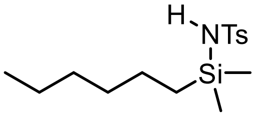

^{1}H NMR (400 MHz, $CDCl_3$): δ = 7.75 (d, *J* = 8.3 Hz, 2H), 7.26 (d, *J* = 8.2 Hz, 2H), 4.73 (s, 1H), 2.41 (s, 3H), 1.22 (m, 8H), 0.86 (m, 3H), 0.69 (m, 2H), 0.21 (s, 6H).

^{13}C{^{1}H} NMR (100 MHz, $CDCl_3$): δ = 142.7, 141.1, 129.5, 126.2, 33.0, 31.6, 23.0, 22.7, 21.6, 16.3, 14.2, -1.5.

^{29}Si NMR (100 MHz, $CDCl_3$): δ = 11.1.

HRMS ESI [M+Na^{+}] Calculated for $C_{15}H_{27}O_2NNaSSi$= 336.1424. Found = 336.1421.

Product 14

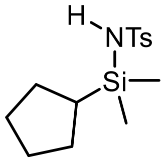

^{1}H NMR (400 MHz, $CDCl_3$): δ = 7.75 (d, *J* = 8.3 Hz, 2H), 7.27 (d, *J* = 8.2 Hz, 2H), 4.32 (s, 1H), 2.41 (s, 3H), 1.72 (m, 2H), 1.53 (m, 4H), 1.25 (m, 2H), 1.12 (m, 1H), 0.21 (s, 6H).

^{13}C{^{1}H} NMR (100 MHz, $CDCl_3$): δ = 142.8, 141.0, 129.6, 126.3, 27.5, 27.2, 25.5, 21.6, -2.9.

^{29}Si NMR (80 MHz, $CDCl_3$): δ = 11.4.

HRMS ESI [M+Na$^+$] Calculated for $C_{14}H_{23}O_2NNaSSi$ = 320.1111. Found = 320.1108.

Product 15

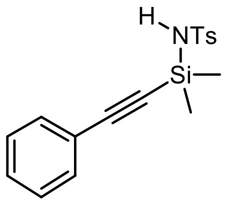

^{1}H NMR (400 MHz, $CDCl_3$): δ = 7.84 (m, 2H), 7.41 (m, 2H), 7.33 (m, 3H), 4.58 (m, 1H), 2.39 (s, 3H), 0.53 (m, 6H).

^{13}C{^{1}H} NMR (100 MHz, $CDCl_3$): δ = 143.1, 140.3, 132.2, 129.6, 129.4, 128.4, 126.6, 121.1, 106.9, 89.6, 21.6, 0.9.

^{29}Si NMR (100 MHz, $CDCl_3$): δ = -15.8.

HRMS ESI [M+Na$^+$] Calculated for $C_{17}H_{19}O_2NNaSSi$= 352.0798. Found = 352.0798.

Product 16

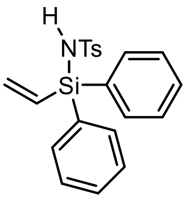

^{1}H NMR (400 MHz, $CDCl_3$): δ = 7.82 (d, *J* = 8.3 Hz, 2H), 7.63 (m, 2H), 7.40 (m, 8H), 7.32 (d, *J* = 8.0 Hz, 2H), 6.49 (m, 1H), 6.26 (m, 1H), 5.93 (m, 1H), 4.76 (s, 1H), 2.43 (s, 3H).

^{13}C{^{1}H} NMR (100 MHz, $CDCl_3$): δ = 143.8, 139.2, 136.6, 135.6, 135.3, 134.7, 134.6, 130.2, 129.9, 128.1, 126.6, 31.1, 29.8, 21.7.

^{29}Si NMR (100 MHz, $CDCl_3$): δ = -15.6.

HRMS ESI [M+Na$^+$] Calculated for $C_{21}H_{21}O_2NNaSSi$= 402.0954. Found = 402.0947.

Product 17

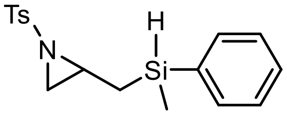

^{1}H NMR (400 MHz, $CDCl_3$): δ = 7.78 (m, 2H), 7.49 (m, 2H), 7.38 (m, 3H), 7.31 (m, 2H), 4.35 (m, 1H), 2.88 (m, 1H), 2.58 (m, 1H), 2.43 (s, 3H), 1.93 (m, 1H), 1.31 (m, 1H), 1.00 (m, 1H), 0.37 (m, 3H).

^{13}C{^{1}H} NMR (100 MHz, $CDCl_3$): δ = 144.5, 135.4, 134.6, 134.4, 129.9, 129.8, 128.2, 128.0, 38.3, 38.1, 35.4, 35.3, 21.8, 17.2, 17.0, -5.5, -5.6.

^{29}Si NMR (100 MHz, $CDCl_3$): δ = -16.3 and -16.8.

HRMS ESI [M+Na$^+$] Calculated for $C_{17}H_{21}O_2NNaSSi$= 354.0954. Found = 354.0954.

Product 18

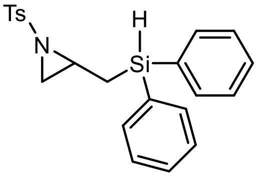

^{1}H NMR (400 MHz, $CDCl_3$): δ = 7.73 (m, 2H), 7.50 (m, 4H), 7.38 (m, 6H), 7.28 (m, 2H), 4.84 (dd, *J* = 4.1, 3.2 Hz, 1H), 2.91 (ddt, *J* = 8.6, 7.0, 4.9 Hz, 1H), 2.56 (d, *J* = 7.0 Hz, 1H), 2.43 (s, 3H), 1.93 (d, *J* = 4.6 Hz, 1H), 1.67 (ddd, *J* = 14.8, 5.1, 3.2 Hz, 1H), 1.25 (ddt, *J* = 16.9, 8.3, 4.1 Hz, 1H).

^{13}C{^{1}H} NMR (100 MHz, $CDCl_3$): δ = 144.5, 135.4, 135.2, 132.8, 132.7, 130.0, 129.8, 128.3,128.0, 38.0, 35.3, 21.8, 16.1.

^{29}Si NMR (100 MHz, $CDCl_3$): δ = -18.0.

HRMS ESI [M+Na$^+$] Calculated for $C_{22}H_{23}O_2NNaSSi$= 416.1111. Found = 416.1108.

Product 19

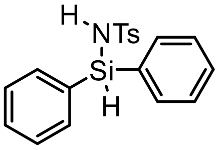

^{1}H NMR (400 MHz, $CDCl_3$): δ = 7.65 (m, 4H), 7.56 (m, 4H), 7.41 (m, 2H), 7.36 (m, 4H), 5.60 (s, 1H), 5.53 (s, 1H), 2.43 (s, 3H).

^{13}C{^{1}H} NMR (100 MHz, $CDCl_3$): δ = 143.8, 139.2, 135.1, 130.6, 130.4, 128.2, 128.1, 21.4.

^{29}Si NMR (100 MHz, $CDCl_3$): δ = -20.99.

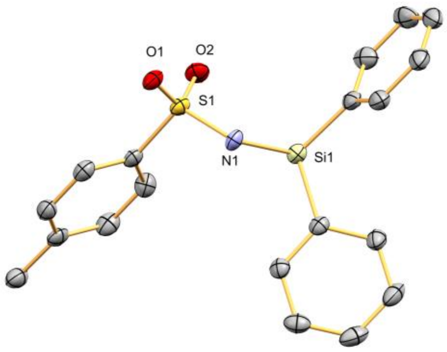

ORTEP for **19.**

Product 20

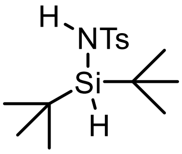

^{1}H NMR (400 MHz, $CDCl_3$): δ = 7.78 (d, *J* = 6.6 Hz, 2H), 7.26 (d, *J* = 6.6 Hz, 2H), 4.57 (d, *J* = 6.0 Hz, 1H), 4.09 (d, *J* = 6.1 Hz, 1H), 2.40 (s, 3H), 0.95 (s, 18H).

^{13}C{^{1}H} NMR (100 MHz, $CDCl_3$): δ = 142.9, 140.4, 129.5, 126.6, 27.7, 21.6, 19.5.

^{29}Si NMR (100 MHz, $CDCl_3$): δ = 6.92.

HRMS ESI [M+Na$^+$] Calculated for $C_{15}H_{27}O_2NNaSSi$= 336.1424. Found = 336.1423.

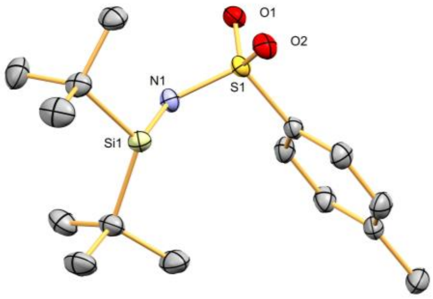

ORTEP for **20.**

Product 21

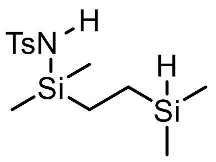

^{1}H NMR (400 MHz, CDCl$_3$): δ = 7.75 (d, *J* = 8.3 Hz, 2H), 7.28 (d, *J* = 8.3 Hz, 2H), 4.29 (s, 1H), 3.79 (dh, *J* = 3.5 Hz, 1H), 2.41 (s, 3H), 0.66 (m, 2H), 0.43 (m, 2H), 0.23 (s, 6H), 0.04 (d, *J* = 3.6 Hz, 6H).

^{13}C{^{1}H} NMR (100 MHz, CDCl$_3$): δ = 142.9, 141.0, 129.6, 126.3, 21.6, 9.3, 5.7, 1.2, -4.8.

^{29}Si NMR (100 MHz, CDCl$_3$): δ = 11.6, -9.8.

HRMS ESI [M+Na$^+$] Calculated for $C_{13}H_{25}O_2NNaSSi_2$ = 338.1037. Found = 338.1038.

Product 22

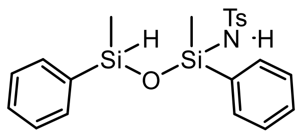

^{1}H NMR (400 MHz, CDCl$_3$): δ = 7.82 (d, *J* = 8.4 Hz, 2H), 7.60 (m, 2H), 7.38 (m, 8H), 7.32 (d, *J* = 8.0 Hz, 2H), 5.19 (m, 1H), 4.75 (m, 1H), 2.44 (s, 3H), 0.48 (d, *J* = 2.8 Hz, 3H), 0.38 (s, 3H).

^{13}C{^{1}H} NMR (100 MHz, CDCl$_3$): δ = 143.8, 139.2, 137.2, 136.7, 133.6, 133.5, 133.4, 130.2, 130.1, 129.9, 128.1, 128.0, 126.6, 21.7, -0.3, -0.4, -1.0, -1.1.

^{29}Si NMR (100 MHz, CDCl$_3$): δ = -12.8, -12.9, -13.4, -13.5.

Product 23

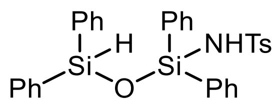

^{1}H NMR (400 MHz, CDCl$_3$): δ = 7.59 (m, 4H), 7.55 (m, 4H), 7.43 (m, 4H), 7.36-7.28 (m, 10H), 7.02 (m, 2H), 5.69 (s, 1H), 4.96 (br, 1H), 2.34 (s, 3H).

^{13}C{^{1}H} NMR (100 MHz, CDCl$_3$): δ = 142.8, 140.0, 134.9, 134.6, 132.0, 130.9, 130.5, 129.3, 128.2, 128.1, 126.6, 21.6.

^{29}Si NMR (100 MHz, CDCl$_3$): δ = -19.1, -20.6.

HRMS ESI [M+Na$^+$] Calculated for $C_{31}H_{29}O_3NNaSSi_2$= 574.1299. Found = 574.1294.

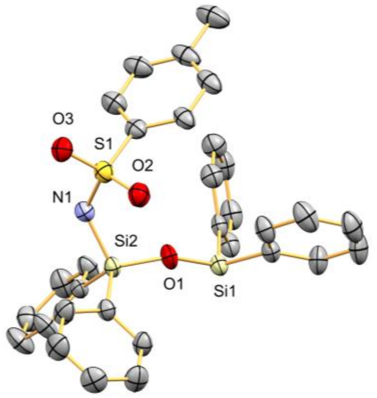

ORTEP for **24.**

Product 24

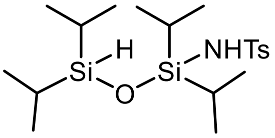

^{1}H NMR (400 MHz, $CDCl_3$): δ = 7.78 (d, *J* = 8.0 Hz, 2H), 7.28 (d, *J* = 8.0 Hz, 2H), 4.60 (s, 1H), 3.18 (s, 1H), 2.42 (s, 3H), 1.14 (m, 2H), 1.06-0.96 (m, 26H).

^{13}C{^{1}H} NMR (100 MHz, $CDCl_3$): δ = 143.2, 140.3, 129.6, 126.4, 21.6, 17.4, 17.3, 17.1, 13.5, 13.3.

^{29}Si NMR (100 MHz, $CDCl_3$): δ = -11.8, -12.9.

HRMS ESI [M+Na$^+$] Calculated for $C_{19}H_{37}O_3NNaSSi_2$= 438.1928. Found = 438.2444.

5. NMR spectra of products

^{1}H NMR spectrum for **1** (400 MHz, $CDCl_3$)*

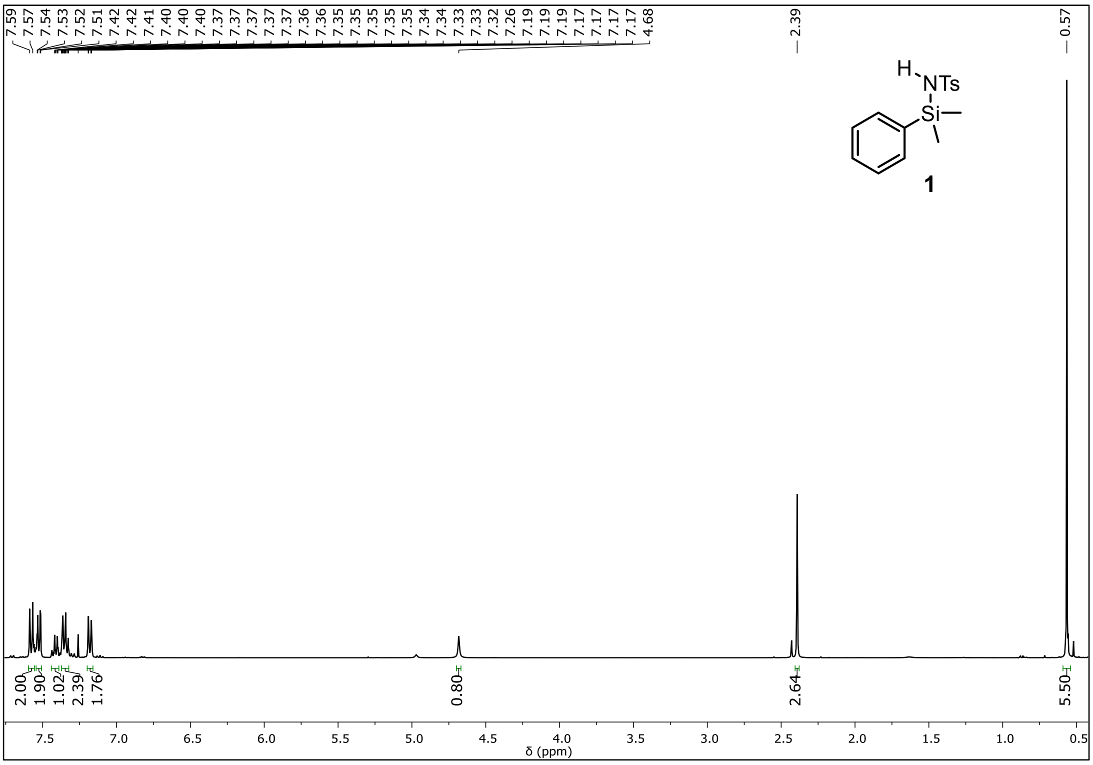

*5% $TsNH_2$ is observed.

$^{13}C\{^1H\}$ NMR spectrum for **1** (100 MHz, $CDCl_3$)

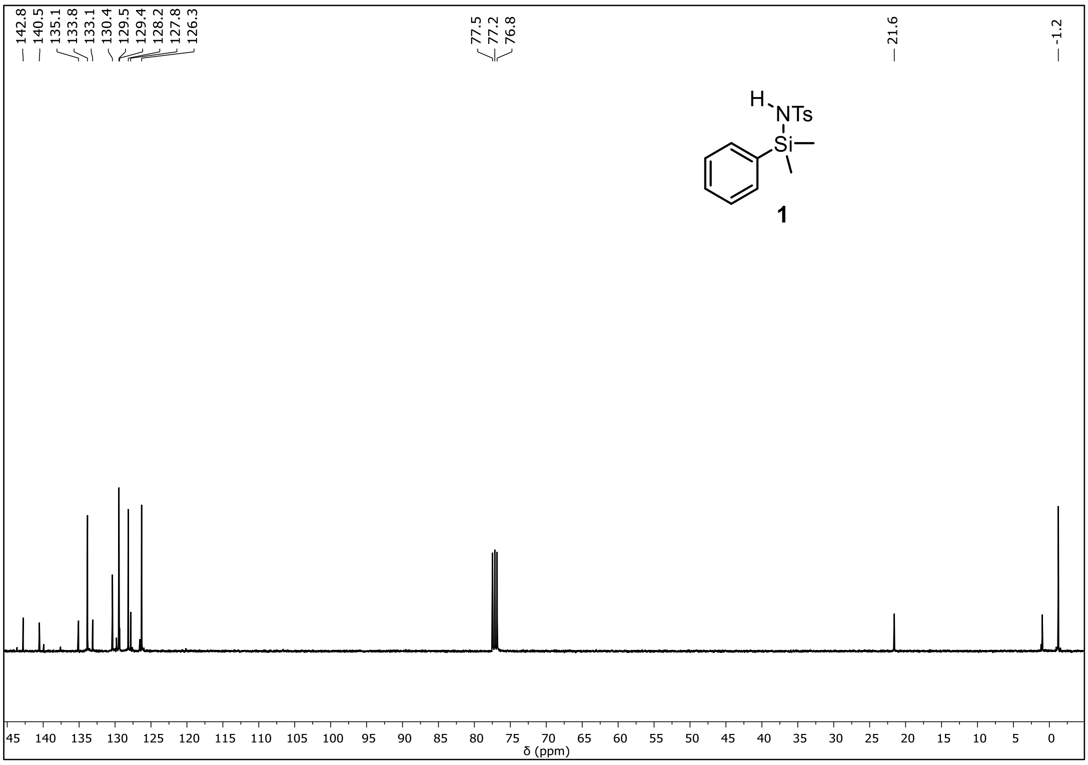

^{15}N NMR spectrum for **1** (81 MHz, $CDCl_3$)

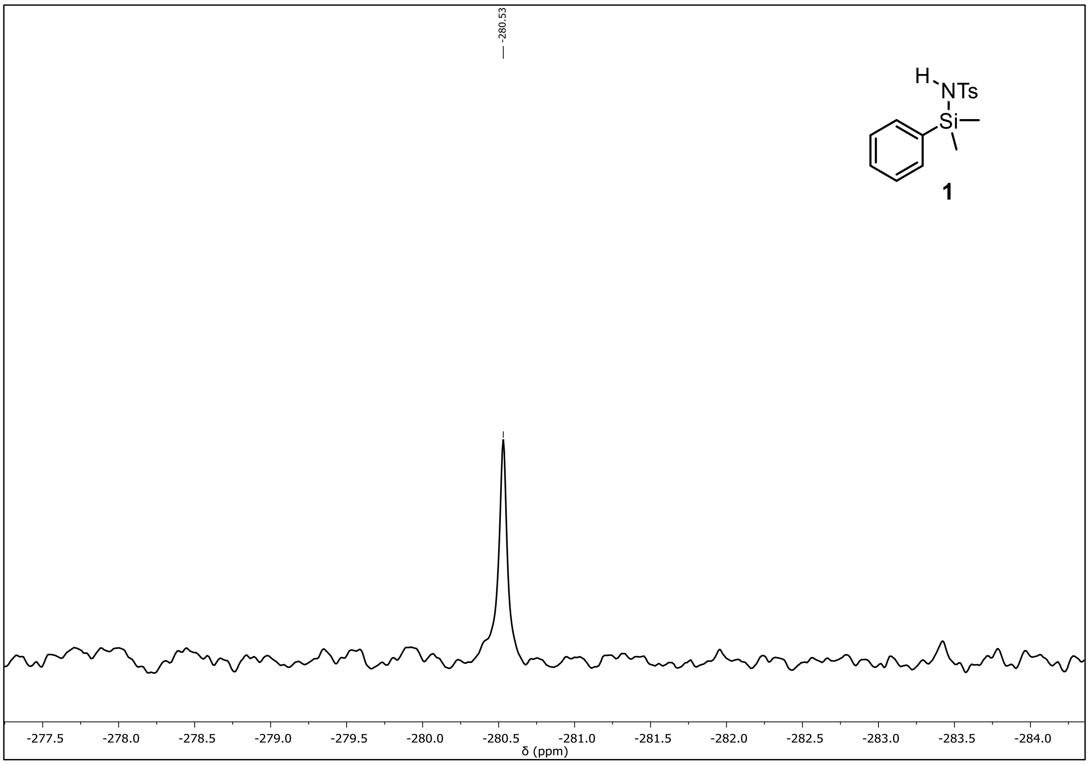

^{1}H NMR spectrum for **2** (400 MHz, $CDCl_3$)

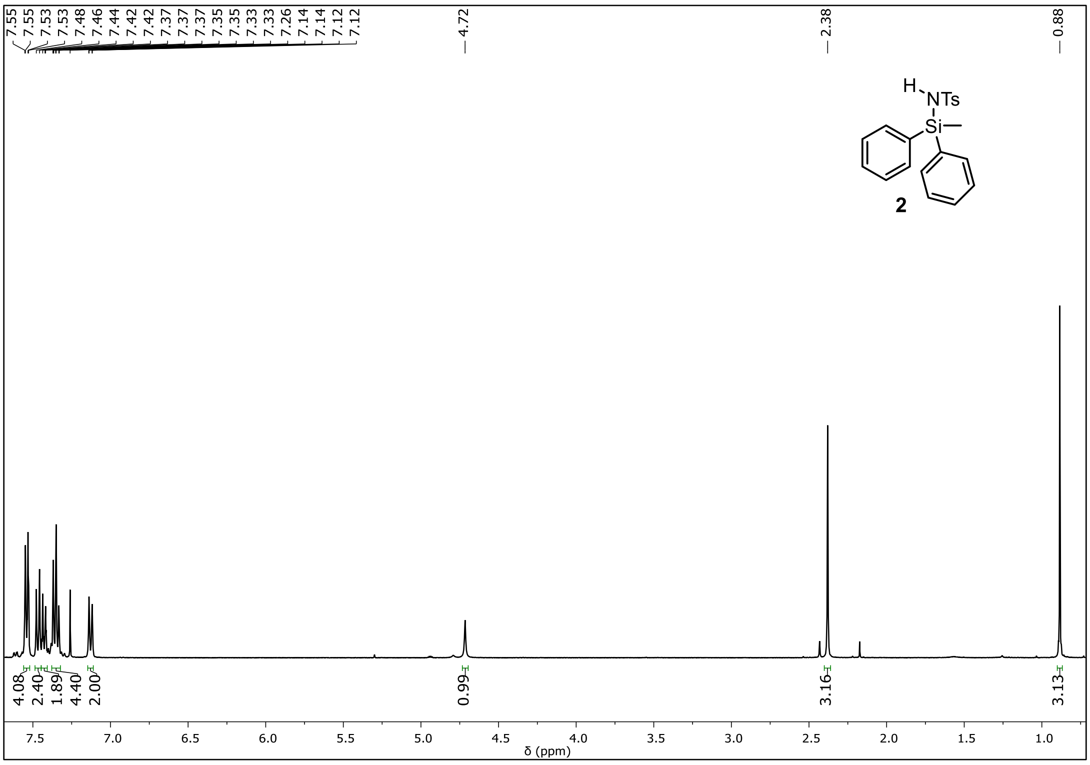

^{13}C{^{1}H} NMR spectrum for **2** (100 MHz, $CDCl_3$)

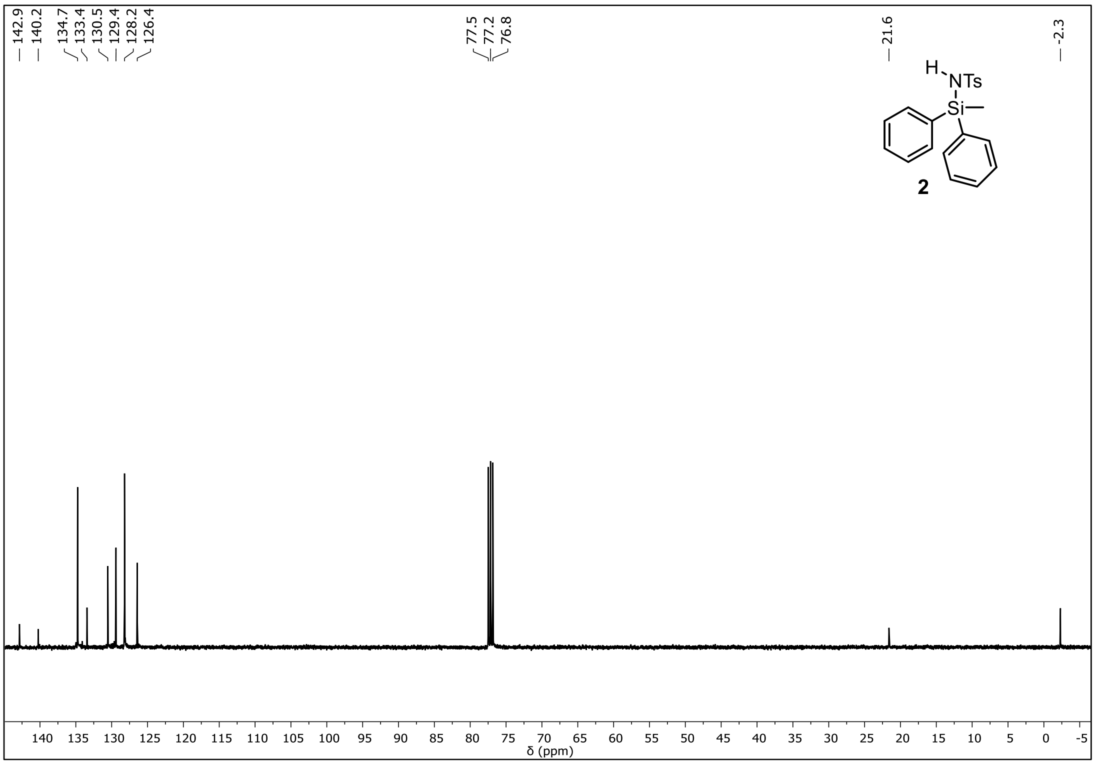

^{1}H NMR spectrum for **3** (400 MHz, $CDCl_3$)

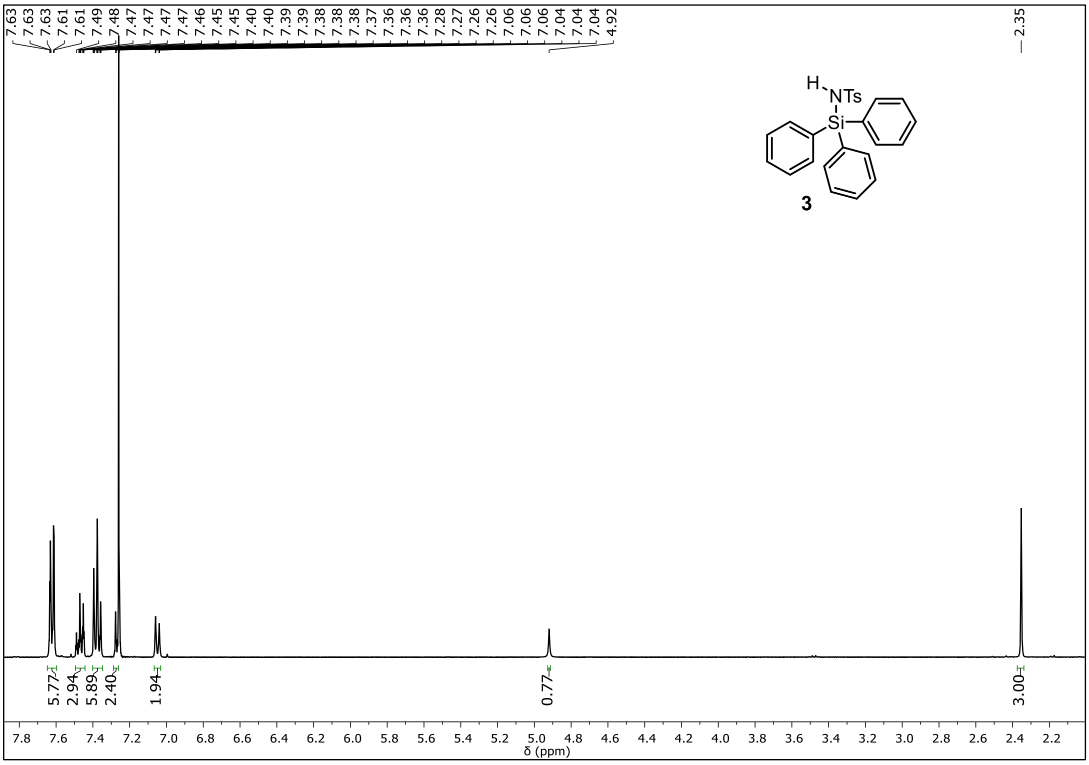

^{13}C{^{1}H} NMR spectrum for **3** (100 MHz, $CDCl_3$)

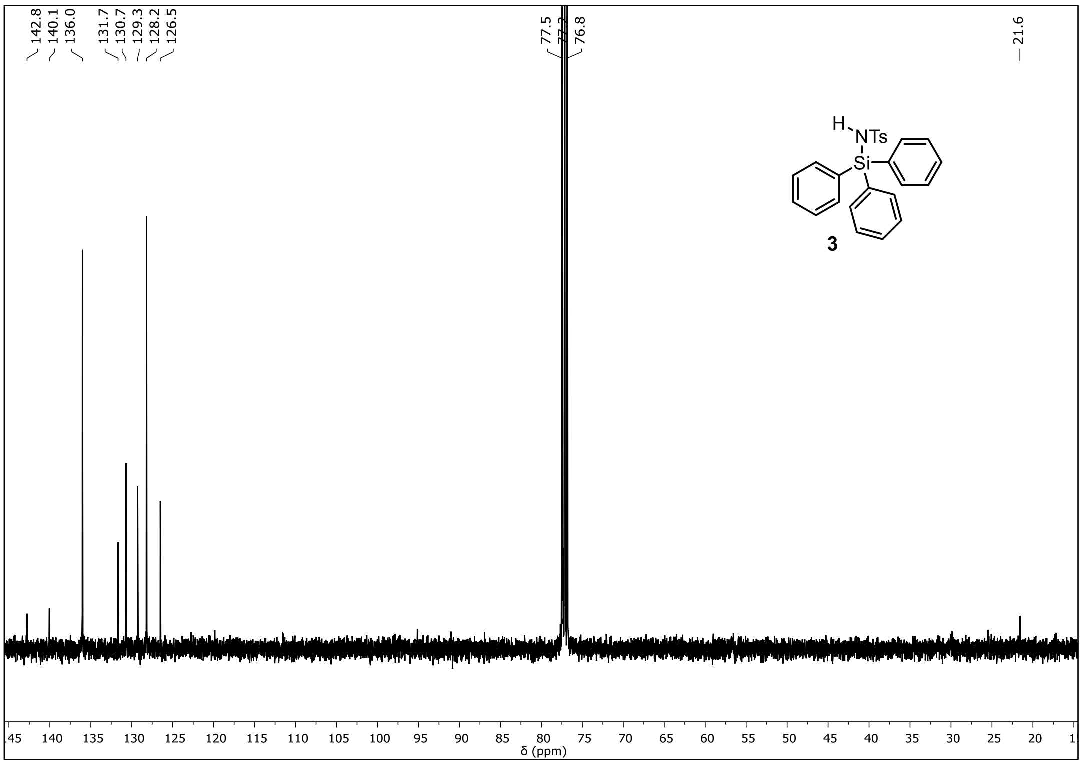

^{1}H NMR spectrum for **4** (400 MHz, $CDCl_3$)

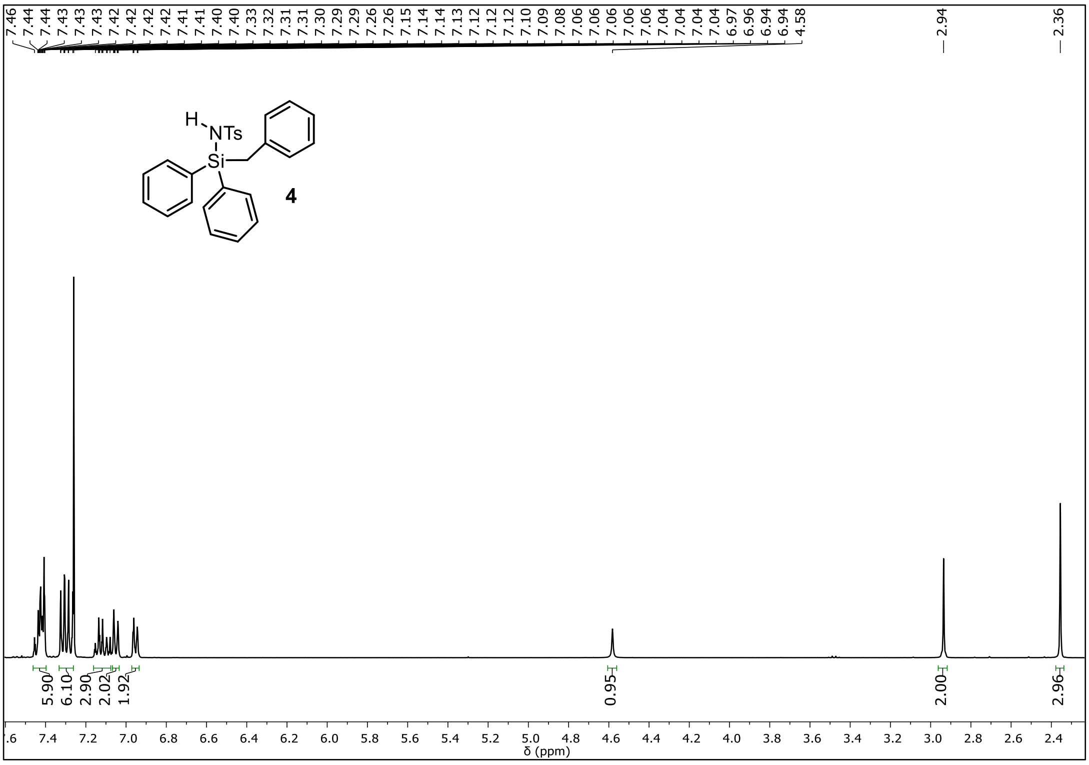

$^{13}C\{^{1}H\}$ NMR spectrum for **4** (100 MHz, $CDCl_3$)

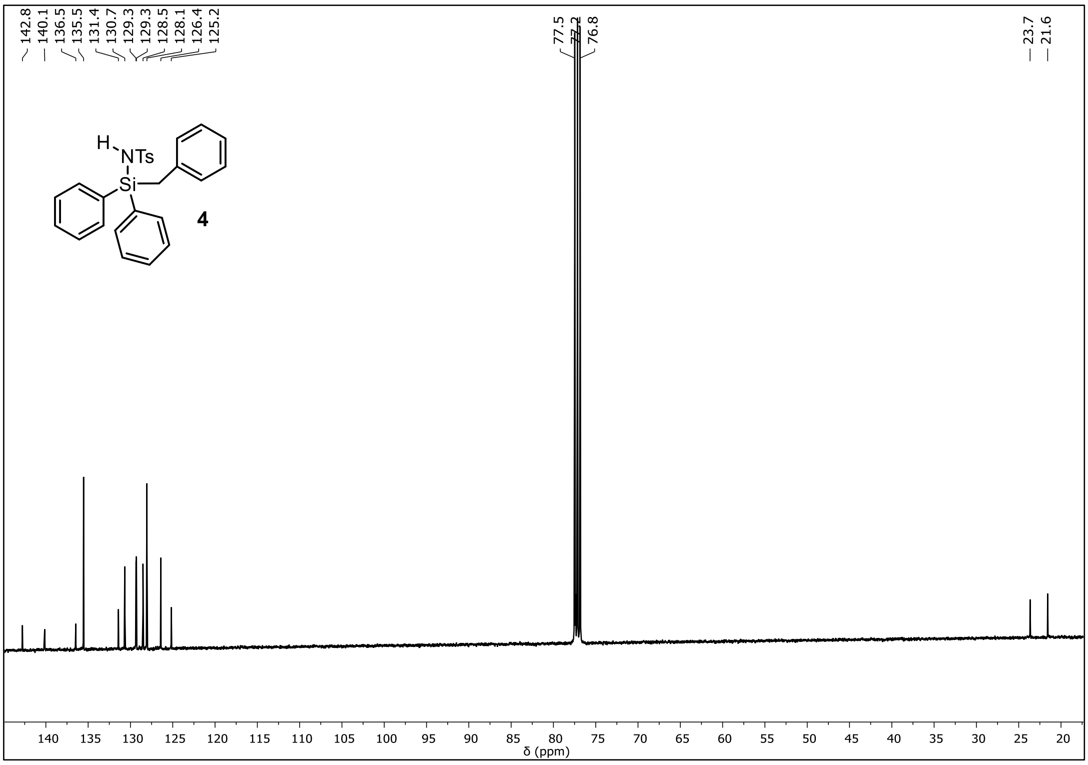

^{15}N NMR spectrum for **4** (81 MHz, $CDCl_3$)

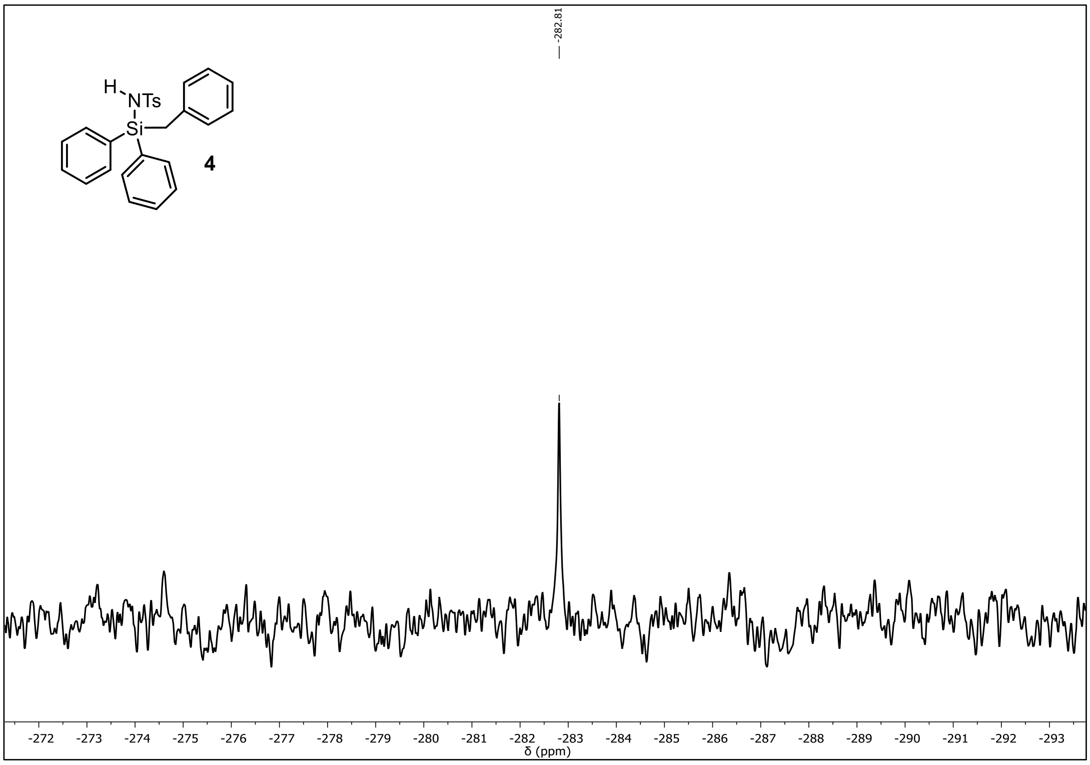

^{1}H NMR spectrum for **5** (400 MHz, $CDCl_3$)

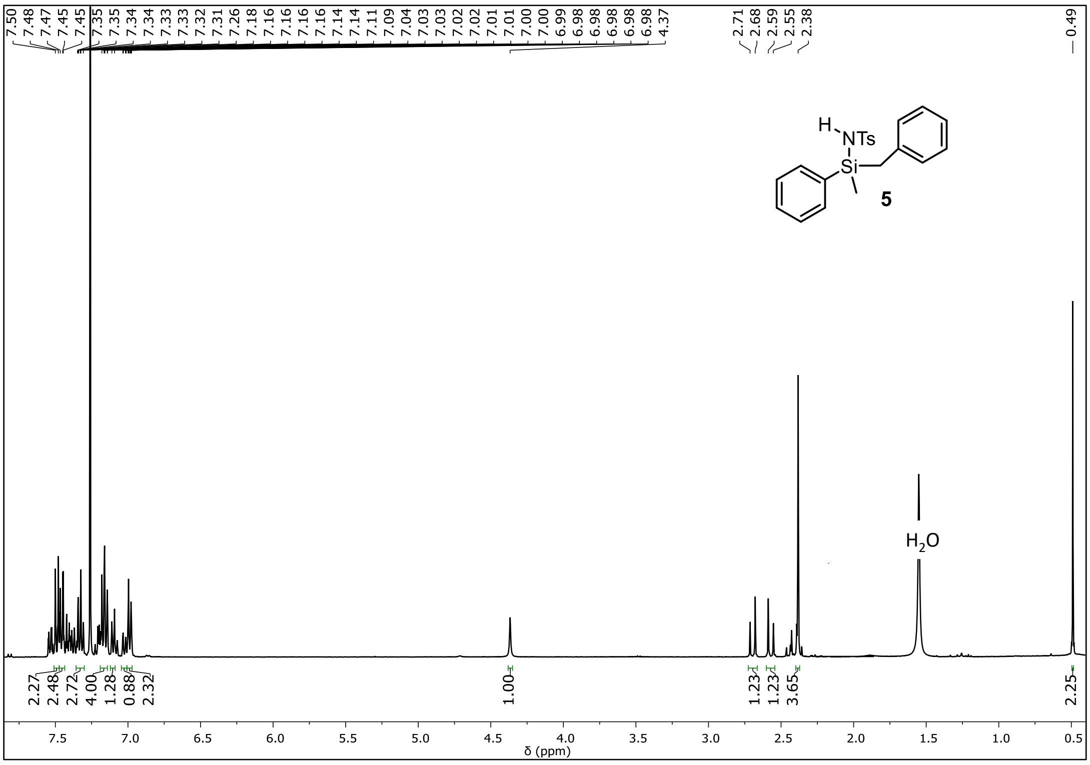

$^{13}C\{^{1}H\}$ NMR spectrum for **5** (100 MHz, $CDCl_3$)

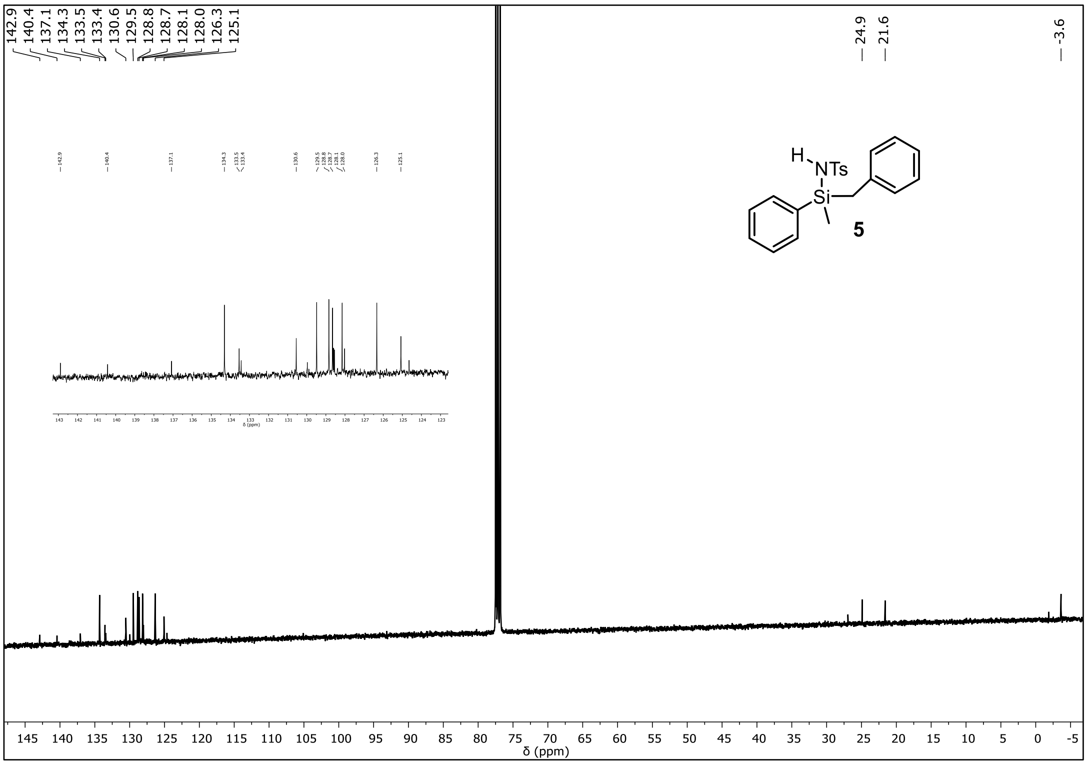

^{1}H-^{15}N HSQC NMR spectrum for **5** ($CDCl_3$)

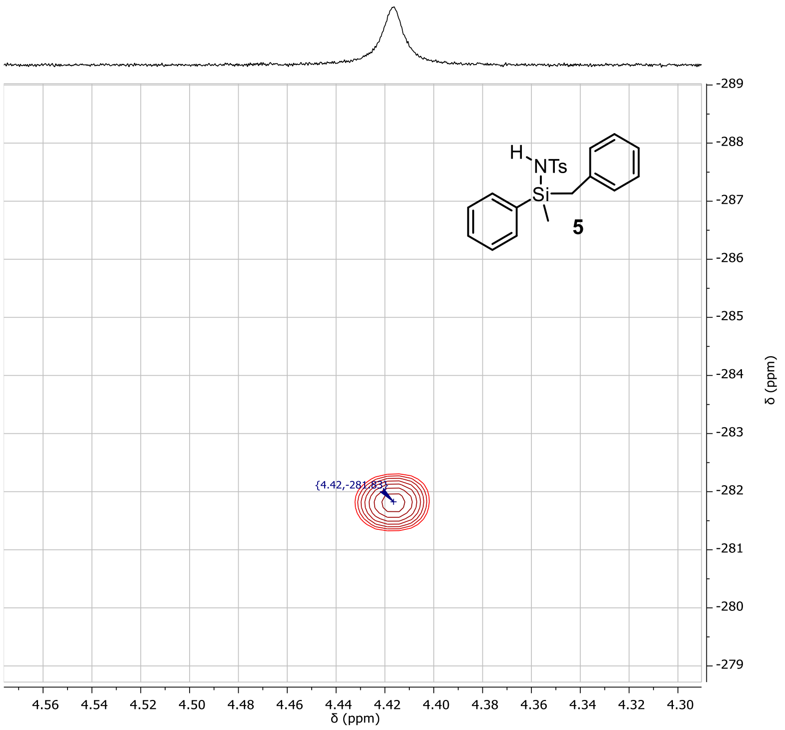

^{1}H NMR spectrum for **6** (400 MHz, $CDCl_3$)

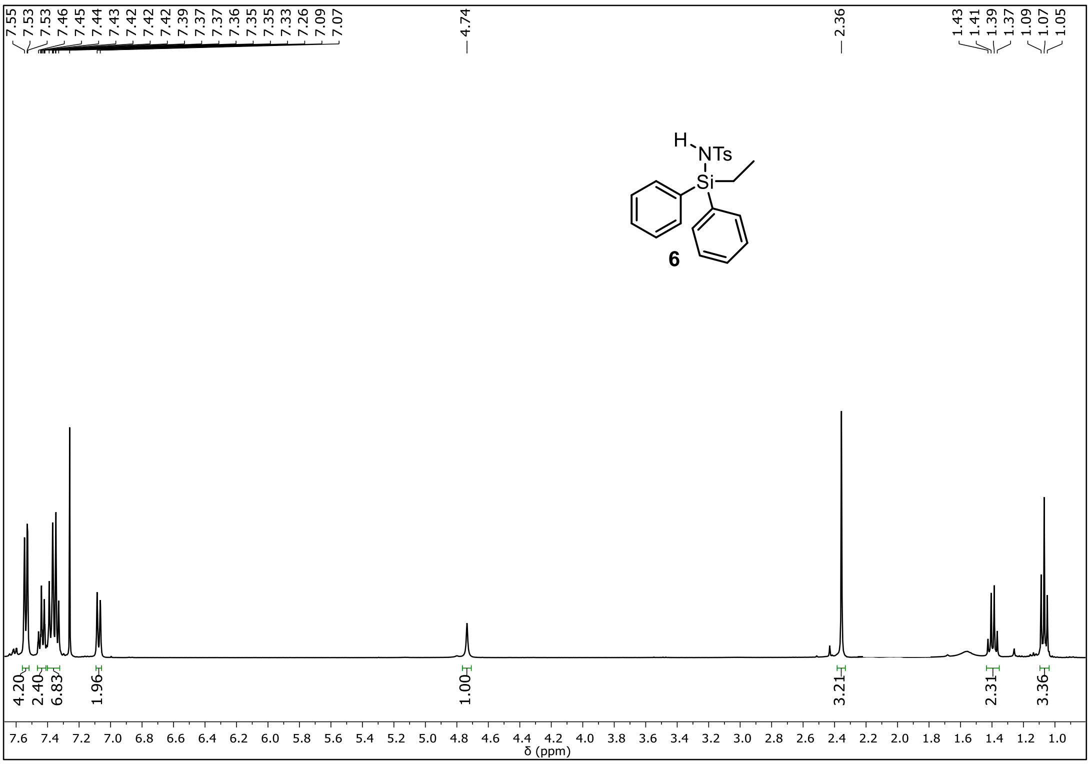

^{13}C{^{1}H} NMR spectrum for **6** (100 MHz, $CDCl_3$)

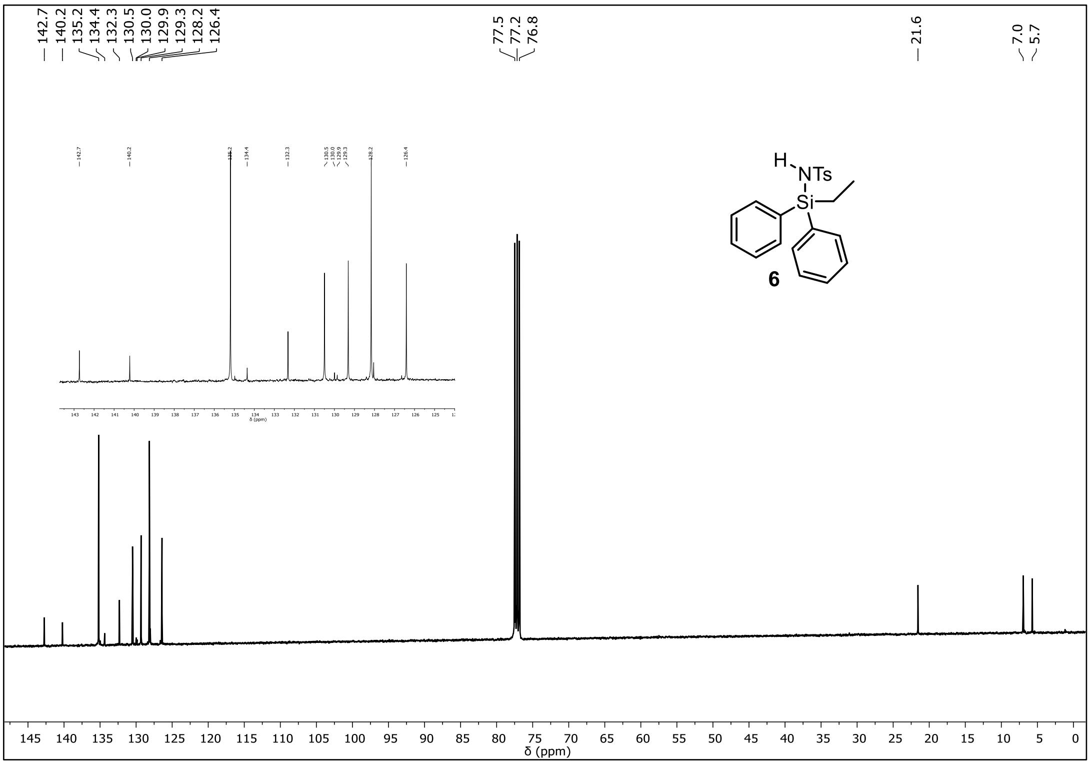

^{1}H-^{15}N HSQC NMR spectrum for **6** (CDCl$_3$)

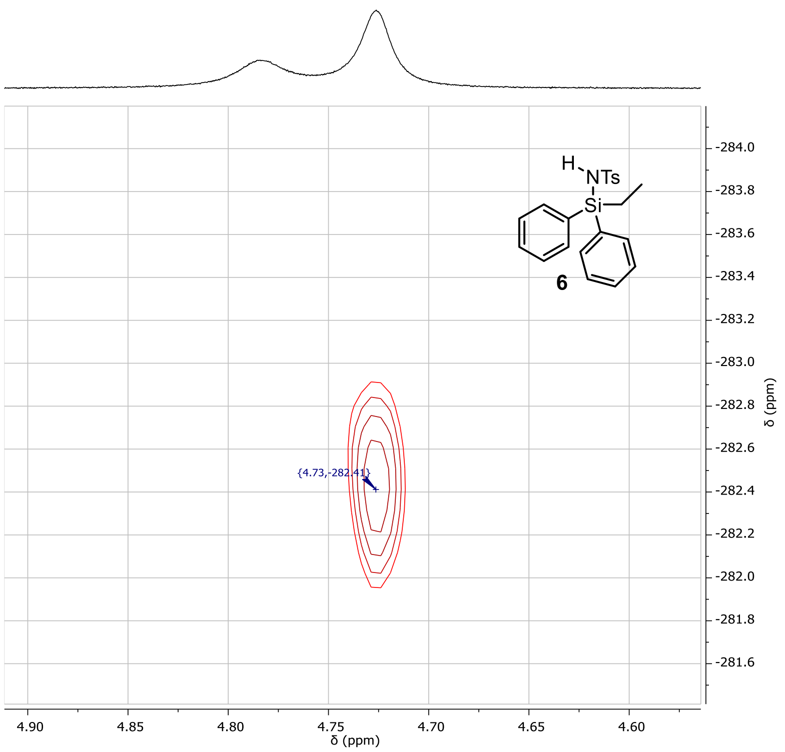

^{1}H NMR spectrum for **7** (400 MHz, $CDCl_3$)

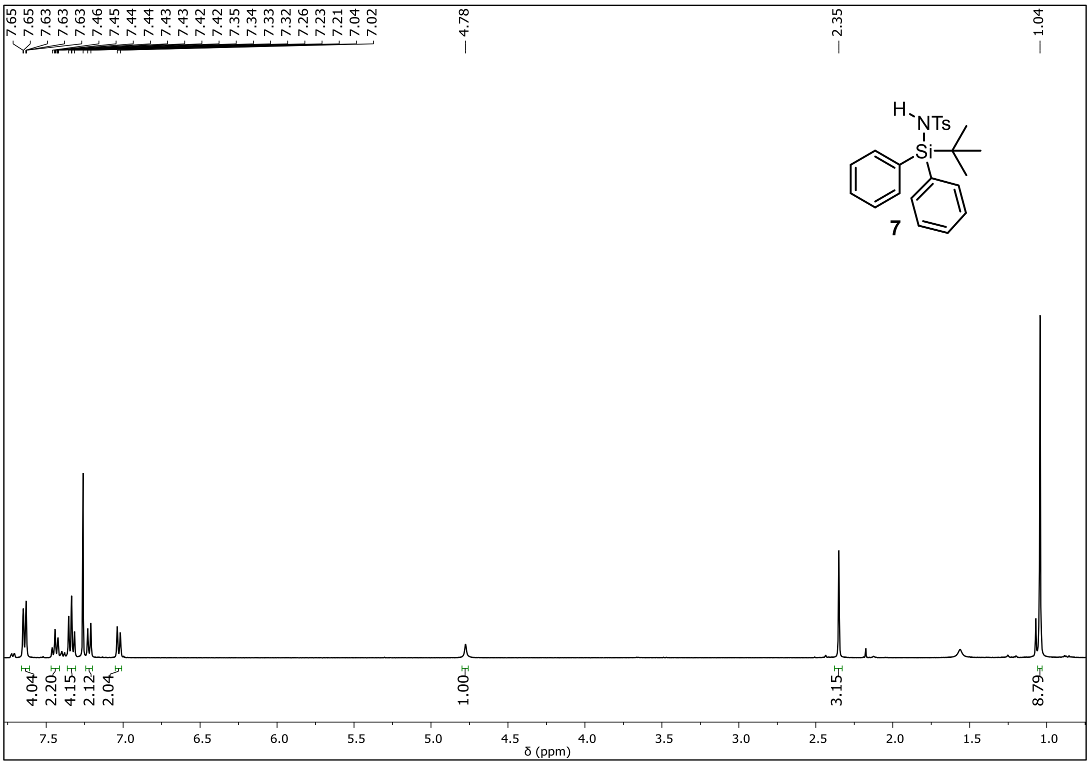

$^{13}C\{^{1}H\}$ NMR spectrum for **7** (100 MHz, $CDCl_3$)

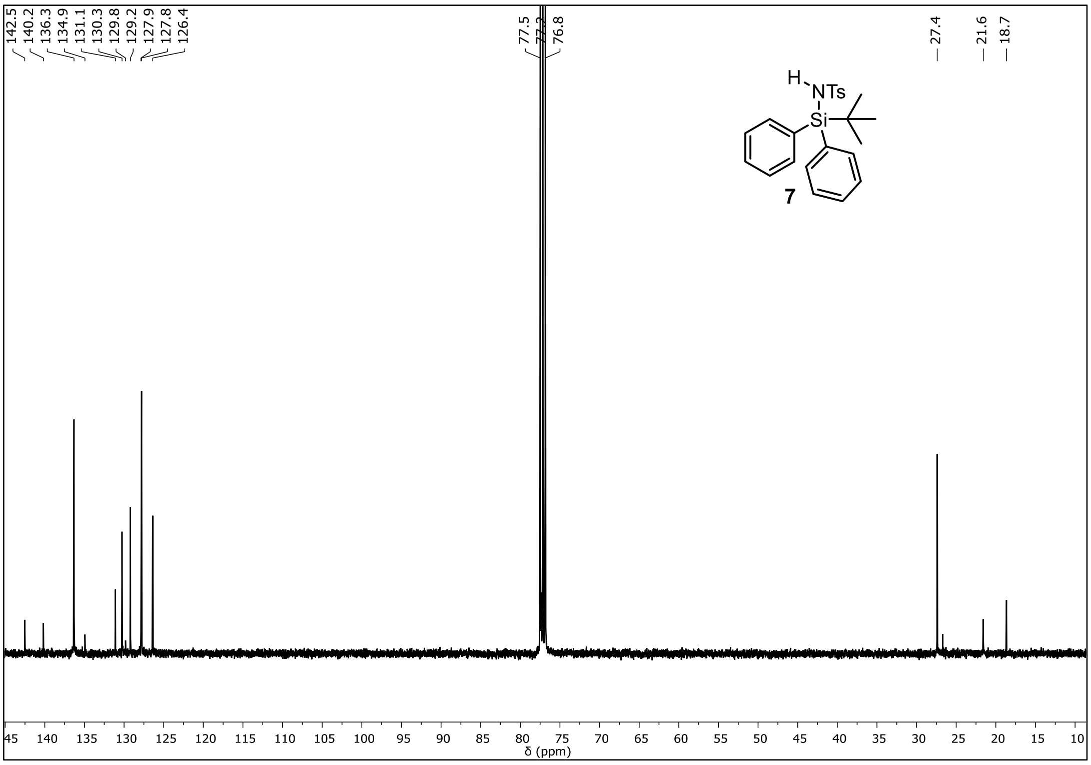

^{1}H NMR spectrum for **8** (400 MHz, $CDCl_3$)

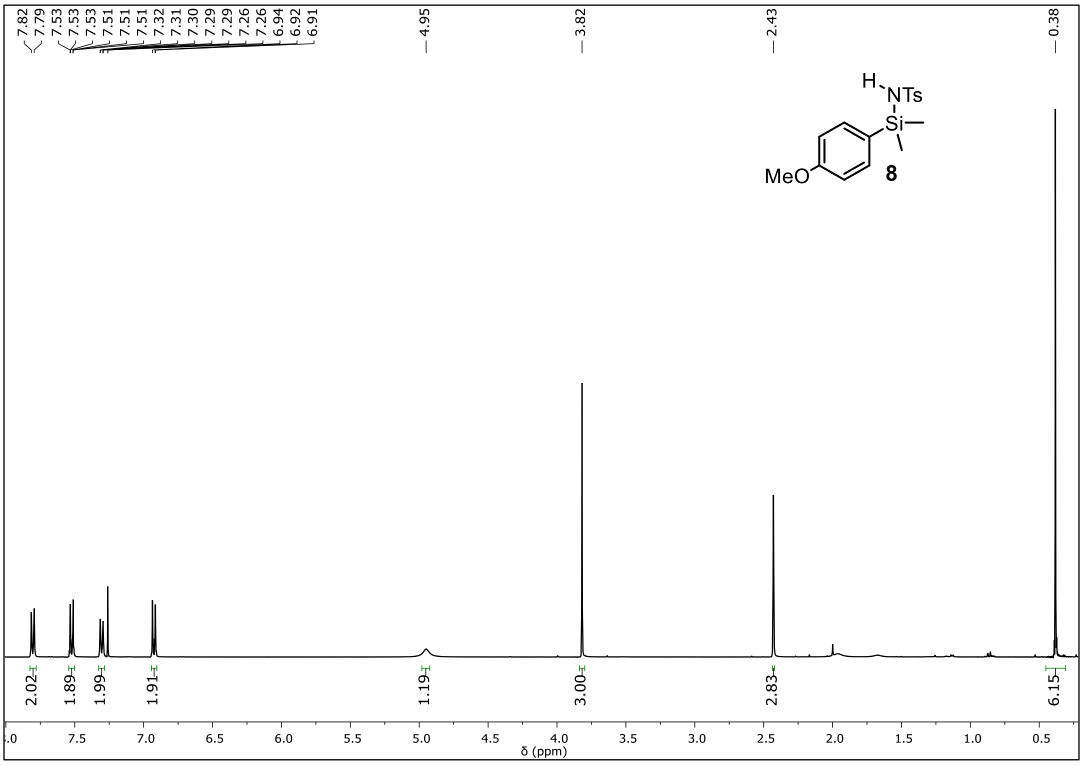

^{13}C{^{1}H} NMR spectrum for **8** (100 MHz, $CDCl_3$)

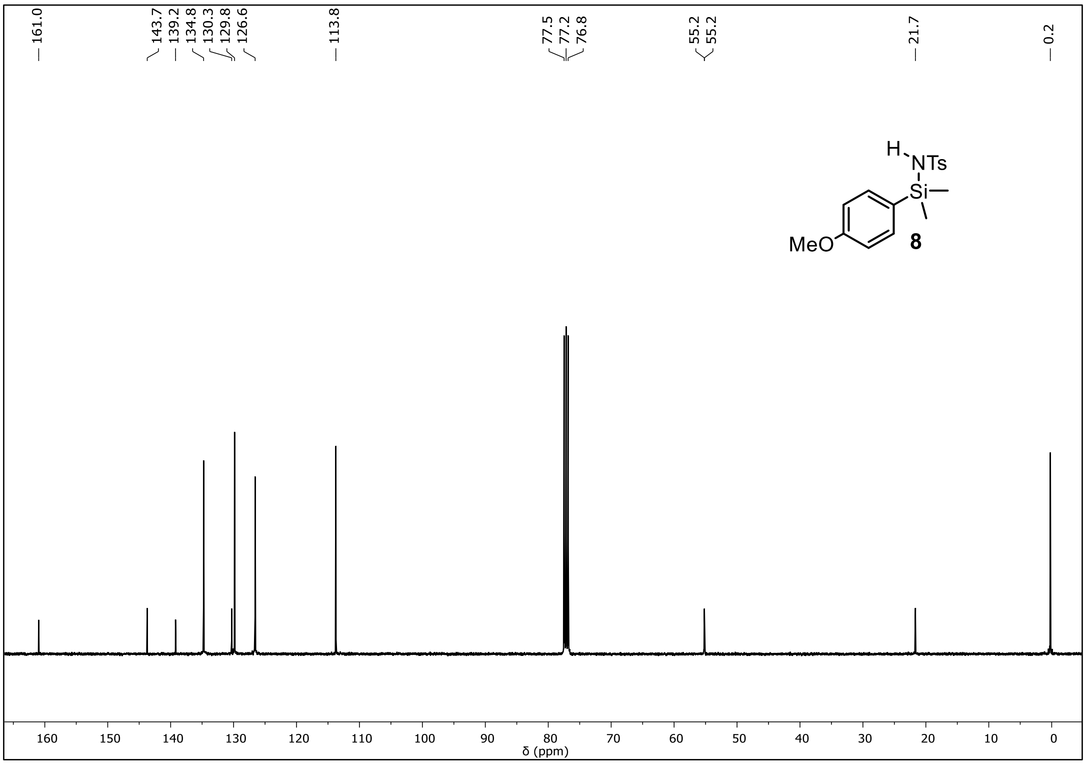

^{1}H-^{15}N HSQC NMR spectrum for **8** ($CDCl_3$)

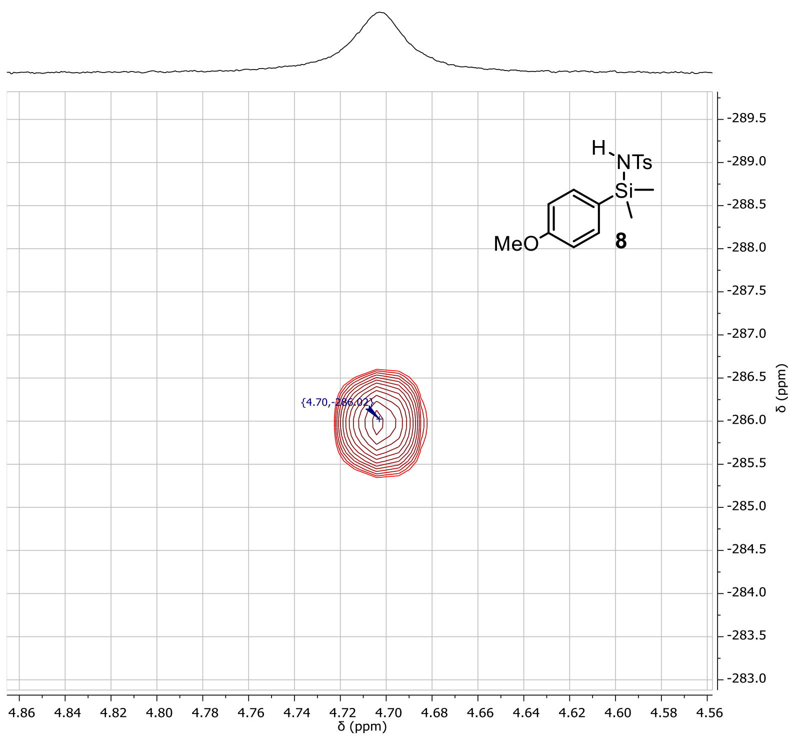

^{1}H NMR spectrum for **9** (400 MHz, $CDCl_3$)

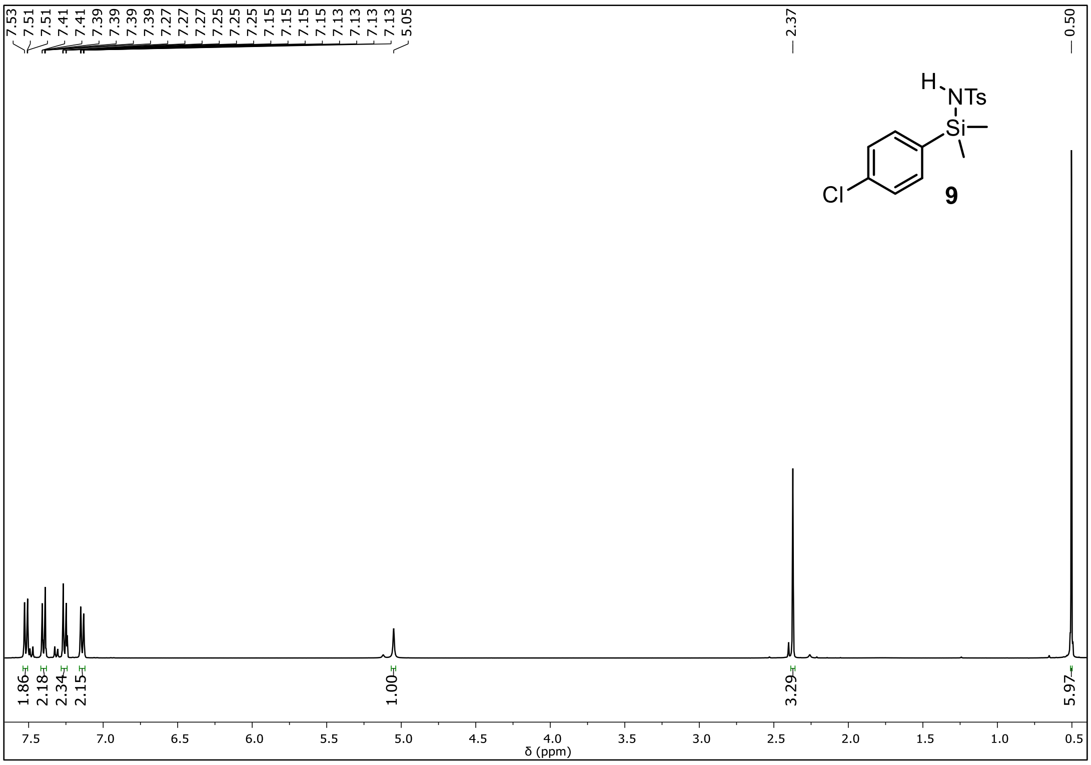

$^{13}C\{^{1}H\}$ NMR spectrum for **9** (100 MHz, $CDCl_3$)

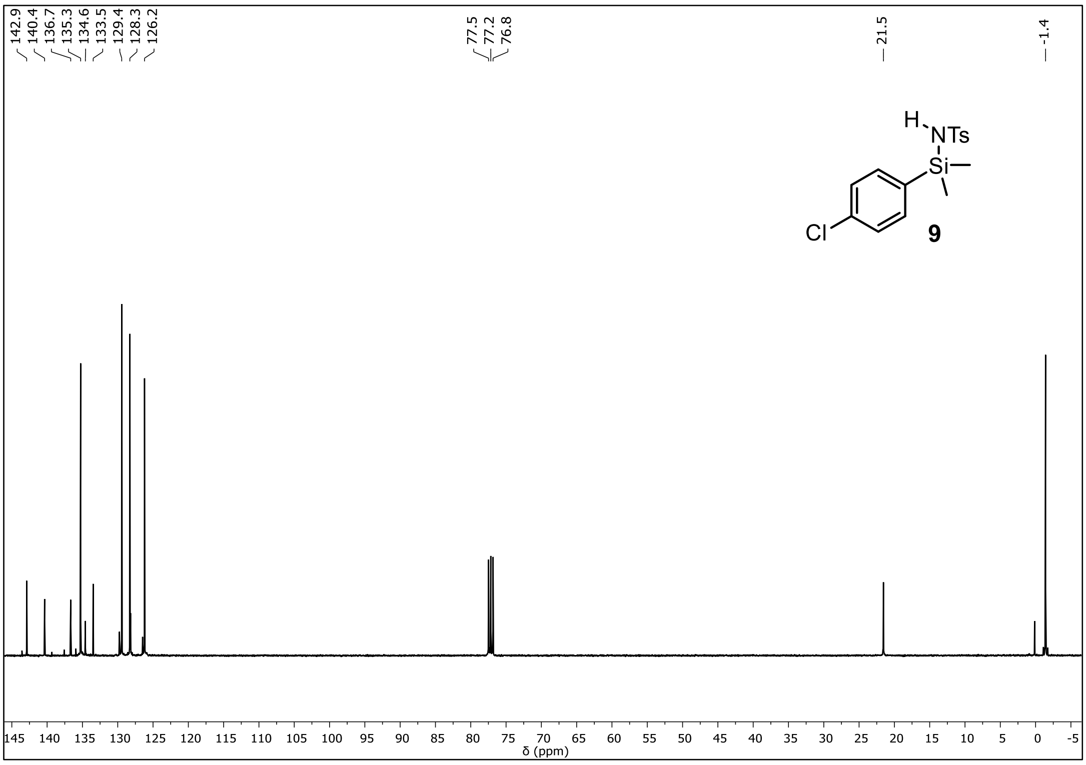

^{1}H NMR spectrum for **10** (400 MHz, $CDCl_3$)

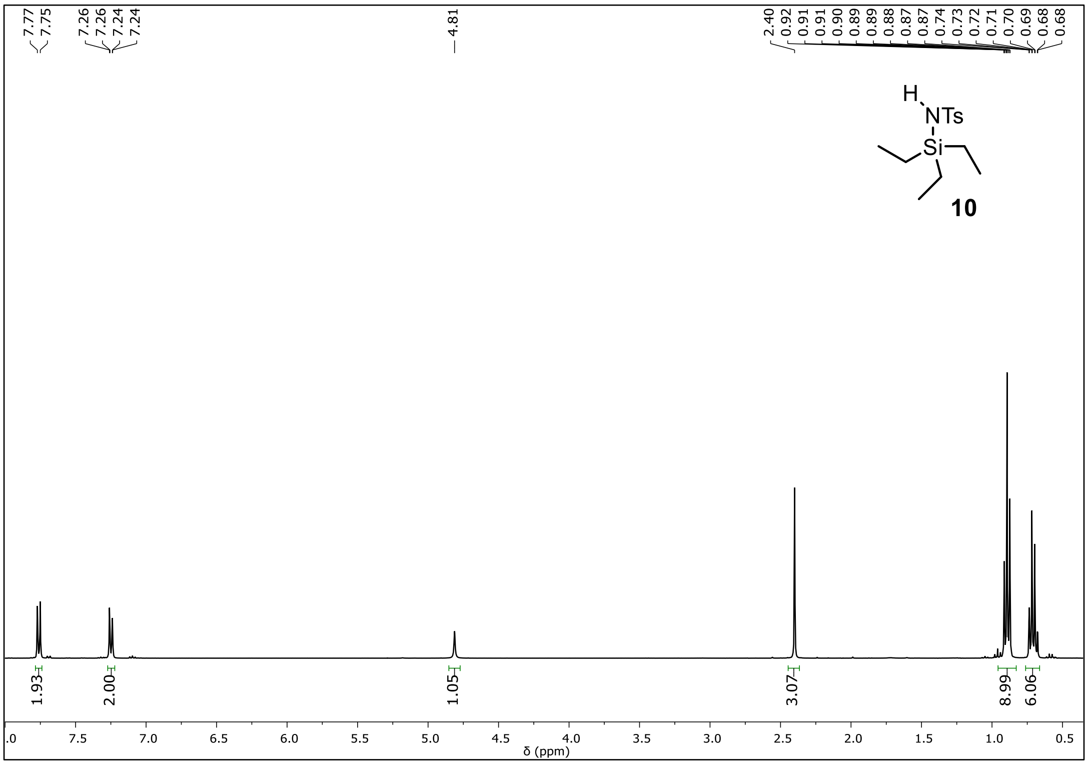

^{13}C{^{1}H} NMR spectrum for **10** (100 MHz, $CDCl_3$)

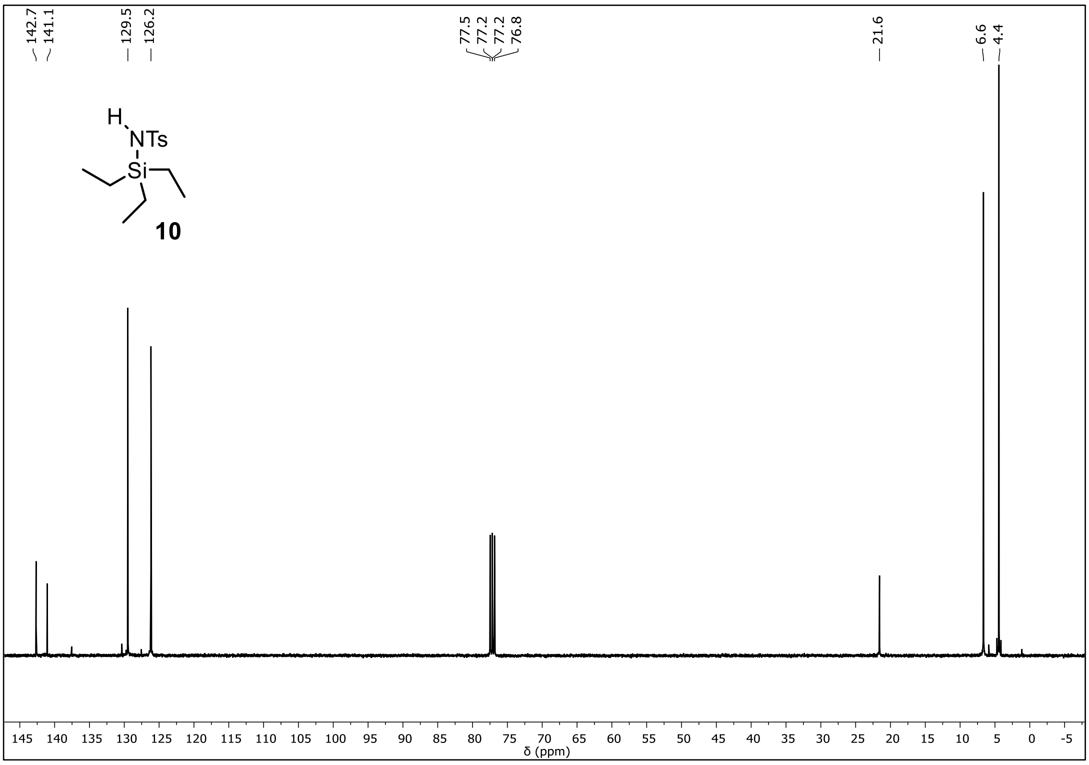

^{15}N NMR spectrum for **10** (81 MHz, $CDCl_3$)

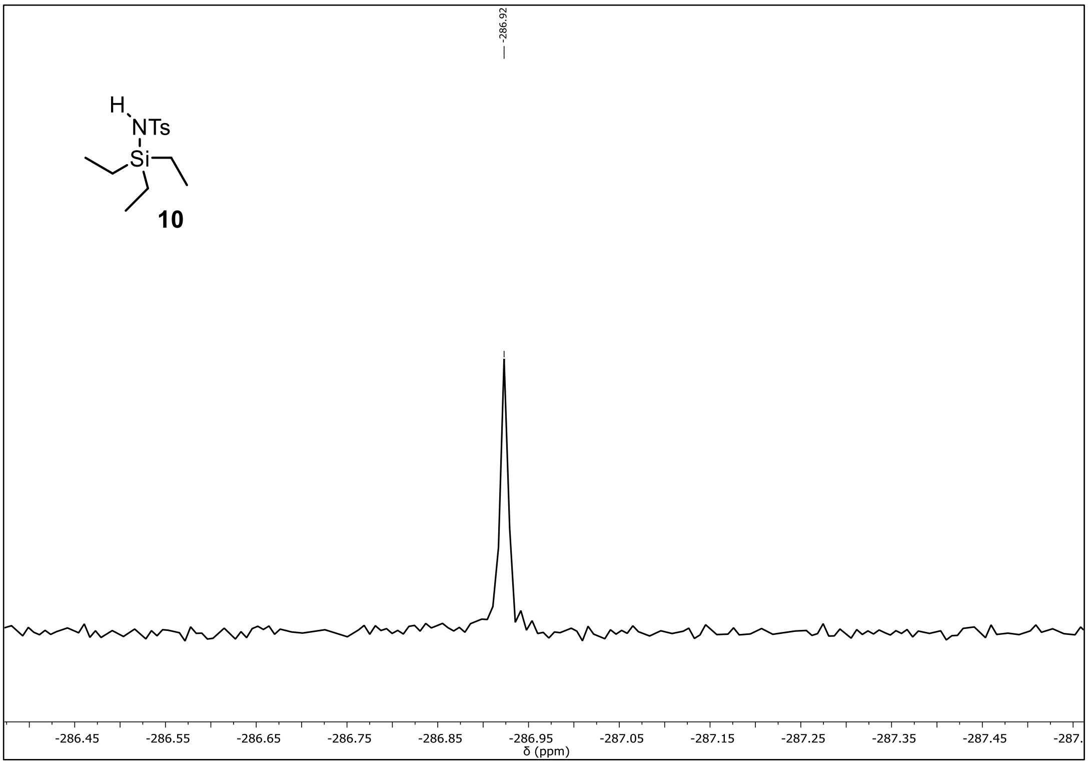

^{1}H NMR spectrum for **11** (400 MHz, $CDCl_3$)

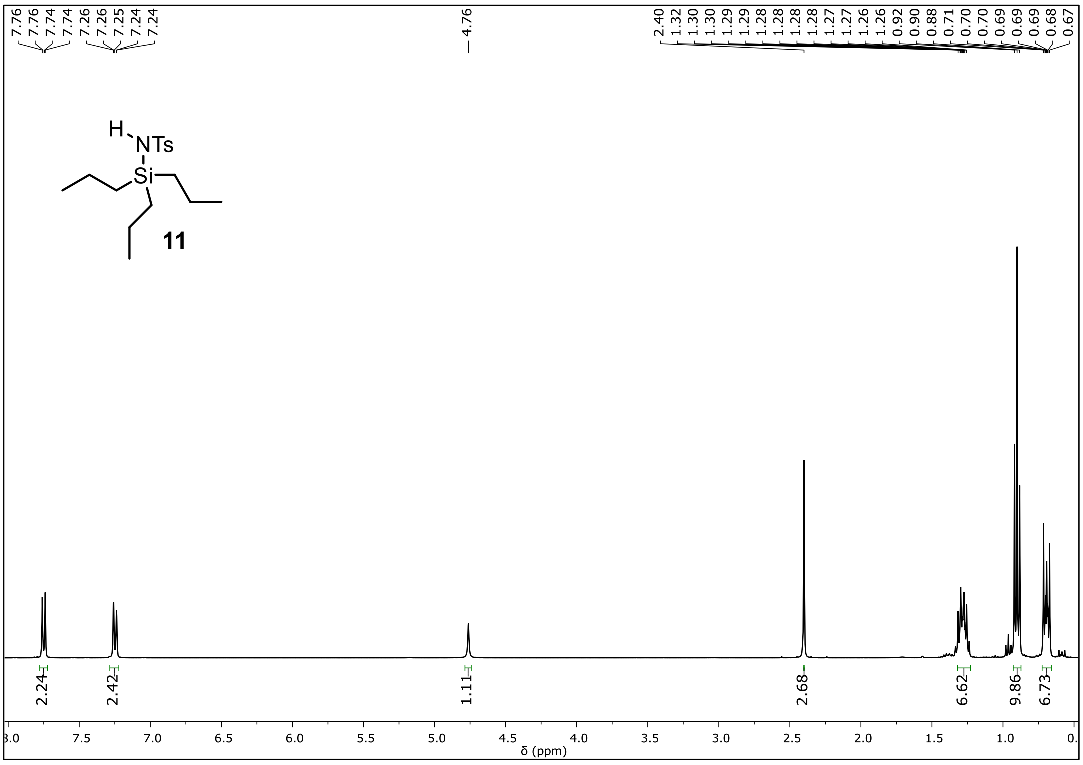

^{13}C{^{1}H} NMR spectrum for **11** (100 MHz, $CDCl_3$)

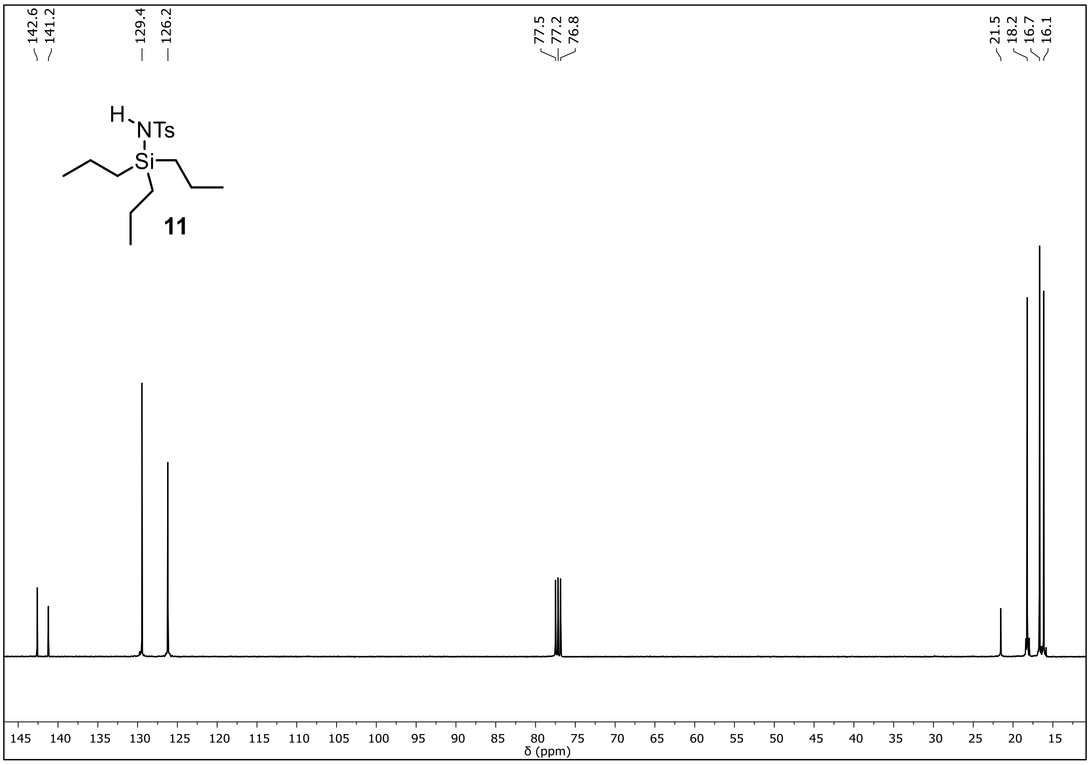

^{15}N NMR spectrum for **11** (81 MHz, $CDCl_3$)

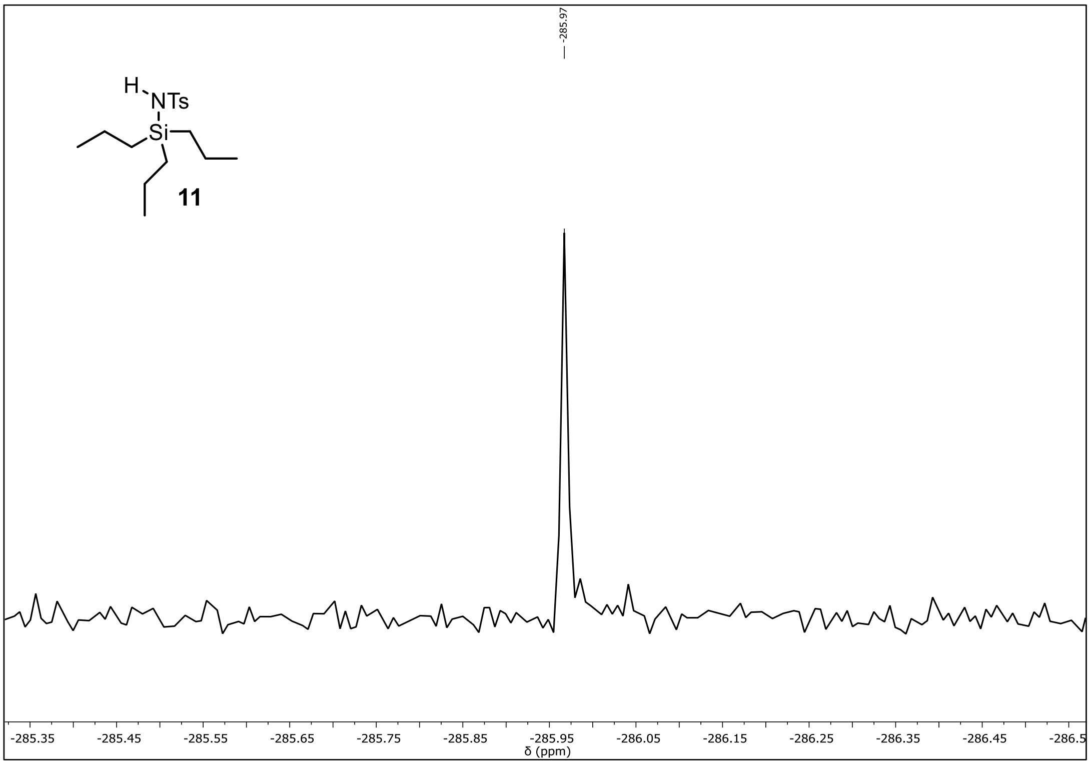

^{1}H NMR spectrum for **12** (400 MHz, $CDCl_3$)

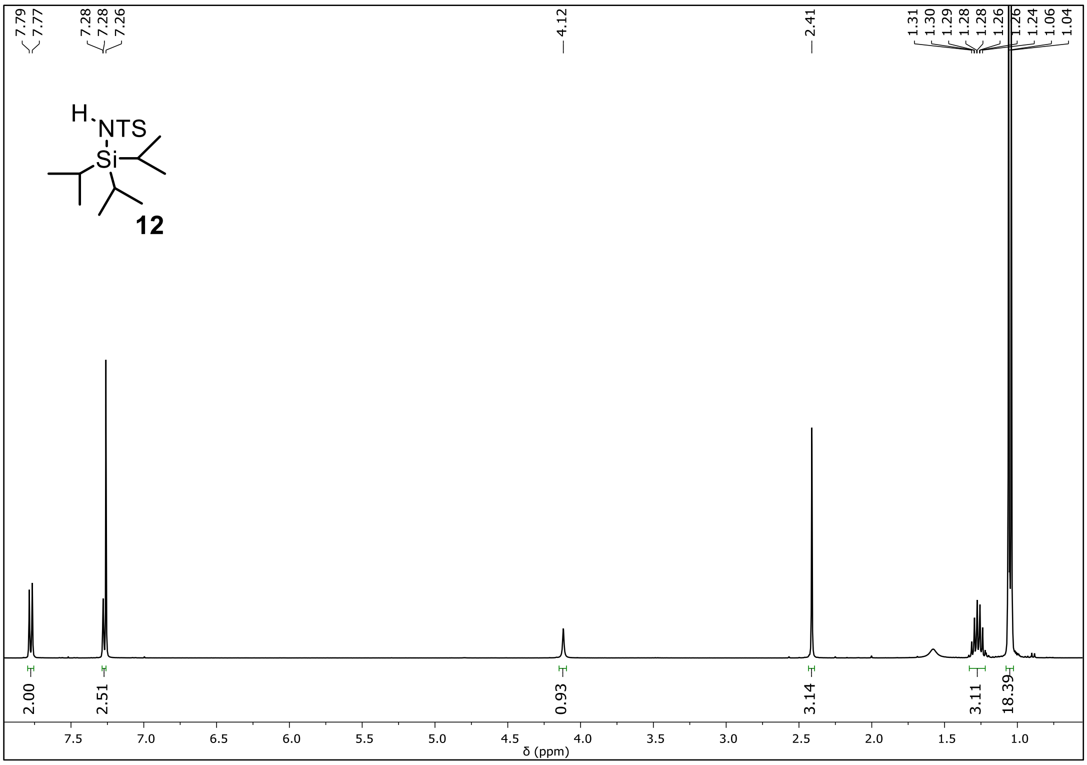

$^{13}C\{^{1}H\}$ NMR spectrum for **12** (100 MHz, $CDCl_3$)

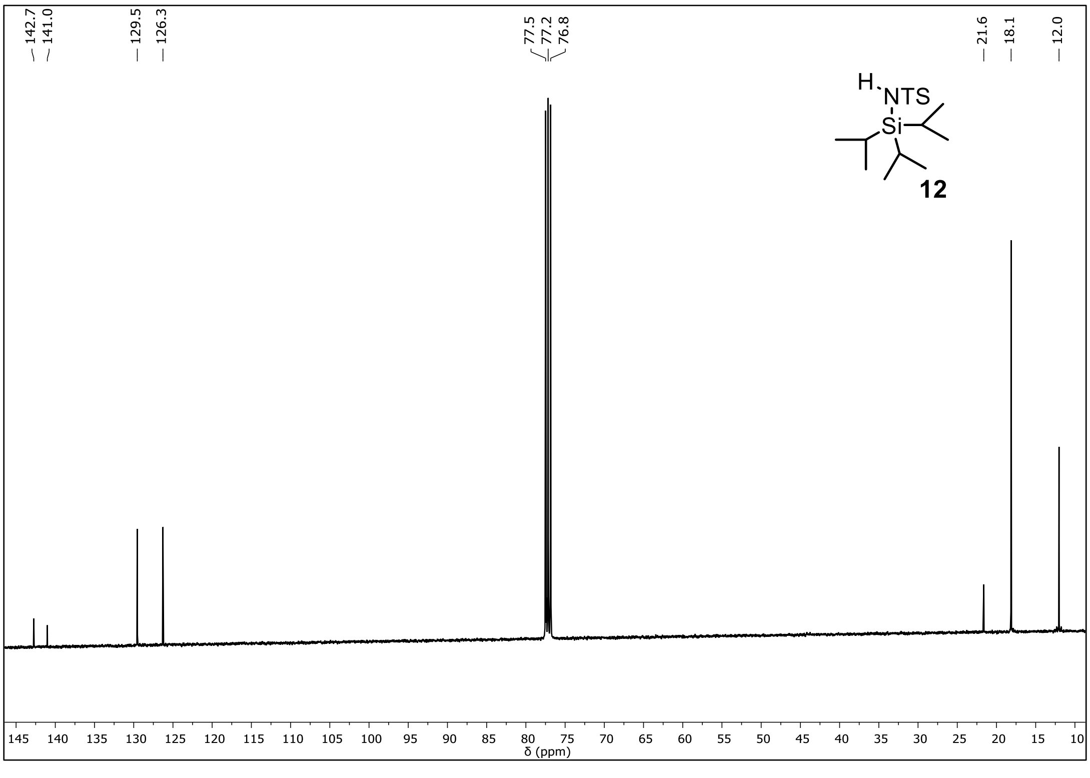

^{1}H NMR spectrum for **13** (400 MHz, $CDCl_3$)

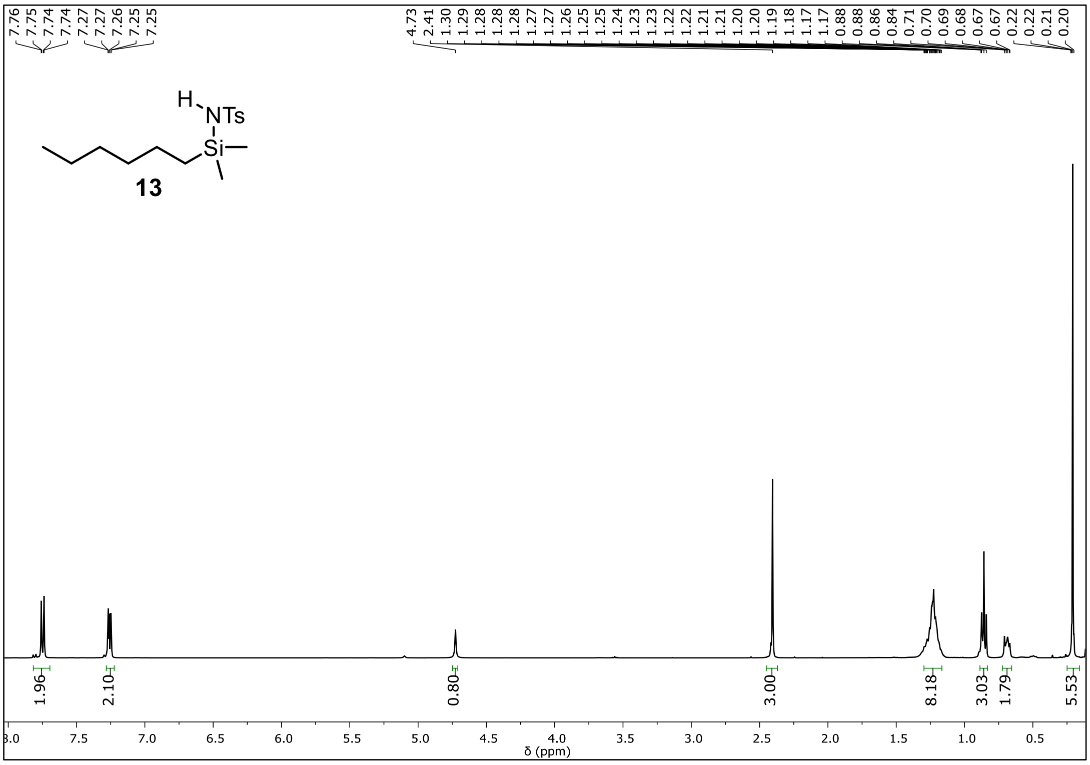

$^{13}C\{^{1}H\}$ NMR spectrum for **13** (100 MHz, $CDCl_3$)

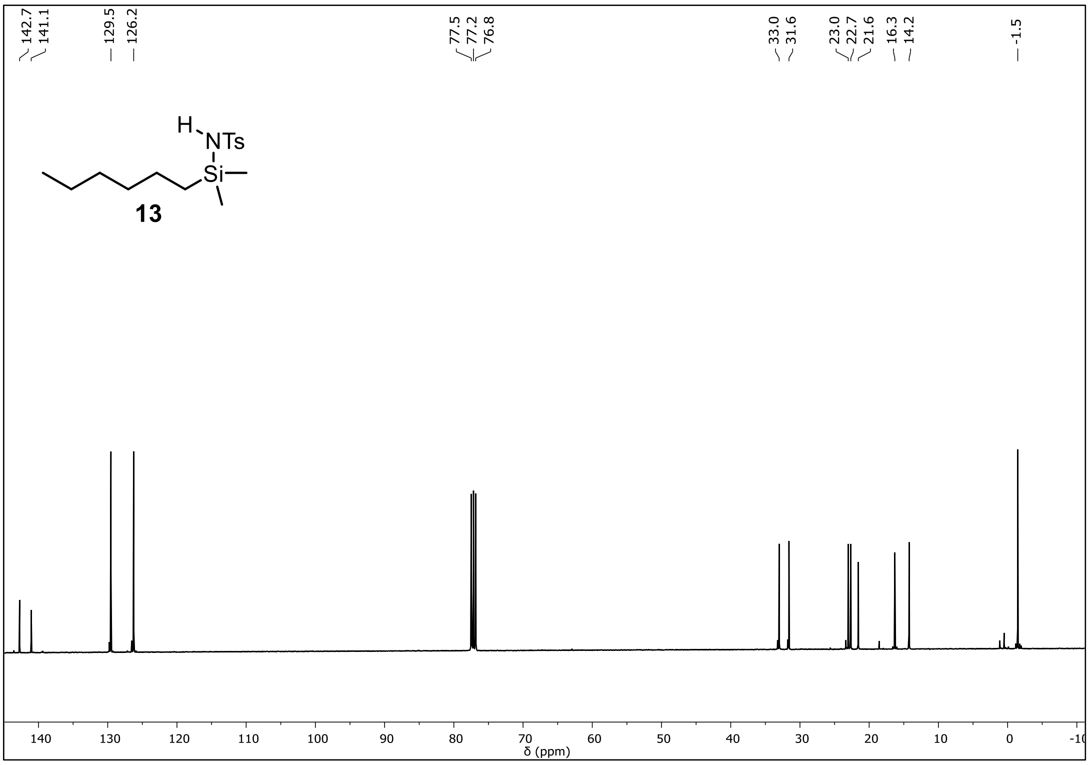

^{1}H NMR spectrum for **14** (400 MHz, $CDCl_3$)

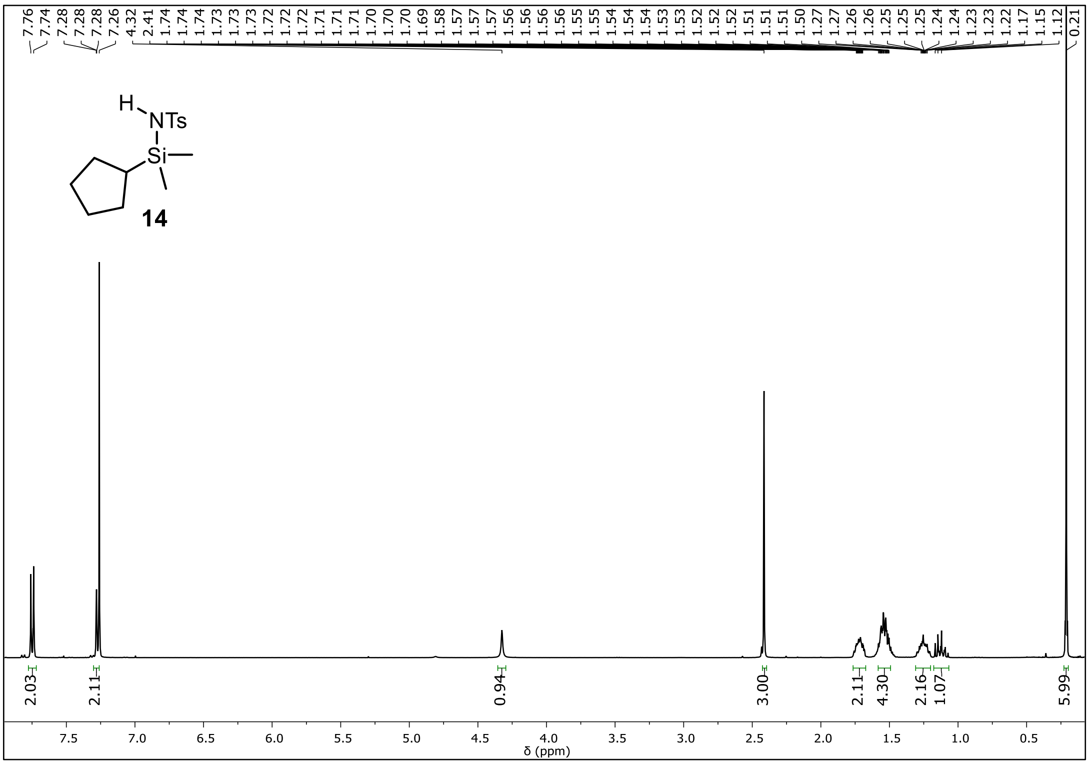

^{13}C{^{1}H} NMR spectrum for **14** (100 MHz, $CDCl_3$)

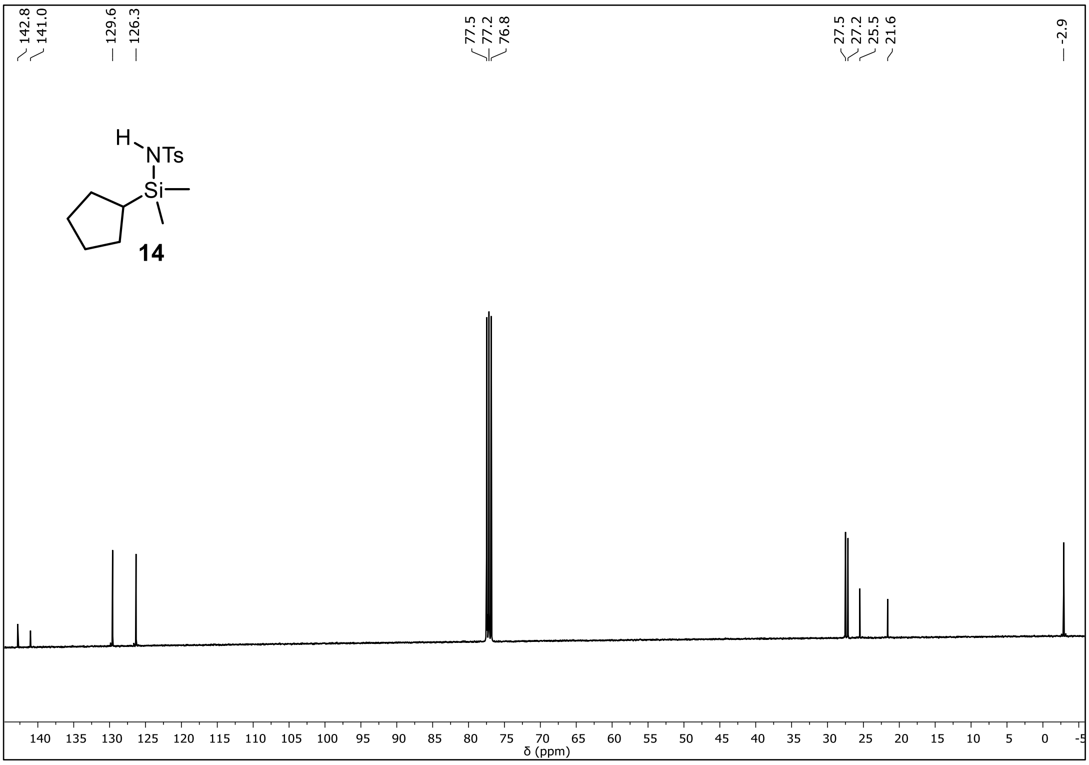

^{1}H NMR spectrum for **15** (400 MHz, $CDCl_3$)*

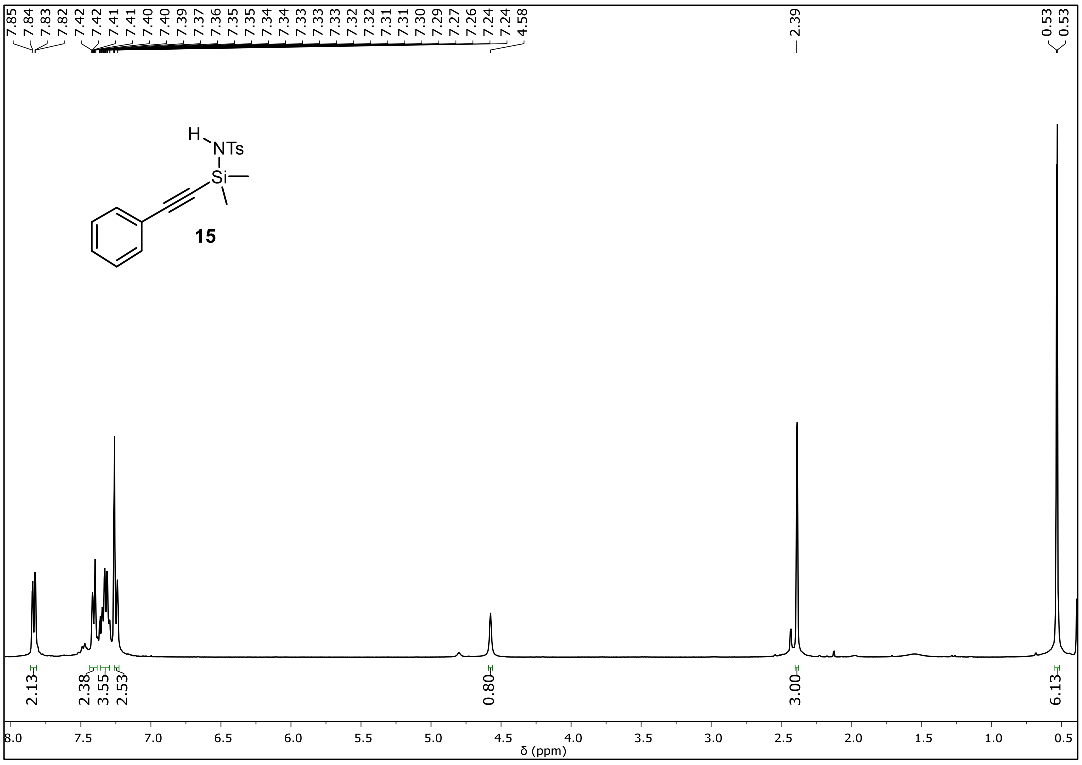

*5% $TsNH_2$ is observed.

$^{13}C\{^{1}H\}$ NMR spectrum for **15** (100 MHz, $CDCl_3$)

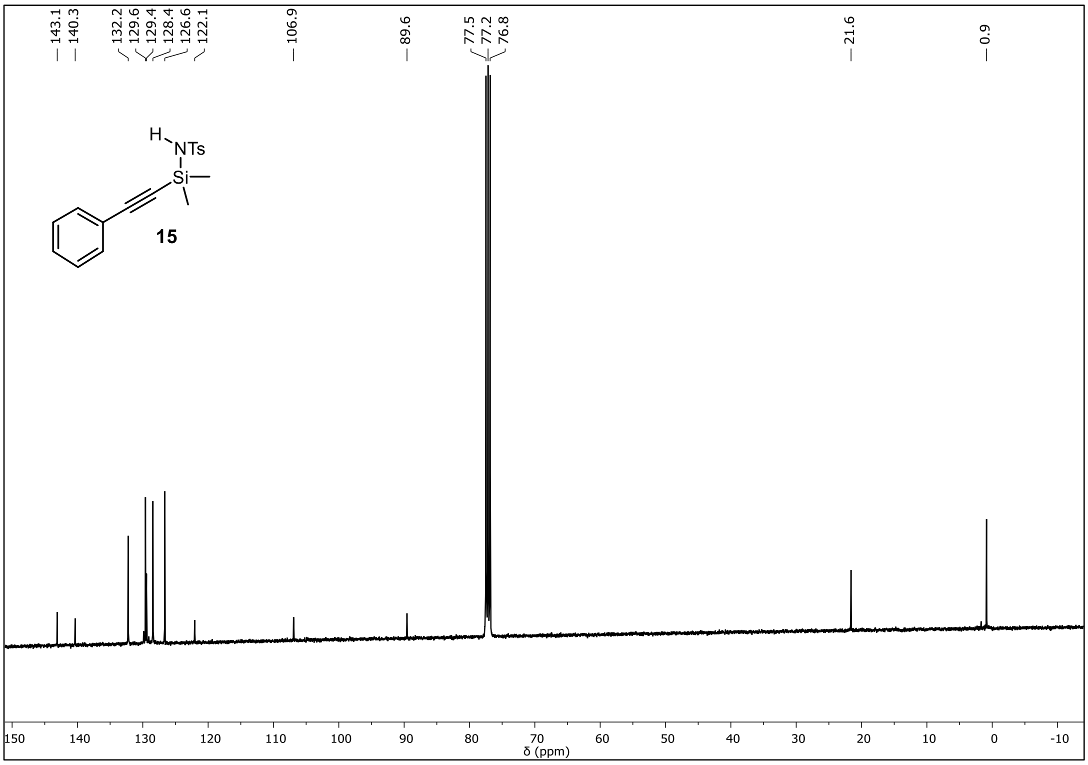

^{1}H NMR spectrum for **16** (400 MHz, $CDCl_3$)

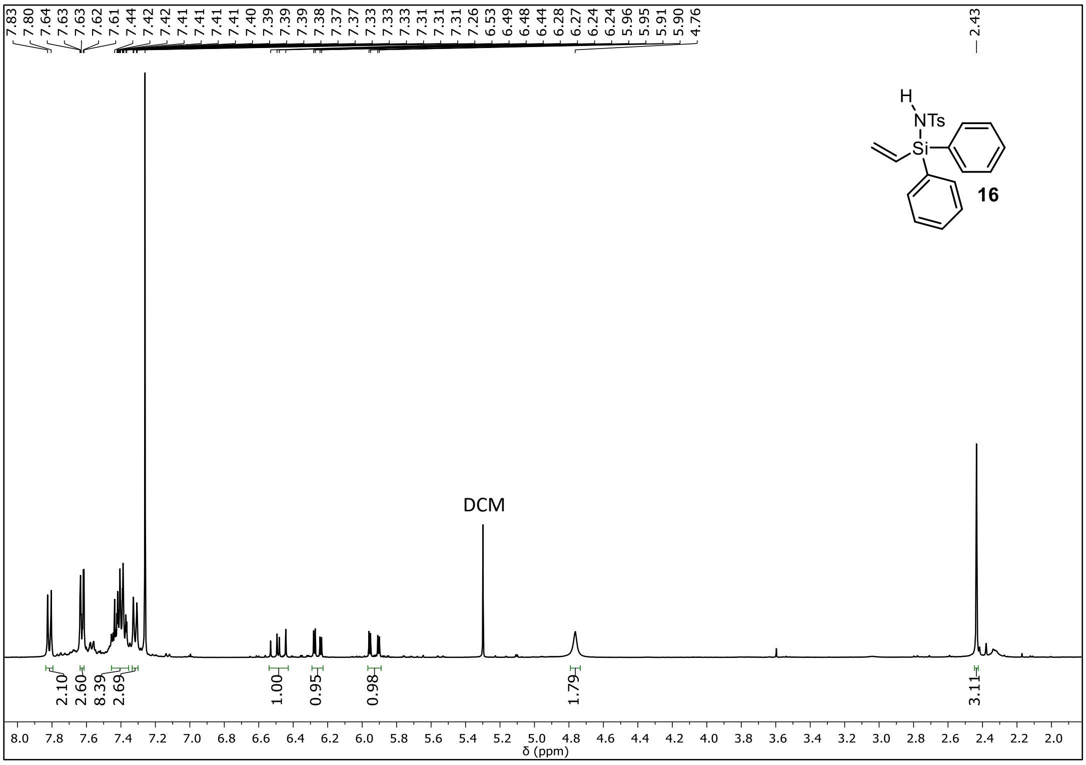

$^{13}C\{^1H\}$ NMR spectrum for **16** (100 MHz, $CDCl_3$)

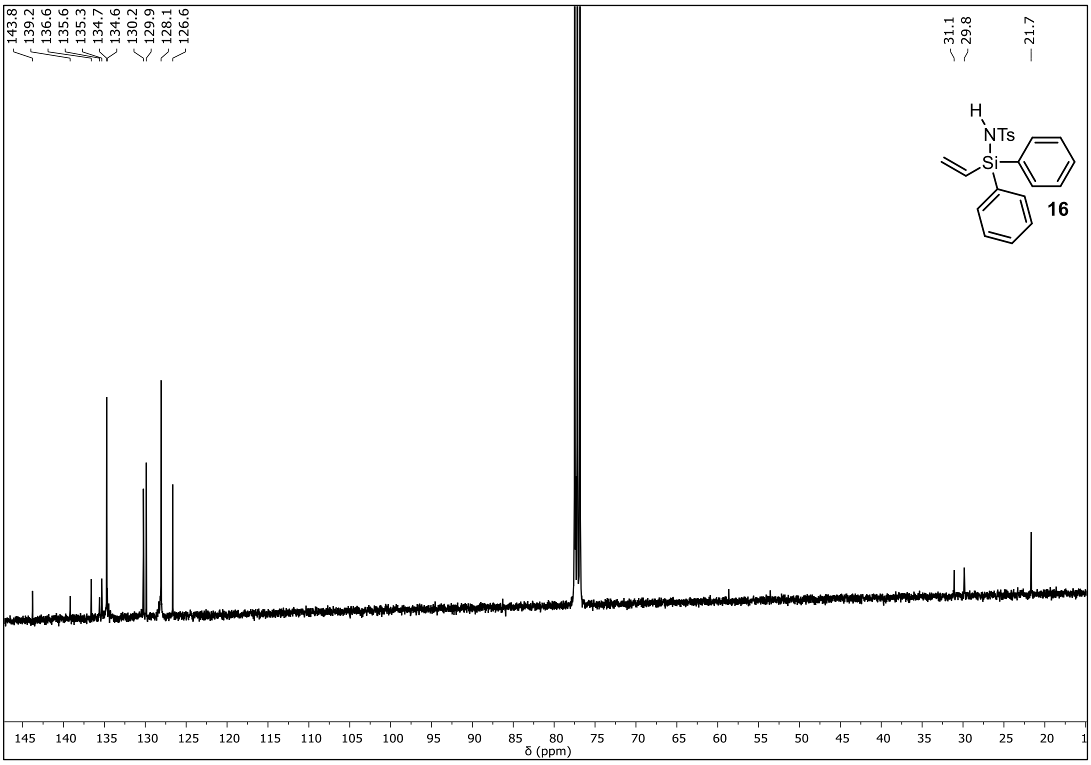

^{1}H NMR spectrum for **17** (400 MHz, $CDCl_3$)

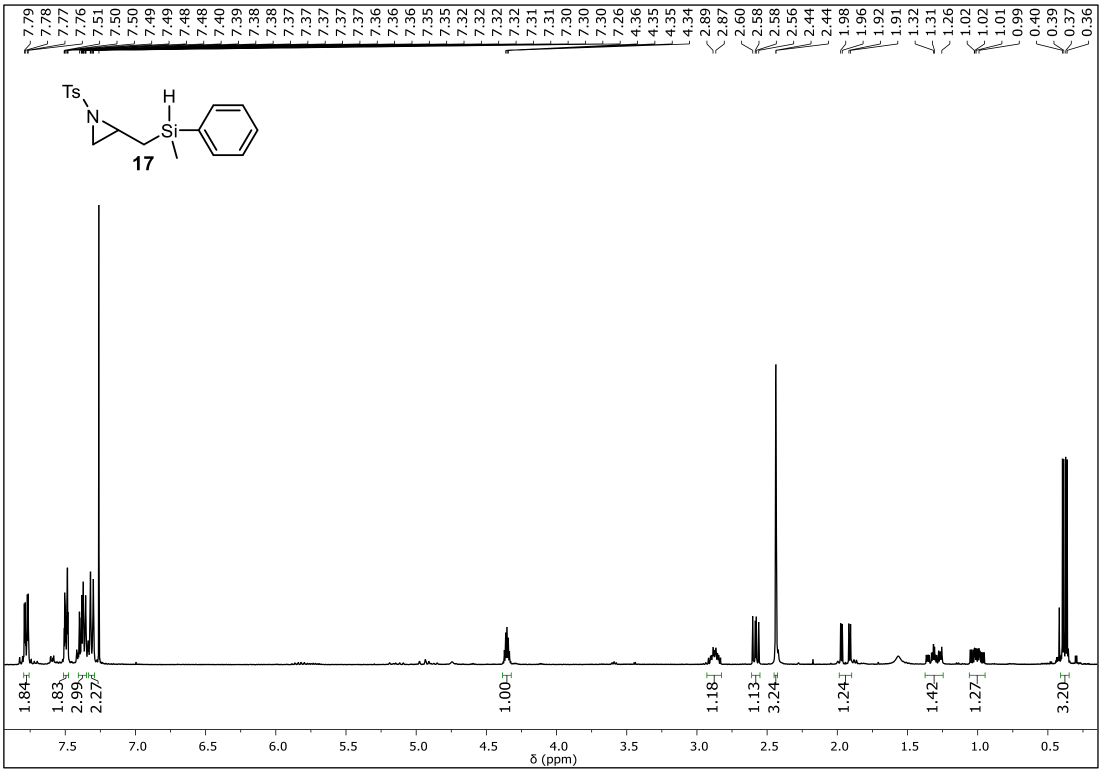

^{13}C{^{1}H} NMR spectrum for **17** (100 MHz, $CDCl_3$)

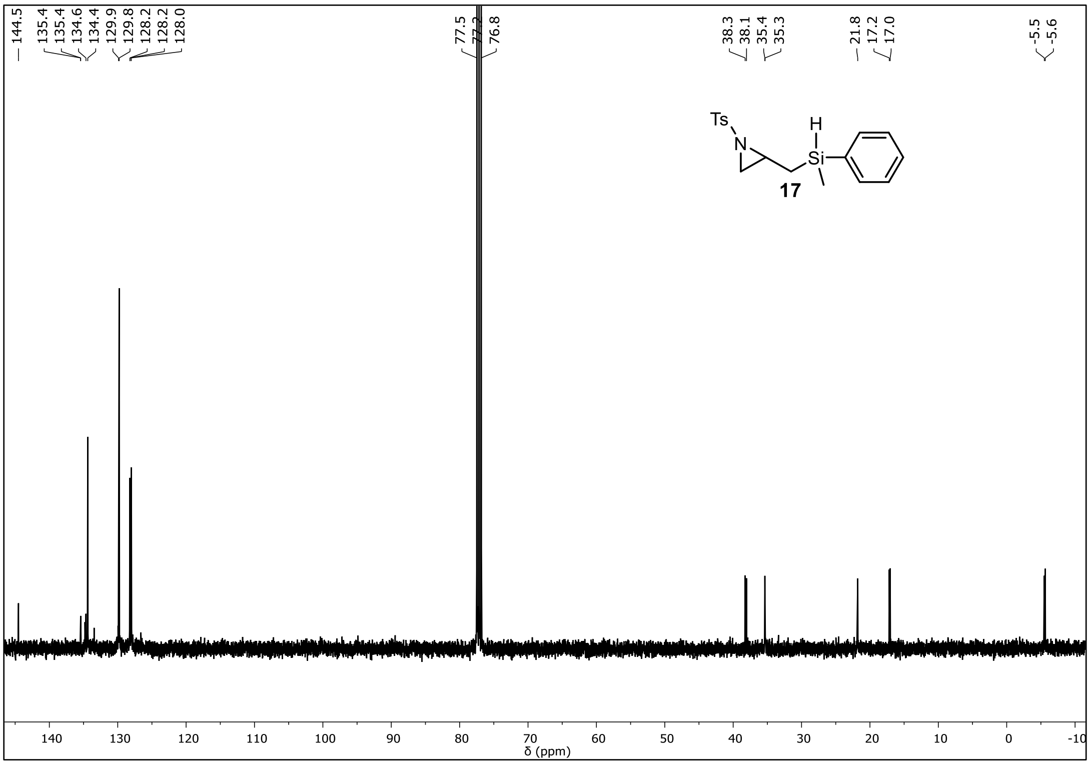

^{1}H NMR spectrum for **18** (400 MHz, $CDCl_3$)

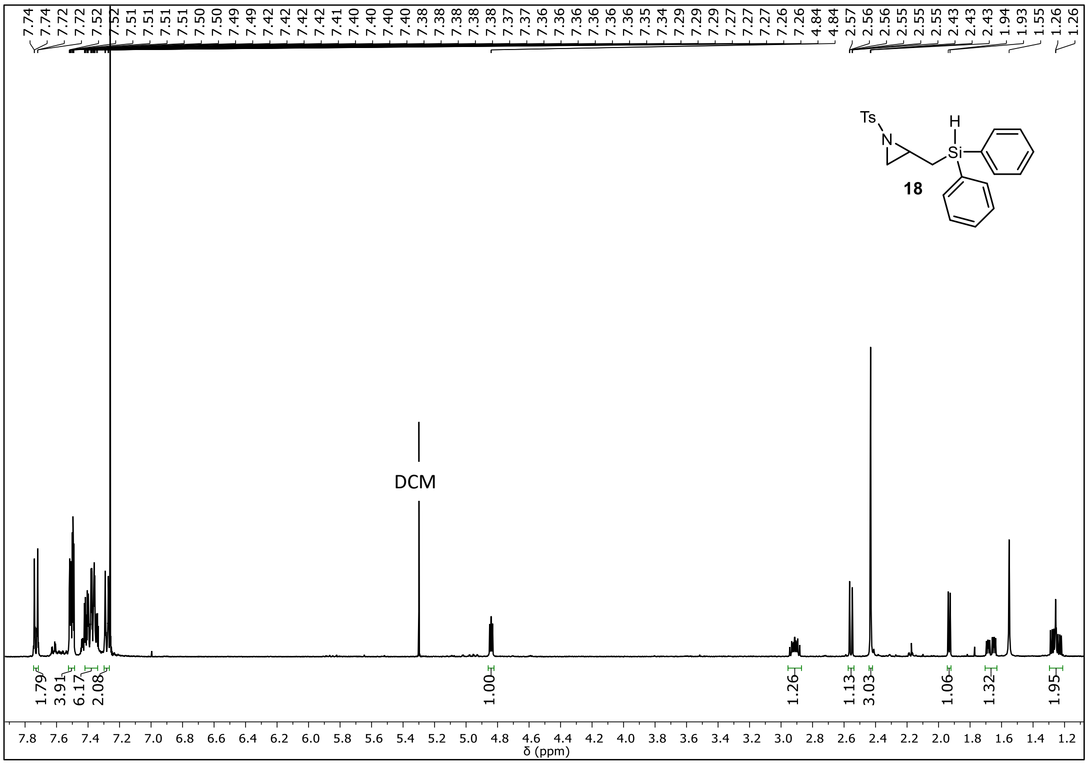

$^{13}C\{^{1}H\}$ NMR spectrum for **18** (100 MHz, $CDCl_3$)

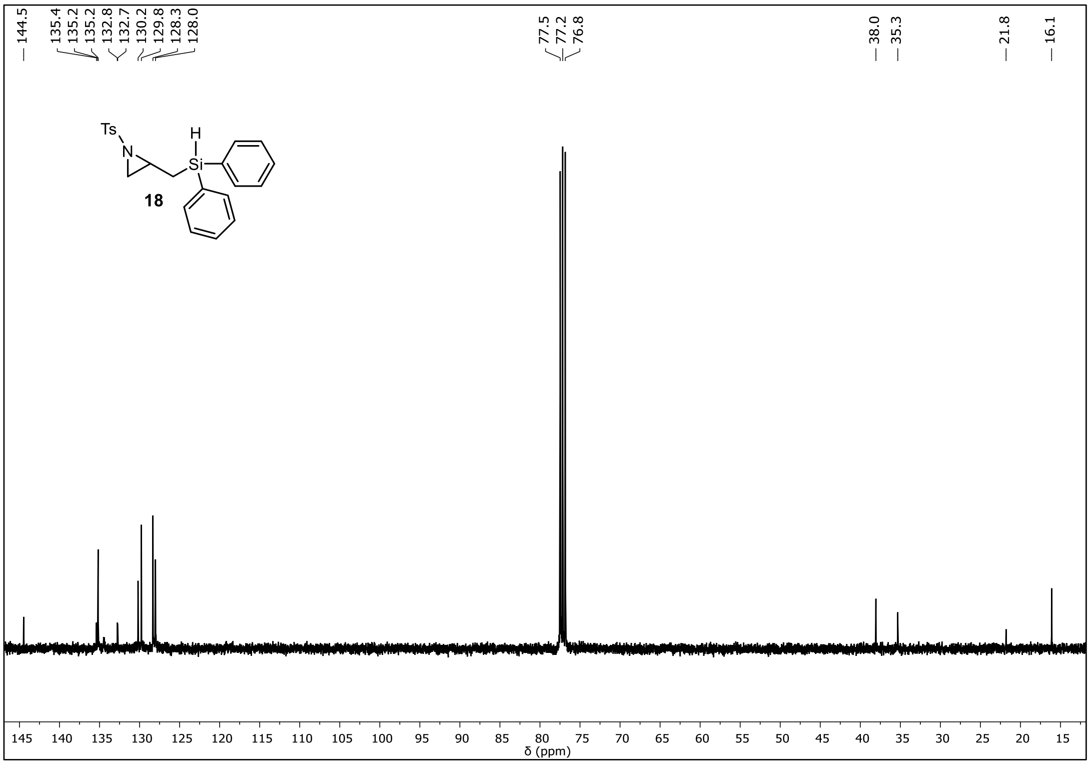

^{1}H NMR spectrum for **19** (400 MHz, $CDCl_3$)

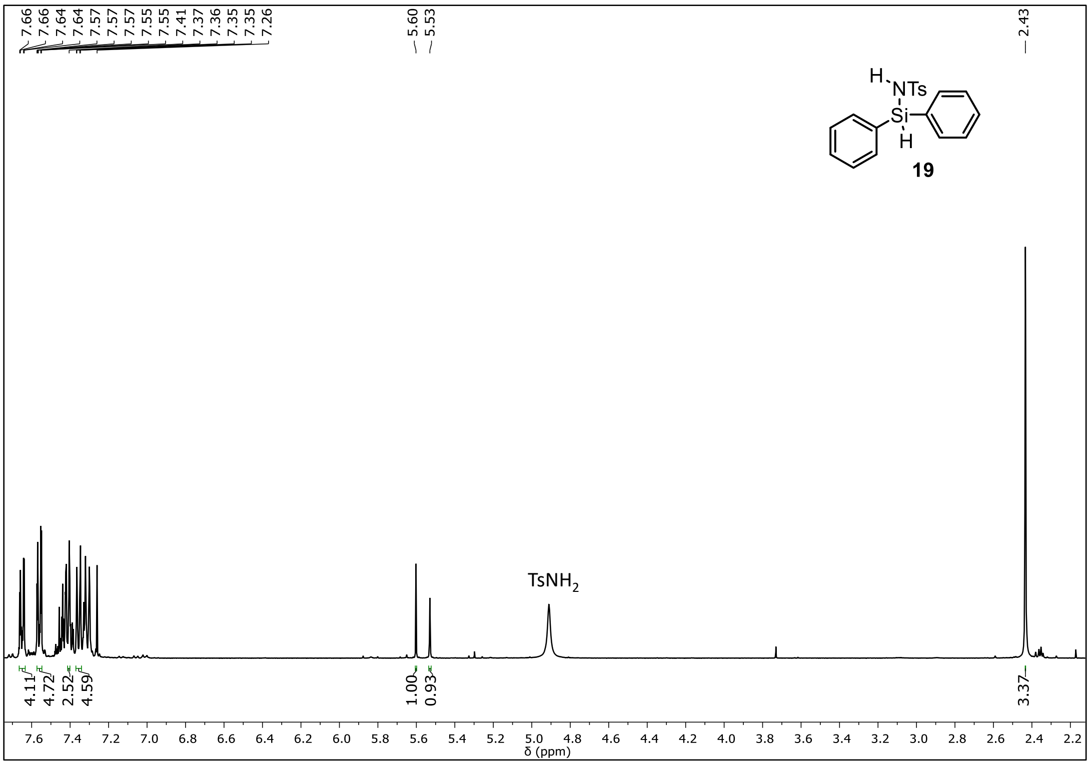

^{13}C{^{1}H} NMR spectrum for **19** (100 MHz, $CDCl_3$)

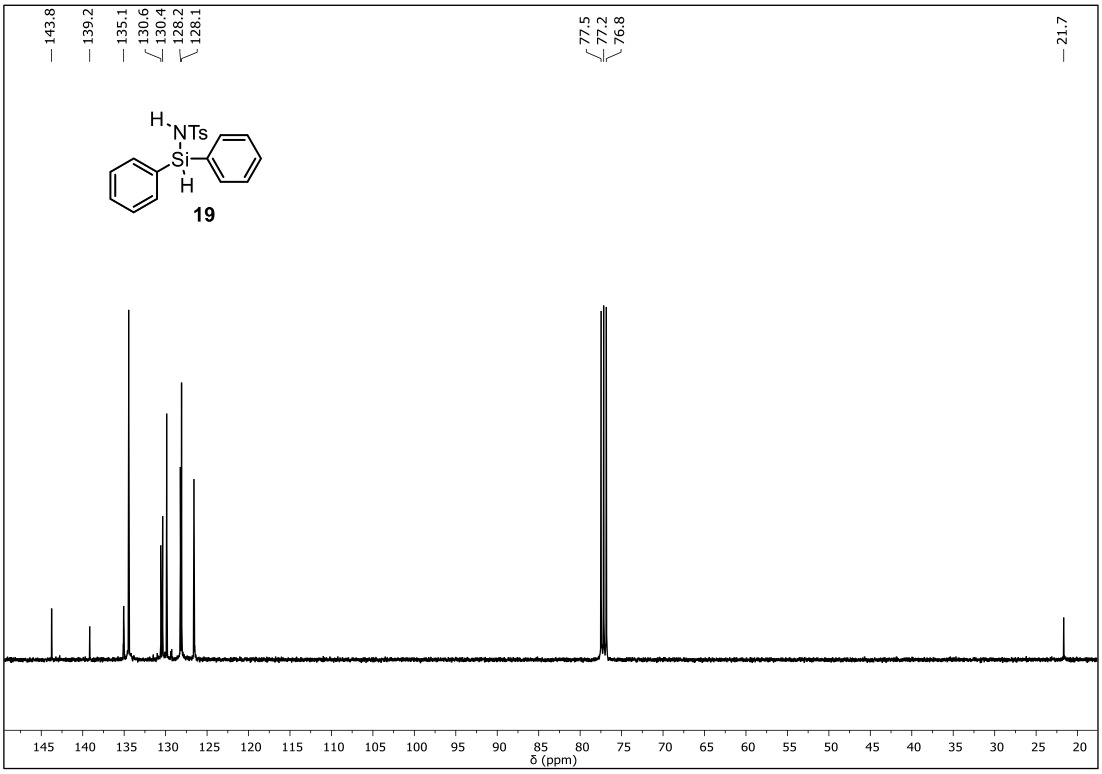

^{1}H NMR spectrum for **20** (400 MHz, $CDCl_3$)

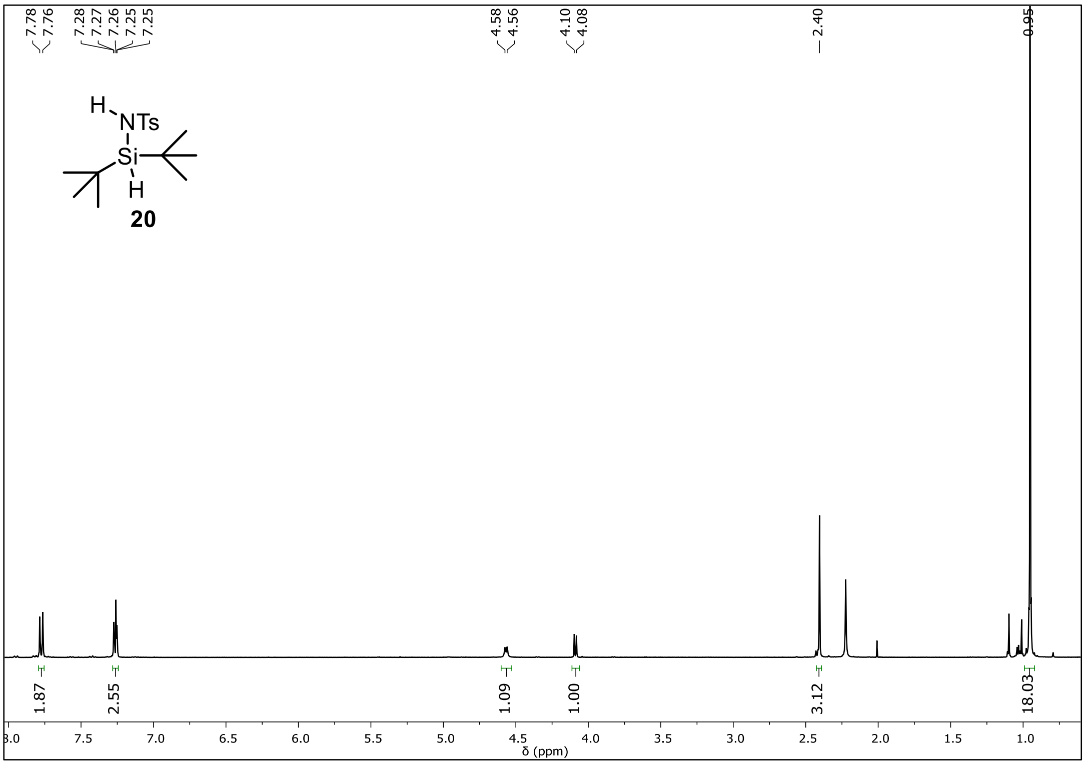

$^{13}C\{^{1}H\}$ NMR spectrum for **20** (100 MHz, $CDCl_3$)

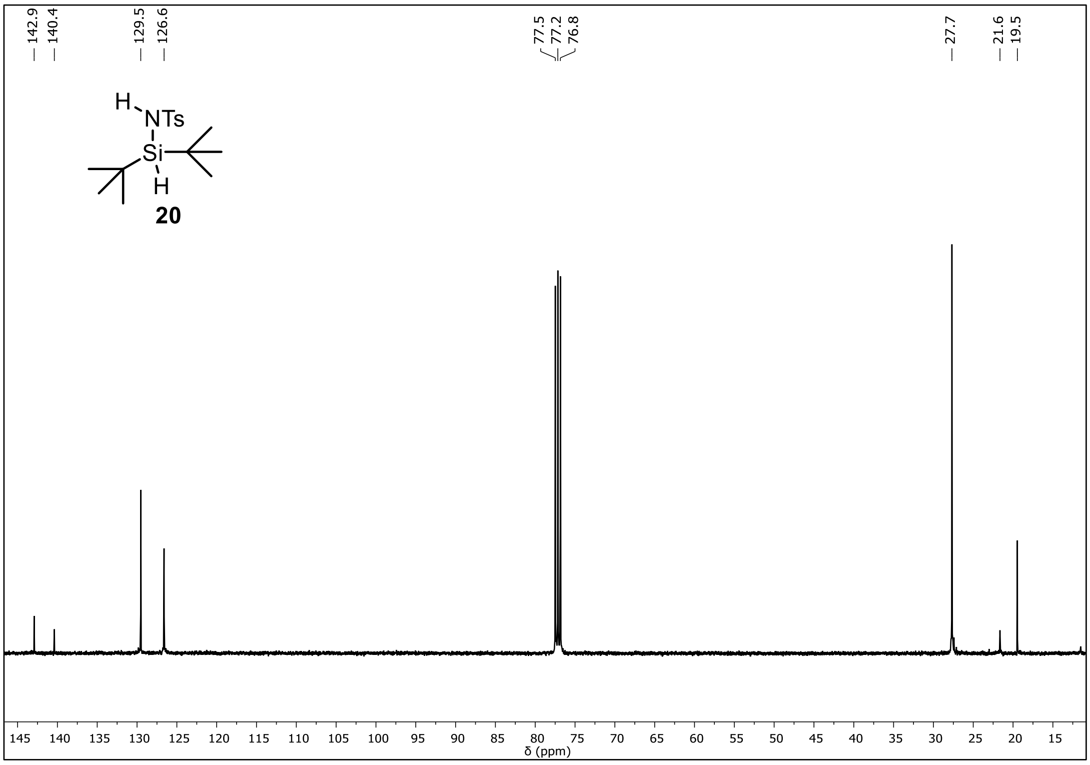

^{1}H NMR spectrum for **21** (400 MHz, $CDCl_3$)

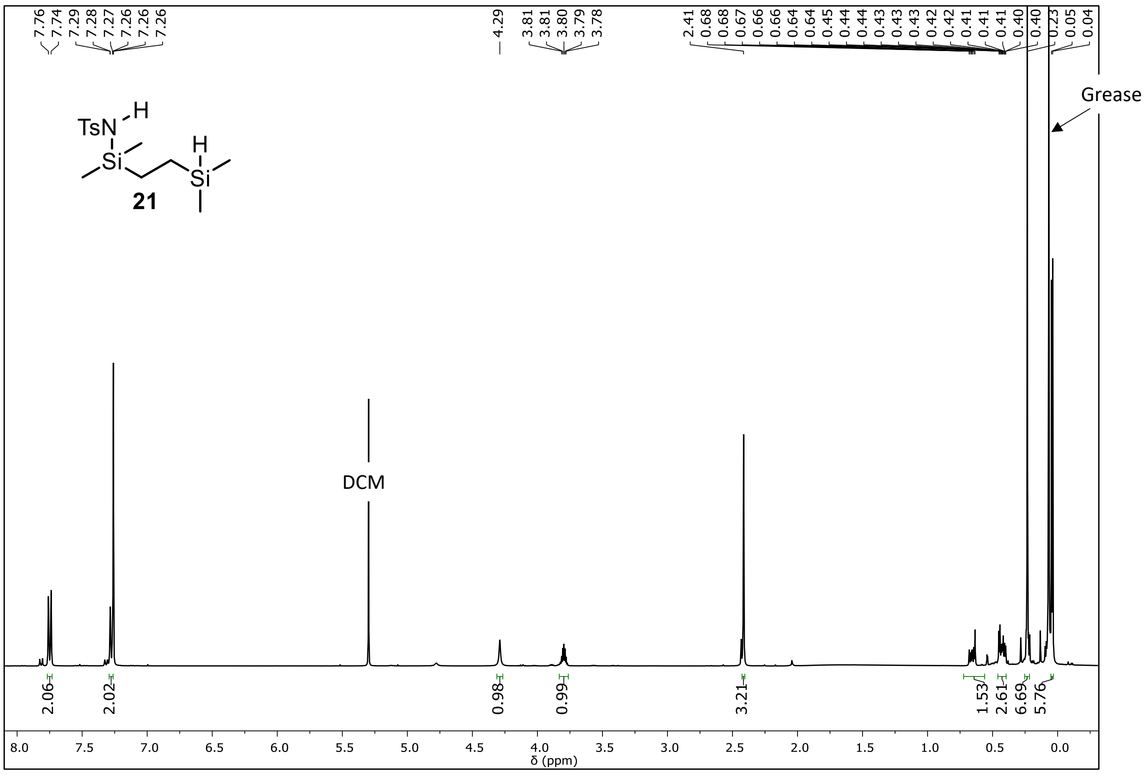

^{13}C{^{1}H} NMR spectrum for **21** (100 MHz, $CDCl_3$)

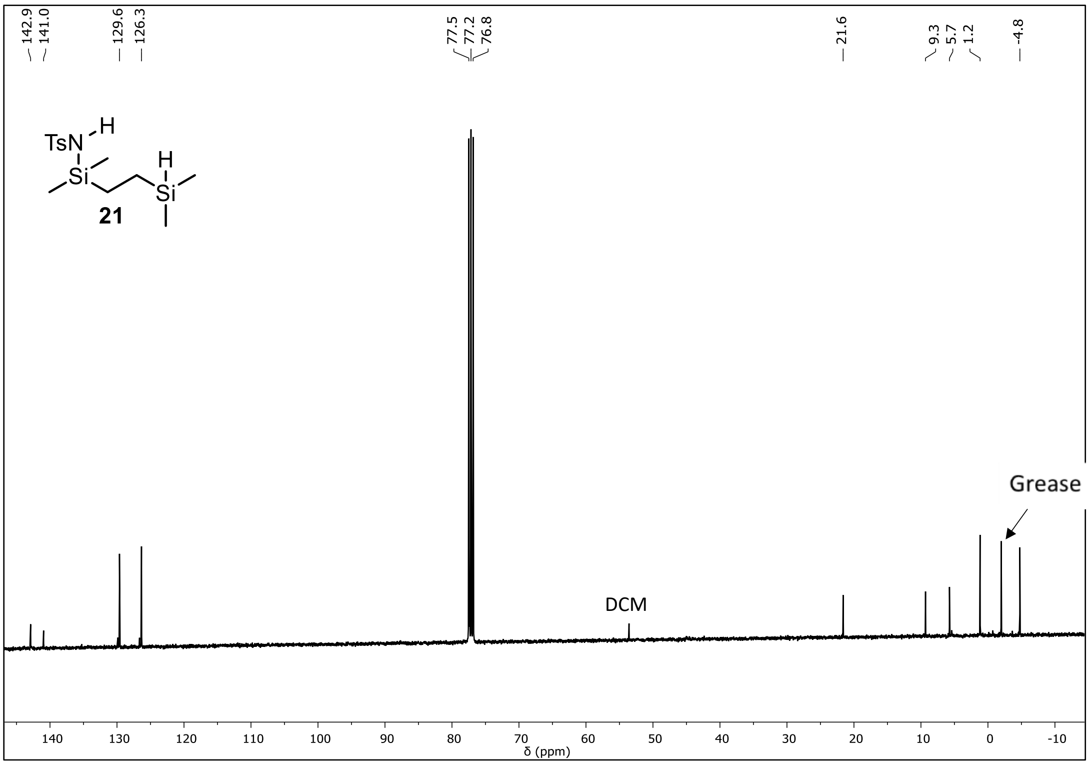

^{1}H NMR spectrum for **22** (500 MHz, $CDCl_3$)

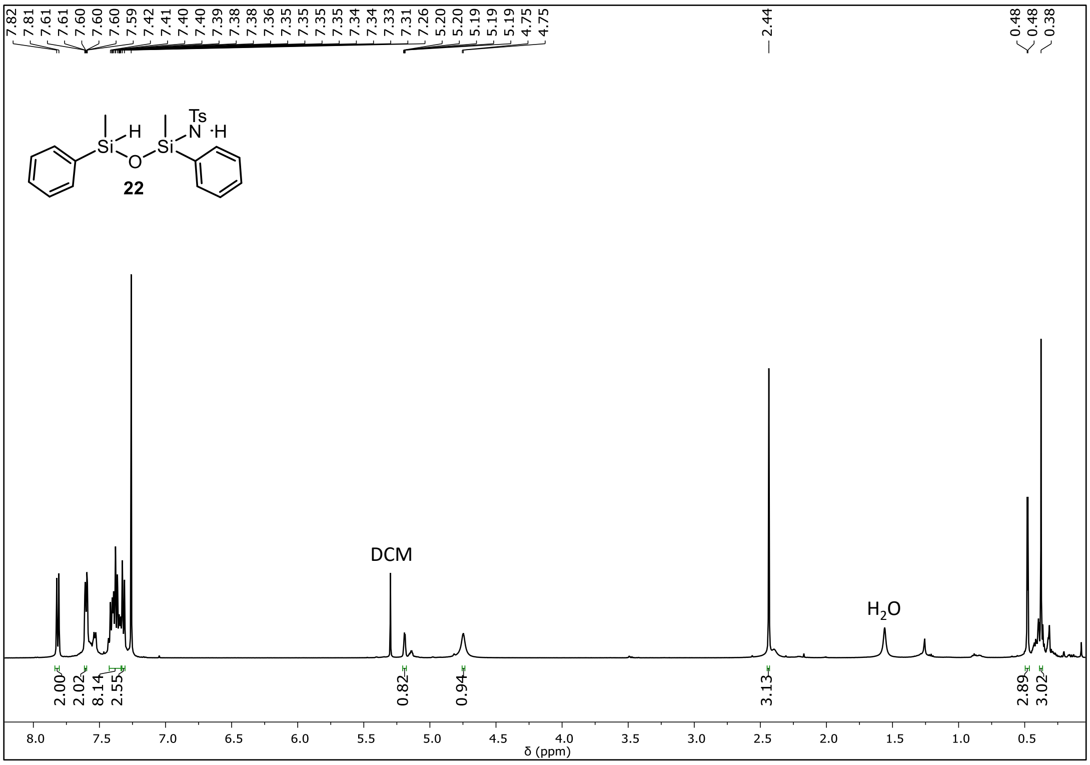

$^{13}C\{^{1}H\}$ NMR spectrum for **22** (125 MHz, $CDCl_3$)

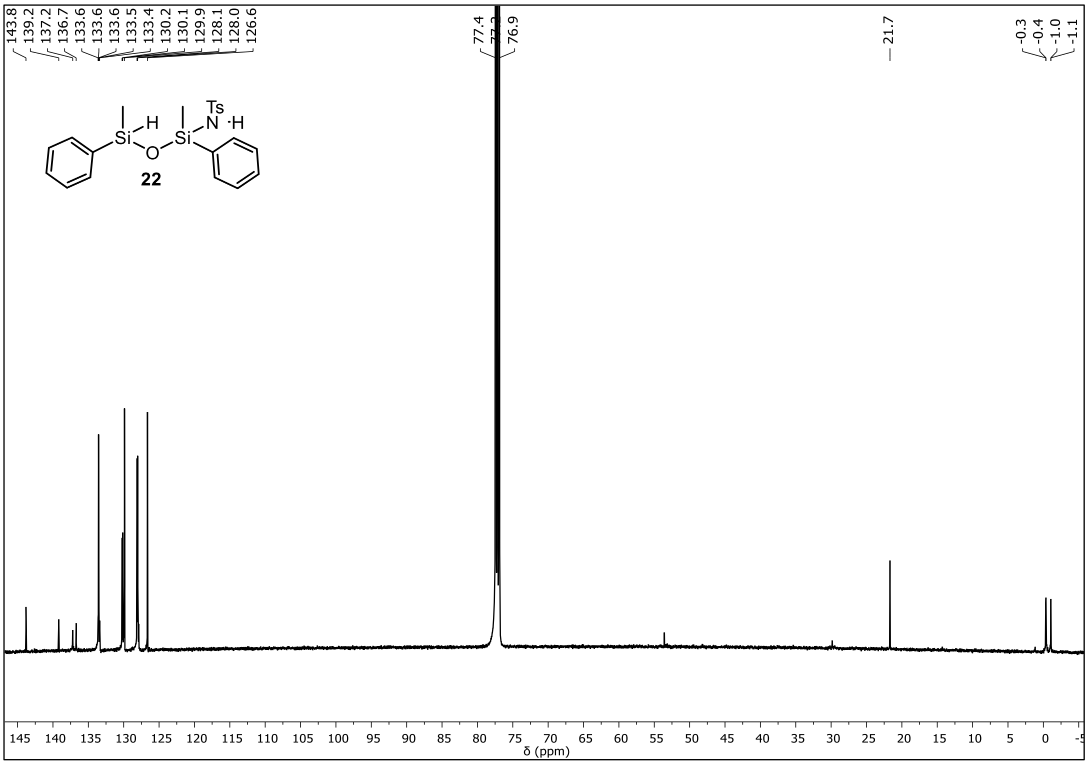

^{1}H NMR spectrum for **23** (400 MHz, $CDCl_3$)

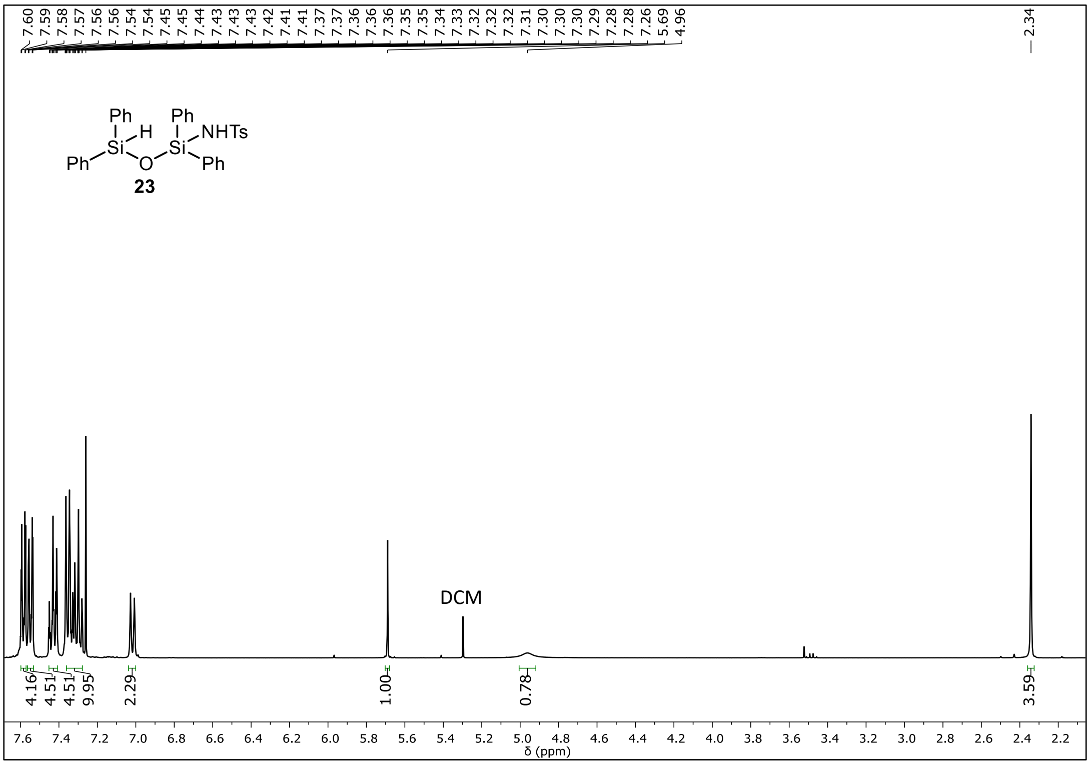

$^{13}C\{^{1}H\}$ NMR spectrum for **23** (100 MHz, $CDCl_3$)

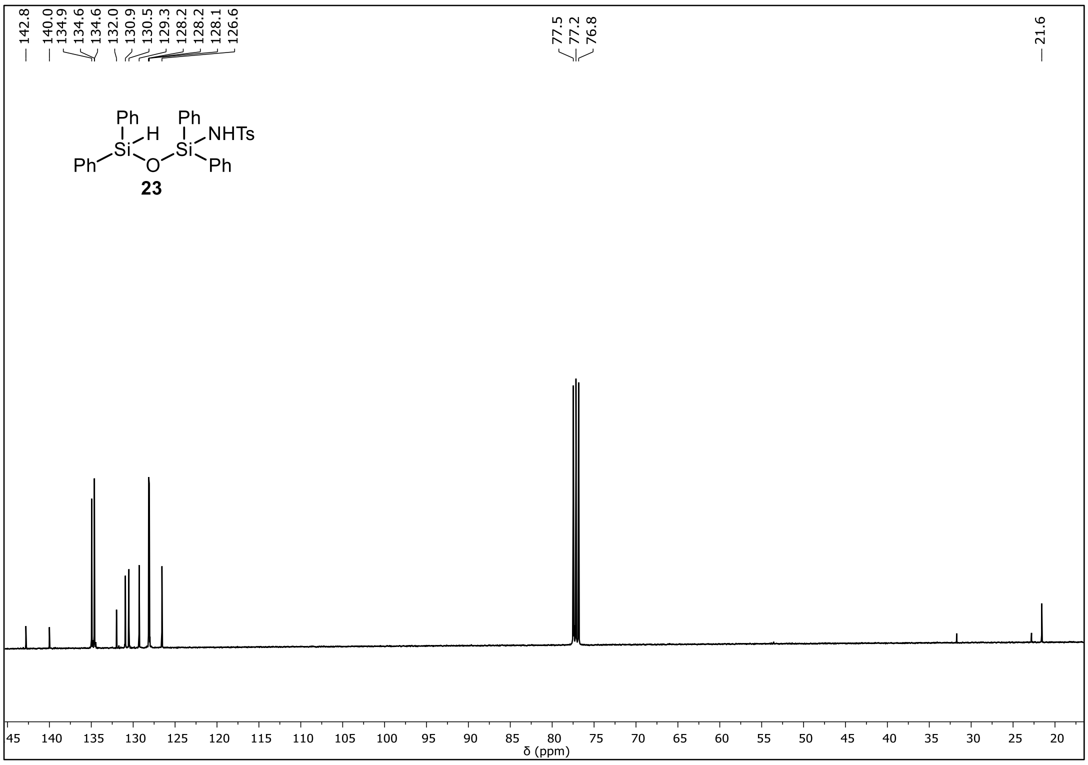

^{1}H NMR spectrum for **24** (400 MHz, $CDCl_3$)

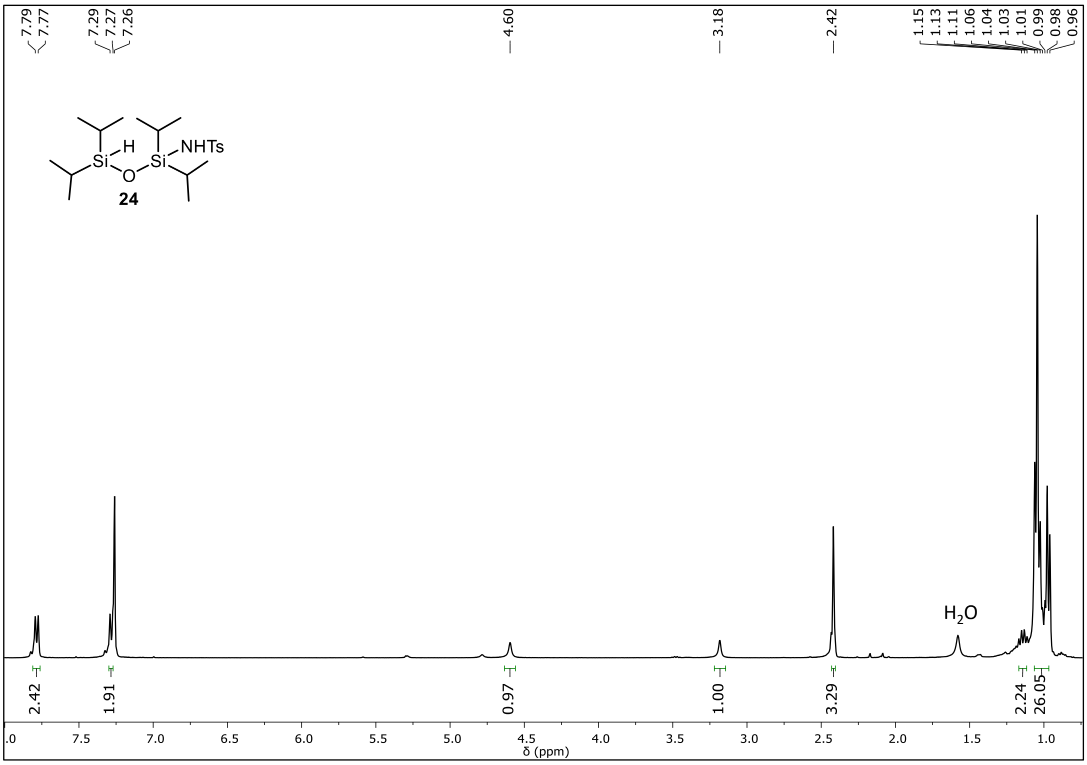

$^{13}C\{^{1}H\}$ NMR spectrum for **24** (100 MHz, $CDCl_3$)

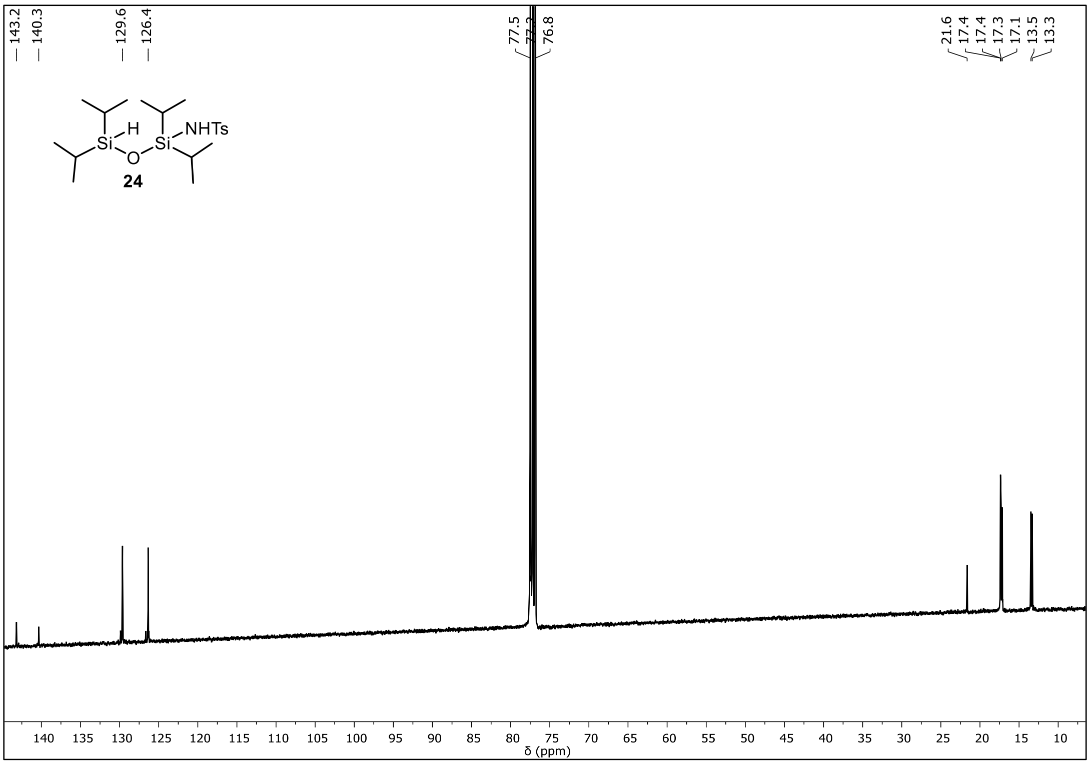

6. References for experimental section

(1) Mairena, M. A.; Urbano, J.; Carbajo, J.; Maraver, J. J.; Álvarez, E; Díaz-Requejo, M. M.; Pérez, P. J. Effects of the Substituents in the Tp^xCu Activation of Dioxygen: An Experimental Study. *Inorg. Chem.* **2007**, *46*, 7428-7435.

(2) Gómez-Emeterio, B. P.; Urbano, J.; Díaz-Requejo, M. M.; Pérez, P. J. Easy Alkane Catalytic Functionalization. *Organometallics* **2008**, *27*, 4126-4130.

(3) Santoro, O.; Collado, A.; Slawin, A. M. Z.; Nolan, S. P.; Cazin, C. S. J. A General Synthetic Route to [Cu(x)(NHC)] (NHC = N-heterocyclic carbene, X = Cl, Br, I) Complexes. *Chem. Commun*. **2013**, *49*, 10483-10485.

(4) Fructos, M. R.; Belderrain, T. R.; De Frémont, P.; Scott, N. M.; Nolan, S. P.; Díaz-Requejo, M. M.; Pérez, P. J. A Gold Catalyst for Carbene-Transfer Reactions from Ethyl Diazoacetate. *Angew. Chem.* **2005**, *44*, 5284-5288.

(5) Yamada, Y.; Yamamoto, T.; Okawara, M. Synthesis and Reaction of New Type I-N Ylide, N-Tosyliminoiodinane. *Chem. Lett.* **1975**, 361-362.

(6) Harris, R. K.; Becker, E. D.; Cabral de Menezes, S. M.; Goodfellow, R.; Granger, P. NMR Nomenclature. Nuclear Spin Properties and Conventions for Chemical Shifts. *Pure Appl. Chem*. **2001**, *73*, 1795-1818.

(7) Savela, R.; Zawartka, W.; Leino, R. Iron-Catalyzed Chlorination of Silanes. *Organometallics* **2012**, *31*, 3199-3206.

(8) Hirone, N.; Sanjiki, H.; Tanaka, R.; Hata, T.; Urabe, H. Acceleration of the Substitution of Silanes with Grignard Reagents by Using either LiCl or YCl_3/MeLi. *Angew. Chem. Int. Ed.* **2010**, *49*, 7762-7764.

(9) Wang, K.; Zhou, J.; Jiang, Y.; Zhang, M.; Wang, C.; Xue, D.; Tang, W.; Sun, H.; Xiao, J.; Li, C. Selective Manganese-Catalyzed Oxidation of Hydrosilanes to Silanols under Neutral Reaction Conditions. *Angew. Chem. Int. Ed.* **2019**, *58*, 6380-6384.

(10) Suzuki, T.; Lo, P. Y. New Aspects of Platinum-Catalyzed hydrosilylation of disilylethynes. *J. Organomet. Chem.* **1990,** *391*, 19-25.

(11) Sridhar, M.; Ramanaiah, B. C.; Narsaiah, C.; Swamy, M. K.; Mahesh, B.; Reddy, M. K. K. An Efficient and Simple Method for the preparation of Symmetrical Disiloxanes from Hydrosilanes by Lewis Acid-Catalyzed Air Oxidation. *Tetrahedron Letters* **2009**, *50*, 7166-7168.

(12) Kelly, A. T.; Franz, A. K. Metal-Free Synthesis of 1,3-Disoloxanediols and Aryl Siloxanols. *ACS Omega* **2019**, *4*, 6295-6300.

(13) Straus, D. A.; Zhang, C.; Quimbita, G. E.; Grumbine, S. D.; Heyn, R. H.; Tilley, T. D.; Rheingold, A. L.; Geib, S. J. Silyl and Diphenylsilylene Derivates of (η^5-C_5Me_5)(PMe_3)$_2$Ru. Evidence for the Base-Free Silylene Complex [(η^5-C_5Me_5)(PMe_3)$_2$Ru=$SiPh_2$]. *J. Am. Chem. Soc.* **1990**, *112*.
(14) Shankar, R.; Sharma, A.; Jangir, B.; Chaudhary, M.; Kociok-Köhn, G. Catalytic Oxidation of Diorganosilanes to 1,1,3,3-tetraorganodisiloxanes with Gold Nanoparticle Assembly at the Water-Chloroform Interface. *New J. Chem*. **2019**, *43*, 813-819.
(15) Chang, Y. H.; Chiu, F.-T.; Zon, G. Investigation of Thermally Induced α-Deoxysilylation of Organosilylated Hydroxyalmine Derivates as a General Method for Nitrene Production. *J. Org. Chem.* **1981**, *46*, 342-354.

7. Computational Data

Computational Details

DFT calculations were carried out with Gaussian09 (Revision D.01)[1] with a grid of 99 radial shells and 590 angular points per shell ("Ultrafine" grid in Gaussian) using B3LYP-D3[2] (where D3 stands for Grimme's empirical dispersion GD3[3]) and implicit SMD[4] solvation for dichloromethane (DCM). The structures were optimized using a smaller basis set, BS1, coupled with a frequency calculation to ensure its minima/transition state nature and then single point (SP) calculations were carried out with a larger basis set, BS2. The BS1 basis set for Cu, Br is LANL2DZ (ECP)[5] with additional shells of d and p polarization functions[6]; and for the remaining elements is 6-31G(d,p).[7] BS2 set corresponds to def2-TZVP basis set for all elements but Cu, where a def2-QZVP with a def2 ECP was used.[8] Free energy corrections were obtained using the Goodvibes software[9] with a frequency cutoff of 50 cm^{-1} for Grimme's quasi-harmonic approximations[10] and a reference state correction to 1M.[11] Automation of the information extraction from the output files such as potential energies and mulliken spin values was carried out using the pyssian library.[12]

MECP calculations were carried out at the same level of theory as optimizations using the easymecp software[13] which is a wrapper of the software developed by Harvey et al.[14] As the difference in geometry between the triplet state minima optimized from the MECP is really close to the geometry of the MECP free energy corrections of the triplet were applied to the MECP to estimate its free energy. SP calculations were carried out in triplet and singlet states with BS2 on the MECP and the value presented in the main text comes from an average of both values.

Overall free energy profile

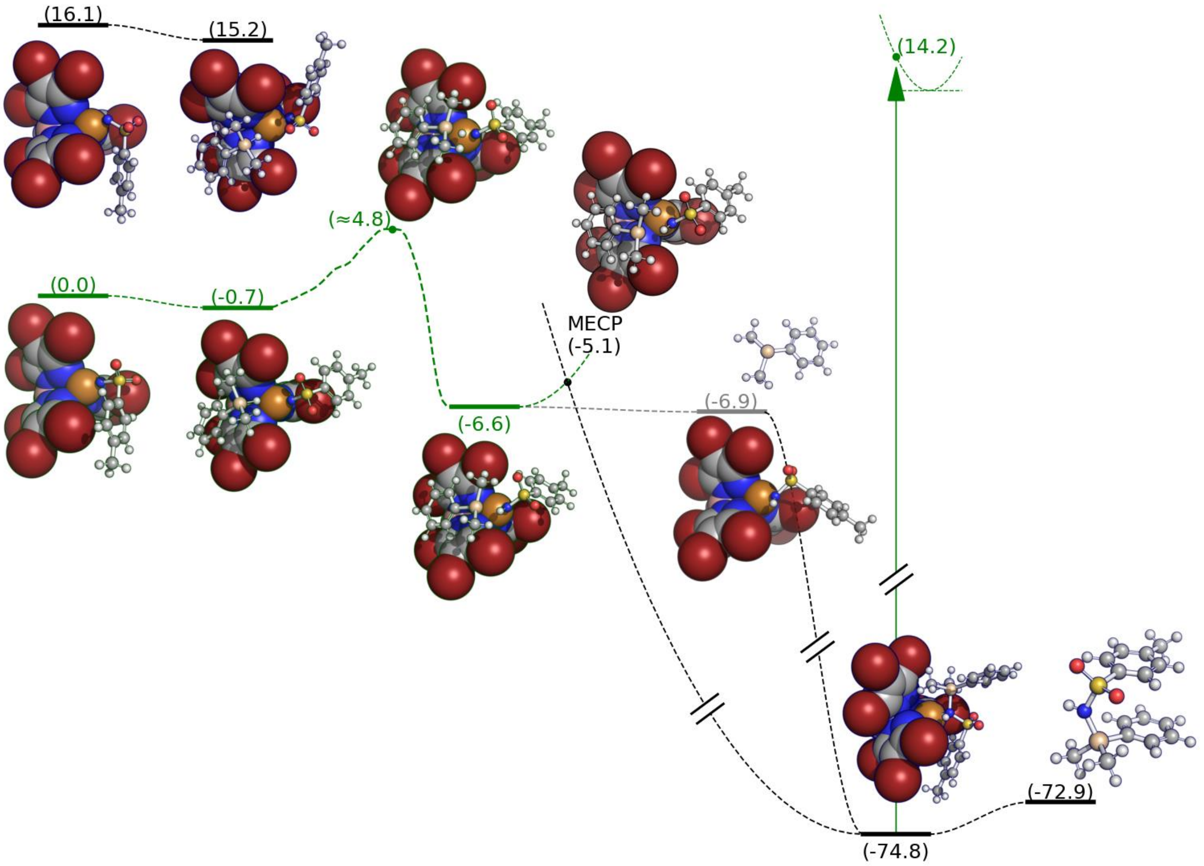

Figure S2: Computed free energy profile of the reaction. Green lines correspond to triplet states, black lines to closed shell singlet states and the grey intermediate corresponds to the two separated radicals in doublet state. Numbers are relative free energies with respect to the initial catalyst in triplet state and the silane. The geometries of each step are represented in vdW spheres for the $Tp^{Br3}Cu$ and in ball and stick for the rest. C atoms are in grey, N atoms in blue, H atoms in white, O atoms in red, Br atoms in dark red, B atom in pink, Si in light orange, S in yellow and Cu in light brown.

Computational study of the hydrogen atom transfer

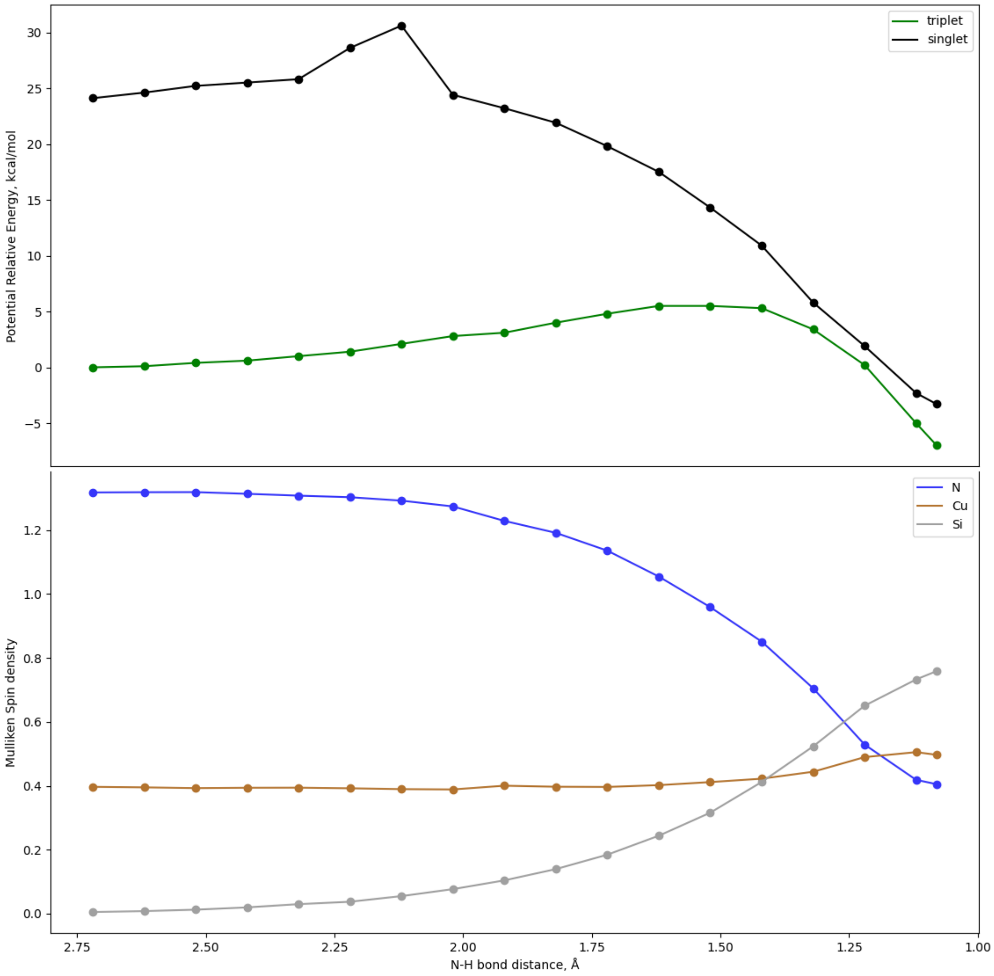

Figure S3: Singlet and triplet potential energies relative to the initial geometry of the scan and Mulliken spin densities of the atoms with higher spin density values in the triplet state as a function of the scan coordinate, the N-H bond distance.

It would have been desirable to compute a transition state for the homolytic cleavage of the Si-H bond but the potential energy surface is too flat for that. A relaxed scan of the H-N distance was carried out from the adduct between $Tp^{Br3}Cu(NTs)$ and $Si(Me)_2(Ph)$. 17 steps where computed, all but the last one with a change of 0.1 A. Each geometry was minimized with the BS1 at the triplet state and for each one of the resulting geometries a SP calculation in triplet and another one in singlet were performed using BS2. The results are shown in Figure S3. These results demonstrate that the hydrogen transfer process does proceed without problems for a reaction at 50ºC.

Cartesian coordinates and potential energies

Me2PhSiH

Energy (POTENTIAL) = -601.630460841 Eh

	Atom	X	Y	Z
1	H	-0.3347	-2.5525	-0.9891
2	Si	-0.4061	-3.8479	-1.7294
3	C	-1.0841	-3.5347	-3.4627
4	C	-1.5203	-5.0552	-0.8033
5	C	1.3567	-4.5173	-1.8112
6	H	-2.1001	-3.1243	-3.4209
7	H	-0.4570	-2.8293	-4.0192
8	H	-1.1257	-4.4684	-4.0365
9	H	-1.1444	-5.2553	0.2061
10	H	-2.5398	-4.6623	-0.7134
11	H	-1.5813	-6.0130	-1.3335
12	C	2.4636	-3.7047	-1.5031
13	C	1.6085	-5.8482	-2.1985
14	C	3.7698	-4.1963	-1.5828
15	H	2.3072	-2.6734	-1.1939
16	C	2.9108	-6.3457	-2.2803
17	H	0.7788	-6.5104	-2.4383
18	C	3.9954	-5.5189	-1.9721
19	H	4.6089	-3.5499	-1.3392
20	H	3.0807	-7.3764	-2.5805
21	H	5.0096	-5.9041	-2.0335

Me2PhSi

Energy (POTENTIAL) = -600.978411362 Eh

	Atom	X	Y	Z
1	Si	-0.4596	-4.1067	-1.5066
2	C	-0.9228	-2.8384	-2.8356
3	C	-1.6562	-5.5760	-1.4940
4	C	1.3165	-4.6860	-1.7102
5	H	-1.9694	-2.5331	-2.7267
6	H	-0.3064	-1.9354	-2.7770
7	H	-0.7994	-3.2659	-3.8400
8	H	-1.4568	-6.2608	-0.6633
9	H	-2.6908	-5.2287	-1.3983
10	H	-1.5765	-6.1470	-2.4289
11	C	2.2928	-3.8626	-2.3120
12	C	1.7307	-5.9510	-1.2390
13	C	3.6157	-4.2862	-2.4468
14	H	2.0165	-2.8802	-2.6879
15	C	3.0530	-6.3769	-1.3716
16	H	1.0103	-6.6164	-0.7688
17	C	4.0008	-5.5455	-1.9766
18	H	4.3464	-3.6360	-2.9210
19	H	3.3449	-7.3583	-1.0066
20	H	5.0307	-5.8760	-2.0806

Me2PhSiH_SP
Energy (POTENTIAL) = -601.769459 Eh

	Atom	X	Y	Z
1	H	-0.3347	-2.5525	-0.9891
2	Si	-0.4061	-3.8479	-1.7294
3	C	-1.0841	-3.5347	-3.4627
4	C	-1.5203	-5.0552	-0.8033
5	C	1.3567	-4.5173	-1.8112
6	H	-2.1001	-3.1243	-3.4209
7	H	-0.4570	-2.8293	-4.0192
8	H	-1.1257	-4.4684	-4.0365
9	H	-1.1444	-5.2553	0.2061
10	H	-2.5398	-4.6623	-0.7134
11	H	-1.5813	-6.0130	-1.3335
12	C	2.4636	-3.7047	-1.5031
13	C	1.6085	-5.8482	-2.1985
14	C	3.7698	-4.1963	-1.5828
15	H	2.3072	-2.6734	-1.1939
16	C	2.9108	-6.3457	-2.2803
17	H	0.7788	-6.5104	-2.4383
18	C	3.9954	-5.5189	-1.9721
19	H	4.6089	-3.5499	-1.3392
20	H	3.0807	-7.3764	-2.5805
21	H	5.0096	-5.9041	-2.0335

Me2PhSi_SP
Energy (POTENTIAL) = -601.1163583 Eh

	Atom	X	Y	Z
1	Si	-0.4596	-4.1067	-1.5066
2	C	-0.9228	-2.8384	-2.8356
3	C	-1.6562	-5.5760	-1.4940
4	C	1.3165	-4.6860	-1.7102
5	H	-1.9694	-2.5331	-2.7267
6	H	-0.3064	-1.9354	-2.7770
7	H	-0.7994	-3.2659	-3.8400
8	H	-1.4568	-6.2608	-0.6633
9	H	-2.6908	-5.2287	-1.3983
10	H	-1.5765	-6.1470	-2.4289
11	C	2.2928	-3.8626	-2.3120
12	C	1.7307	-5.9510	-1.2390
13	C	3.6157	-4.2862	-2.4468
14	H	2.0165	-2.8802	-2.6879
15	C	3.0530	-6.3769	-1.3716
16	H	1.0103	-6.6164	-0.7688
17	C	4.0008	-5.5455	-1.9766
18	H	4.3464	-3.6360	-2.9210
19	H	3.3449	-7.3583	-1.0066
20	H	5.0307	-5.8760	-2.0806

HNSi_s_v1
Energy (POTENTIAL) = -1476.00649053 Eh

	Atom	X	Y	Z
1	S	2.5462	0.7021	1.7580
2	O	1.9101	2.0292	1.7338
3	O	2.5139	-0.1180	2.9795
4	C	4.2651	0.9132	1.2986
5	C	5.2530	0.1949	1.9736
6	C	4.5874	1.7610	0.2368
7	C	6.5791	0.3231	1.5669
8	H	4.9825	-0.4573	2.7955
9	C	5.9188	1.8732	-0.1594
10	H	3.8130	2.3279	-0.2706
11	C	6.9319	1.1531	0.4922
12	H	7.3510	-0.2390	2.0851
13	H	6.1757	2.5293	-0.9866
14	N	1.8471	-0.1925	0.5518
15	C	8.3655	1.2357	0.0325
16	H	8.5320	2.0966	-0.6209
17	H	9.0532	1.3078	0.8814
18	H	8.6403	0.3331	-0.5279
19	H	1.5679	0.3935	-0.2315
20	C	3.8333	-2.3908	0.1383
21	C	4.4840	-3.2288	1.0612
22	C	4.5990	-1.8477	-0.9120
23	C	5.8484	-3.5108	0.9452
24	H	3.9257	-3.6624	1.8871
25	C	5.9607	-2.1244	-1.0332
26	H	4.1324	-1.1862	-1.6388
27	C	6.5887	-2.9577	-0.1018
28	H	6.3321	-4.1577	1.6721
29	H	6.5334	-1.6880	-1.8470
30	H	7.6502	-3.1721	-0.1916
31	C	1.2181	-2.8737	1.7181
32	H	1.7149	-2.6301	2.6614
33	H	0.1649	-2.5874	1.8127
34	H	1.2621	-3.9585	1.5686
35	C	1.1215	-2.2148	-1.3414
36	H	1.6143	-1.6691	-2.1539
37	H	1.1168	-3.2773	-1.6098
38	H	0.0809	-1.8762	-1.2855
39	Si	2.0091	-1.9600	0.2914

HNSi_s_v2
Energy (POTENTIAL) = -1476.00324422 Eh

	Atom	X	Y	Z
1	S	3.5942	1.4780	1.4809
2	O	3.7583	0.5801	2.6371
3	O	4.6719	2.4045	1.0993
4	C	2.1223	2.4596	1.7708
5	C	1.8909	3.5888	0.9817
6	C	1.2136	2.0716	2.7573
7	C	0.7266	4.3278	1.1820
8	H	2.6122	3.8905	0.2291
9	C	0.0548	2.8236	2.9439
10	H	1.4152	1.1992	3.3681
11	C	-0.2082	3.9586	2.1618
12	H	0.5417	5.2070	0.5707
13	H	-0.6551	2.5255	3.7109
14	N	3.2588	0.5341	0.1664
15	C	-1.4519	4.7790	2.3941
16	H	-1.3041	5.4768	3.2279
17	H	-1.7136	5.3707	1.5122
18	H	-2.3059	4.1442	2.6511
19	H	3.4796	1.0113	-0.7041
20	C	3.9422	-2.3913	0.2674
21	C	4.8462	-2.3417	1.3479
22	C	4.0826	-3.4396	-0.6611
23	C	5.8456	-3.3050	1.4956
24	H	4.7704	-1.5380	2.0760
25	C	5.0822	-4.4062	-0.5164
26	H	3.4070	-3.5095	-1.5101
27	C	5.9650	-4.3407	0.5637
28	H	6.5332	-3.2473	2.3354
29	H	5.1721	-5.2065	-1.2462
30	H	6.7432	-5.0906	0.6782
31	C	1.3316	-1.3033	1.5030
32	H	1.8311	-1.2646	2.4747
33	H	0.5710	-0.5162	1.4735
34	H	0.8236	-2.2713	1.4197
35	C	1.7596	-1.2024	-1.5855
36	H	2.4804	-1.0357	-2.3938
37	H	1.2928	-2.1799	-1.7506
38	H	0.9751	-0.4419	-1.6678
39	Si	2.5661	-1.1175	0.1060

HNSi_s_v1_SP
Energy (POTENTIAL) = -1476.4103607 Eh

	Atom	X	Y	Z
1	S	2.5462	0.7021	1.7580
2	O	1.9101	2.0292	1.7338
3	O	2.5139	-0.1180	2.9795
4	C	4.2651	0.9132	1.2986
5	C	5.2530	0.1949	1.9736
6	C	4.5874	1.7610	0.2368
7	C	6.5791	0.3231	1.5669
8	H	4.9825	-0.4573	2.7955
9	C	5.9188	1.8732	-0.1594
10	H	3.8130	2.3279	-0.2706
11	C	6.9319	1.1531	0.4922
12	H	7.3510	-0.2390	2.0851
13	H	6.1757	2.5293	-0.9866
14	N	1.8471	-0.1925	0.5518
15	C	8.3655	1.2357	0.0325
16	H	8.5320	2.0966	-0.6209
17	H	9.0532	1.3078	0.8814
18	H	8.6403	0.3331	-0.5279
19	H	1.5679	0.3935	-0.2315
20	C	3.8333	-2.3908	0.1383
21	C	4.4840	-3.2288	1.0612
22	C	4.5990	-1.8477	-0.9120
23	C	5.8484	-3.5108	0.9452
24	H	3.9257	-3.6624	1.8871
25	C	5.9607	-2.1244	-1.0332
26	H	4.1324	-1.1862	-1.6388
27	C	6.5887	-2.9577	-0.1018
28	H	6.3321	-4.1577	1.6721
29	H	6.5334	-1.6880	-1.8470
30	H	7.6502	-3.1721	-0.1916
31	C	1.2181	-2.8737	1.7181
32	H	1.7149	-2.6301	2.6614
33	H	0.1649	-2.5874	1.8127
34	H	1.2621	-3.9585	1.5686
35	C	1.1215	-2.2148	-1.3414
36	H	1.6143	-1.6691	-2.1539
37	H	1.1168	-3.2773	-1.6098
38	H	0.0809	-1.8762	-1.2855
39	Si	2.0091	-1.9600	0.2914

HNSi_s_v2_SP
Energy (POTENTIAL) = -1476.4070494 Eh

	Atom	X	Y	Z
1	S	3.5942	1.4780	1.4809
2	O	3.7583	0.5801	2.6371
3	O	4.6719	2.4045	1.0993
4	C	2.1223	2.4596	1.7708
5	C	1.8909	3.5888	0.9817
6	C	1.2136	2.0716	2.7573
7	C	0.7266	4.3278	1.1820
8	H	2.6122	3.8905	0.2291
9	C	0.0548	2.8236	2.9439
10	H	1.4152	1.1992	3.3681
11	C	-0.2082	3.9586	2.1618
12	H	0.5417	5.2070	0.5707
13	H	-0.6551	2.5255	3.7109
14	N	3.2588	0.5341	0.1664
15	C	-1.4519	4.7790	2.3941
16	H	-1.3041	5.4768	3.2279
17	H	-1.7136	5.3707	1.5122
18	H	-2.3059	4.1442	2.6511
19	H	3.4796	1.0113	-0.7041
20	C	3.9422	-2.3913	0.2674
21	C	4.8462	-2.3417	1.3479
22	C	4.0826	-3.4396	-0.6611
23	C	5.8456	-3.3050	1.4956
24	H	4.7704	-1.5380	2.0760
25	C	5.0822	-4.4062	-0.5164
26	H	3.4070	-3.5095	-1.5101
27	C	5.9650	-4.3407	0.5637
28	H	6.5332	-3.2473	2.3354
29	H	5.1721	-5.2065	-1.2462
30	H	6.7432	-5.0906	0.6782
31	C	1.3316	-1.3033	1.5030
32	H	1.8311	-1.2646	2.4747
33	H	0.5710	-0.5162	1.4735
34	H	0.8236	-2.2713	1.4197
35	C	1.7596	-1.2024	-1.5855
36	H	2.4804	-1.0357	-2.3938
37	H	1.2928	-2.1799	-1.7506
38	H	0.9751	-0.4419	-1.6678
39	Si	2.5661	-1.1175	0.1060

TpBr3Cu_v1
Energy (POTENTIAL) = -1011.75573636 Eh

	Atom	X	Y	Z
1	Cu	-0.5504	-0.5701	-1.7113
2	N	1.2824	-1.3390	-2.4837
3	N	2.3635	-0.8222	-1.8263
4	N	0.2030	1.4339	-1.8352
5	N	1.4761	1.5160	-1.3419
6	N	0.1196	-0.7953	0.2897
7	N	1.3783	-0.2910	0.4625
8	B	2.1918	0.2743	-0.7363
9	C	1.7480	-2.2292	-3.3529
10	C	3.1514	-2.3187	-3.2851
11	C	3.4969	-1.4012	-2.2963
12	C	-0.1120	2.6418	-2.2911
13	C	0.9509	3.5470	-2.1086
14	C	1.9414	2.7810	-1.5004
15	C	-0.2906	-1.2264	1.4774
16	C	0.6923	-1.0130	2.4621
17	C	1.7379	-0.4131	1.7652
18	H	3.2597	0.5994	-0.3507
19	Br	-1.9914	-2.0144	1.6890
20	Br	-1.7987	2.9883	-3.0684
21	Br	0.5772	-3.1835	-4.4849
22	Br	4.2991	-3.4206	-4.2946
23	Br	5.2252	-0.9904	-1.6700
24	Br	3.3929	0.1601	2.4577
25	Br	0.6160	-1.4422	4.2952
26	Br	1.0203	5.3701	-2.5802
27	Br	3.6587	3.3456	-0.9690

TpBr3Cu_v2
Energy (POTENTIAL) = -1011.75573165 Eh

	Atom	X	Y	Z
1	Cu	-0.6046	-0.5192	-1.5718
2	N	1.1139	-1.4134	-2.4768
3	N	2.2630	-0.9135	-1.9293
4	N	0.1949	1.4335	-1.8189
5	N	1.5094	1.4763	-1.4448
6	N	0.2169	-0.7433	0.3733
7	N	1.4994	-0.2712	0.4195
8	B	2.2258	0.2217	-0.8651
9	C	1.4762	-2.3471	-3.3506
10	C	2.8773	-2.4822	-3.3960
11	C	3.3339	-1.5463	-2.4718
12	C	-0.1163	2.6411	-2.2787
13	C	0.9922	3.5070	-2.2182
14	C	2.0047	2.7177	-1.6792
15	C	-0.1094	-1.0992	1.6115
16	C	0.9546	-0.8641	2.5031
17	C	1.9576	-0.3352	1.6951
18	H	3.3342	0.5152	-0.5835
19	Br	-1.8076	-1.8258	2.0066
20	Br	-1.8542	3.0348	-2.9066
21	Br	0.1925	-3.3080	-4.3532
22	Br	3.9007	-3.6546	-4.4587
23	Br	5.1214	-1.1773	-2.0044
24	Br	3.6781	0.2213	2.2238
25	Br	1.0111	-1.1877	4.3583
26	Br	1.0862	5.3143	-2.7430
27	Br	3.7804	3.2286	-1.3120

TpBr3Cu_v1_SP
Energy (POTENTIAL) = -25505.5696242 Eh

	Atom	X	Y	Z
1	Cu	-0.5504	-0.5701	-1.7113
2	N	1.2824	-1.3390	-2.4837
3	N	2.3635	-0.8222	-1.8263
4	N	0.2030	1.4339	-1.8352
5	N	1.4761	1.5160	-1.3419
6	N	0.1196	-0.7953	0.2897
7	N	1.3783	-0.2910	0.4625
8	B	2.1918	0.2743	-0.7363
9	C	1.7480	-2.2292	-3.3529
10	C	3.1514	-2.3187	-3.2851
11	C	3.4969	-1.4012	-2.2963
12	C	-0.1120	2.6418	-2.2911
13	C	0.9509	3.5470	-2.1086
14	C	1.9414	2.7810	-1.5004
15	C	-0.2906	-1.2264	1.4774
16	C	0.6923	-1.0130	2.4621
17	C	1.7379	-0.4131	1.7652
18	H	3.2597	0.5994	-0.3507
19	Br	-1.9914	-2.0144	1.6890
20	Br	-1.7987	2.9883	-3.0684
21	Br	0.5772	-3.1835	-4.4849
22	Br	4.2991	-3.4206	-4.2946
23	Br	5.2252	-0.9904	-1.6700
24	Br	3.3929	0.1601	2.4577
25	Br	0.6160	-1.4422	4.2952
26	Br	1.0203	5.3701	-2.5802
27	Br	3.6587	3.3456	-0.9690

TpBr3Cu_v2_SP
Energy (POTENTIAL) = -25505.5696124 Eh

	Atom	X	Y	Z
1	Cu	-0.6046	-0.5192	-1.5718
2	N	1.1139	-1.4134	-2.4768
3	N	2.2630	-0.9135	-1.9293
4	N	0.1949	1.4335	-1.8189
5	N	1.5094	1.4763	-1.4448
6	N	0.2169	-0.7433	0.3733
7	N	1.4994	-0.2712	0.4195
8	B	2.2258	0.2217	-0.8651
9	C	1.4762	-2.3471	-3.3506
10	C	2.8773	-2.4822	-3.3960
11	C	3.3339	-1.5463	-2.4718
12	C	-0.1163	2.6411	-2.2787
13	C	0.9922	3.5070	-2.2182
14	C	2.0047	2.7177	-1.6792
15	C	-0.1094	-1.0992	1.6115
16	C	0.9546	-0.8641	2.5031
17	C	1.9576	-0.3352	1.6951
18	H	3.3342	0.5152	-0.5835
19	Br	-1.8076	-1.8258	2.0066
20	Br	-1.8542	3.0348	-2.9066
21	Br	0.1925	-3.3080	-4.3532
22	Br	3.9007	-3.6546	-4.4587
23	Br	5.1214	-1.1773	-2.0044
24	Br	3.6781	0.2213	2.2238
25	Br	1.0111	-1.1877	4.3583
26	Br	1.0862	5.3143	-2.7430
27	Br	3.7804	3.2286	-1.3120

TpBr3CuN_s_v2
Energy (POTENTIAL) = -1885.97907013 Eh

	Atom	X	Y	Z
1	Cu	-0.5447	-0.5278	-1.9950
2	N	1.2726	-1.3875	-2.4135
3	N	2.3908	-0.9103	-1.7977
4	N	0.3083	1.3375	-1.8797
5	N	1.5764	1.4548	-1.3971
6	N	0.1537	-0.6698	0.2472
7	N	1.4378	-0.2530	0.4483
8	B	2.2865	0.2295	-0.7462
9	C	1.6611	-2.3165	-3.2868
10	C	3.0584	-2.4722	-3.2486
11	C	3.4770	-1.5536	-2.2865
12	C	-0.0817	2.5432	-2.2946
13	C	0.9425	3.4843	-2.0857
14	C	1.9758	2.7435	-1.5116
15	C	-0.3375	-0.9807	1.4414
16	C	0.6211	-0.7753	2.4551
17	C	1.7379	-0.3076	1.7699
18	H	3.3680	0.5414	-0.3875
19	N	-2.1787	-0.6365	-2.7598
20	S	-3.7783	-0.7837	-2.7654
21	O	-3.7767	-2.0619	-3.5367
22	O	-4.4300	0.4187	-3.3018
23	C	-4.5014	-1.1084	-1.1725
24	C	-4.6171	-2.4260	-0.7261
25	C	-4.9399	-0.0314	-0.3936
26	C	-5.1922	-2.6637	0.5222
27	H	-4.2758	-3.2485	-1.3447
28	C	-5.4984	-0.2900	0.8530
29	H	-4.8552	0.9854	-0.7617
30	C	-5.6375	-1.6055	1.3298
31	H	-5.2974	-3.6866	0.8721
32	H	-5.8400	0.5388	1.4666
33	C	-6.2717	-1.8612	2.6724
34	H	-5.8453	-1.2067	3.4401
35	H	-7.3482	-1.6541	2.6352
36	H	-6.1400	-2.8990	2.9899
37	Br	-2.1108	-1.5907	1.6232
38	Br	-1.7916	2.8080	-3.0278
39	Br	0.4210	-3.2046	-4.3911
40	Br	4.1195	-3.6465	-4.2664
41	Br	5.2347	-1.2142	-1.7102

42	Br	3.4087	0.1881	2.4844
43	Br	0.4354	-1.0641	4.3072
44	Br	0.9220	5.3218	-2.4917
45	Br	3.6634	3.3621	-0.9588

TpBr3CuN_s_v1
Energy (POTENTIAL) = -1885.98472388 Eh

	Atom	X	Y	Z
1	N	2.4131	0.6485	-0.3884
2	N	1.5703	1.7116	-0.5284
3	N	1.1953	-0.5008	1.4996
4	N	0.1456	0.3586	1.6540
5	N	0.8034	-1.2245	-0.9007
6	N	-0.2950	-0.4712	-1.1912
7	C	2.2988	2.7228	-0.9809
8	C	3.6468	2.3432	-1.1493
9	C	3.6682	1.0078	-0.7573
10	C	-0.0975	0.4558	2.9633
11	C	0.7891	-0.3582	3.6910
12	C	1.5942	-0.9428	2.7158
13	C	-0.9337	-1.0685	-2.1978
14	C	-0.2623	-2.2455	-2.5765
15	C	0.8393	-2.2987	-1.7243
16	B	1.8619	-0.7085	0.1110
17	H	2.7369	-1.4961	0.2047
18	N	-1.8545	2.5303	-0.4270
19	S	-3.4676	2.6015	-0.3227
20	O	-3.4296	3.3093	0.9953
21	O	-4.1020	3.2469	-1.4774
22	C	-4.2059	0.9938	-0.1271
23	C	-5.3258	0.6620	-0.8926
24	C	-3.6440	0.0751	0.7684
25	C	-5.8765	-0.6120	-0.7637
26	H	-5.7478	1.3820	-1.5844
27	C	-4.2011	-1.1926	0.8759
28	H	-2.7814	0.3416	1.3663
29	C	-5.3236	-1.5587	0.1114
30	H	-6.7462	-0.8764	-1.3582
31	H	-3.7619	-1.9106	1.5630
32	Cu	-0.6517	1.2204	-0.0492
33	C	-5.9206	-2.9337	0.2524
34	H	-6.4517	-3.0257	1.2079
35	H	-6.6318	-3.1490	-0.5493
36	H	-5.1420	-3.7038	0.2430
37	Br	-1.4443	1.5786	3.6531
38	Br	0.8704	-0.6043	5.5545
39	Br	3.0112	-2.1514	2.9765
40	Br	1.5126	4.4033	-1.3155
41	Br	5.0858	3.3950	-1.7583

42	Br	5.1437	-0.1619	-0.7176
43	Br	-2.4906	-0.3364	-2.9545
44	Br	-0.7430	-3.4689	-3.9232
45	Br	2.1889	-3.6073	-1.6723

TpBr3CuN_s_v2_SP
Energy (POTENTIAL) = -26380.063254 Eh

	Atom	X	Y	Z
1	Cu	-0.5447	-0.5278	-1.9950
2	N	1.2726	-1.3875	-2.4135
3	N	2.3908	-0.9103	-1.7977
4	N	0.3083	1.3375	-1.8797
5	N	1.5764	1.4548	-1.3971
6	N	0.1537	-0.6698	0.2472
7	N	1.4378	-0.2530	0.4483
8	B	2.2865	0.2295	-0.7462
9	C	1.6611	-2.3165	-3.2868
10	C	3.0584	-2.4722	-3.2486
11	C	3.4770	-1.5536	-2.2865
12	C	-0.0817	2.5432	-2.2946
13	C	0.9425	3.4843	-2.0857
14	C	1.9758	2.7435	-1.5116
15	C	-0.3375	-0.9807	1.4414
16	C	0.6211	-0.7753	2.4551
17	C	1.7379	-0.3076	1.7699
18	H	3.3680	0.5414	-0.3875
19	N	-2.1787	-0.6365	-2.7598
20	S	-3.7783	-0.7837	-2.7654
21	O	-3.7767	-2.0619	-3.5367
22	O	-4.4300	0.4187	-3.3018
23	C	-4.5014	-1.1084	-1.1725
24	C	-4.6171	-2.4260	-0.7261
25	C	-4.9399	-0.0314	-0.3936
26	C	-5.1922	-2.6637	0.5222
27	H	-4.2758	-3.2485	-1.3447
28	C	-5.4984	-0.2900	0.8530
29	H	-4.8552	0.9854	-0.7617
30	C	-5.6375	-1.6055	1.3298
31	H	-5.2974	-3.6866	0.8721
32	H	-5.8400	0.5388	1.4666
33	C	-6.2717	-1.8612	2.6724
34	H	-5.8453	-1.2067	3.4401
35	H	-7.3482	-1.6541	2.6352
36	H	-6.1400	-2.8990	2.9899
37	Br	-2.1108	-1.5907	1.6232
38	Br	-1.7916	2.8080	-3.0278
39	Br	0.4210	-3.2046	-4.3911
40	Br	4.1195	-3.6465	-4.2664
41	Br	5.2347	-1.2142	-1.7102

42	Br	3.4087	0.1881	2.4844
43	Br	0.4354	-1.0641	4.3072
44	Br	0.9220	5.3218	-2.4917
45	Br	3.6634	3.3621	-0.9588

TpBr3CuN_s_v1_SP
Energy (POTENTIAL) = -26380.0667085 Eh

	Atom	X	Y	Z
1	N	2.4131	0.6485	-0.3884
2	N	1.5703	1.7116	-0.5284
3	N	1.1953	-0.5008	1.4996
4	N	0.1456	0.3586	1.6540
5	N	0.8034	-1.2244	-0.9007
6	N	-0.2950	-0.4712	-1.1912
7	C	2.2988	2.7228	-0.9809
8	C	3.6468	2.3432	-1.1493
9	C	3.6682	1.0078	-0.7573
10	C	-0.0975	0.4558	2.9633
11	C	0.7891	-0.3582	3.6910
12	C	1.5942	-0.9428	2.7158
13	C	-0.9337	-1.0685	-2.1978
14	C	-0.2623	-2.2455	-2.5765
15	C	0.8393	-2.2987	-1.7243
16	B	1.8619	-0.7085	0.1110
17	H	2.7369	-1.4961	0.2047
18	N	-1.8545	2.5303	-0.4270
19	S	-3.4676	2.6015	-0.3228
20	O	-3.4296	3.3093	0.9953
21	O	-4.1020	3.2469	-1.4774
22	C	-4.2059	0.9938	-0.1271
23	C	-5.3258	0.6620	-0.8926
24	C	-3.6440	0.0751	0.7684
25	C	-5.8765	-0.6120	-0.7637
26	H	-5.7478	1.3820	-1.5844
27	C	-4.2011	-1.1926	0.8759
28	H	-2.7814	0.3416	1.3663
29	C	-5.3236	-1.5587	0.1114
30	H	-6.7462	-0.8764	-1.3582
31	H	-3.7619	-1.9106	1.5630
32	Cu	-0.6517	1.2204	-0.0492
33	C	-5.9206	-2.9337	0.2524
34	H	-6.4517	-3.0257	1.2079
35	H	-6.6318	-3.1490	-0.5493
36	H	-5.1420	-3.7038	0.2430
37	Br	-1.4443	1.5786	3.6531
38	Br	0.8704	-0.6043	5.5545
39	Br	3.0112	-2.1514	2.9765
40	Br	1.5126	4.4033	-1.3155
41	Br	5.0858	3.3950	-1.7583

42	Br	5.1436	-0.1619	-0.7176
43	Br	-2.4906	-0.3364	-2.9545
44	Br	-0.7430	-3.4689	-3.9232
45	Br	2.1889	-3.6073	-1.6723

TpBr3CuN_t_v2
Energy (POTENTIAL) = -1886.00803414 Eh

	Atom	X	Y	Z
1	Cu	-0.5732	-0.5541	-1.7417
2	N	1.2949	-1.3152	-2.4019
3	N	2.4176	-0.8011	-1.8257
4	N	0.3044	1.4023	-1.8177
5	N	1.5688	1.5301	-1.3259
6	N	0.1756	-0.7107	0.2347
7	N	1.4503	-0.2710	0.4461
8	B	2.2957	0.2935	-0.7306
9	C	1.6854	-2.2254	-3.2890
10	C	3.0895	-2.3257	-3.3124
11	C	3.5099	-1.3965	-2.3618
12	C	-0.0808	2.6024	-2.2406
13	C	0.9385	3.5519	-2.0338
14	C	1.9692	2.8189	-1.4473
15	C	-0.2947	-1.1404	1.4030
16	C	0.6735	-0.9883	2.4131
17	C	1.7672	-0.4308	1.7534
18	H	3.3695	0.6012	-0.3456
19	N	-2.2211	-0.9213	-2.3947
20	S	-3.8183	-1.0911	-2.8300
21	O	-3.9383	-2.3535	-3.5746
22	O	-4.2359	0.1718	-3.4575
23	C	-4.5752	-1.2555	-1.2372
24	C	-4.7850	-2.5329	-0.7083
25	C	-4.8871	-0.0978	-0.5103
26	C	-5.3324	-2.6461	0.5675
27	H	-4.5309	-3.4146	-1.2865
28	C	-5.4309	-0.2345	0.7601
29	H	-4.7134	0.8834	-0.9388
30	C	-5.6624	-1.5051	1.3193
31	H	-5.5106	-3.6326	0.9854
32	H	-5.6816	0.6541	1.3321
33	C	-6.2739	-1.6286	2.6893
34	H	-5.8085	-0.9313	3.3937
35	H	-7.3426	-1.3831	2.6526
36	H	-6.1765	-2.6429	3.0850
37	Br	-2.0431	-1.8261	1.5469
38	Br	-1.7917	2.8506	-2.9902
39	Br	0.4236	-3.1830	-4.3095
40	Br	4.1534	-3.4552	-4.3780
41	Br	5.2740	-0.9836	-1.8536

42	Br	3.4326	0.0452	2.4892
43	Br	0.5254	-1.4361	4.2356
44	Br	0.9157	5.3870	-2.4543
45	Br	3.6560	3.4411	-0.8908

TpBr3CuN_t_v3
Energy (POTENTIAL) = -1886.00696267 Eh

	Atom	X	Y	Z
1	Cu	-0.5471	-0.4005	-2.1887
2	N	1.3229	-1.1670	-2.7545
3	N	2.3934	-0.7539	-2.0134
4	N	0.3800	1.4908	-1.9182
5	N	1.5481	1.4897	-1.2126
6	N	0.0254	-0.9808	-0.1522
7	N	1.2671	-0.5743	0.2380
8	B	2.1972	0.1545	-0.7658
9	C	1.7920	-1.9766	-3.7033
10	C	3.1891	-2.1043	-3.6039
11	C	3.5244	-1.3068	-2.5116
12	C	0.0795	2.7600	-2.1853
13	C	1.0540	3.6224	-1.6488
14	C	1.9677	2.7657	-1.0388
15	C	-0.5279	-1.6019	0.8840
16	C	0.3479	-1.6115	1.9871
17	C	1.4798	-0.9439	1.5244
18	H	3.2433	0.3685	-0.2608
19	N	-2.3729	-0.3841	-2.4252
20	S	-3.7422	0.4452	-1.9529
21	O	-4.2194	1.1286	-3.1702
22	O	-3.4741	1.2344	-0.7402
23	C	-4.8280	-0.8988	-1.5433
24	C	-5.1932	-1.8097	-2.5434
25	C	-5.2794	-1.0385	-0.2278
26	C	-6.0153	-2.8793	-2.2061
27	H	-4.8381	-1.6864	-3.5613
28	C	-6.1086	-2.1123	0.0861
29	H	-4.9815	-0.3212	0.5283
30	C	-6.4846	-3.0487	-0.8915
31	H	-6.3009	-3.5947	-2.9719
32	H	-6.4699	-2.2267	1.1042
33	C	-7.4022	-4.1926	-0.5503
34	H	-7.3616	-4.4370	0.5149
35	H	-8.4407	-3.9301	-0.7895
36	H	-7.1534	-5.0893	-1.1258
37	Br	-2.2619	-2.3252	0.7593
38	Br	-1.4587	3.2062	-3.1668
39	Br	0.6524	-2.8101	-4.9528
40	Br	4.3414	-3.1185	-4.6929
41	Br	5.2381	-1.0029	-1.7979

42	Br	3.0660	-0.5729	2.4668
43	Br	0.0593	-2.3579	3.6915
44	Br	1.1119	5.5017	-1.7367
45	Br	3.5364	3.2305	-0.1096

TpBr3CuN_t_v1
Energy (POTENTIAL) = -1886.00788815 Eh

	Atom	X	Y	Z
1	N	2.4885	0.7803	-0.6453
2	N	1.4168	1.6199	-0.7036
3	N	1.9208	-0.3963	1.5211
4	N	0.7396	0.2206	1.8100
5	N	1.2517	-1.4215	-0.6912
6	N	-0.0217	-0.9397	-0.7761
7	C	1.8053	2.7146	-1.3515
8	C	3.1562	2.6120	-1.7337
9	C	3.5467	1.3601	-1.2603
10	C	0.6281	0.2634	3.1347
11	C	1.7484	-0.3304	3.7465
12	C	2.5441	-0.7339	2.6756
13	C	-0.7429	-1.8394	-1.4393
14	C	0.0543	-2.9401	-1.8070
15	C	1.3170	-2.6259	-1.3082
16	B	2.3509	-0.6055	0.0429
17	H	3.3811	-1.1825	-0.0004
18	N	-1.9671	1.8323	0.2721
19	S	-3.4773	2.4548	0.5959
20	O	-3.5267	2.7312	2.0400
21	O	-3.7278	3.5383	-0.3657
22	C	-4.4920	1.0559	0.2102
23	C	-5.0525	0.9460	-1.0668
24	C	-4.6499	0.0482	1.1719
25	C	-5.7912	-0.1933	-1.3755
26	H	-4.9131	1.7360	-1.7967
27	C	-5.3891	-1.0803	0.8415
28	H	-4.2045	0.1532	2.1553
29	C	-5.9674	-1.2222	-0.4331
30	H	-6.2389	-0.2878	-2.3607
31	H	-5.5189	-1.8682	1.5776
32	Cu	-0.3943	0.9459	0.1624
33	C	-6.7724	-2.4482	-0.7713
34	H	-7.7963	-2.3508	-0.3884
35	H	-6.8365	-2.6020	-1.8520
36	H	-6.3411	-3.3442	-0.3146
37	Br	-0.8796	1.0473	3.9473
38	Br	2.0936	-0.5343	5.5858
39	Br	4.2146	-1.5973	2.7438
40	Br	0.6074	4.1377	-1.6538
41	Br	4.1965	3.8727	-2.6674

42	Br	5.2360	0.5457	-1.4112
43	Br	-2.5789	-1.5583	-1.7571
44	Br	-0.4653	-4.4830	-2.7527
45	Br	2.8912	-3.6504	-1.4316

TpBr3CuN_t_v2_SP
Energy (POTENTIAL) = -26380.0898912 Eh

	Atom	X	Y	Z
1	Cu	-0.5732	-0.5541	-1.7417
2	N	1.2949	-1.3152	-2.4019
3	N	2.4176	-0.8011	-1.8257
4	N	0.3044	1.4023	-1.8177
5	N	1.5688	1.5301	-1.3259
6	N	0.1756	-0.7107	0.2347
7	N	1.4503	-0.2710	0.4461
8	B	2.2957	0.2935	-0.7306
9	C	1.6854	-2.2254	-3.2890
10	C	3.0895	-2.3257	-3.3124
11	C	3.5099	-1.3965	-2.3618
12	C	-0.0808	2.6024	-2.2406
13	C	0.9386	3.5519	-2.0338
14	C	1.9692	2.8189	-1.4473
15	C	-0.2947	-1.1404	1.4030
16	C	0.6735	-0.9883	2.4131
17	C	1.7672	-0.4308	1.7534
18	H	3.3695	0.6012	-0.3456
19	N	-2.2211	-0.9213	-2.3947
20	S	-3.8183	-1.0911	-2.8300
21	O	-3.9383	-2.3535	-3.5746
22	O	-4.2359	0.1718	-3.4575
23	C	-4.5752	-1.2555	-1.2372
24	C	-4.7850	-2.5329	-0.7083
25	C	-4.8871	-0.0978	-0.5103
26	C	-5.3324	-2.6461	0.5675
27	H	-4.5309	-3.4146	-1.2865
28	C	-5.4309	-0.2345	0.7601
29	H	-4.7134	0.8834	-0.9388
30	C	-5.6624	-1.5051	1.3193
31	H	-5.5106	-3.6326	0.9854
32	H	-5.6816	0.6541	1.3321
33	C	-6.2739	-1.6286	2.6893
34	H	-5.8085	-0.9313	3.3937
35	H	-7.3426	-1.3831	2.6526
36	H	-6.1765	-2.6429	3.0850
37	Br	-2.0431	-1.8261	1.5469
38	Br	-1.7917	2.8506	-2.9902
39	Br	0.4236	-3.1830	-4.3095
40	Br	4.1534	-3.4552	-4.3780
41	Br	5.2740	-0.9836	-1.8536

42	Br	3.4326	0.0452	2.4892
43	Br	0.5254	-1.4361	4.2356
44	Br	0.9157	5.3870	-2.4543
45	Br	3.6560	3.4411	-0.8908

TpBr3CuN_t_v3_SP
Energy (POTENTIAL) = -26380.0883161 Eh

	Atom	X	Y	Z
1	Cu	-0.5471	-0.4005	-2.1887
2	N	1.3229	-1.1670	-2.7545
3	N	2.3934	-0.7539	-2.0134
4	N	0.3800	1.4908	-1.9182
5	N	1.5481	1.4897	-1.2126
6	N	0.0254	-0.9808	-0.1522
7	N	1.2671	-0.5743	0.2380
8	B	2.1972	0.1545	-0.7658
9	C	1.7920	-1.9766	-3.7033
10	C	3.1891	-2.1043	-3.6039
11	C	3.5244	-1.3068	-2.5116
12	C	0.0795	2.7600	-2.1853
13	C	1.0540	3.6224	-1.6488
14	C	1.9677	2.7657	-1.0388
15	C	-0.5279	-1.6019	0.8840
16	C	0.3479	-1.6115	1.9871
17	C	1.4798	-0.9439	1.5244
18	H	3.2433	0.3685	-0.2608
19	N	-2.3729	-0.3841	-2.4252
20	S	-3.7422	0.4452	-1.9529
21	O	-4.2194	1.1286	-3.1702
22	O	-3.4741	1.2344	-0.7402
23	C	-4.8280	-0.8988	-1.5433
24	C	-5.1932	-1.8097	-2.5434
25	C	-5.2794	-1.0385	-0.2278
26	C	-6.0153	-2.8793	-2.2061
27	H	-4.8381	-1.6864	-3.5613
28	C	-6.1086	-2.1123	0.0861
29	H	-4.9815	-0.3212	0.5283
30	C	-6.4846	-3.0487	-0.8915
31	H	-6.3009	-3.5947	-2.9719
32	H	-6.4699	-2.2267	1.1042
33	C	-7.4022	-4.1926	-0.5503
34	H	-7.3616	-4.4370	0.5149
35	H	-8.4407	-3.9301	-0.7895
36	H	-7.1534	-5.0893	-1.1258
37	Br	-2.2619	-2.3252	0.7593
38	Br	-1.4587	3.2062	-3.1668
39	Br	0.6524	-2.8101	-4.9528
40	Br	4.3414	-3.1185	-4.6929
41	Br	5.2381	-1.0029	-1.7979

42	Br	3.0660	-0.5729	2.4668
43	Br	0.0593	-2.3579	3.6915
44	Br	1.1119	5.5017	-1.7367
45	Br	3.5364	3.2305	-0.1096

TpBr3CuN_t_v1_SP
Energy (POTENTIAL) = -26380.0897482 Eh

	Atom	X	Y	Z
1	N	2.4885	0.7803	-0.6453
2	N	1.4168	1.6199	-0.7036
3	N	1.9208	-0.3963	1.5211
4	N	0.7396	0.2206	1.8100
5	N	1.2517	-1.4215	-0.6912
6	N	-0.0217	-0.9397	-0.7761
7	C	1.8053	2.7146	-1.3515
8	C	3.1562	2.6120	-1.7337
9	C	3.5467	1.3601	-1.2603
10	C	0.6281	0.2634	3.1347
11	C	1.7484	-0.3304	3.7465
12	C	2.5441	-0.7339	2.6756
13	C	-0.7429	-1.8394	-1.4393
14	C	0.0543	-2.9401	-1.8070
15	C	1.3170	-2.6259	-1.3082
16	B	2.3509	-0.6055	0.0429
17	H	3.3811	-1.1825	-0.0004
18	N	-1.9671	1.8323	0.2721
19	S	-3.4773	2.4548	0.5959
20	O	-3.5267	2.7312	2.0400
21	O	-3.7278	3.5383	-0.3657
22	C	-4.4920	1.0559	0.2102
23	C	-5.0525	0.9460	-1.0668
24	C	-4.6499	0.0482	1.1719
25	C	-5.7912	-0.1933	-1.3755
26	H	-4.9131	1.7360	-1.7967
27	C	-5.3891	-1.0803	0.8415
28	H	-4.2045	0.1532	2.1553
29	C	-5.9674	-1.2222	-0.4331
30	H	-6.2389	-0.2878	-2.3607
31	H	-5.5189	-1.8682	1.5776
32	Cu	-0.3943	0.9459	0.1624
33	C	-6.7724	-2.4482	-0.7713
34	H	-7.7963	-2.3508	-0.3884
35	H	-6.8365	-2.6020	-1.8520
36	H	-6.3411	-3.3442	-0.3146
37	Br	-0.8796	1.0473	3.9473
38	Br	2.0936	-0.5343	5.5858
39	Br	4.2146	-1.5973	2.7438
40	Br	0.6074	4.1377	-1.6538
41	Br	4.1965	3.8727	-2.6674

42	Br	5.2360	0.5457	-1.4112
43	Br	-2.5789	-1.5583	-1.7571
44	Br	-0.4653	-4.4830	-2.7527
45	Br	2.8912	-3.6504	-1.4316

TpBr3CuNH_v2

Energy (POTENTIAL) = -1886.67657605 Eh

	Atom	X	Y	Z
1	N	2.0270	-0.1229	0.2559
2	N	1.6762	1.2003	0.2326
3	N	-0.1539	-1.1429	0.9712
4	N	-0.7163	0.0765	1.2196
5	N	0.4786	-1.0450	-1.5195
6	N	0.1199	0.1896	-1.9724
7	C	2.7179	1.8860	0.7000
8	C	3.7786	1.0228	1.0346
9	C	3.2884	-0.2452	0.7365
10	C	-1.3755	-0.0304	2.3653
11	C	-1.2857	-1.3409	2.8833
12	C	-0.4890	-2.0106	1.9616
13	C	-0.1296	0.0809	-3.2782
14	C	0.0439	-1.2493	-3.7006
15	C	0.4334	-1.9268	-2.5453
16	B	0.9916	-1.2423	-0.0667
17	H	1.5106	-2.3003	0.0155
18	N	-1.7483	2.7582	-0.9250
19	S	-3.3865	2.4593	-1.1724
20	O	-4.1499	3.2619	-0.2001
21	O	-3.6793	2.6342	-2.6078
22	C	-3.5475	0.7334	-0.7585
23	C	-3.0790	-0.2280	-1.6601
24	C	-4.1185	0.3669	0.4571
25	C	-3.1148	-1.5683	-1.2958
26	H	-2.6968	0.0721	-2.6272
27	C	-4.1597	-0.9846	0.8011
28	H	-4.5057	1.1263	1.1249
29	C	-3.6364	-1.9655	-0.0516
30	H	-2.7368	-2.3190	-1.9843
31	H	-4.5911	-1.2777	1.7536
32	Cu	-0.3015	1.5895	-0.5214
33	C	-3.6335	-3.4195	0.3420
34	H	-3.9526	-3.5567	1.3785
35	H	-4.3053	-4.0014	-0.3002
36	H	-2.6328	-3.8511	0.2325
37	Br	4.1762	-1.8914	0.9445
38	Br	5.4691	1.4766	1.7270
39	Br	2.6914	3.7643	0.8876
40	Br	-0.1976	-1.9554	-5.4272
41	Br	-0.6575	1.5614	-4.3099

42	Br	0.8329	-3.7548	-2.3664
43	Br	0.0572	-3.8114	1.9993
44	Br	-2.0731	-2.0305	4.4484
45	Br	-2.2240	1.4555	3.1593
46	H	-1.5336	3.5824	-1.4982

TpBr3CuNH_v3
Energy (POTENTIAL) = -1886.67273111 Eh

	Atom	X	Y	Z
1	N	2.4525	0.5003	-0.6562
2	N	1.2862	1.1945	-0.4858
3	N	2.3291	-0.9204	1.4288
4	N	1.1280	-0.4746	1.9016
5	N	1.6735	-1.9041	-0.8163
6	N	0.3341	-1.6560	-0.8063
7	C	1.4685	2.4028	-1.0249
8	C	2.7591	2.5146	-1.5698
9	C	3.3462	1.2789	-1.3096
10	C	1.1455	-0.6133	3.2276
11	C	2.3722	-1.1598	3.6483
12	C	3.0903	-1.3383	2.4682
13	C	-0.2414	-2.6292	-1.5025
14	C	0.7171	-3.5441	-1.9847
15	C	1.9268	-3.0358	-1.5194
16	B	2.6374	-0.9370	-0.0931
17	H	3.7537	-1.2823	-0.2628
18	N	-2.0195	-0.3424	0.8757
19	S	-3.1794	0.4972	1.7342
20	O	-3.4794	-0.1435	3.0285
21	O	-2.7831	1.9152	1.7100
22	C	-4.5887	0.2533	0.6633
23	C	-4.7353	1.0599	-0.4703
24	C	-5.4836	-0.7852	0.9353
25	C	-5.8021	0.8227	-1.3319
26	H	-4.0283	1.8593	-0.6663
27	C	-6.5434	-1.0090	0.0591
28	H	-5.3541	-1.3977	1.8212
29	C	-6.7201	-0.2125	-1.0836
30	H	-5.9276	1.4477	-2.2118
31	H	-7.2462	-1.8115	0.2658
32	Cu	-0.2589	0.2077	0.4664
33	Br	0.1428	3.7392	-0.9892
34	Br	3.5236	3.9956	-2.4425
35	Br	5.0825	0.7230	-1.7709
36	Br	0.4351	-5.0946	-3.0158
37	Br	3.6529	-3.7405	-1.7813
38	Br	-2.1136	-2.6822	-1.7588
39	Br	-0.3314	-0.1306	4.2826
40	Br	2.9192	-1.5578	5.4044
41	Br	4.8246	-2.0426	2.2783

42	C	-7.8899	-0.4394	-2.0057
43	H	-8.7395	0.1884	-1.7077
44	H	-7.6407	-0.1806	-3.0392
45	H	-8.2264	-1.4799	-1.9779
46	H	-2.2027	-1.3438	0.9736

TpBr3CuNH_v1
Energy (POTENTIAL) = -1886.67472584 Eh

	Atom	X	Y	Z
1	N	1.7384	-0.8951	1.3152
2	N	0.3991	-0.6343	1.3342
3	N	2.1567	-1.0858	-1.1688
4	N	0.8610	-0.9490	-1.5665
5	N	2.4789	1.1320	-0.0097
6	N	1.2556	1.6881	-0.2458
7	C	-0.1047	-1.2192	2.4216
8	C	0.9076	-1.8810	3.1390
9	C	2.0620	-1.6455	2.3945
10	C	0.7428	-1.5958	-2.7181
11	C	1.9680	-2.1739	-3.1069
12	C	2.8416	-1.8175	-2.0822
13	C	1.4412	2.9929	-0.4482
14	C	2.8067	3.3159	-0.3408
15	C	3.4256	2.0984	-0.0643
16	B	2.6300	-0.4064	0.1409
17	H	3.7588	-0.6785	0.3579
18	Cu	-0.4077	0.4464	-0.2195
19	S	-3.1977	1.9012	-0.4111
20	O	-2.6841	2.4021	0.8713
21	O	-3.5144	2.8503	-1.4934
22	C	-4.6346	0.8913	-0.0974
23	C	-4.9260	0.5053	1.2130
24	C	-5.4315	0.4783	-1.1709
25	C	-6.0307	-0.3107	1.4462
26	H	-4.3044	0.8564	2.0290
27	C	-6.5285	-0.3400	-0.9181
28	H	-5.2046	0.8010	-2.1822
29	C	-6.8445	-0.7484	0.3889
30	H	-6.2697	-0.6079	2.4637
31	H	-7.1535	-0.6646	-1.7453
32	N	-2.0865	0.7798	-0.9760
33	H	-2.3789	0.4535	-1.8997
34	Br	-0.9154	-1.6696	-3.6217
35	Br	2.3413	-3.1894	-4.6481
36	Br	4.6691	-2.2398	-1.9219
37	Br	5.2604	1.7758	0.1956
38	Br	3.6135	5.0058	-0.5258
39	Br	0.0052	4.1421	-0.8386
40	Br	3.8063	-2.2422	2.7660
41	Br	0.7423	-2.8577	4.7382

42	Br	-1.9370	-1.1153	2.8436
43	C	-8.0554	-1.6051	0.6531
44	H	-8.9497	-0.9790	0.7658
45	H	-8.2450	-2.2963	-0.1737
46	H	-7.9431	-2.1847	1.5739

TpBr3CuNH_v4
Energy (POTENTIAL) = -1886.67473016 Eh

	Atom	X	Y	Z
1	N	1.7335	-0.8836	1.3235
2	N	0.3946	-0.6215	1.3444
3	N	2.1398	-1.0876	-1.1612
4	N	0.8445	-0.9423	-1.5568
5	N	2.4754	1.1347	-0.0138
6	N	1.2523	1.6952	-0.2403
7	C	-0.1073	-1.2009	2.4356
8	C	0.9060	-1.8601	3.1539
9	C	2.0589	-1.6291	2.4056
10	C	0.7167	-1.5997	-2.7012
11	C	1.9350	-2.1939	-3.0874
12	C	2.8149	-1.8358	-2.0687
13	C	1.4415	2.9987	-0.4482
14	C	2.8093	3.3161	-0.3544
15	C	3.4256	2.0969	-0.0798
16	B	2.6217	-0.4036	0.1429
17	H	3.7506	-0.6788	0.3557
18	Cu	-0.4146	0.4606	-0.2067
19	S	-3.2024	1.9203	-0.3951
20	O	-2.6919	2.4120	0.8921
21	O	-3.5120	2.8769	-1.4728
22	C	-4.6418	0.9100	-0.0952
23	C	-4.9406	0.5135	1.2099
24	C	-5.4338	0.5071	-1.1768
25	C	-6.0458	-0.3067	1.4300
26	H	-4.3270	0.8622	2.0329
27	C	-6.5302	-0.3148	-0.9371
28	H	-5.2049	0.8438	-2.1831
29	C	-6.8495	-0.7403	0.3642
30	H	-6.2920	-0.6092	2.4441
31	H	-7.1538	-0.6290	-1.7696
32	N	-2.0930	0.7981	-0.9618
33	H	-2.3845	0.4757	-1.8872
34	C	-8.0198	-1.6600	0.5975
35	H	-8.8772	-1.3769	-0.0216
36	H	-7.7551	-2.6911	0.3309
37	H	-8.3317	-1.6577	1.6454
38	Br	-1.9387	-1.0937	2.8604
39	Br	0.7436	-2.8289	4.7582
40	Br	3.8037	-2.2250	2.7762
41	Br	-0.9453	-1.6674	-3.5982

42	Br	2.2941	-3.2280	-4.6196
43	Br	4.6383	-2.2755	-1.9080
44	Br	0.0063	4.1526	-0.8273
45	Br	3.6216	5.0021	-0.5515
46	Br	5.2611	1.7673	0.1656

TpBr3CuNH_v2_SP
Energy (POTENTIAL) = -26380.7558562 Eh

	Atom	X	Y	Z
1	N	2.0270	-0.1229	0.2559
2	N	1.6762	1.2003	0.2326
3	N	-0.1539	-1.1429	0.9712
4	N	-0.7163	0.0765	1.2196
5	N	0.4786	-1.0450	-1.5195
6	N	0.1199	0.1896	-1.9724
7	C	2.7179	1.8860	0.7000
8	C	3.7786	1.0228	1.0346
9	C	3.2884	-0.2452	0.7365
10	C	-1.3755	-0.0304	2.3653
11	C	-1.2857	-1.3409	2.8833
12	C	-0.4890	-2.0106	1.9616
13	C	-0.1296	0.0809	-3.2782
14	C	0.0439	-1.2493	-3.7006
15	C	0.4334	-1.9268	-2.5453
16	B	0.9916	-1.2423	-0.0667
17	H	1.5106	-2.3003	0.0155
18	N	-1.7483	2.7582	-0.9250
19	S	-3.3865	2.4593	-1.1724
20	O	-4.1499	3.2619	-0.2001
21	O	-3.6793	2.6342	-2.6078
22	C	-3.5475	0.7334	-0.7585
23	C	-3.0790	-0.2280	-1.6601
24	C	-4.1185	0.3669	0.4571
25	C	-3.1148	-1.5683	-1.2958
26	H	-2.6968	0.0721	-2.6272
27	C	-4.1597	-0.9846	0.8011
28	H	-4.5057	1.1263	1.1249
29	C	-3.6364	-1.9655	-0.0516
30	H	-2.7368	-2.3190	-1.9843
31	H	-4.5911	-1.2777	1.7536
32	Cu	-0.3015	1.5895	-0.5214
33	C	-3.6335	-3.4195	0.3420
34	H	-3.9526	-3.5567	1.3785
35	H	-4.3053	-4.0014	-0.3002
36	H	-2.6328	-3.8511	0.2325
37	Br	4.1762	-1.8914	0.9445
38	Br	5.4691	1.4766	1.7270
39	Br	2.6914	3.7643	0.8876
40	Br	-0.1976	-1.9554	-5.4272
41	Br	-0.6575	1.5614	-4.3099

42	Br	0.8329	-3.7548	-2.3664
43	Br	0.0572	-3.8114	1.9993
44	Br	-2.0731	-2.0305	4.4484
45	Br	-2.2240	1.4555	3.1593
46	H	-1.5336	3.5824	-1.4982

TpBr3CuNH_v3_SP
Energy (POTENTIAL) = -26380.754622 Eh

	Atom	X	Y	Z
1	N	2.4525	0.5003	-0.6562
2	N	1.2862	1.1945	-0.4858
3	N	2.3291	-0.9204	1.4288
4	N	1.1280	-0.4746	1.9016
5	N	1.6735	-1.9041	-0.8163
6	N	0.3341	-1.6560	-0.8063
7	C	1.4685	2.4028	-1.0249
8	C	2.7591	2.5146	-1.5698
9	C	3.3462	1.2789	-1.3096
10	C	1.1455	-0.6133	3.2276
11	C	2.3722	-1.1598	3.6483
12	C	3.0903	-1.3383	2.4682
13	C	-0.2414	-2.6292	-1.5025
14	C	0.7171	-3.5441	-1.9847
15	C	1.9268	-3.0358	-1.5194
16	B	2.6374	-0.9370	-0.0931
17	H	3.7537	-1.2823	-0.2628
18	N	-2.0195	-0.3424	0.8757
19	S	-3.1794	0.4972	1.7342
20	O	-3.4794	-0.1435	3.0285
21	O	-2.7831	1.9152	1.7100
22	C	-4.5887	0.2533	0.6633
23	C	-4.7353	1.0599	-0.4703
24	C	-5.4836	-0.7852	0.9353
25	C	-5.8021	0.8227	-1.3319
26	H	-4.0283	1.8593	-0.6663
27	C	-6.5434	-1.0090	0.0591
28	H	-5.3541	-1.3977	1.8212
29	C	-6.7201	-0.2125	-1.0836
30	H	-5.9276	1.4477	-2.2118
31	H	-7.2462	-1.8115	0.2658
32	Cu	-0.2589	0.2077	0.4664
33	Br	0.1428	3.7392	-0.9892
34	Br	3.5236	3.9956	-2.4425
35	Br	5.0825	0.7230	-1.7709
36	Br	0.4351	-5.0946	-3.0158
37	Br	3.6529	-3.7405	-1.7813
38	Br	-2.1136	-2.6822	-1.7588
39	Br	-0.3314	-0.1306	4.2826
40	Br	2.9192	-1.5578	5.4044
41	Br	4.8246	-2.0426	2.2783

42	C	-7.8899	-0.4394	-2.0057
43	H	-8.7395	0.1884	-1.7077
44	H	-7.6407	-0.1806	-3.0392
45	H	-8.2264	-1.4799	-1.9779
46	H	-2.2027	-1.3438	0.9736

TpBr3CuNH_v1_SP
Energy (POTENTIAL) = -26380.7571955 Eh

	Atom	X	Y	Z
1	N	1.7384	-0.8951	1.3152
2	N	0.3991	-0.6343	1.3342
3	N	2.1567	-1.0858	-1.1688
4	N	0.8610	-0.9490	-1.5665
5	N	2.4789	1.1320	-0.0097
6	N	1.2556	1.6881	-0.2458
7	C	-0.1047	-1.2192	2.4216
8	C	0.9076	-1.8810	3.1390
9	C	2.0620	-1.6455	2.3945
10	C	0.7428	-1.5958	-2.7181
11	C	1.9680	-2.1739	-3.1070
12	C	2.8416	-1.8175	-2.0822
13	C	1.4412	2.9929	-0.4482
14	C	2.8067	3.3159	-0.3408
15	C	3.4256	2.0984	-0.0643
16	B	2.6300	-0.4064	0.1409
17	H	3.7588	-0.6785	0.3579
18	Cu	-0.4077	0.4464	-0.2195
19	S	-3.1977	1.9012	-0.4111
20	O	-2.6841	2.4021	0.8713
21	O	-3.5144	2.8503	-1.4934
22	C	-4.6346	0.8913	-0.0974
23	C	-4.9260	0.5053	1.2130
24	C	-5.4315	0.4783	-1.1709
25	C	-6.0307	-0.3107	1.4462
26	H	-4.3044	0.8564	2.0290
27	C	-6.5285	-0.3400	-0.9181
28	H	-5.2046	0.8010	-2.1822
29	C	-6.8445	-0.7484	0.3889
30	H	-6.2697	-0.6079	2.4637
31	H	-7.1535	-0.6646	-1.7453
32	N	-2.0865	0.7798	-0.9760
33	H	-2.3789	0.4535	-1.8997
34	Br	-0.9154	-1.6696	-3.6217
35	Br	2.3413	-3.1894	-4.6481
36	Br	4.6691	-2.2398	-1.9219
37	Br	5.2604	1.7758	0.1956
38	Br	3.6135	5.0058	-0.5258
39	Br	0.0052	4.1421	-0.8386
40	Br	3.8063	-2.2422	2.7660
41	Br	0.7423	-2.8577	4.7382

42	Br	-1.9370	-1.1153	2.8436
43	C	-8.0554	-1.6051	0.6531
44	H	-8.9497	-0.9790	0.7658
45	H	-8.2450	-2.2963	-0.1737
46	H	-7.9431	-2.1847	1.5739

TpBr3CuNH_v4_SP
Energy (POTENTIAL) = -26380.7572018 Eh

	Atom	X	Y	Z
1	N	1.7335	-0.8836	1.3235
2	N	0.3946	-0.6215	1.3444
3	N	2.1398	-1.0876	-1.1612
4	N	0.8445	-0.9423	-1.5568
5	N	2.4754	1.1347	-0.0138
6	N	1.2523	1.6952	-0.2403
7	C	-0.1073	-1.2009	2.4356
8	C	0.9060	-1.8601	3.1539
9	C	2.0589	-1.6291	2.4056
10	C	0.7167	-1.5997	-2.7012
11	C	1.9350	-2.1939	-3.0874
12	C	2.8149	-1.8358	-2.0687
13	C	1.4415	2.9987	-0.4482
14	C	2.8093	3.3161	-0.3544
15	C	3.4256	2.0969	-0.0798
16	B	2.6217	-0.4036	0.1429
17	H	3.7506	-0.6788	0.3557
18	Cu	-0.4146	0.4606	-0.2067
19	S	-3.2024	1.9203	-0.3951
20	O	-2.6919	2.4120	0.8921
21	O	-3.5120	2.8769	-1.4728
22	C	-4.6418	0.9100	-0.0952
23	C	-4.9406	0.5135	1.2099
24	C	-5.4338	0.5071	-1.1768
25	C	-6.0458	-0.3067	1.4300
26	H	-4.3270	0.8622	2.0329
27	C	-6.5302	-0.3148	-0.9371
28	H	-5.2049	0.8438	-2.1831
29	C	-6.8495	-0.7403	0.3642
30	H	-6.2920	-0.6092	2.4441
31	H	-7.1538	-0.6290	-1.7696
32	N	-2.0930	0.7981	-0.9618
33	H	-2.3845	0.4757	-1.8872
34	C	-8.0198	-1.6600	0.5975
35	H	-8.8772	-1.3769	-0.0216
36	H	-7.7551	-2.6911	0.3309
37	H	-8.3317	-1.6577	1.6454
38	Br	-1.9387	-1.0937	2.8604
39	Br	0.7436	-2.8289	4.7582
40	Br	3.8037	-2.2250	2.7762
41	Br	-0.9453	-1.6674	-3.5982

42	Br	2.2941	-3.2280	-4.6196
43	Br	4.6383	-2.2755	-1.9080
44	Br	0.0063	4.1526	-0.8273
45	Br	3.6216	5.0021	-0.5515
46	Br	5.2611	1.7673	0.1656

TpBr3CuNH_Si_s_v2_SP
Energy (POTENTIAL) = -26981.8747129 Eh

	Atom	X	Y	Z
1	N	1.6215	0.2820	1.2964
2	N	1.3054	1.6135	1.2673
3	N	-0.6487	-0.7489	1.6269
4	N	-1.2317	0.4573	1.8813
5	N	0.3484	-0.4759	-0.7368
6	N	0.0065	0.7905	-1.1046
7	C	2.3421	2.2697	1.7850
8	C	3.3594	1.3773	2.1719
9	C	2.8521	0.1239	1.8401
10	C	-2.0817	0.2785	2.8841
11	C	-2.0945	-1.0703	3.2963
12	C	-1.1602	-1.6843	2.4667
13	C	0.0634	0.8629	-2.4325
14	C	0.4359	-0.3837	-2.9678
15	C	0.6076	-1.2015	-1.8517
16	B	0.6322	-0.7998	0.7566
17	H	1.1254	-1.8704	0.8359
18	N	-2.1352	3.0120	-0.2214
19	S	-3.6802	2.5189	-0.6641
20	O	-4.6072	2.8508	0.4312
21	O	-3.9680	3.0404	-2.0155
22	C	-3.5603	0.7388	-0.7736
23	C	-3.0521	0.1652	-1.9432
24	C	-3.9591	-0.0540	0.3013
25	C	-2.9004	-1.2154	-2.0090
26	H	-2.7901	0.7938	-2.7862
27	C	-3.8036	-1.4378	0.2175
28	H	-4.3880	0.4076	1.1827
29	C	-3.2555	-2.0368	-0.9252
30	H	-2.5058	-1.6670	-2.9147
31	H	-4.1128	-2.0601	1.0519
32	Cu	-0.6350	2.0394	0.4075
33	C	-3.0363	-3.5259	-0.9995
34	H	-3.5220	-4.0484	-0.1711
35	H	-3.4183	-3.9365	-1.9403
36	H	-1.9652	-3.7589	-0.9607
37	Br	3.6826	-1.5472	2.0842
38	Br	5.0223	1.7820	2.9561
39	Br	2.3722	4.1509	1.9280
40	Br	0.6372	-0.8429	-4.7815
41	Br	-0.3459	2.4914	-3.3248

42	Br	1.0843	-3.0199	-1.8149
43	Br	-0.6623	-3.4980	2.4283
44	Br	-3.1481	-1.8631	4.6400
45	Br	-3.0798	1.7179	3.5792
46	H	-1.9023	3.7657	-0.8757
47	Si	-1.2373	5.8508	-3.7322
48	C	-2.7628	6.2789	-4.7581
49	C	0.3174	6.7851	-4.2634
50	C	-1.5583	6.0313	-1.8929
51	H	-2.5469	6.1854	-5.8278
52	H	-3.6025	5.6161	-4.5256
53	H	-3.0834	7.3117	-4.5664
54	H	1.1984	6.4556	-3.7027
55	H	0.5203	6.6305	-5.3285
56	H	0.1942	7.8634	-4.0937
57	C	-2.8748	6.0875	-1.3842
58	C	-0.4943	6.0731	-0.9633
59	C	-3.1175	6.1925	-0.0130
60	H	-3.7204	6.0369	-2.0629
61	C	-0.7375	6.1818	0.4071
62	H	0.5345	6.0266	-1.3130
63	C	-2.0495	6.2418	0.8867
64	H	-4.1396	6.2250	0.3526
65	H	0.0951	6.2229	1.1031
66	H	-2.2379	6.3191	1.9537

TpBr3CuNH_Si_s_v3_SP
Energy (POTENTIAL) = -26981.8874829 Eh

	Atom	X	Y	Z
1	N	2.1040	0.5010	0.0600
2	N	1.4978	1.7230	0.1432
3	N	0.1357	-0.9587	0.5796
4	N	-0.5831	0.1312	0.9793
5	N	0.7845	-0.5219	-1.8558
6	N	0.0335	0.5725	-2.1596
7	C	2.2887	2.5084	0.8693
8	C	3.4495	1.8173	1.2639
9	C	3.2814	0.5424	0.7302
10	C	-1.2171	-0.2092	2.0909
11	C	-0.9631	-1.5579	2.4286
12	C	-0.0878	-1.9890	1.4378
13	C	-0.3191	0.4693	-3.4425
14	C	0.1964	-0.7141	-3.9995
15	C	0.8948	-1.3102	-2.9509
16	B	1.3109	-0.7502	-0.4203
17	H	2.0173	-1.6972	-0.4104
18	N	-2.0488	2.8592	-0.8272
19	S	-3.6763	2.4318	-0.8847
20	O	-4.3841	3.1615	0.1833
21	O	-4.1445	2.6122	-2.2732
22	C	-3.6828	0.6903	-0.4913
23	C	-3.0572	-0.2130	-1.3557
24	C	-4.3473	0.2480	0.6497
25	C	-3.0307	-1.5623	-1.0283
26	H	-2.5952	0.1375	-2.2664
27	C	-4.3301	-1.1141	0.9547
28	H	-4.8509	0.9580	1.2945
29	C	-3.6534	-2.0323	0.1415
30	H	-2.5246	-2.2624	-1.6876
31	H	-4.8347	-1.4633	1.8507
32	Cu	-0.4328	1.8959	-0.6231
33	C	-3.5687	-3.4905	0.5099
34	H	-4.2292	-3.7364	1.3452
35	H	-3.8294	-4.1307	-0.3397
36	H	-2.5450	-3.7499	0.8051
37	Br	4.4580	-0.9165	0.8659
38	Br	4.9098	2.4776	2.2489
39	Br	1.8067	4.2778	1.2877
40	Br	0.0190	-1.3289	-5.7678
41	Br	-1.3882	1.7629	-4.2819

42	Br	1.8542	-2.9248	-2.9895
43	Br	0.6792	-3.6959	1.2300
44	Br	-1.6509	-2.5433	3.8778
45	Br	-2.1799	1.0705	3.0853
46	H	-1.9782	3.7079	-1.4006
47	Si	1.5916	4.1619	-2.9597
48	C	2.3467	5.7056	-2.1633
49	C	0.8926	4.6115	-4.6625
50	C	2.8218	2.7463	-3.0524
51	H	1.5682	6.4338	-1.9098
52	H	2.9034	5.4833	-1.2497
53	H	3.0352	6.1856	-2.8719
54	H	0.6855	3.7323	-5.2777
55	H	-0.0389	5.1809	-4.5704
56	H	1.6155	5.2389	-5.2012
57	C	3.8705	2.6246	-2.1158
58	C	2.6872	1.7195	-4.0110
59	C	4.7341	1.5291	-2.1291
60	H	4.0204	3.3963	-1.3659
61	C	3.5461	0.6200	-4.0250
62	H	1.9032	1.7769	-4.7611
63	C	4.5704	0.5171	-3.0794
64	H	5.5364	1.4640	-1.3996
65	H	3.4217	-0.1539	-4.7773
66	H	5.2382	-0.3396	-3.0872

TpBr3CuNH_Si_s_v4_SP
Energy (POTENTIAL) = -26981.8784902 Eh

	Atom	X	Y	Z
1	N	2.1864	-0.3035	0.1460
2	N	1.8776	1.0038	0.4069
3	N	0.0718	-1.4382	0.9225
4	N	-0.4137	-0.2969	1.4899
5	N	0.3841	-0.8095	-1.5374
6	N	-0.1142	0.4576	-1.5760
7	C	3.0154	1.6089	0.7269
8	C	4.1013	0.7110	0.6815
9	C	3.5199	-0.4962	0.3068
10	C	-0.9679	-0.6367	2.6498
11	C	-0.8841	-2.0288	2.8511
12	C	-0.2089	-2.4941	1.7254
13	C	-0.4164	0.7296	-2.8447
14	C	-0.1407	-0.3811	-3.6648
15	C	0.3716	-1.3340	-2.7860
16	B	1.0813	-1.3258	-0.2504
17	H	1.5634	-2.3840	-0.4608
18	N	-1.6711	2.8278	0.2851
19	S	-3.3386	2.7293	0.1271
20	O	-3.9523	3.2654	1.3579
21	O	-3.7262	3.3292	-1.1621
22	C	-3.6007	0.9655	0.0717
23	C	-3.3537	0.2796	-1.1203
24	C	-3.9652	0.2876	1.2342
25	C	-3.4049	-1.1107	-1.1222
26	H	-3.1183	0.8271	-2.0240
27	C	-4.0231	-1.1051	1.2113
28	H	-4.1820	0.8408	2.1394
29	C	-3.7199	-1.8248	0.0460
30	H	-3.1951	-1.6511	-2.0413
31	H	-4.2941	-1.6410	2.1164
32	Cu	-0.3939	1.4417	0.2418
33	C	-3.7597	-3.3310	0.0342
34	H	-3.6098	-3.7442	1.0356
35	H	-4.7314	-3.6894	-0.3282
36	H	-2.9939	-3.7434	-0.6299
37	Br	4.3795	-2.1526	0.0479
38	Br	5.9167	1.0611	1.0427
39	Br	3.0600	3.4400	1.1855
40	Br	-0.4042	-0.5444	-5.5209
41	Br	-1.1078	2.4028	-3.3515

42	Br	0.9527	-3.0767	-3.1882
43	Br	0.2570	-4.2678	1.3082
44	Br	-1.5441	-3.0291	4.3018
45	Br	-1.6551	0.6666	3.8207
46	H	-1.4020	3.8232	0.1597
47	Si	-1.1599	6.2024	0.8288
48	C	-2.9979	6.6041	0.6572
49	C	-0.0356	7.5907	0.2164
50	C	-0.7043	5.6117	2.5481
51	H	-3.2546	6.7956	-0.3903
52	H	-3.6232	5.7789	1.0099
53	H	-3.2535	7.5011	1.2374
54	H	1.0183	7.2940	0.2128
55	H	-0.3053	7.8778	-0.8057
56	H	-0.1343	8.4808	0.8522
57	C	-1.6048	4.8356	3.3110
58	C	0.5660	5.8793	3.1030
59	C	-1.2554	4.3669	4.5780
60	H	-2.5833	4.5871	2.9103
61	C	0.9182	5.4038	4.3674
62	H	1.2856	6.4776	2.5489
63	C	0.0074	4.6461	5.1102
64	H	-1.9732	3.7904	5.1567
65	H	1.8988	5.6314	4.7773
66	H	0.2781	4.2805	6.0970

TpBr3CuNH_Si_s_v5_SP
Energy (POTENTIAL) = -26981.8908047 Eh

	Atom	X	Y	Z
1	N	1.7569	0.3409	-0.0517
2	N	1.3129	1.5966	0.2693
3	N	-0.2365	-0.9797	0.7029
4	N	-0.9092	0.1051	1.1848
5	N	0.0653	-0.3564	-1.7855
6	N	-0.3634	0.9278	-1.9448
7	C	2.3777	2.2932	0.6657
8	C	3.5428	1.5050	0.6096
9	C	3.0948	0.2712	0.1474
10	C	-1.4151	-0.2387	2.3614
11	C	-1.0976	-1.5790	2.6718
12	C	-0.3415	-2.0066	1.5854
13	C	-0.7317	1.0808	-3.2165
14	C	-0.5598	-0.1273	-3.9193
15	C	-0.0500	-1.0098	-2.9655
16	B	0.7641	-0.7889	-0.4632
17	H	1.3547	-1.7949	-0.6506
18	N	-2.4999	2.6714	-0.4072
19	S	-4.0180	2.0433	-0.7266
20	O	-4.9736	2.7262	0.1708
21	O	-4.2812	2.0984	-2.1787
22	C	-3.9085	0.3298	-0.2494
23	C	-3.2579	-0.5675	-1.0981
24	C	-4.5111	-0.0987	0.9336
25	C	-3.1676	-1.9056	-0.7257
26	H	-2.8400	-0.2279	-2.0376
27	C	-4.4213	-1.4437	1.2846
28	H	-5.0343	0.6109	1.5634
29	C	-3.7370	-2.3620	0.4729
30	H	-2.6561	-2.6084	-1.3767
31	H	-4.8816	-1.7859	2.2074
32	Cu	-0.7698	1.9055	-0.1706
33	C	-3.6043	-3.8016	0.8964
34	H	-2.9048	-3.8914	1.7356
35	H	-4.5637	-4.2064	1.2354
36	H	-3.2312	-4.4294	0.0829
37	Br	4.1231	-1.2728	-0.1693
38	Br	5.3020	2.0027	1.0574
39	Br	2.2601	4.0861	1.2328
40	Br	-0.9144	-0.4697	-5.7351
41	Br	-1.3295	2.7422	-3.8600

42	Br	0.4133	-2.8172	-3.1948
43	Br	0.4081	-3.7080	1.2956
44	Br	-1.5957	-2.5626	4.1970
45	Br	-2.3623	1.0000	3.4196
46	H	-2.4592	3.5152	-0.9908
47	Si	-1.5406	5.1299	1.5827
48	C	-0.3762	6.2651	2.5587
49	C	-3.1186	4.7276	2.5414
50	C	-1.9203	5.8606	-0.1105
51	H	0.0143	5.7612	3.4494
52	H	0.4743	6.5941	1.9521
53	H	-0.9100	7.1662	2.8904
54	H	-3.7116	3.9740	2.0146
55	H	-2.8702	4.3426	3.5366
56	H	-3.7388	5.6239	2.6768
57	C	-0.9057	6.4531	-0.8929
58	C	-3.2020	5.7357	-0.6941
59	C	-1.1537	6.8922	-2.1946
60	H	0.0950	6.5720	-0.4836
61	C	-3.4512	6.1670	-1.9992
62	H	-4.0120	5.2783	-0.1320
63	C	-2.4272	6.7457	-2.7553
64	H	-0.3544	7.3483	-2.7729
65	H	-4.4447	6.0519	-2.4240
66	H	-2.6204	7.0832	-3.7698

TpBr3CuNH_Si_s_v6_SP
Energy (POTENTIAL) = -26981.8899801 Eh

	Atom	X	Y	Z
1	Cu	-0.7372	-0.3937	-1.5495
2	N	1.2242	-1.1799	-2.4952
3	N	2.3481	-0.6578	-1.9266
4	N	0.1000	1.5091	-1.3895
5	N	1.4260	1.5594	-1.0660
6	N	0.1741	-0.8812	0.2683
7	N	1.4701	-0.4786	0.4288
8	B	2.2243	0.2757	-0.7039
9	C	1.6288	-2.0360	-3.4260
10	C	3.0362	-2.0909	-3.4892
11	C	3.4498	-1.1923	-2.5104
12	C	-0.3076	2.7639	-1.5911
13	C	0.7608	3.6626	-1.4158
14	C	1.8404	2.8485	-1.0774
15	C	-0.1685	-1.5530	1.3689
16	C	0.9070	-1.6025	2.2738
17	C	1.9271	-0.9069	1.6288
18	H	3.2984	0.5832	-0.3219
19	N	-1.9817	-0.7561	-2.9117
20	S	-3.6337	-0.5787	-3.0681
21	O	-4.1041	-1.6823	-3.9277
22	O	-3.9701	0.8013	-3.4681
23	C	-4.2241	-0.8490	-1.4043
24	C	-4.6819	-2.1152	-1.0431
25	C	-4.2598	0.2204	-0.5037
26	C	-5.1827	-2.3090	0.2455
27	H	-4.6584	-2.9293	-1.7594
28	C	-4.7570	0.0068	0.7775
29	H	-3.9163	1.2024	-0.8066
30	C	-5.2308	-1.2565	1.1710
31	H	-5.5488	-3.2910	0.5315
32	H	-4.7862	0.8326	1.4833
33	C	-5.7781	-1.4618	2.5607
34	H	-5.0681	-1.1119	3.3185
35	H	-6.7036	-0.8911	2.7024
36	H	-5.9983	-2.5147	2.7564
37	Br	-1.8915	-2.2775	1.5858
38	Br	-2.0864	3.1666	-2.0424
39	Br	0.3959	-3.0155	-4.4648
40	Br	4.1087	-3.1362	-4.6276
41	Br	5.2107	-0.7450	-2.0233

42	Br	3.6702	-0.5864	2.2595
43	Br	0.9555	-2.4181	3.9691
44	Br	0.7383	5.5341	-1.6064
45	Br	3.6008	3.3863	-0.6917
46	H	-1.5872	-0.7307	-3.8709
47	Si	-0.5414	0.5619	-5.8398
48	C	-1.5256	2.1682	-5.6854
49	C	-0.8932	-0.3423	-7.4623
50	C	1.2957	0.8302	-5.5717
51	H	-2.5695	1.9666	-5.4269
52	H	-1.1105	2.8155	-4.9083
53	H	-1.4997	2.7189	-6.6354
54	H	-0.3914	-1.3142	-7.5045
55	H	-1.9688	-0.5162	-7.5766
56	H	-0.5556	0.2507	-8.3230
57	C	1.7663	1.7485	-4.6080
58	C	2.2577	0.0995	-6.3007
59	C	3.1310	1.9407	-4.3970
60	H	1.0602	2.3240	-4.0164
61	C	3.6245	0.2922	-6.0930
62	H	1.9381	-0.6231	-7.0470
63	C	4.0662	1.2141	-5.1409
64	H	3.4685	2.6584	-3.6556
65	H	4.3449	-0.2775	-6.6728
66	H	5.1293	1.3654	-4.9779

TpBr3CuNH_Si_s_v1_SP
Energy (POTENTIAL) = -26981.8902286 Eh

	Atom	X	Y	Z
1	Cu	-0.6558	-0.4463	-1.6269
2	N	1.3044	-1.3318	-2.5553
3	N	2.4433	-0.8851	-1.9534
4	N	0.3459	1.3750	-1.4474
5	N	1.6410	1.3481	-1.0198
6	N	0.2094	-1.0701	0.1689
7	N	1.5185	-0.7439	0.3863
8	B	2.3490	0.0088	-0.6950
9	C	1.6758	-2.2138	-3.4731
10	C	3.0783	-2.3602	-3.5002
11	C	3.5232	-1.4906	-2.5093
12	C	0.0168	2.6535	-1.6334
13	C	1.0993	3.4928	-1.3185
14	C	2.1118	2.6149	-0.9372
15	C	-0.2002	-1.7791	1.2220
16	C	0.8404	-1.9241	2.1566
17	C	1.9138	-1.2503	1.5776
18	H	3.4311	0.2301	-0.2770
19	N	-1.9247	-0.6687	-2.9975
20	S	-3.5685	-0.3994	-3.1148
21	O	-4.1217	-1.4763	-3.9587
22	O	-3.8358	0.9962	-3.5116
23	C	-4.1327	-0.6272	-1.4350
24	C	-4.7895	-1.8073	-1.0927
25	C	-3.9565	0.4031	-0.5042
26	C	-5.2800	-1.9539	0.2068
27	H	-4.9243	-2.5904	-1.8306
28	C	-4.4445	0.2351	0.7872
29	H	-3.4597	1.3229	-0.7915
30	C	-5.1181	-0.9406	1.1619
31	H	-5.8027	-2.8672	0.4771
32	H	-4.3108	1.0299	1.5159
33	C	-5.6364	-1.1016	2.5684
34	H	-4.8136	-1.0741	3.2928
35	H	-6.3194	-0.2861	2.8312
36	H	-6.1688	-2.0479	2.6960
37	Br	-1.9584	-2.4366	1.3371
38	Br	-1.6721	3.1336	-2.2907
39	Br	0.4024	-3.0995	-4.5467
40	Br	4.1142	-3.4395	-4.6431
41	Br	5.2985	-1.1412	-1.9924

42	Br	3.6476	-1.0433	2.2786
43	Br	0.7909	-2.8167	3.8125
44	Br	1.1805	5.3678	-1.4408
45	Br	3.8640	3.0500	-0.4142
46	H	-1.5547	-0.6819	-3.9688
47	Si	-0.6357	0.3468	-6.0778
48	C	-1.9160	1.7224	-6.2714
49	C	-0.4714	-0.7374	-7.6166
50	C	1.0216	1.0007	-5.4942
51	H	-2.8600	1.3109	-6.6439
52	H	-2.1280	2.2065	-5.3141
53	H	-1.5723	2.4855	-6.9825
54	H	0.2951	-1.5083	-7.4899
55	H	-1.4191	-1.2402	-7.8386
56	H	-0.1998	-0.1311	-8.4912
57	C	1.1281	2.2509	-4.8483
58	C	2.2023	0.2424	-5.6449
59	C	2.3558	2.7233	-4.3827
60	H	0.2436	2.8684	-4.7188
61	C	3.4293	0.7083	-5.1728
62	H	2.1648	-0.7243	-6.1398
63	C	3.5104	1.9509	-4.5372
64	H	2.4136	3.6970	-3.9055
65	H	4.3237	0.1060	-5.3047
66	H	4.4658	2.3162	-4.1717

TpBr3CuNH_Si_t_v2
Energy (POTENTIAL) = -2487.67510491 Eh

	Atom	X	Y	Z
1	N	1.6215	0.2820	1.2964
2	N	1.3054	1.6135	1.2673
3	N	-0.6487	-0.7489	1.6269
4	N	-1.2317	0.4573	1.8813
5	N	0.3484	-0.4759	-0.7368
6	N	0.0065	0.7905	-1.1046
7	C	2.3421	2.2697	1.7850
8	C	3.3594	1.3773	2.1719
9	C	2.8521	0.1239	1.8401
10	C	-2.0817	0.2785	2.8841
11	C	-2.0945	-1.0703	3.2963
12	C	-1.1602	-1.6843	2.4667
13	C	0.0634	0.8629	-2.4325
14	C	0.4359	-0.3837	-2.9678
15	C	0.6076	-1.2015	-1.8517
16	B	0.6322	-0.7998	0.7566
17	H	1.1254	-1.8704	0.8359
18	N	-2.1352	3.0120	-0.2214
19	S	-3.6802	2.5189	-0.6641
20	O	-4.6072	2.8508	0.4312
21	O	-3.9680	3.0404	-2.0155
22	C	-3.5603	0.7388	-0.7736
23	C	-3.0521	0.1652	-1.9432
24	C	-3.9591	-0.0540	0.3013
25	C	-2.9004	-1.2154	-2.0090
26	H	-2.7901	0.7938	-2.7862
27	C	-3.8036	-1.4378	0.2175
28	H	-4.3880	0.4076	1.1827
29	C	-3.2555	-2.0368	-0.9252
30	H	-2.5058	-1.6670	-2.9147
31	H	-4.1128	-2.0601	1.0519
32	Cu	-0.6350	2.0394	0.4075
33	C	-3.0363	-3.5259	-0.9995
34	H	-3.5220	-4.0484	-0.1711
35	H	-3.4183	-3.9365	-1.9403
36	H	-1.9652	-3.7589	-0.9607
37	Br	3.6826	-1.5472	2.0842
38	Br	5.0223	1.7820	2.9561
39	Br	2.3722	4.1509	1.9280
40	Br	0.6372	-0.8429	-4.7815
41	Br	-0.3459	2.4914	-3.3248

42	Br	1.0843	-3.0199	-1.8149
43	Br	-0.6623	-3.4980	2.4283
44	Br	-3.1481	-1.8631	4.6400
45	Br	-3.0798	1.7179	3.5792
46	H	-1.9023	3.7657	-0.8757
47	Si	-1.2373	5.8508	-3.7322
48	C	-2.7628	6.2789	-4.7581
49	C	0.3174	6.7851	-4.2634
50	C	-1.5583	6.0313	-1.8929
51	H	-2.5469	6.1854	-5.8278
52	H	-3.6025	5.6161	-4.5256
53	H	-3.0834	7.3117	-4.5664
54	H	1.1984	6.4556	-3.7027
55	H	0.5203	6.6305	-5.3285
56	H	0.1942	7.8634	-4.0937
57	C	-2.8748	6.0875	-1.3842
58	C	-0.4943	6.0731	-0.9633
59	C	-3.1175	6.1925	-0.0130
60	H	-3.7204	6.0369	-2.0629
61	C	-0.7375	6.1818	0.4071
62	H	0.5345	6.0266	-1.3130
63	C	-2.0495	6.2418	0.8867
64	H	-4.1396	6.2250	0.3526
65	H	0.0951	6.2229	1.1031
66	H	-2.2379	6.3191	1.9537

TpBr3CuNH_Si_t_v3
Energy (POTENTIAL) = -2487.68025473 Eh

	Atom	X	Y	Z
1	N	2.1040	0.5010	0.0600
2	N	1.4978	1.7230	0.1432
3	N	0.1357	-0.9587	0.5796
4	N	-0.5831	0.1312	0.9793
5	N	0.7845	-0.5219	-1.8558
6	N	0.0335	0.5725	-2.1596
7	C	2.2887	2.5084	0.8693
8	C	3.4495	1.8173	1.2639
9	C	3.2814	0.5424	0.7302
10	C	-1.2171	-0.2092	2.0909
11	C	-0.9631	-1.5579	2.4286
12	C	-0.0878	-1.9890	1.4378
13	C	-0.3191	0.4693	-3.4425
14	C	0.1964	-0.7141	-3.9995
15	C	0.8948	-1.3102	-2.9509
16	B	1.3109	-0.7502	-0.4203
17	H	2.0173	-1.6972	-0.4104
18	N	-2.0488	2.8592	-0.8272
19	S	-3.6763	2.4318	-0.8847
20	O	-4.3841	3.1615	0.1833
21	O	-4.1445	2.6122	-2.2732
22	C	-3.6828	0.6903	-0.4913
23	C	-3.0572	-0.2130	-1.3557
24	C	-4.3473	0.2480	0.6497
25	C	-3.0307	-1.5623	-1.0283
26	H	-2.5952	0.1375	-2.2664
27	C	-4.3301	-1.1141	0.9547
28	H	-4.8509	0.9580	1.2945
29	C	-3.6534	-2.0323	0.1415
30	H	-2.5246	-2.2624	-1.6876
31	H	-4.8347	-1.4633	1.8507
32	Cu	-0.4328	1.8959	-0.6231
33	C	-3.5687	-3.4905	0.5099
34	H	-4.2292	-3.7364	1.3452
35	H	-3.8294	-4.1307	-0.3397
36	H	-2.5450	-3.7499	0.8051
37	Br	4.4580	-0.9165	0.8659
38	Br	4.9098	2.4776	2.2489
39	Br	1.8067	4.2778	1.2877
40	Br	0.0190	-1.3289	-5.7678
41	Br	-1.3882	1.7629	-4.2819

42	Br	1.8542	-2.9248	-2.9895
43	Br	0.6792	-3.6959	1.2300
44	Br	-1.6509	-2.5433	3.8778
45	Br	-2.1799	1.0705	3.0853
46	H	-1.9782	3.7079	-1.4006
47	Si	1.5915	4.1619	-2.9597
48	C	2.3467	5.7056	-2.1633
49	C	0.8926	4.6115	-4.6625
50	C	2.8218	2.7463	-3.0524
51	H	1.5682	6.4338	-1.9098
52	H	2.9034	5.4833	-1.2497
53	H	3.0352	6.1856	-2.8719
54	H	0.6855	3.7323	-5.2777
55	H	-0.0389	5.1809	-4.5704
56	H	1.6155	5.2389	-5.2012
57	C	3.8705	2.6246	-2.1158
58	C	2.6872	1.7195	-4.0110
59	C	4.7341	1.5291	-2.1291
60	H	4.0204	3.3963	-1.3659
61	C	3.5461	0.6200	-4.0250
62	H	1.9032	1.7769	-4.7611
63	C	4.5704	0.5171	-3.0794
64	H	5.5364	1.4640	-1.3996
65	H	3.4217	-0.1539	-4.7773
66	H	5.2382	-0.3396	-3.0872

TpBr3CuNH_Si_t_v4
Energy (POTENTIAL) = -2487.67186850 Eh

	Atom	X	Y	Z
1	N	2.1864	-0.3035	0.1460
2	N	1.8776	1.0038	0.4069
3	N	0.0718	-1.4382	0.9225
4	N	-0.4137	-0.2969	1.4899
5	N	0.3841	-0.8095	-1.5374
6	N	-0.1142	0.4576	-1.5760
7	C	3.0154	1.6089	0.7269
8	C	4.1013	0.7111	0.6815
9	C	3.5199	-0.4962	0.3068
10	C	-0.9679	-0.6367	2.6498
11	C	-0.8841	-2.0288	2.8511
12	C	-0.2089	-2.4941	1.7254
13	C	-0.4164	0.7296	-2.8447
14	C	-0.1407	-0.3811	-3.6648
15	C	0.3716	-1.3340	-2.7860
16	B	1.0813	-1.3258	-0.2504
17	H	1.5634	-2.3840	-0.4608
18	N	-1.6711	2.8278	0.2851
19	S	-3.3386	2.7293	0.1271
20	O	-3.9523	3.2654	1.3579
21	O	-3.7262	3.3292	-1.1621
22	C	-3.6007	0.9655	0.0717
23	C	-3.3537	0.2796	-1.1203
24	C	-3.9652	0.2876	1.2342
25	C	-3.4049	-1.1107	-1.1222
26	H	-3.1183	0.8271	-2.0240
27	C	-4.0231	-1.1051	1.2113
28	H	-4.1820	0.8408	2.1394
29	C	-3.7199	-1.8248	0.0460
30	H	-3.1951	-1.6511	-2.0413
31	H	-4.2941	-1.6410	2.1164
32	Cu	-0.3939	1.4417	0.2418
33	C	-3.7597	-3.3310	0.0342
34	H	-3.6098	-3.7442	1.0356
35	H	-4.7314	-3.6894	-0.3282
36	H	-2.9939	-3.7434	-0.6299
37	Br	4.3795	-2.1526	0.0479
38	Br	5.9167	1.0611	1.0427
39	Br	3.0600	3.4400	1.1855
40	Br	-0.4042	-0.5444	-5.5209
41	Br	-1.1078	2.4028	-3.3515

42	Br	0.9527	-3.0767	-3.1882
43	Br	0.2570	-4.2678	1.3082
44	Br	-1.5441	-3.0291	4.3018
45	Br	-1.6551	0.6666	3.8207
46	H	-1.4020	3.8232	0.1597
47	Si	-1.1599	6.2024	0.8288
48	C	-2.9979	6.6041	0.6572
49	C	-0.0356	7.5907	0.2164
50	C	-0.7043	5.6117	2.5481
51	H	-3.2546	6.7956	-0.3903
52	H	-3.6232	5.7789	1.0099
53	H	-3.2535	7.5011	1.2374
54	H	1.0183	7.2940	0.2128
55	H	-0.3053	7.8778	-0.8057
56	H	-0.1343	8.4808	0.8522
57	C	-1.6048	4.8356	3.3110
58	C	0.5660	5.8793	3.1030
59	C	-1.2554	4.3669	4.5780
60	H	-2.5833	4.5871	2.9103
61	C	0.9182	5.4038	4.3674
62	H	1.2856	6.4776	2.5489
63	C	0.0074	4.6461	5.1102
64	H	-1.9732	3.7904	5.1567
65	H	1.8988	5.6314	4.7773
66	H	0.2781	4.2805	6.0970

TpBr3CuNH_Si_t_v5
Energy (POTENTIAL) = -2487.67684814 Eh

	Atom	X	Y	Z
1	N	1.7569	0.3409	-0.0517
2	N	1.3129	1.5966	0.2693
3	N	-0.2365	-0.9797	0.7029
4	N	-0.9092	0.1051	1.1848
5	N	0.0653	-0.3564	-1.7855
6	N	-0.3634	0.9278	-1.9448
7	C	2.3777	2.2932	0.6657
8	C	3.5428	1.5050	0.6096
9	C	3.0949	0.2712	0.1474
10	C	-1.4151	-0.2387	2.3614
11	C	-1.0976	-1.5790	2.6718
12	C	-0.3415	-2.0066	1.5854
13	C	-0.7317	1.0808	-3.2165
14	C	-0.5598	-0.1273	-3.9193
15	C	-0.0500	-1.0098	-2.9655
16	B	0.7641	-0.7889	-0.4632
17	H	1.3547	-1.7949	-0.6506
18	N	-2.4999	2.6714	-0.4072
19	S	-4.0180	2.0433	-0.7266
20	O	-4.9736	2.7262	0.1708
21	O	-4.2812	2.0984	-2.1787
22	C	-3.9085	0.3298	-0.2494
23	C	-3.2579	-0.5675	-1.0981
24	C	-4.5111	-0.0987	0.9336
25	C	-3.1676	-1.9055	-0.7257
26	H	-2.8400	-0.2279	-2.0376
27	C	-4.4213	-1.4437	1.2846
28	H	-5.0343	0.6109	1.5634
29	C	-3.7370	-2.3620	0.4729
30	H	-2.6561	-2.6084	-1.3767
31	H	-4.8816	-1.7859	2.2074
32	Cu	-0.7698	1.9055	-0.1706
33	C	-3.6043	-3.8016	0.8964
34	H	-2.9048	-3.8914	1.7356
35	H	-4.5637	-4.2064	1.2354
36	H	-3.2312	-4.4294	0.0829
37	Br	4.1231	-1.2728	-0.1693
38	Br	5.3020	2.0027	1.0574
39	Br	2.2601	4.0861	1.2328
40	Br	-0.9144	-0.4697	-5.7351
41	Br	-1.3295	2.7422	-3.8600

42	Br	0.4133	-2.8172	-3.1948
43	Br	0.4081	-3.7080	1.2956
44	Br	-1.5957	-2.5626	4.1971
45	Br	-2.3623	1.0001	3.4196
46	H	-2.4592	3.5152	-0.9908
47	Si	-1.5406	5.1299	1.5827
48	C	-0.3762	6.2651	2.5587
49	C	-3.1186	4.7276	2.5414
50	C	-1.9203	5.8606	-0.1105
51	H	0.0143	5.7612	3.4494
52	H	0.4743	6.5941	1.9521
53	H	-0.9100	7.1662	2.8904
54	H	-3.7116	3.9740	2.0146
55	H	-2.8702	4.3426	3.5366
56	H	-3.7388	5.6239	2.6768
57	C	-0.9057	6.4531	-0.8929
58	C	-3.2020	5.7357	-0.6941
59	C	-1.1537	6.8922	-2.1946
60	H	0.0950	6.5720	-0.4836
61	C	-3.4512	6.1670	-1.9992
62	H	-4.0120	5.2783	-0.1320
63	C	-2.4272	6.7457	-2.7553
64	H	-0.3544	7.3483	-2.7729
65	H	-4.4447	6.0519	-2.4240
66	H	-2.6204	7.0832	-3.7698

TpBr3CuNH_Si_t_v6
Energy (POTENTIAL) = -2487.68042228 Eh

	Atom	X	Y	Z
1	Cu	-0.7372	-0.3937	-1.5495
2	N	1.2242	-1.1799	-2.4952
3	N	2.3481	-0.6578	-1.9266
4	N	0.1000	1.5091	-1.3895
5	N	1.4260	1.5594	-1.0660
6	N	0.1741	-0.8812	0.2683
7	N	1.4701	-0.4786	0.4288
8	B	2.2243	0.2757	-0.7039
9	C	1.6288	-2.0360	-3.4260
10	C	3.0362	-2.0909	-3.4892
11	C	3.4498	-1.1923	-2.5105
12	C	-0.3076	2.7639	-1.5911
13	C	0.7608	3.6626	-1.4158
14	C	1.8404	2.8485	-1.0774
15	C	-0.1685	-1.5530	1.3689
16	C	0.9070	-1.6025	2.2738
17	C	1.9271	-0.9069	1.6288
18	H	3.2984	0.5832	-0.3219
19	N	-1.9817	-0.7561	-2.9117
20	S	-3.6337	-0.5787	-3.0681
21	O	-4.1041	-1.6823	-3.9277
22	O	-3.9701	0.8013	-3.4681
23	C	-4.2241	-0.8490	-1.4043
24	C	-4.6819	-2.1152	-1.0431
25	C	-4.2598	0.2204	-0.5037
26	C	-5.1827	-2.3090	0.2455
27	H	-4.6584	-2.9293	-1.7594
28	C	-4.7570	0.0068	0.7775
29	H	-3.9163	1.2024	-0.8066
30	C	-5.2308	-1.2565	1.1710
31	H	-5.5488	-3.2910	0.5315
32	H	-4.7862	0.8326	1.4833
33	C	-5.7781	-1.4618	2.5607
34	H	-5.0681	-1.1119	3.3185
35	H	-6.7036	-0.8911	2.7024
36	H	-5.9983	-2.5147	2.7564
37	Br	-1.8915	-2.2775	1.5858
38	Br	-2.0864	3.1666	-2.0424
39	Br	0.3959	-3.0155	-4.4648
40	Br	4.1087	-3.1362	-4.6276
41	Br	5.2107	-0.7450	-2.0233

42	Br	3.6702	-0.5864	2.2595
43	Br	0.9555	-2.4181	3.9691
44	Br	0.7383	5.5341	-1.6064
45	Br	3.6008	3.3863	-0.6917
46	H	-1.5872	-0.7307	-3.8709
47	Si	-0.5414	0.5619	-5.8398
48	C	-1.5256	2.1682	-5.6854
49	C	-0.8932	-0.3423	-7.4623
50	C	1.2957	0.8302	-5.5717
51	H	-2.5695	1.9666	-5.4269
52	H	-1.1105	2.8155	-4.9083
53	H	-1.4997	2.7189	-6.6354
54	H	-0.3914	-1.3142	-7.5045
55	H	-1.9688	-0.5162	-7.5766
56	H	-0.5556	0.2507	-8.3230
57	C	1.7663	1.7485	-4.6080
58	C	2.2577	0.0995	-6.3007
59	C	3.1310	1.9407	-4.3970
60	H	1.0602	2.3240	-4.0164
61	C	3.6245	0.2922	-6.0930
62	H	1.9381	-0.6231	-7.0470
63	C	4.0662	1.2141	-5.1409
64	H	3.4685	2.6584	-3.6556
65	H	4.3449	-0.2775	-6.6728
66	H	5.1293	1.3654	-4.9779

TpBr3CuNH_Si_t_v1
Energy (POTENTIAL) = -2487.68183374 Eh

	Atom	X	Y	Z
1	Cu	-0.6558	-0.4463	-1.6269
2	N	1.3044	-1.3318	-2.5553
3	N	2.4433	-0.8851	-1.9534
4	N	0.3459	1.3750	-1.4474
5	N	1.6410	1.3481	-1.0198
6	N	0.2094	-1.0701	0.1689
7	N	1.5185	-0.7439	0.3863
8	B	2.3490	0.0088	-0.6950
9	C	1.6758	-2.2139	-3.4731
10	C	3.0783	-2.3602	-3.5002
11	C	3.5232	-1.4906	-2.5093
12	C	0.0168	2.6535	-1.6334
13	C	1.0993	3.4928	-1.3185
14	C	2.1118	2.6149	-0.9372
15	C	-0.2002	-1.7791	1.2220
16	C	0.8404	-1.9241	2.1566
17	C	1.9138	-1.2503	1.5776
18	H	3.4311	0.2301	-0.2770
19	N	-1.9247	-0.6687	-2.9975
20	S	-3.5685	-0.3994	-3.1148
21	O	-4.1217	-1.4763	-3.9587
22	O	-3.8358	0.9962	-3.5116
23	C	-4.1327	-0.6272	-1.4350
24	C	-4.7895	-1.8073	-1.0927
25	C	-3.9565	0.4031	-0.5042
26	C	-5.2800	-1.9539	0.2068
27	H	-4.9243	-2.5904	-1.8306
28	C	-4.4445	0.2351	0.7872
29	H	-3.4597	1.3229	-0.7915
30	C	-5.1181	-0.9406	1.1619
31	H	-5.8027	-2.8672	0.4771
32	H	-4.3108	1.0299	1.5159
33	C	-5.6364	-1.1016	2.5684
34	H	-4.8136	-1.0741	3.2928
35	H	-6.3195	-0.2861	2.8312
36	H	-6.1688	-2.0479	2.6960
37	Br	-1.9584	-2.4366	1.3371
38	Br	-1.6721	3.1336	-2.2907
39	Br	0.4024	-3.0995	-4.5467
40	Br	4.1142	-3.4395	-4.6431
41	Br	5.2985	-1.1412	-1.9924

42	Br	3.6476	-1.0433	2.2786
43	Br	0.7909	-2.8167	3.8125
44	Br	1.1805	5.3678	-1.4408
45	Br	3.8640	3.0500	-0.4142
46	H	-1.5547	-0.6819	-3.9688
47	Si	-0.6357	0.3468	-6.0778
48	C	-1.9160	1.7224	-6.2714
49	C	-0.4714	-0.7374	-7.6166
50	C	1.0216	1.0007	-5.4942
51	H	-2.8600	1.3109	-6.6439
52	H	-2.1280	2.2065	-5.3141
53	H	-1.5723	2.4855	-6.9825
54	H	0.2951	-1.5083	-7.4899
55	H	-1.4191	-1.2402	-7.8386
56	H	-0.1998	-0.1311	-8.4912
57	C	1.1281	2.2509	-4.8483
58	C	2.2023	0.2424	-5.6449
59	C	2.3558	2.7233	-4.3827
60	H	0.2436	2.8684	-4.7188
61	C	3.4293	0.7083	-5.1728
62	H	2.1648	-0.7243	-6.1398
63	C	3.5104	1.9509	-4.5372
64	H	2.4136	3.6970	-3.9055
65	H	4.3237	0.1060	-5.3047
66	H	4.4658	2.3162	-4.1717

TpBr3CuNH_Si_t_v2_SP
Energy (POTENTIAL) = -26981.8883126 Eh

	Atom	X	Y	Z
1	N	1.6215	0.2820	1.2964
2	N	1.3054	1.6135	1.2673
3	N	-0.6487	-0.7489	1.6269
4	N	-1.2317	0.4573	1.8813
5	N	0.3484	-0.4759	-0.7368
6	N	0.0065	0.7905	-1.1046
7	C	2.3421	2.2697	1.7850
8	C	3.3594	1.3773	2.1719
9	C	2.8521	0.1239	1.8401
10	C	-2.0817	0.2785	2.8841
11	C	-2.0945	-1.0703	3.2963
12	C	-1.1602	-1.6843	2.4667
13	C	0.0634	0.8629	-2.4325
14	C	0.4359	-0.3837	-2.9678
15	C	0.6076	-1.2015	-1.8517
16	B	0.6322	-0.7998	0.7566
17	H	1.1254	-1.8704	0.8359
18	N	-2.1352	3.0120	-0.2214
19	S	-3.6802	2.5189	-0.6641
20	O	-4.6072	2.8508	0.4312
21	O	-3.9680	3.0404	-2.0155
22	C	-3.5603	0.7388	-0.7736
23	C	-3.0521	0.1652	-1.9432
24	C	-3.9591	-0.0540	0.3013
25	C	-2.9004	-1.2154	-2.0090
26	H	-2.7901	0.7938	-2.7862
27	C	-3.8036	-1.4378	0.2175
28	H	-4.3880	0.4076	1.1827
29	C	-3.2555	-2.0368	-0.9252
30	H	-2.5058	-1.6670	-2.9147
31	H	-4.1128	-2.0601	1.0519
32	Cu	-0.6350	2.0394	0.4075
33	C	-3.0363	-3.5259	-0.9995
34	H	-3.5220	-4.0484	-0.1711
35	H	-3.4183	-3.9365	-1.9403
36	H	-1.9652	-3.7589	-0.9607
37	Br	3.6826	-1.5472	2.0842
38	Br	5.0223	1.7820	2.9561
39	Br	2.3722	4.1509	1.9280
40	Br	0.6372	-0.8429	-4.7815
41	Br	-0.3459	2.4914	-3.3248

42	Br	1.0843	-3.0199	-1.8149
43	Br	-0.6623	-3.4980	2.4283
44	Br	-3.1481	-1.8631	4.6400
45	Br	-3.0798	1.7179	3.5792
46	H	-1.9023	3.7657	-0.8757
47	Si	-1.2373	5.8508	-3.7322
48	C	-2.7628	6.2789	-4.7581
49	C	0.3174	6.7851	-4.2634
50	C	-1.5583	6.0313	-1.8929
51	H	-2.5469	6.1854	-5.8278
52	H	-3.6025	5.6161	-4.5256
53	H	-3.0834	7.3117	-4.5664
54	H	1.1984	6.4556	-3.7027
55	H	0.5203	6.6305	-5.3285
56	H	0.1942	7.8634	-4.0937
57	C	-2.8748	6.0875	-1.3842
58	C	-0.4943	6.0731	-0.9633
59	C	-3.1175	6.1925	-0.0130
60	H	-3.7204	6.0369	-2.0629
61	C	-0.7375	6.1818	0.4071
62	H	0.5345	6.0266	-1.3130
63	C	-2.0495	6.2418	0.8867
64	H	-4.1396	6.2250	0.3526
65	H	0.0951	6.2229	1.1031
66	H	-2.2379	6.3191	1.9537

TpBr3CuNH_Si_t_v3_SP
Energy (POTENTIAL) = -26981.89277 Eh

	Atom	X	Y	Z
1	N	2.1040	0.5010	0.0600
2	N	1.4978	1.7230	0.1432
3	N	0.1357	-0.9587	0.5796
4	N	-0.5831	0.1312	0.9793
5	N	0.7845	-0.5219	-1.8558
6	N	0.0335	0.5725	-2.1596
7	C	2.2887	2.5084	0.8693
8	C	3.4495	1.8173	1.2639
9	C	3.2814	0.5424	0.7302
10	C	-1.2171	-0.2092	2.0909
11	C	-0.9631	-1.5579	2.4286
12	C	-0.0878	-1.9890	1.4378
13	C	-0.3191	0.4693	-3.4425
14	C	0.1964	-0.7141	-3.9995
15	C	0.8948	-1.3102	-2.9509
16	B	1.3109	-0.7502	-0.4203
17	H	2.0173	-1.6972	-0.4104
18	N	-2.0488	2.8592	-0.8272
19	S	-3.6763	2.4318	-0.8847
20	O	-4.3841	3.1615	0.1833
21	O	-4.1445	2.6122	-2.2732
22	C	-3.6828	0.6903	-0.4913
23	C	-3.0572	-0.2130	-1.3557
24	C	-4.3473	0.2480	0.6497
25	C	-3.0307	-1.5623	-1.0283
26	H	-2.5952	0.1375	-2.2664
27	C	-4.3301	-1.1141	0.9547
28	H	-4.8509	0.9580	1.2945
29	C	-3.6534	-2.0323	0.1415
30	H	-2.5246	-2.2624	-1.6876
31	H	-4.8347	-1.4633	1.8507
32	Cu	-0.4328	1.8959	-0.6231
33	C	-3.5687	-3.4905	0.5099
34	H	-4.2292	-3.7364	1.3452
35	H	-3.8294	-4.1307	-0.3397
36	H	-2.5450	-3.7499	0.8051
37	Br	4.4580	-0.9165	0.8659
38	Br	4.9098	2.4776	2.2489
39	Br	1.8067	4.2778	1.2877
40	Br	0.0190	-1.3289	-5.7678
41	Br	-1.3882	1.7629	-4.2819

42	Br	1.8542	-2.9248	-2.9895
43	Br	0.6792	-3.6959	1.2300
44	Br	-1.6509	-2.5433	3.8778
45	Br	-2.1799	1.0705	3.0853
46	H	-1.9782	3.7079	-1.4006
47	Si	1.5916	4.1619	-2.9597
48	C	2.3467	5.7056	-2.1633
49	C	0.8926	4.6115	-4.6625
50	C	2.8218	2.7463	-3.0524
51	H	1.5682	6.4338	-1.9098
52	H	2.9034	5.4833	-1.2497
53	H	3.0352	6.1856	-2.8719
54	H	0.6855	3.7323	-5.2777
55	H	-0.0389	5.1809	-4.5704
56	H	1.6155	5.2389	-5.2012
57	C	3.8705	2.6246	-2.1158
58	C	2.6872	1.7195	-4.0110
59	C	4.7341	1.5291	-2.1291
60	H	4.0204	3.3963	-1.3659
61	C	3.5461	0.6200	-4.0250
62	H	1.9032	1.7769	-4.7611
63	C	4.5704	0.5171	-3.0794
64	H	5.5364	1.4640	-1.3996
65	H	3.4217	-0.1539	-4.7773
66	H	5.2382	-0.3396	-3.0872

TpBr3CuNH_Si_t_v4_SP
Energy (POTENTIAL) = -26981.8838146 Eh

	Atom	X	Y	Z
1	N	2.1864	-0.3035	0.1460
2	N	1.8776	1.0038	0.4069
3	N	0.0718	-1.4382	0.9225
4	N	-0.4137	-0.2969	1.4899
5	N	0.3841	-0.8095	-1.5374
6	N	-0.1142	0.4576	-1.5760
7	C	3.0154	1.6089	0.7269
8	C	4.1013	0.7110	0.6815
9	C	3.5199	-0.4962	0.3068
10	C	-0.9679	-0.6367	2.6498
11	C	-0.8841	-2.0288	2.8511
12	C	-0.2089	-2.4941	1.7254
13	C	-0.4164	0.7296	-2.8447
14	C	-0.1407	-0.3811	-3.6648
15	C	0.3716	-1.3340	-2.7860
16	B	1.0813	-1.3258	-0.2504
17	H	1.5634	-2.3840	-0.4608
18	N	-1.6711	2.8278	0.2851
19	S	-3.3386	2.7293	0.1271
20	O	-3.9523	3.2654	1.3579
21	O	-3.7262	3.3292	-1.1621
22	C	-3.6007	0.9655	0.0717
23	C	-3.3537	0.2796	-1.1203
24	C	-3.9652	0.2876	1.2342
25	C	-3.4049	-1.1107	-1.1222
26	H	-3.1183	0.8271	-2.0240
27	C	-4.0231	-1.1051	1.2113
28	H	-4.1820	0.8408	2.1394
29	C	-3.7199	-1.8248	0.0460
30	H	-3.1951	-1.6511	-2.0413
31	H	-4.2941	-1.6410	2.1164
32	Cu	-0.3939	1.4417	0.2418
33	C	-3.7597	-3.3310	0.0342
34	H	-3.6098	-3.7442	1.0356
35	H	-4.7314	-3.6894	-0.3282
36	H	-2.9939	-3.7434	-0.6299
37	Br	4.3795	-2.1526	0.0479
38	Br	5.9167	1.0611	1.0427
39	Br	3.0600	3.4400	1.1855
40	Br	-0.4042	-0.5444	-5.5209
41	Br	-1.1078	2.4028	-3.3515

42	Br	0.9527	-3.0767	-3.1882
43	Br	0.2570	-4.2678	1.3082
44	Br	-1.5441	-3.0291	4.3018
45	Br	-1.6551	0.6666	3.8207
46	H	-1.4020	3.8232	0.1597
47	Si	-1.1599	6.2024	0.8288
48	C	-2.9979	6.6041	0.6572
49	C	-0.0356	7.5907	0.2164
50	C	-0.7043	5.6117	2.5481
51	H	-3.2546	6.7956	-0.3903
52	H	-3.6232	5.7789	1.0099
53	H	-3.2535	7.5011	1.2374
54	H	1.0183	7.2940	0.2128
55	H	-0.3053	7.8778	-0.8057
56	H	-0.1343	8.4808	0.8522
57	C	-1.6048	4.8356	3.3110
58	C	0.5660	5.8793	3.1030
59	C	-1.2554	4.3669	4.5780
60	H	-2.5833	4.5871	2.9103
61	C	0.9182	5.4038	4.3674
62	H	1.2856	6.4776	2.5489
63	C	0.0074	4.6461	5.1102
64	H	-1.9732	3.7904	5.1567
65	H	1.8988	5.6314	4.7773
66	H	0.2781	4.2805	6.0970

TpBr3CuNH_Si_t_v5_SP
Energy (POTENTIAL) = -26981.888909 Eh

	Atom	X	Y	Z
1	N	1.7569	0.3409	-0.0517
2	N	1.3129	1.5966	0.2693
3	N	-0.2365	-0.9797	0.7029
4	N	-0.9092	0.1051	1.1848
5	N	0.0653	-0.3564	-1.7855
6	N	-0.3634	0.9278	-1.9448
7	C	2.3777	2.2932	0.6657
8	C	3.5428	1.5050	0.6096
9	C	3.0948	0.2712	0.1474
10	C	-1.4151	-0.2387	2.3614
11	C	-1.0976	-1.5790	2.6718
12	C	-0.3415	-2.0066	1.5854
13	C	-0.7317	1.0808	-3.2165
14	C	-0.5598	-0.1273	-3.9193
15	C	-0.0500	-1.0098	-2.9655
16	B	0.7641	-0.7889	-0.4632
17	H	1.3547	-1.7949	-0.6506
18	N	-2.4999	2.6714	-0.4072
19	S	-4.0180	2.0433	-0.7266
20	O	-4.9736	2.7262	0.1708
21	O	-4.2812	2.0984	-2.1787
22	C	-3.9085	0.3298	-0.2494
23	C	-3.2579	-0.5675	-1.0981
24	C	-4.5111	-0.0987	0.9336
25	C	-3.1676	-1.9056	-0.7257
26	H	-2.8400	-0.2279	-2.0376
27	C	-4.4213	-1.4437	1.2846
28	H	-5.0343	0.6109	1.5634
29	C	-3.7370	-2.3620	0.4729
30	H	-2.6561	-2.6084	-1.3767
31	H	-4.8816	-1.7859	2.2074
32	Cu	-0.7698	1.9055	-0.1706
33	C	-3.6043	-3.8016	0.8964
34	H	-2.9048	-3.8914	1.7356
35	H	-4.5637	-4.2064	1.2354
36	H	-3.2312	-4.4294	0.0829
37	Br	4.1231	-1.2728	-0.1693
38	Br	5.3020	2.0027	1.0574
39	Br	2.2601	4.0861	1.2328
40	Br	-0.9144	-0.4697	-5.7351
41	Br	-1.3295	2.7422	-3.8600

42	Br	0.4133	-2.8172	-3.1948
43	Br	0.4081	-3.7080	1.2956
44	Br	-1.5957	-2.5626	4.1970
45	Br	-2.3623	1.0000	3.4196
46	H	-2.4592	3.5152	-0.9908
47	Si	-1.5406	5.1299	1.5827
48	C	-0.3762	6.2651	2.5587
49	C	-3.1186	4.7276	2.5414
50	C	-1.9203	5.8606	-0.1105
51	H	0.0143	5.7612	3.4494
52	H	0.4743	6.5941	1.9521
53	H	-0.9100	7.1662	2.8904
54	H	-3.7116	3.9740	2.0146
55	H	-2.8702	4.3426	3.5366
56	H	-3.7388	5.6239	2.6768
57	C	-0.9057	6.4531	-0.8929
58	C	-3.2020	5.7357	-0.6941
59	C	-1.1537	6.8922	-2.1946
60	H	0.0950	6.5720	-0.4836
61	C	-3.4512	6.1670	-1.9992
62	H	-4.0120	5.2783	-0.1320
63	C	-2.4272	6.7457	-2.7553
64	H	-0.3544	7.3483	-2.7729
65	H	-4.4447	6.0519	-2.4240
66	H	-2.6204	7.0832	-3.7698

TpBr3CuNH_Si_t_v6_SP
Energy (POTENTIAL) = -26981.8935282 Eh

	Atom	X	Y	Z
1	Cu	-0.7372	-0.3937	-1.5495
2	N	1.2242	-1.1799	-2.4952
3	N	2.3481	-0.6578	-1.9266
4	N	0.1000	1.5091	-1.3895
5	N	1.4260	1.5594	-1.0660
6	N	0.1741	-0.8812	0.2683
7	N	1.4701	-0.4786	0.4288
8	B	2.2243	0.2757	-0.7039
9	C	1.6288	-2.0360	-3.4260
10	C	3.0362	-2.0909	-3.4892
11	C	3.4498	-1.1923	-2.5104
12	C	-0.3076	2.7639	-1.5911
13	C	0.7608	3.6626	-1.4158
14	C	1.8404	2.8485	-1.0774
15	C	-0.1685	-1.5530	1.3689
16	C	0.9070	-1.6025	2.2738
17	C	1.9271	-0.9069	1.6288
18	H	3.2984	0.5832	-0.3219
19	N	-1.9817	-0.7561	-2.9117
20	S	-3.6337	-0.5787	-3.0681
21	O	-4.1041	-1.6823	-3.9277
22	O	-3.9701	0.8013	-3.4681
23	C	-4.2241	-0.8490	-1.4043
24	C	-4.6819	-2.1152	-1.0431
25	C	-4.2598	0.2204	-0.5037
26	C	-5.1827	-2.3090	0.2455
27	H	-4.6584	-2.9293	-1.7594
28	C	-4.7570	0.0068	0.7775
29	H	-3.9163	1.2024	-0.8066
30	C	-5.2308	-1.2565	1.1710
31	H	-5.5488	-3.2910	0.5315
32	H	-4.7862	0.8326	1.4833
33	C	-5.7781	-1.4618	2.5607
34	H	-5.0681	-1.1119	3.3185
35	H	-6.7036	-0.8911	2.7024
36	H	-5.9983	-2.5147	2.7564
37	Br	-1.8915	-2.2775	1.5858
38	Br	-2.0864	3.1666	-2.0424
39	Br	0.3959	-3.0155	-4.4648
40	Br	4.1087	-3.1362	-4.6276
41	Br	5.2107	-0.7450	-2.0233

42	Br	3.6702	-0.5864	2.2595
43	Br	0.9555	-2.4181	3.9691
44	Br	0.7383	5.5341	-1.6064
45	Br	3.6008	3.3863	-0.6917
46	H	-1.5872	-0.7307	-3.8709
47	Si	-0.5414	0.5619	-5.8398
48	C	-1.5256	2.1682	-5.6854
49	C	-0.8932	-0.3423	-7.4623
50	C	1.2957	0.8302	-5.5717
51	H	-2.5695	1.9666	-5.4269
52	H	-1.1105	2.8155	-4.9083
53	H	-1.4997	2.7189	-6.6354
54	H	-0.3914	-1.3142	-7.5045
55	H	-1.9688	-0.5162	-7.5766
56	H	-0.5556	0.2507	-8.3230
57	C	1.7663	1.7485	-4.6080
58	C	2.2577	0.0995	-6.3007
59	C	3.1310	1.9407	-4.3970
60	H	1.0602	2.3240	-4.0164
61	C	3.6245	0.2922	-6.0930
62	H	1.9381	-0.6231	-7.0470
63	C	4.0662	1.2141	-5.1409
64	H	3.4685	2.6584	-3.6556
65	H	4.3449	-0.2775	-6.6728
66	H	5.1293	1.3654	-4.9779

TpBr3CuNH_Si_t_v1_SP
Energy (POTENTIAL) = -26981.8945625 Eh

	Atom	X	Y	Z
1	Cu	-0.6558	-0.4463	-1.6269
2	N	1.3044	-1.3318	-2.5553
3	N	2.4433	-0.8851	-1.9534
4	N	0.3459	1.3750	-1.4474
5	N	1.6410	1.3481	-1.0198
6	N	0.2094	-1.0701	0.1689
7	N	1.5185	-0.7439	0.3863
8	B	2.3490	0.0088	-0.6950
9	C	1.6758	-2.2138	-3.4731
10	C	3.0783	-2.3602	-3.5002
11	C	3.5232	-1.4906	-2.5093
12	C	0.0168	2.6535	-1.6334
13	C	1.0993	3.4928	-1.3185
14	C	2.1118	2.6149	-0.9372
15	C	-0.2002	-1.7791	1.2220
16	C	0.8404	-1.9241	2.1566
17	C	1.9138	-1.2503	1.5776
18	H	3.4311	0.2301	-0.2770
19	N	-1.9247	-0.6687	-2.9975
20	S	-3.5685	-0.3994	-3.1148
21	O	-4.1217	-1.4763	-3.9587
22	O	-3.8358	0.9962	-3.5116
23	C	-4.1327	-0.6272	-1.4350
24	C	-4.7895	-1.8073	-1.0927
25	C	-3.9565	0.4031	-0.5042
26	C	-5.2800	-1.9539	0.2068
27	H	-4.9243	-2.5904	-1.8306
28	C	-4.4445	0.2351	0.7872
29	H	-3.4597	1.3229	-0.7915
30	C	-5.1181	-0.9406	1.1619
31	H	-5.8027	-2.8672	0.4771
32	H	-4.3108	1.0299	1.5159
33	C	-5.6364	-1.1016	2.5684
34	H	-4.8136	-1.0741	3.2928
35	H	-6.3194	-0.2861	2.8312
36	H	-6.1688	-2.0479	2.6960
37	Br	-1.9584	-2.4366	1.3371
38	Br	-1.6721	3.1336	-2.2907
39	Br	0.4024	-3.0995	-4.5467
40	Br	4.1142	-3.4395	-4.6431
41	Br	5.2985	-1.1412	-1.9924

42	Br	3.6476	-1.0433	2.2786
43	Br	0.7909	-2.8167	3.8125
44	Br	1.1805	5.3678	-1.4408
45	Br	3.8640	3.0500	-0.4142
46	H	-1.5547	-0.6819	-3.9688
47	Si	-0.6357	0.3468	-6.0778
48	C	-1.9160	1.7224	-6.2714
49	C	-0.4714	-0.7374	-7.6166
50	C	1.0216	1.0007	-5.4942
51	H	-2.8600	1.3109	-6.6439
52	H	-2.1280	2.2065	-5.3141
53	H	-1.5723	2.4855	-6.9825
54	H	0.2951	-1.5083	-7.4899
55	H	-1.4191	-1.2402	-7.8386
56	H	-0.1998	-0.1311	-8.4912
57	C	1.1281	2.2509	-4.8483
58	C	2.2023	0.2424	-5.6449
59	C	2.3558	2.7233	-4.3827
60	H	0.2436	2.8684	-4.7188
61	C	3.4293	0.7083	-5.1728
62	H	2.1648	-0.7243	-6.1398
63	C	3.5104	1.9509	-4.5372
64	H	2.4136	3.6970	-3.9055
65	H	4.3237	0.1060	-5.3047
66	H	4.4658	2.3162	-4.1717

TpBr3CuN_SiH_s_v1
Energy (POTENTIAL) = -2487.64205569 Eh

	Atom	X	Y	Z
1	Cu	-0.0255	-1.2124	-1.7201
2	N	1.7227	-1.3632	-2.7511
3	N	2.8187	-0.6805	-2.3126
4	N	0.2852	0.7618	-1.2603
5	N	1.5684	1.2094	-1.1619
6	N	1.3998	-1.5282	0.1895
7	N	2.4839	-0.7026	0.1899
8	B	2.7510	0.1979	-1.0399
9	C	2.0937	-2.0661	-3.8211
10	C	3.4561	-1.8573	-4.1000
11	C	3.8736	-0.9682	-3.1115
12	C	-0.5236	1.8158	-1.1423
13	C	0.2316	2.9915	-0.9769
14	C	1.5560	2.5526	-0.9902
15	C	1.3527	-2.0992	1.3841
16	C	2.4155	-1.6597	2.2024
17	C	3.1084	-0.7638	1.3929
18	H	3.7629	0.7925	-0.9064
19	N	-1.6589	-1.8840	-1.3116
20	S	-3.0097	-1.9838	-2.2023
21	O	-2.9592	-3.4815	-2.2408
22	O	-3.1003	-1.2655	-3.4792
23	C	-4.3201	-1.4012	-1.1556
24	C	-4.3556	-1.8086	0.1846
25	C	-5.2671	-0.5118	-1.6719
26	C	-5.3575	-1.3117	1.0101
27	H	-3.6007	-2.4834	0.5729
28	C	-6.2605	-0.0234	-0.8263
29	H	-5.2181	-0.2053	-2.7107
30	C	-6.3218	-0.4116	0.5223
31	H	-5.3899	-1.6167	2.0522
32	H	-6.9989	0.6703	-1.2180
33	C	-7.4107	0.1034	1.4257
34	H	-7.8485	1.0288	1.0416
35	H	-8.2174	-0.6360	1.5091
36	H	-7.0349	0.2874	2.4369
37	Br	-0.0211	-3.3201	1.8130
38	Br	-2.3896	1.6172	-1.1450
39	Br	0.8742	-3.1502	-4.7560
40	Br	4.4793	-2.6099	-5.4849
41	Br	5.5978	-0.2691	-2.8513

42	Br	4.6520	0.2294	1.8165
43	Br	2.8131	-2.1693	3.9722
44	Br	-0.4048	4.7515	-0.7867
45	Br	3.1102	3.5966	-0.8269
46	H	-0.7329	-0.1394	-3.8802
47	Si	-0.6465	0.6878	-5.1038
48	C	-1.5917	2.2912	-4.7958
49	C	-1.3799	-0.2828	-6.5395
50	C	1.1795	1.0685	-5.3962
51	H	-2.6400	2.0826	-4.5558
52	H	-1.1626	2.8503	-3.9574
53	H	-1.5680	2.9427	-5.6773
54	H	-0.8560	-1.2327	-6.6889
55	H	-2.4294	-0.5166	-6.3287
56	H	-1.3402	0.2799	-7.4791
57	C	1.8651	1.9385	-4.5258
58	C	1.9115	0.4874	-6.4469
59	C	3.2250	2.2075	-4.6879
60	H	1.3291	2.4166	-3.7098
61	C	3.2720	0.7587	-6.6208
62	H	1.4190	-0.1878	-7.1424
63	C	3.9323	1.6161	-5.7389
64	H	3.7332	2.8751	-3.9976
65	H	3.8154	0.2976	-7.4406
66	H	4.9907	1.8235	-5.8684

TpBr3CuN_SiH_s_v2
Energy (POTENTIAL) = -2487.63721882 Eh

	Atom	X	Y	Z
1	Cu	-0.8904	-0.3094	-2.0037
2	N	0.7400	-1.4200	-2.5100
3	N	1.9481	-1.0874	-1.9683
4	N	0.2219	1.4442	-1.7575
5	N	1.5160	1.3295	-1.3536
6	N	-0.1516	-0.7231	0.2036
7	N	1.1831	-0.4819	0.3659
8	B	2.0495	-0.0354	-0.8313
9	C	0.9668	-2.3095	-3.4796
10	C	2.3456	-2.5659	-3.5931
11	C	2.9269	-1.7623	-2.6137
12	C	0.0199	2.7165	-2.1054
13	C	1.2041	3.4596	-1.9454
14	C	2.1281	2.5306	-1.4664
15	C	-0.6334	-1.0243	1.4039
16	C	0.3798	-0.9849	2.3839
17	C	1.5213	-0.6331	1.6712
18	H	3.1773	0.0821	-0.5007
19	N	-2.5531	0.0260	-2.6343
20	S	-4.1529	0.1161	-2.5392
21	O	-4.3644	-0.8626	-3.6502
22	O	-4.6514	1.4925	-2.6535
23	C	-4.8529	-0.5855	-1.0620
24	C	-4.9325	-1.9768	-0.9383
25	C	-5.3034	0.2641	-0.0490
26	C	-5.4818	-2.5156	0.2206
27	H	-4.5803	-2.6220	-1.7358
28	C	-5.8421	-0.2965	1.1071
29	H	-5.2414	1.3403	-0.1668
30	C	-5.9436	-1.6883	1.2603
31	H	-5.5540	-3.5947	0.3238
32	H	-6.1930	0.3567	1.9009
33	C	-6.5572	-2.2888	2.4989
34	H	-6.5582	-1.5788	3.3305
35	H	-7.5978	-2.5795	2.3082
36	H	-6.0217	-3.1911	2.8109
37	Br	-2.4574	-1.4306	1.6399
38	Br	-1.6678	3.3060	-2.6812
39	Br	-0.4232	-3.0572	-4.4958
40	Br	3.2068	-3.7259	-4.7971
41	Br	4.7558	-1.5755	-2.2248

42	Br	3.2665	-0.3903	2.3380
43	Br	0.2262	-1.3281	4.2297
44	Br	1.4768	5.2877	-2.2949
45	Br	3.9380	2.8163	-1.0496
46	H	2.2003	0.7756	-3.8093
47	Si	2.2585	1.0477	-5.2668
48	C	3.7879	0.2169	-5.9875
49	C	2.3260	2.9200	-5.4930
50	C	0.6847	0.3372	-6.0228
51	H	4.6918	0.5703	-5.4779
52	H	3.7450	-0.8714	-5.8730
53	H	3.9031	0.4375	-7.0549
54	H	1.4290	3.3985	-5.0851
55	H	3.1965	3.3467	-4.9812
56	H	2.3920	3.1893	-6.5536
57	C	-0.5569	0.5374	-5.3902
58	C	0.6948	-0.3883	-7.2281
59	C	-1.7417	0.0411	-5.9351
60	H	-0.6077	1.0769	-4.4485
61	C	-0.4884	-0.8836	-7.7843
62	H	1.6353	-0.5747	-7.7417
63	C	-1.7087	-0.6674	-7.1395
64	H	-2.6780	0.1819	-5.4052
65	H	-0.4567	-1.4395	-8.7180
66	H	-2.6288	-1.0565	-7.5677

TpBr3CuN_SiH_s_v1_SP
Energy (POTENTIAL) = -26981.8584555 Eh

	Atom	X	Y	Z
1	Cu	-0.0255	-1.2124	-1.7201
2	N	1.7227	-1.3632	-2.7511
3	N	2.8187	-0.6805	-2.3126
4	N	0.2852	0.7618	-1.2603
5	N	1.5684	1.2094	-1.1619
6	N	1.3998	-1.5282	0.1895
7	N	2.4839	-0.7026	0.1899
8	B	2.7510	0.1979	-1.0399
9	C	2.0937	-2.0661	-3.8211
10	C	3.4561	-1.8573	-4.1000
11	C	3.8736	-0.9682	-3.1115
12	C	-0.5236	1.8158	-1.1423
13	C	0.2316	2.9915	-0.9769
14	C	1.5560	2.5526	-0.9902
15	C	1.3527	-2.0992	1.3841
16	C	2.4155	-1.6597	2.2024
17	C	3.1084	-0.7638	1.3929
18	H	3.7629	0.7925	-0.9064
19	N	-1.6589	-1.8840	-1.3116
20	S	-3.0098	-1.9838	-2.2023
21	O	-2.9592	-3.4815	-2.2408
22	O	-3.1003	-1.2655	-3.4792
23	C	-4.3201	-1.4012	-1.1556
24	C	-4.3556	-1.8086	0.1846
25	C	-5.2671	-0.5118	-1.6719
26	C	-5.3575	-1.3117	1.0101
27	H	-3.6007	-2.4834	0.5729
28	C	-6.2605	-0.0234	-0.8263
29	H	-5.2181	-0.2053	-2.7107
30	C	-6.3218	-0.4116	0.5223
31	H	-5.3899	-1.6167	2.0522
32	H	-6.9989	0.6703	-1.2180
33	C	-7.4107	0.1034	1.4257
34	H	-7.8485	1.0288	1.0416
35	H	-8.2174	-0.6360	1.5091
36	H	-7.0349	0.2874	2.4369
37	Br	-0.0211	-3.3201	1.8130
38	Br	-2.3896	1.6172	-1.1450
39	Br	0.8742	-3.1502	-4.7560
40	Br	4.4793	-2.6099	-5.4849
41	Br	5.5978	-0.2691	-2.8513

42	Br	4.6520	0.2294	1.8165
43	Br	2.8131	-2.1693	3.9722
44	Br	-0.4048	4.7515	-0.7867
45	Br	3.1102	3.5966	-0.8269
46	H	-0.7329	-0.1394	-3.8802
47	Si	-0.6465	0.6878	-5.1038
48	C	-1.5917	2.2912	-4.7958
49	C	-1.3799	-0.2828	-6.5395
50	C	1.1795	1.0685	-5.3962
51	H	-2.6400	2.0826	-4.5558
52	H	-1.1626	2.8503	-3.9574
53	H	-1.5680	2.9427	-5.6773
54	H	-0.8560	-1.2327	-6.6889
55	H	-2.4294	-0.5166	-6.3287
56	H	-1.3402	0.2799	-7.4791
57	C	1.8651	1.9386	-4.5258
58	C	1.9115	0.4874	-6.4469
59	C	3.2250	2.2075	-4.6879
60	H	1.3291	2.4166	-3.7098
61	C	3.2720	0.7587	-6.6208
62	H	1.4190	-0.1878	-7.1424
63	C	3.9323	1.6161	-5.7389
64	H	3.7332	2.8751	-3.9976
65	H	3.8154	0.2976	-7.4406
66	H	4.9907	1.8235	-5.8684

TpBr3CuN_SiH_s_v2_SP
Energy (POTENTIAL) = -26981.8544274 Eh

	Atom	X	Y	Z
1	Cu	-0.8904	-0.3094	-2.0037
2	N	0.7400	-1.4200	-2.5100
3	N	1.9481	-1.0874	-1.9683
4	N	0.2219	1.4442	-1.7575
5	N	1.5160	1.3295	-1.3536
6	N	-0.1516	-0.7231	0.2036
7	N	1.1831	-0.4819	0.3659
8	B	2.0495	-0.0354	-0.8313
9	C	0.9668	-2.3095	-3.4796
10	C	2.3456	-2.5659	-3.5931
11	C	2.9269	-1.7623	-2.6137
12	C	0.0199	2.7165	-2.1054
13	C	1.2041	3.4596	-1.9454
14	C	2.1281	2.5306	-1.4664
15	C	-0.6334	-1.0243	1.4039
16	C	0.3798	-0.9849	2.3839
17	C	1.5213	-0.6331	1.6712
18	H	3.1773	0.0821	-0.5007
19	N	-2.5531	0.0260	-2.6343
20	S	-4.1529	0.1161	-2.5392
21	O	-4.3644	-0.8626	-3.6502
22	O	-4.6514	1.4925	-2.6535
23	C	-4.8529	-0.5855	-1.0620
24	C	-4.9325	-1.9768	-0.9383
25	C	-5.3034	0.2641	-0.0490
26	C	-5.4818	-2.5156	0.2206
27	H	-4.5803	-2.6220	-1.7358
28	C	-5.8421	-0.2965	1.1071
29	H	-5.2414	1.3403	-0.1668
30	C	-5.9436	-1.6883	1.2603
31	H	-5.5540	-3.5947	0.3238
32	H	-6.1930	0.3567	1.9009
33	C	-6.5572	-2.2888	2.4989
34	H	-6.5582	-1.5788	3.3305
35	H	-7.5978	-2.5795	2.3082
36	H	-6.0217	-3.1911	2.8109
37	Br	-2.4574	-1.4306	1.6399
38	Br	-1.6678	3.3060	-2.6812
39	Br	-0.4232	-3.0572	-4.4958
40	Br	3.2068	-3.7259	-4.7971
41	Br	4.7558	-1.5756	-2.2248

42	Br	3.2665	-0.3903	2.3380
43	Br	0.2262	-1.3281	4.2297
44	Br	1.4768	5.2877	-2.2949
45	Br	3.9380	2.8163	-1.0496
46	H	2.2003	0.7756	-3.8093
47	Si	2.2585	1.0477	-5.2668
48	C	3.7879	0.2169	-5.9875
49	C	2.3260	2.9200	-5.4930
50	C	0.6847	0.3372	-6.0228
51	H	4.6918	0.5703	-5.4779
52	H	3.7450	-0.8714	-5.8730
53	H	3.9031	0.4375	-7.0549
54	H	1.4290	3.3985	-5.0851
55	H	3.1965	3.3467	-4.9812
56	H	2.3920	3.1893	-6.5536
57	C	-0.5569	0.5374	-5.3902
58	C	0.6948	-0.3883	-7.2281
59	C	-1.7417	0.0411	-5.9351
60	H	-0.6077	1.0769	-4.4485
61	C	-0.4884	-0.8836	-7.7843
62	H	1.6353	-0.5747	-7.7417
63	C	-1.7087	-0.6674	-7.1395
64	H	-2.6780	0.1819	-5.4052
65	H	-0.4567	-1.4395	-8.7180
66	H	-2.6288	-1.0565	-7.5677

TpBr3CuN_SiH_t_v1
Energy (POTENTIAL) = -2487.66581063 Eh

	Atom	X	Y	Z
1	Cu	-0.6163	-0.4114	-1.8718
2	N	1.2210	-1.0819	-2.6464
3	N	2.3584	-0.6256	-2.0479
4	N	0.1840	1.5490	-1.6880
5	N	1.4909	1.6501	-1.3168
6	N	0.2401	-0.7701	0.0886
7	N	1.4988	-0.2896	0.2959
8	B	2.2739	0.3825	-0.8739
9	C	1.5868	-1.9783	-3.5584
10	C	2.9856	-2.1339	-3.5701
11	C	3.4319	-1.2488	-2.5908
12	C	-0.2643	2.7803	-1.9173
13	C	0.7622	3.7239	-1.7119
14	C	1.8591	2.9541	-1.3255
15	C	-0.1864	-1.2734	1.2426
16	C	0.7967	-1.1306	2.2409
17	C	1.8520	-0.4968	1.5869
18	H	3.3599	0.6856	-0.5204
19	N	-2.3272	-0.7543	-2.3572
20	S	-3.9408	-0.8178	-2.7588
21	O	-4.1319	-1.9746	-3.6469
22	O	-4.3446	0.5219	-3.2124
23	C	-4.6342	-1.1565	-1.1645
24	C	-4.8022	-2.4855	-0.7609
25	C	-4.9255	-0.0858	-0.3081
26	C	-5.2833	-2.7383	0.5210
27	H	-4.5647	-3.2992	-1.4375
28	C	-5.4061	-0.3615	0.9655
29	H	-4.7854	0.9366	-0.6419
30	C	-5.5912	-1.6864	1.4013
31	H	-5.4270	-3.7655	0.8433
32	H	-5.6425	0.4589	1.6370
33	C	-6.1320	-1.9631	2.7787
34	H	-5.6819	-1.2965	3.5214
35	H	-7.2151	-1.7892	2.8042
36	H	-5.9531	-2.9980	3.0823
37	Br	-1.9058	-2.0333	1.3787
38	Br	-2.0542	3.0692	-2.4134
39	Br	0.3099	-2.8478	-4.6324
40	Br	4.0128	-3.2840	-4.6456
41	Br	5.2038	-0.9283	-2.0514

42	Br	3.5150	0.0148	2.3047
43	Br	0.7080	-1.6707	4.0426
44	Br	0.6750	5.5927	-1.9212
45	Br	3.5921	3.5493	-0.9007
46	H	-0.8320	0.5867	-4.3319
47	Si	-0.2812	1.0720	-5.6257
48	C	-0.7307	2.8923	-5.8383
49	C	-1.0058	0.0432	-7.0274
50	C	1.5999	0.9150	-5.5711
51	H	-1.8150	3.0436	-5.8024
52	H	-0.2796	3.5106	-5.0552
53	H	-0.3694	3.2640	-6.8048
54	H	-0.7203	-1.0105	-6.9474
55	H	-2.1009	0.0909	-7.0046
56	H	-0.6767	0.4105	-8.0063
57	C	2.3448	1.6998	-4.6694
58	C	2.3101	0.0501	-6.4224
59	C	3.7367	1.6171	-4.6095
60	H	1.8324	2.3925	-4.0077
61	C	3.7052	-0.0298	-6.3762
62	H	1.7740	-0.5715	-7.1351
63	C	4.4212	0.7503	-5.4664
64	H	4.2873	2.2273	-3.8989
65	H	4.2310	-0.7038	-7.0464
66	H	5.5047	0.6841	-5.4242

TpBr3CuN_SiH_t_v2
Energy (POTENTIAL) = -2487.66551060 Eh

	Atom	X	Y	Z
1	Cu	-0.8858	-0.4077	-1.7507
2	N	0.8269	-1.3846	-2.5228
3	N	2.0367	-1.0428	-1.9969
4	N	0.2121	1.4177	-1.6645
5	N	1.5171	1.3294	-1.2832
6	N	-0.0950	-0.7921	0.1896
7	N	1.2418	-0.5727	0.3549
8	B	2.1043	-0.0345	-0.8216
9	C	1.0501	-2.2393	-3.5175
10	C	2.4328	-2.4636	-3.6708
11	C	3.0185	-1.6772	-2.6796
12	C	-0.0223	2.6892	-1.9809
13	C	1.1446	3.4621	-1.8296
14	C	2.0986	2.5479	-1.3838
15	C	-0.5688	-1.2449	1.3468
16	C	0.4602	-1.3347	2.3032
17	C	1.5940	-0.8942	1.6231
18	H	3.2238	0.1067	-0.4711
19	N	-2.5495	-0.6923	-2.3916
20	S	-4.1146	-0.9677	-2.8817
21	O	-4.1576	-2.2492	-3.6002
22	O	-4.5730	0.2601	-3.5530
23	C	-4.9014	-1.1283	-1.3024
24	C	-5.1125	-2.4036	-0.7698
25	C	-5.2229	0.0326	-0.5842
26	C	-5.6711	-2.5121	0.5014
27	H	-4.8491	-3.2869	-1.3412
28	C	-5.7745	-0.0996	0.6833
29	H	-5.0476	1.0124	-1.0156
30	C	-6.0070	-1.3686	1.2466
31	H	-5.8497	-3.4971	0.9226
32	H	-6.0304	0.7909	1.2501
33	C	-6.6266	-1.4882	2.6134
34	H	-6.1745	-0.7802	3.3156
35	H	-7.6980	-1.2557	2.5673
36	H	-6.5204	-2.4982	3.0176
37	Br	-2.3948	-1.6669	1.5398
38	Br	-1.7352	3.2486	-2.5308
39	Br	-0.3724	-2.9832	-4.4903
40	Br	3.2890	-3.5746	-4.9255
41	Br	4.8491	-1.4550	-2.3137

42	Br	3.3475	-0.7412	2.2895
43	Br	0.3334	-1.9158	4.0895
44	Br	1.3638	5.3034	-2.1479
45	Br	3.9109	2.8694	-1.0012
46	H	2.2799	0.8781	-3.7761
47	Si	2.2786	1.1713	-5.2304
48	C	3.7785	0.3614	-6.0324
49	C	2.3334	3.0478	-5.4290
50	C	0.6719	0.4790	-5.9347
51	H	4.7025	0.7122	-5.5584
52	H	3.7471	-0.7287	-5.9355
53	H	3.8432	0.6014	-7.1000
54	H	1.4410	3.5154	-4.9990
55	H	3.2094	3.4704	-4.9231
56	H	2.3818	3.3347	-6.4860
57	C	-0.5371	0.6677	-5.2391
58	C	0.6152	-0.1919	-7.1694
59	C	-1.7561	0.2293	-5.7569
60	H	-0.5292	1.1571	-4.2705
61	C	-0.6005	-0.6452	-7.6916
62	H	1.5279	-0.3672	-7.7346
63	C	-1.7888	-0.4294	-6.9896
64	H	-2.6730	0.3956	-5.1993
65	H	-0.6198	-1.1620	-8.6477
66	H	-2.7349	-0.7765	-7.3962

TpBr3CuN_SiH_t_v1_SP
Energy (POTENTIAL) = -26981.8816481 Eh

	Atom	X	Y	Z
1	Cu	-0.6163	-0.4114	-1.8718
2	N	1.2210	-1.0819	-2.6464
3	N	2.3584	-0.6256	-2.0479
4	N	0.1840	1.5490	-1.6880
5	N	1.4909	1.6501	-1.3168
6	N	0.2401	-0.7701	0.0886
7	N	1.4988	-0.2896	0.2959
8	B	2.2739	0.3825	-0.8739
9	C	1.5868	-1.9783	-3.5584
10	C	2.9856	-2.1339	-3.5701
11	C	3.4319	-1.2488	-2.5908
12	C	-0.2643	2.7803	-1.9173
13	C	0.7622	3.7239	-1.7119
14	C	1.8591	2.9541	-1.3256
15	C	-0.1864	-1.2734	1.2426
16	C	0.7967	-1.1306	2.2409
17	C	1.8520	-0.4968	1.5869
18	H	3.3599	0.6856	-0.5204
19	N	-2.3272	-0.7543	-2.3572
20	S	-3.9408	-0.8178	-2.7588
21	O	-4.1319	-1.9746	-3.6469
22	O	-4.3446	0.5219	-3.2124
23	C	-4.6342	-1.1565	-1.1645
24	C	-4.8022	-2.4855	-0.7609
25	C	-4.9255	-0.0858	-0.3081
26	C	-5.2833	-2.7383	0.5210
27	H	-4.5647	-3.2992	-1.4375
28	C	-5.4061	-0.3615	0.9655
29	H	-4.7854	0.9366	-0.6419
30	C	-5.5912	-1.6864	1.4013
31	H	-5.4270	-3.7655	0.8433
32	H	-5.6425	0.4589	1.6370
33	C	-6.1320	-1.9631	2.7787
34	H	-5.6819	-1.2965	3.5214
35	H	-7.2151	-1.7892	2.8042
36	H	-5.9531	-2.9980	3.0823
37	Br	-1.9058	-2.0333	1.3787
38	Br	-2.0542	3.0692	-2.4134
39	Br	0.3100	-2.8478	-4.6324
40	Br	4.0128	-3.2840	-4.6456
41	Br	5.2038	-0.9283	-2.0514

42	Br	3.5150	0.0148	2.3047
43	Br	0.7080	-1.6707	4.0426
44	Br	0.6750	5.5927	-1.9212
45	Br	3.5921	3.5493	-0.9007
46	H	-0.8320	0.5867	-4.3319
47	Si	-0.2812	1.0720	-5.6257
48	C	-0.7307	2.8923	-5.8383
49	C	-1.0058	0.0432	-7.0274
50	C	1.5999	0.9150	-5.5711
51	H	-1.8150	3.0436	-5.8024
52	H	-0.2796	3.5106	-5.0552
53	H	-0.3694	3.2640	-6.8048
54	H	-0.7203	-1.0105	-6.9474
55	H	-2.1009	0.0909	-7.0046
56	H	-0.6767	0.4105	-8.0063
57	C	2.3448	1.6998	-4.6694
58	C	2.3101	0.0501	-6.4224
59	C	3.7367	1.6171	-4.6095
60	H	1.8324	2.3925	-4.0077
61	C	3.7052	-0.0298	-6.3762
62	H	1.7740	-0.5715	-7.1351
63	C	4.4212	0.7503	-5.4664
64	H	4.2873	2.2273	-3.8989
65	H	4.2310	-0.7038	-7.0464
66	H	5.5047	0.6841	-5.4242

TpBr3CuN_SiH_t_v2_SP
Energy (POTENTIAL) = -26981.8810423 Eh

	Atom	X	Y	Z
1	Cu	-0.8858	-0.4077	-1.7507
2	N	0.8269	-1.3846	-2.5228
3	N	2.0367	-1.0428	-1.9969
4	N	0.2121	1.4177	-1.6645
5	N	1.5171	1.3294	-1.2832
6	N	-0.0950	-0.7921	0.1896
7	N	1.2418	-0.5727	0.3549
8	B	2.1043	-0.0345	-0.8216
9	C	1.0501	-2.2393	-3.5175
10	C	2.4328	-2.4636	-3.6708
11	C	3.0185	-1.6772	-2.6796
12	C	-0.0223	2.6892	-1.9809
13	C	1.1446	3.4621	-1.8296
14	C	2.0986	2.5479	-1.3838
15	C	-0.5688	-1.2449	1.3468
16	C	0.4602	-1.3347	2.3032
17	C	1.5940	-0.8942	1.6231
18	H	3.2238	0.1067	-0.4711
19	N	-2.5495	-0.6923	-2.3916
20	S	-4.1146	-0.9677	-2.8817
21	O	-4.1576	-2.2492	-3.6002
22	O	-4.5730	0.2601	-3.5530
23	C	-4.9014	-1.1283	-1.3024
24	C	-5.1126	-2.4036	-0.7698
25	C	-5.2229	0.0326	-0.5842
26	C	-5.6711	-2.5121	0.5014
27	H	-4.8491	-3.2869	-1.3412
28	C	-5.7745	-0.0996	0.6833
29	H	-5.0476	1.0124	-1.0156
30	C	-6.0070	-1.3686	1.2466
31	H	-5.8497	-3.4971	0.9226
32	H	-6.0304	0.7909	1.2501
33	C	-6.6266	-1.4882	2.6134
34	H	-6.1745	-0.7802	3.3156
35	H	-7.6980	-1.2557	2.5673
36	H	-6.5204	-2.4982	3.0176
37	Br	-2.3948	-1.6669	1.5398
38	Br	-1.7352	3.2486	-2.5308
39	Br	-0.3724	-2.9832	-4.4903
40	Br	3.2890	-3.5746	-4.9255
41	Br	4.8491	-1.4550	-2.3137

42	Br	3.3475	-0.7412	2.2895
43	Br	0.3334	-1.9158	4.0895
44	Br	1.3638	5.3034	-2.1479
45	Br	3.9109	2.8694	-1.0012
46	H	2.2799	0.8781	-3.7761
47	Si	2.2786	1.1713	-5.2304
48	C	3.7785	0.3614	-6.0324
49	C	2.3334	3.0478	-5.4290
50	C	0.6719	0.4790	-5.9347
51	H	4.7025	0.7122	-5.5584
52	H	3.7471	-0.7287	-5.9355
53	H	3.8432	0.6014	-7.1000
54	H	1.4410	3.5154	-4.9990
55	H	3.2094	3.4704	-4.9231
56	H	2.3818	3.3347	-6.4860
57	C	-0.5371	0.6677	-5.2391
58	C	0.6152	-0.1919	-7.1694
59	C	-1.7561	0.2293	-5.7569
60	H	-0.5292	1.1571	-4.2705
61	C	-0.6005	-0.6452	-7.6916
62	H	1.5279	-0.3672	-7.7346
63	C	-1.7888	-0.4294	-6.9896
64	H	-2.6730	0.3956	-5.1993
65	H	-0.6198	-1.1620	-8.6477
66	H	-2.7349	-0.7765	-7.3962

TpBr3Cu_HNSi_s_v2
Energy (POTENTIAL) = -2487.80218887 Eh

	Atom	X	Y	Z
1	N	-3.1792	0.0200	1.3204
2	N	-1.9360	0.0155	1.8878
3	N	-2.4906	1.2653	-0.7900
4	N	-1.1428	1.1073	-0.9547
5	N	-2.8359	-1.2340	-0.8360
6	N	-2.4255	-2.2707	-0.0528
7	C	-2.1055	-0.1626	3.1979
8	C	-3.4713	-0.2817	3.5133
9	C	-4.1129	-0.1641	2.2828
10	C	-0.7053	2.2089	-1.5611
11	C	-1.7531	3.1175	-1.7946
12	C	-2.8738	2.4673	-1.2796
13	C	-2.1218	-3.2590	-0.8846
14	C	-2.3212	-2.8936	-2.2327
15	C	-2.7862	-1.5854	-2.1453
16	B	-3.3458	0.0989	-0.2150
17	H	-4.4875	0.2919	-0.4884
18	S	2.5916	0.6432	1.9018
19	O	1.9450	1.9589	1.9930
20	O	2.5790	-0.2861	3.0364
21	C	4.2684	0.8850	1.3460
22	C	5.2861	0.0868	1.8706
23	C	4.5276	1.8625	0.3819
24	C	6.5831	0.2622	1.3972
25	H	5.0629	-0.6596	2.6229
26	C	5.8311	2.0146	-0.0849
27	H	3.7326	2.5046	0.0164
28	C	6.8735	1.2128	0.4060
29	H	7.3812	-0.3586	1.7932
30	H	6.0427	2.7703	-0.8359
31	Cu	-0.2676	0.0932	0.7324
32	N	1.7990	-0.1713	0.5961
33	C	8.2748	1.3412	-0.1319
34	H	8.4024	2.2566	-0.7158
35	H	9.0123	1.3393	0.6774
36	H	8.5103	0.4908	-0.7840
37	Br	1.1117	2.4005	-2.0505
38	Br	-1.6713	4.8072	-2.6225
39	Br	-4.6489	3.0894	-1.2452
40	Br	-3.2957	-0.4299	-3.5455
41	Br	-2.0109	-3.9091	-3.7898

42	Br	-1.4918	-4.9190	-0.2263
43	Br	-0.6309	-0.2411	4.3650
44	Br	-4.2527	-0.5300	5.2090
45	Br	-5.9635	-0.2381	1.9442
46	H	2.0135	0.3722	-0.2462
47	C	3.8500	-2.4127	0.1852
48	C	4.4667	-3.1651	1.2019
49	C	4.6349	-2.0075	-0.9117
50	C	5.8215	-3.4969	1.1296
51	H	3.8882	-3.4927	2.0617
52	C	5.9892	-2.3340	-0.9862
53	H	4.1894	-1.4271	-1.7164
54	C	6.5842	-3.0801	0.0359
55	H	6.2805	-4.0782	1.9244
56	H	6.5802	-2.0087	-1.8378
57	H	7.6386	-3.3361	-0.0215
58	C	1.1329	-2.8666	1.6379
59	H	1.5307	-2.5702	2.6118
60	H	0.0686	-2.6109	1.6122
61	H	1.2238	-3.9522	1.5329
62	C	1.2250	-2.1064	-1.4194
63	H	1.7633	-1.5138	-2.1678
64	H	1.2138	-3.1460	-1.7629
65	H	0.1930	-1.7455	-1.3866
66	Si	2.0326	-1.9816	0.2664

TpBr3Cu_HNSi_s_v1
Energy (POTENTIAL) = -2487.80411952 Eh

	Atom	X	Y	Z
1	N	-3.2402	-0.1798	1.4304
2	N	-1.9920	-0.2647	1.9853
3	N	-2.5330	1.2959	-0.5156
4	N	-1.2260	1.0790	-0.8537
5	N	-2.9570	-1.1619	-0.8664
6	N	-2.4808	-2.2659	-0.2249
7	C	-2.1650	-0.6189	3.2608
8	C	-3.5304	-0.7762	3.5603
9	C	-4.1730	-0.4903	2.3594
10	C	-0.7629	2.2362	-1.3248
11	C	-1.7461	3.2411	-1.2762
12	C	-2.8595	2.5874	-0.7480
13	C	-2.1711	-3.1365	-1.1769
14	C	-2.4303	-2.6225	-2.4657
15	C	-2.9389	-1.3530	-2.2085
16	B	-3.4222	0.0954	-0.0807
17	H	-4.5598	0.3587	-0.3111
18	S	2.6742	0.6514	1.6977
19	O	2.5602	-0.1437	2.9269
20	O	3.9882	0.9263	1.1033
21	C	1.8107	2.2027	1.8894
22	C	2.2491	3.3221	1.1832
23	C	0.6768	2.2562	2.7072
24	C	1.5167	4.5060	1.2809
25	H	3.1416	3.2694	0.5699
26	C	-0.0438	3.4421	2.7854
27	H	0.3662	1.3808	3.2627
28	C	0.3584	4.5822	2.0669
29	H	1.8481	5.3809	0.7294
30	H	-0.9300	3.4867	3.4128
31	Cu	-0.3535	-0.0474	0.7842
32	N	1.7065	-0.1758	0.5185
33	C	-0.4402	5.8560	2.1625
34	H	-0.3653	6.2854	3.1688
35	H	-0.0911	6.6056	1.4478
36	H	-1.5022	5.6673	1.9720
37	Br	0.9828	2.3603	-2.0354
38	Br	-1.6041	5.0413	-1.8094
39	Br	-4.5601	3.3033	-0.3819
40	Br	-3.5277	-0.0491	-3.4359
41	Br	-2.1502	-3.4405	-4.1407

42	Br	-1.4554	-4.8320	-0.7316
43	Br	-0.7182	-0.8567	4.4452
44	Br	-4.3126	-1.2515	5.2057
45	Br	-6.0235	-0.5171	2.0181
46	H	1.8854	0.3176	-0.3624
47	C	3.8405	-2.3593	0.2844
48	C	4.4878	-2.6167	1.5086
49	C	4.6108	-2.3806	-0.8935
50	C	5.8578	-2.8777	1.5543
51	H	3.9219	-2.5971	2.4358
52	C	5.9817	-2.6427	-0.8509
53	H	4.1415	-2.1935	-1.8561
54	C	6.6066	-2.8905	0.3739
55	H	6.3417	-3.0700	2.5079
56	H	6.5602	-2.6548	-1.7706
57	H	7.6733	-3.0943	0.4089
58	C	1.0573	-2.8792	1.5699
59	H	1.3356	-2.4887	2.5525
60	H	-0.0207	-2.7377	1.4381
61	H	1.2658	-3.9543	1.5495
62	C	1.2802	-2.1541	-1.4864
63	H	1.8440	-1.5711	-2.2230
64	H	1.2684	-3.1971	-1.8177
65	H	0.2495	-1.7849	-1.4884
66	Si	2.0089	-1.9933	0.2305

TpBr3Cu_HNSi_s_v2_SP
Energy (POTENTIAL) = -26982.0074691 Eh

	Atom	X	Y	Z
1	N	-3.1792	0.0200	1.3204
2	N	-1.9360	0.0155	1.8878
3	N	-2.4906	1.2653	-0.7900
4	N	-1.1428	1.1073	-0.9547
5	N	-2.8359	-1.2340	-0.8360
6	N	-2.4255	-2.2707	-0.0528
7	C	-2.1055	-0.1626	3.1979
8	C	-3.4713	-0.2817	3.5133
9	C	-4.1129	-0.1641	2.2828
10	C	-0.7053	2.2089	-1.5611
11	C	-1.7531	3.1175	-1.7946
12	C	-2.8738	2.4673	-1.2796
13	C	-2.1218	-3.2590	-0.8846
14	C	-2.3212	-2.8936	-2.2327
15	C	-2.7862	-1.5854	-2.1453
16	B	-3.3458	0.0989	-0.2150
17	H	-4.4874	0.2919	-0.4884
18	S	2.5916	0.6432	1.9018
19	O	1.9450	1.9589	1.9930
20	O	2.5790	-0.2861	3.0364
21	C	4.2684	0.8850	1.3460
22	C	5.2861	0.0868	1.8706
23	C	4.5276	1.8625	0.3819
24	C	6.5831	0.2622	1.3972
25	H	5.0629	-0.6596	2.6229
26	C	5.8311	2.0146	-0.0849
27	H	3.7326	2.5046	0.0164
28	C	6.8735	1.2128	0.4060
29	H	7.3812	-0.3586	1.7932
30	H	6.0427	2.7703	-0.8359
31	Cu	-0.2676	0.0932	0.7324
32	N	1.7990	-0.1713	0.5961
33	C	8.2748	1.3412	-0.1319
34	H	8.4024	2.2566	-0.7158
35	H	9.0123	1.3393	0.6774
36	H	8.5103	0.4908	-0.7840
37	Br	1.1118	2.4005	-2.0505
38	Br	-1.6713	4.8072	-2.6225
39	Br	-4.6489	3.0894	-1.2452
40	Br	-3.2957	-0.4299	-3.5455
41	Br	-2.0109	-3.9091	-3.7898

42	Br	-1.4918	-4.9190	-0.2263
43	Br	-0.6309	-0.2411	4.3650
44	Br	-4.2527	-0.5300	5.2090
45	Br	-5.9636	-0.2381	1.9442
46	H	2.0135	0.3722	-0.2462
47	C	3.8500	-2.4127	0.1852
48	C	4.4667	-3.1651	1.2019
49	C	4.6349	-2.0075	-0.9117
50	C	5.8215	-3.4969	1.1296
51	H	3.8882	-3.4927	2.0617
52	C	5.9892	-2.3340	-0.9862
53	H	4.1894	-1.4271	-1.7164
54	C	6.5842	-3.0801	0.0359
55	H	6.2805	-4.0782	1.9244
56	H	6.5802	-2.0087	-1.8378
57	H	7.6386	-3.3361	-0.0215
58	C	1.1329	-2.8666	1.6379
59	H	1.5307	-2.5702	2.6118
60	H	0.0686	-2.6109	1.6122
61	H	1.2238	-3.9522	1.5329
62	C	1.2250	-2.1064	-1.4194
63	H	1.7633	-1.5138	-2.1678
64	H	1.2138	-3.1460	-1.7629
65	H	0.1930	-1.7455	-1.3866
66	Si	2.0326	-1.9816	0.2664

TpBr3Cu_HNSi_s_v1_SP
Energy (POTENTIAL) = -26982.0090722 Eh

	Atom	X	Y	Z
1	N	-3.2402	-0.1798	1.4304
2	N	-1.9920	-0.2647	1.9853
3	N	-2.5330	1.2959	-0.5156
4	N	-1.2260	1.0790	-0.8537
5	N	-2.9570	-1.1619	-0.8664
6	N	-2.4808	-2.2659	-0.2249
7	C	-2.1650	-0.6189	3.2608
8	C	-3.5304	-0.7762	3.5603
9	C	-4.1730	-0.4903	2.3594
10	C	-0.7629	2.2362	-1.3248
11	C	-1.7461	3.2411	-1.2762
12	C	-2.8595	2.5874	-0.7480
13	C	-2.1711	-3.1365	-1.1769
14	C	-2.4303	-2.6225	-2.4657
15	C	-2.9389	-1.3530	-2.2085
16	B	-3.4222	0.0954	-0.0807
17	H	-4.5598	0.3587	-0.3111
18	S	2.6742	0.6514	1.6977
19	O	2.5602	-0.1437	2.9270
20	O	3.9882	0.9263	1.1033
21	C	1.8107	2.2027	1.8894
22	C	2.2491	3.3221	1.1832
23	C	0.6768	2.2562	2.7072
24	C	1.5167	4.5060	1.2809
25	H	3.1416	3.2694	0.5699
26	C	-0.0438	3.4421	2.7854
27	H	0.3662	1.3808	3.2627
28	C	0.3584	4.5822	2.0669
29	H	1.8481	5.3809	0.7294
30	H	-0.9300	3.4867	3.4128
31	Cu	-0.3535	-0.0474	0.7842
32	N	1.7065	-0.1758	0.5185
33	C	-0.4402	5.8560	2.1625
34	H	-0.3653	6.2854	3.1688
35	H	-0.0911	6.6056	1.4478
36	H	-1.5022	5.6673	1.9720
37	Br	0.9828	2.3603	-2.0354
38	Br	-1.6041	5.0413	-1.8094
39	Br	-4.5601	3.3033	-0.3819
40	Br	-3.5277	-0.0491	-3.4359
41	Br	-2.1502	-3.4405	-4.1407

42	Br	-1.4554	-4.8320	-0.7316
43	Br	-0.7182	-0.8567	4.4452
44	Br	-4.3126	-1.2515	5.2057
45	Br	-6.0235	-0.5171	2.0181
46	H	1.8854	0.3176	-0.3624
47	C	3.8405	-2.3593	0.2844
48	C	4.4878	-2.6167	1.5086
49	C	4.6108	-2.3806	-0.8935
50	C	5.8578	-2.8777	1.5543
51	H	3.9219	-2.5971	2.4358
52	C	5.9817	-2.6427	-0.8509
53	H	4.1415	-2.1935	-1.8561
54	C	6.6066	-2.8905	0.3739
55	H	6.3417	-3.0700	2.5079
56	H	6.5602	-2.6548	-1.7706
57	H	7.6733	-3.0943	0.4089
58	C	1.0573	-2.8792	1.5699
59	H	1.3356	-2.4887	2.5525
60	H	-0.0207	-2.7377	1.4381
61	H	1.2658	-3.9543	1.5495
62	C	1.2802	-2.1541	-1.4864
63	H	1.8440	-1.5711	-2.2230
64	H	1.2684	-3.1971	-1.8177
65	H	0.2495	-1.7849	-1.4884
66	Si	2.0089	-1.9933	0.2305

TpBr3Cu_HNSi_t_v2_SP
Energy (POTENTIAL) = -26981.8645228 Eh

	Atom	X	Y	Z
1	N	-3.1792	0.0200	1.3204
2	N	-1.9360	0.0155	1.8878
3	N	-2.4906	1.2653	-0.7900
4	N	-1.1428	1.1073	-0.9547
5	N	-2.8359	-1.2340	-0.8360
6	N	-2.4255	-2.2707	-0.0528
7	C	-2.1055	-0.1626	3.1979
8	C	-3.4713	-0.2817	3.5133
9	C	-4.1129	-0.1641	2.2828
10	C	-0.7053	2.2089	-1.5611
11	C	-1.7531	3.1175	-1.7946
12	C	-2.8738	2.4673	-1.2796
13	C	-2.1218	-3.2590	-0.8846
14	C	-2.3212	-2.8936	-2.2327
15	C	-2.7862	-1.5854	-2.1453
16	B	-3.3458	0.0989	-0.2150
17	H	-4.4874	0.2919	-0.4884
18	S	2.5916	0.6432	1.9018
19	O	1.9450	1.9589	1.9930
20	O	2.5790	-0.2861	3.0364
21	C	4.2684	0.8850	1.3460
22	C	5.2861	0.0868	1.8706
23	C	4.5276	1.8625	0.3819
24	C	6.5831	0.2622	1.3972
25	H	5.0629	-0.6596	2.6229
26	C	5.8311	2.0146	-0.0849
27	H	3.7326	2.5046	0.0164
28	C	6.8735	1.2128	0.4060
29	H	7.3812	-0.3586	1.7932
30	H	6.0427	2.7703	-0.8359
31	Cu	-0.2676	0.0932	0.7324
32	N	1.7990	-0.1713	0.5961
33	C	8.2748	1.3412	-0.1319
34	H	8.4024	2.2566	-0.7158
35	H	9.0123	1.3393	0.6774
36	H	8.5103	0.4908	-0.7840
37	Br	1.1118	2.4005	-2.0505
38	Br	-1.6713	4.8072	-2.6225
39	Br	-4.6489	3.0894	-1.2452
40	Br	-3.2957	-0.4299	-3.5455
41	Br	-2.0109	-3.9091	-3.7898

42	Br	-1.4918	-4.9190	-0.2263
43	Br	-0.6309	-0.2411	4.3650
44	Br	-4.2527	-0.5300	5.2090
45	Br	-5.9636	-0.2381	1.9442
46	H	2.0135	0.3722	-0.2462
47	C	3.8500	-2.4127	0.1852
48	C	4.4667	-3.1651	1.2019
49	C	4.6349	-2.0075	-0.9117
50	C	5.8215	-3.4969	1.1296
51	H	3.8882	-3.4927	2.0617
52	C	5.9892	-2.3340	-0.9862
53	H	4.1894	-1.4271	-1.7164
54	C	6.5842	-3.0801	0.0359
55	H	6.2805	-4.0782	1.9244
56	H	6.5802	-2.0087	-1.8378
57	H	7.6386	-3.3361	-0.0215
58	C	1.1329	-2.8666	1.6379
59	H	1.5307	-2.5702	2.6118
60	H	0.0686	-2.6109	1.6122
61	H	1.2238	-3.9522	1.5329
62	C	1.2250	-2.1064	-1.4194
63	H	1.7633	-1.5138	-2.1678
64	H	1.2138	-3.1460	-1.7629
65	H	0.1930	-1.7455	-1.3866
66	Si	2.0326	-1.9816	0.2664

TpBr3Cu_HNSi_t_v1_SP
Energy (POTENTIAL) = -26981.867272 Eh

	Atom	X	Y	Z
1	N	-3.2402	-0.1798	1.4304
2	N	-1.9920	-0.2647	1.9853
3	N	-2.5330	1.2959	-0.5156
4	N	-1.2260	1.0790	-0.8537
5	N	-2.9570	-1.1619	-0.8664
6	N	-2.4808	-2.2659	-0.2249
7	C	-2.1650	-0.6189	3.2608
8	C	-3.5304	-0.7762	3.5603
9	C	-4.1730	-0.4903	2.3594
10	C	-0.7629	2.2362	-1.3248
11	C	-1.7461	3.2411	-1.2762
12	C	-2.8595	2.5874	-0.7480
13	C	-2.1711	-3.1365	-1.1769
14	C	-2.4303	-2.6225	-2.4657
15	C	-2.9389	-1.3530	-2.2085
16	B	-3.4222	0.0954	-0.0807
17	H	-4.5598	0.3587	-0.3111
18	S	2.6742	0.6514	1.6977
19	O	2.5602	-0.1437	2.9270
20	O	3.9882	0.9263	1.1033
21	C	1.8107	2.2027	1.8894
22	C	2.2491	3.3221	1.1832
23	C	0.6768	2.2562	2.7072
24	C	1.5167	4.5060	1.2809
25	H	3.1416	3.2694	0.5699
26	C	-0.0438	3.4421	2.7854
27	H	0.3662	1.3808	3.2627
28	C	0.3584	4.5822	2.0669
29	H	1.8481	5.3809	0.7294
30	H	-0.9300	3.4867	3.4128
31	Cu	-0.3535	-0.0474	0.7842
32	N	1.7065	-0.1758	0.5185
33	C	-0.4402	5.8560	2.1625
34	H	-0.3653	6.2854	3.1688
35	H	-0.0911	6.6056	1.4478
36	H	-1.5022	5.6673	1.9720
37	Br	0.9828	2.3603	-2.0354
38	Br	-1.6041	5.0413	-1.8094
39	Br	-4.5601	3.3033	-0.3819
40	Br	-3.5277	-0.0491	-3.4359
41	Br	-2.1502	-3.4405	-4.1407

42	Br	-1.4554	-4.8320	-0.7316
43	Br	-0.7182	-0.8567	4.4452
44	Br	-4.3126	-1.2515	5.2057
45	Br	-6.0235	-0.5171	2.0181
46	H	1.8854	0.3176	-0.3624
47	C	3.8405	-2.3593	0.2844
48	C	4.4878	-2.6167	1.5086
49	C	4.6108	-2.3806	-0.8935
50	C	5.8578	-2.8777	1.5543
51	H	3.9219	-2.5971	2.4358
52	C	5.9817	-2.6427	-0.8509
53	H	4.1415	-2.1935	-1.8561
54	C	6.6066	-2.8905	0.3739
55	H	6.3417	-3.0700	2.5079
56	H	6.5602	-2.6548	-1.7706
57	H	7.6733	-3.0943	0.4089
58	C	1.0573	-2.8792	1.5699
59	H	1.3356	-2.4887	2.5525
60	H	-0.0207	-2.7377	1.4381
61	H	1.2658	-3.9543	1.5495
62	C	1.2802	-2.1541	-1.4864
63	H	1.8440	-1.5711	-2.2230
64	H	1.2684	-3.1971	-1.8177
65	H	0.2495	-1.7849	-1.4884
66	Si	2.0089	-1.9933	0.2305

step_000_t_SP
Energy (POTENTIAL) = -26981.8816319 Eh

	Atom	X	Y	Z
1	Cu	-0.5697	-0.4198	-1.8271
2	N	1.2803	-1.0765	-2.6005
3	N	2.4127	-0.6096	-2.0020
4	N	0.2098	1.5422	-1.6242
5	N	1.5186	1.6510	-1.2610
6	N	0.2823	-0.7859	0.1250
7	N	1.5375	-0.2997	0.3401
8	B	2.3125	0.3885	-0.8211
9	C	1.6544	-1.9649	-3.5166
10	C	3.0553	-2.1028	-3.5324
11	C	3.4927	-1.2162	-2.5504
12	C	-0.2461	2.7706	-1.8541
13	C	0.7770	3.7199	-1.6592
14	C	1.8804	2.9567	-1.2778
15	C	-0.1435	-1.3062	1.2713
16	C	0.8366	-1.1698	2.2733
17	C	1.8893	-0.5214	1.6292
18	H	3.3933	0.7002	-0.4590
19	N	-2.2475	-0.8022	-2.3864
20	S	-3.8473	-0.8820	-2.8363
21	O	-4.0121	-2.0641	-3.6956
22	O	-4.2403	0.4434	-3.3389
23	C	-4.5775	-1.1762	-1.2492
24	C	-4.7460	-2.4935	-0.8088
25	C	-4.8912	-0.0820	-0.4310
26	C	-5.2507	-2.7107	0.4707
27	H	-4.4912	-3.3257	-1.4560
28	C	-5.3938	-0.3224	0.8412
29	H	-4.7520	0.9308	-0.7929
30	C	-5.5805	-1.6348	1.3130
31	H	-5.3958	-3.7287	0.8206
32	H	-5.6476	0.5164	1.4830
33	C	-6.1466	-1.8717	2.6877
34	H	-5.6989	-1.1936	3.4214
35	H	-7.2274	-1.6823	2.6920
36	H	-5.9866	-2.9012	3.0188
37	Br	-1.8598	-2.0764	1.3898
38	Br	-2.0404	3.0500	-2.3395
39	Br	0.3829	-2.8506	-4.5835
40	Br	4.0946	-3.2357	-4.6145
41	Br	5.2617	-0.8689	-2.0179

42	Br	3.5480	-0.0095	2.3567
43	Br	0.7473	-1.7323	4.0680
44	Br	0.6781	5.5876	-1.8727
45	Br	3.6136	3.5613	-0.8668
46	H	-0.8867	0.6085	-4.2725
47	Si	-0.3594	1.0768	-5.5817
48	C	-0.8007	2.8985	-5.8003
49	C	-1.1222	0.0364	-6.9540
50	C	1.5212	0.9068	-5.5629
51	H	-1.8834	3.0568	-5.7491
52	H	-0.3340	3.5181	-5.0274
53	H	-0.4516	3.2625	-6.7742
54	H	-0.8525	-1.0203	-6.8586
55	H	-2.2159	0.1024	-6.9141
56	H	-0.8037	0.3798	-7.9450
57	C	2.2875	1.6720	-4.6624
58	C	2.2100	0.0506	-6.4404
59	C	3.6797	1.5792	-4.6291
60	H	1.7919	2.3567	-3.9797
61	C	3.6050	-0.0397	-6.4203
62	H	1.6564	-0.5555	-7.1529
63	C	4.3426	0.7212	-5.5114
64	H	4.2472	2.1752	-3.9195
65	H	4.1142	-0.7070	-7.1097
66	H	5.4262	0.6462	-5.4890

step_001_t_SP
Energy (POTENTIAL) = -26981.881418 Eh

	Atom	X	Y	Z
1	Cu	-0.5387	-0.4010	-1.8062
2	N	1.3154	-1.0663	-2.5702
3	N	2.4459	-0.5986	-1.9689
4	N	0.2419	1.5583	-1.5908
5	N	1.5516	1.6620	-1.2290
6	N	0.3017	-0.7755	0.1471
7	N	1.5569	-0.2923	0.3691
8	B	2.3403	0.3967	-0.7864
9	C	1.6926	-1.9529	-3.4868
10	C	3.0939	-2.0879	-3.5006
11	C	3.5280	-1.2017	-2.5169
12	C	-0.2096	2.7887	-1.8198
13	C	0.8177	3.7338	-1.6269
14	C	1.9188	2.9662	-1.2472
15	C	-0.1304	-1.2984	1.2896
16	C	0.8454	-1.1671	2.2965
17	C	1.9023	-0.5188	1.6592
18	H	3.4194	0.7058	-0.4169
19	N	-2.2055	-0.7722	-2.4051
20	S	-3.8016	-0.8334	-2.8724
21	O	-3.9666	-2.0021	-3.7499
22	O	-4.1848	0.5008	-3.3585
23	C	-4.5437	-1.1461	-1.2937
24	C	-4.7119	-2.4685	-0.8685
25	C	-4.8681	-0.0617	-0.4667
26	C	-5.2274	-2.7007	0.4040
27	H	-4.4489	-3.2932	-1.5220
28	C	-5.3815	-0.3170	0.7983
29	H	-4.7290	0.9554	-0.8165
30	C	-5.5682	-1.6349	1.2547
31	H	-5.3724	-3.7228	0.7418
32	H	-5.6438	0.5141	1.4465
33	C	-6.1439	-1.8871	2.6226
34	H	-5.6863	-1.2308	3.3701
35	H	-7.2209	-1.6781	2.6274
36	H	-6.0030	-2.9251	2.9355
37	Br	-1.8490	-2.0654	1.3965
38	Br	-2.0037	3.0767	-2.3010
39	Br	0.4251	-2.8409	-4.5563
40	Br	4.1375	-3.2171	-4.5827
41	Br	5.2959	-0.8463	-1.9855

42	Br	3.5586	-0.0130	2.3964
43	Br	0.7466	-1.7349	4.0890
44	Br	0.7250	5.6020	-1.8387
45	Br	3.6548	3.5640	-0.8376
46	H	-0.9265	0.5977	-4.2360
47	Si	-0.4155	1.0517	-5.5572
48	C	-0.8619	2.8700	-5.7931
49	C	-1.1952	-0.0055	-6.9071
50	C	1.4655	0.8823	-5.5573
51	H	-1.9445	3.0266	-5.7331
52	H	-0.3890	3.5004	-5.0328
53	H	-0.5234	3.2225	-6.7749
54	H	-0.9299	-1.0621	-6.7988
55	H	-2.2881	0.0666	-6.8573
56	H	-0.8849	0.3216	-7.9061
57	C	2.2408	1.6407	-4.6588
58	C	2.1456	0.0310	-6.4462
59	C	3.6332	1.5468	-4.6389
60	H	1.7528	2.3204	-3.9659
61	C	3.5406	-0.0610	-6.4392
62	H	1.5847	-0.5706	-7.1568
63	C	4.2873	0.6938	-5.5325
64	H	4.2077	2.1379	-3.9309
65	H	4.0427	-0.7253	-7.1367
66	H	5.3709	0.6171	-5.5198

step_002_t_SP
Energy (POTENTIAL) = -26981.8810227 Eh

	Atom	X	Y	Z
1	Cu	-0.5093	-0.3916	-1.7864
2	N	1.3443	-1.0722	-2.5451
3	N	2.4751	-0.6106	-1.9400
4	N	0.2820	1.5638	-1.5583
5	N	1.5935	1.6530	-1.1988
6	N	0.3186	-0.7828	0.1666
7	N	1.5752	-0.3059	0.3955
8	B	2.3695	0.3802	-0.7541
9	C	1.7208	-1.9519	-3.4687
10	C	3.1221	-2.0868	-3.4844
11	C	3.5569	-1.2089	-2.4936
12	C	-0.1536	2.7991	-1.7938
13	C	0.8865	3.7316	-1.6086
14	C	1.9780	2.9519	-1.2261
15	C	-0.1224	-1.3033	1.3069
16	C	0.8484	-1.1767	2.3191
17	C	1.9122	-0.5340	1.6877
18	H	3.4487	0.6804	-0.3777
19	N	-2.1678	-0.7360	-2.4249
20	S	-3.7618	-0.7739	-2.9028
21	O	-3.9333	-1.9277	-3.7987
22	O	-4.1343	0.5702	-3.3693
23	C	-4.5106	-1.1041	-1.3300
24	C	-4.6860	-2.4309	-0.9221
25	C	-4.8335	-0.0289	-0.4904
26	C	-5.2078	-2.6769	0.3454
27	H	-4.4238	-3.2487	-1.5847
28	C	-5.3529	-0.2979	0.7692
29	H	-4.6885	0.9919	-0.8267
30	C	-5.5472	-1.6206	1.2082
31	H	-5.3584	-3.7026	0.6696
32	H	-5.6141	0.5262	1.4269
33	C	-6.1278	-1.8873	2.5714
34	H	-5.6655	-1.2459	3.3289
35	H	-7.2030	-1.6686	2.5776
36	H	-5.9966	-2.9308	2.8696
37	Br	-1.8456	-2.0616	1.4048
38	Br	-1.9438	3.1117	-2.2764
39	Br	0.4534	-2.8342	-4.5431
40	Br	4.1658	-3.2047	-4.5780
41	Br	5.3254	-0.8557	-1.9625

42	Br	3.5667	-0.0369	2.4347
43	Br	0.7368	-1.7435	4.1111
44	Br	0.8202	5.5992	-1.8335
45	Br	3.7232	3.5289	-0.8258
46	H	-0.9839	0.5680	-4.2274
47	Si	-0.4814	1.0128	-5.5562
48	C	-0.9644	2.8175	-5.8215
49	C	-1.2402	-0.0771	-6.8919
50	C	1.4022	0.8754	-5.5470
51	H	-2.0490	2.9543	-5.7483
52	H	-0.4928	3.4735	-5.0825
53	H	-0.6474	3.1546	-6.8159
54	H	-0.9420	-1.1244	-6.7798
55	H	-2.3342	-0.0376	-6.8334
56	H	-0.9480	0.2529	-7.8955
57	C	2.1592	1.6345	-4.6336
58	C	2.1015	0.0429	-6.4388
59	C	3.5526	1.5602	-4.6032
60	H	1.6563	2.2984	-3.9360
61	C	3.4976	-0.0297	-6.4208
62	H	1.5548	-0.5599	-7.1595
63	C	4.2261	0.7260	-5.5002
64	H	4.1128	2.1511	-3.8837
65	H	4.0148	-0.6801	-7.1206
66	H	5.3105	0.6645	-5.4792

step_003_t_SP
Energy (POTENTIAL) = -26981.8806936 Eh

	Atom	X	Y	Z
1	Cu	-0.4614	-0.3876	-1.8027
2	N	1.3964	-1.0611	-2.5426
3	N	2.5215	-0.5975	-1.9277
4	N	0.3125	1.5527	-1.4977
5	N	1.6225	1.6437	-1.1332
6	N	0.3546	-0.8221	0.1674
7	N	1.6101	-0.3496	0.4116
8	B	2.4059	0.3649	-0.7194
9	C	1.7824	-1.9349	-3.4681
10	C	3.1845	-2.0614	-3.4774
11	C	3.6093	-1.1868	-2.4796
12	C	-0.1376	2.7908	-1.6856
13	C	0.8898	3.7281	-1.4614
14	C	1.9902	2.9473	-1.1075
15	C	-0.1015	-1.3395	1.3025
16	C	0.8577	-1.2171	2.3266
17	C	1.9309	-0.5780	1.7080
18	H	3.4822	0.6623	-0.3326
19	N	-2.1165	-0.7417	-2.4494
20	S	-3.7060	-0.7609	-2.9443
21	O	-3.8878	-1.9189	-3.8328
22	O	-4.0598	0.5839	-3.4234
23	C	-4.4681	-1.0688	-1.3729
24	C	-4.6633	-2.3894	-0.9542
25	C	-4.7827	0.0178	-0.5449
26	C	-5.1967	-2.6173	0.3119
27	H	-4.4072	-3.2164	-1.6077
28	C	-5.3143	-0.2330	0.7133
29	H	-4.6228	1.0335	-0.8896
30	C	-5.5286	-1.5490	1.1628
31	H	-5.3625	-3.6380	0.6443
32	H	-5.5694	0.6004	1.3617
33	C	-6.1235	-1.7956	2.5236
34	H	-5.6645	-1.1479	3.2777
35	H	-7.1974	-1.5705	2.5167
36	H	-6.0018	-2.8362	2.8357
37	Br	-1.8325	-2.0832	1.3804
38	Br	-1.9302	3.0952	-2.1609
39	Br	0.5260	-2.8260	-4.5482
40	Br	4.2407	-3.1662	-4.5724
41	Br	5.3738	-0.8252	-1.9398

42	Br	3.5783	-0.0854	2.4745
43	Br	0.7224	-1.7835	4.1172
44	Br	0.7985	5.6022	-1.6124
45	Br	3.7239	3.5302	-0.6668
46	H	-1.0256	0.4518	-4.2500
47	Si	-0.5419	0.9375	-5.5728
48	C	-1.0923	2.7280	-5.7962
49	C	-1.2735	-0.1564	-6.9204
50	C	1.3448	0.8535	-5.5726
51	H	-2.1803	2.8187	-5.7027
52	H	-0.6354	3.3863	-5.0502
53	H	-0.8043	3.0968	-6.7881
54	H	-0.9428	-1.1956	-6.8240
55	H	-2.3679	-0.1508	-6.8565
56	H	-0.9957	0.1955	-7.9206
57	C	2.0877	1.6381	-4.6690
58	C	2.0595	0.0249	-6.4561
59	C	3.4825	1.5903	-4.6390
60	H	1.5731	2.3005	-3.9785
61	C	3.4565	-0.0209	-6.4388
62	H	1.5241	-0.5977	-7.1683
63	C	4.1710	0.7586	-5.5270
64	H	4.0326	2.2008	-3.9279
65	H	3.9856	-0.6690	-7.1316
66	H	5.2565	0.7178	-5.5061

step_004_t_SP
Energy (POTENTIAL) = -26981.8800546 Eh

	Atom	X	Y	Z
1	Cu	-0.3981	-0.3761	-1.8145
2	N	1.4618	-1.0757	-2.5283
3	N	2.5838	-0.6289	-1.8958
4	N	0.3892	1.5429	-1.4597
5	N	1.7009	1.6139	-1.0978
6	N	0.3928	-0.8570	0.1691
7	N	1.6461	-0.3897	0.4348
8	B	2.4635	0.3234	-0.6800
9	C	1.8514	-1.9395	-3.4621
10	C	3.2529	-2.0739	-3.4599
11	C	3.6733	-1.2170	-2.4453
12	C	-0.0394	2.7878	-1.6569
13	C	1.0050	3.7080	-1.4428
14	C	2.0924	2.9104	-1.0856
15	C	-0.0819	-1.3767	1.2951
16	C	0.8614	-1.2611	2.3348
17	C	1.9462	-0.6235	1.7356
18	H	3.5382	0.6068	-0.2781
19	N	-2.0578	-0.7011	-2.4682
20	S	-3.6476	-0.6929	-2.9663
21	O	-3.8398	-1.8335	-3.8751
22	O	-3.9929	0.6621	-3.4221
23	C	-4.4165	-1.0190	-1.4013
24	C	-4.6268	-2.3433	-1.0033
25	C	-4.7317	0.0587	-0.5617
26	C	-5.1766	-2.5844	0.2538
27	H	-4.3717	-3.1633	-1.6659
28	C	-5.2794	-0.2050	0.6867
29	H	-4.5619	1.0777	-0.8915
30	C	-5.5096	-1.5255	1.1153
31	H	-5.3561	-3.6082	0.5691
32	H	-5.5370	0.6215	1.3430
33	C	-6.1171	-1.7832	2.4684
34	H	-5.5752	-1.2413	3.2513
35	H	-7.1552	-1.4305	2.4957
36	H	-6.1143	-2.8475	2.7173
37	Br	-1.8159	-2.1159	1.3445
38	Br	-1.8232	3.1210	-2.1469
39	Br	0.6023	-2.8153	-4.5633
40	Br	4.3150	-3.1601	-4.5677
41	Br	5.4350	-0.8732	-1.8846

42	Br	3.5824	-0.1385	2.5308
43	Br	0.6957	-1.8335	4.1210
44	Br	0.9479	5.5817	-1.6118
45	Br	3.8400	3.4642	-0.6642
46	H	-1.1326	0.4271	-4.2721
47	Si	-0.6518	0.9077	-5.5993
48	C	-1.2548	2.6760	-5.8554
49	C	-1.3336	-0.2307	-6.9356
50	C	1.2356	0.8729	-5.5622
51	H	-2.3442	2.7357	-5.7536
52	H	-0.8114	3.3626	-5.1273
53	H	-0.9865	3.0312	-6.8577
54	H	-0.9699	-1.2568	-6.8191
55	H	-2.4281	-0.2596	-6.8826
56	H	-1.0568	0.1142	-7.9386
57	C	1.9359	1.6653	-4.6321
58	C	1.9921	0.0659	-6.4308
59	C	3.3297	1.6453	-4.5611
60	H	1.3884	2.3093	-3.9498
61	C	3.3886	0.0481	-6.3729
62	H	1.4901	-0.5631	-7.1614
63	C	4.0604	0.8343	-5.4343
64	H	3.8453	2.2602	-3.8284
65	H	3.9506	-0.5838	-7.0548
66	H	5.1454	0.8139	-5.3813

step_005_t_SP
Energy (POTENTIAL) = -26981.879438 Eh

	Atom	X	Y	Z
1	Cu	-0.3925	-0.2761	-1.7747
2	N	1.4602	-1.0246	-2.4429
3	N	2.5846	-0.5624	-1.8278
4	N	0.4240	1.6606	-1.4816
5	N	1.7286	1.7127	-1.0882
6	N	0.3581	-0.6970	0.2223
7	N	1.6240	-0.2632	0.4852
8	B	2.4634	0.4189	-0.6343
9	C	1.8415	-1.9281	-3.3413
10	C	3.2421	-2.0709	-3.3365
11	C	3.6695	-1.1789	-2.3551
12	C	0.0254	2.9116	-1.7060
13	C	1.0822	3.8145	-1.4774
14	C	2.1446	3.0019	-1.0839
15	C	-0.1202	-1.2270	1.3422
16	C	0.8337	-1.1507	2.3759
17	C	1.9287	-0.5285	1.7789
18	H	3.5376	0.6922	-0.2246
19	N	-2.0318	-0.5549	-2.4891
20	S	-3.6166	-0.5687	-3.0034
21	O	-3.7650	-1.6675	-3.9703
22	O	-4.0031	0.7953	-3.3934
23	C	-4.3844	-0.9975	-1.4618
24	C	-4.5659	-2.3457	-1.1385
25	C	-4.7215	0.0254	-0.5640
26	C	-5.1104	-2.6680	0.1028
27	H	-4.2926	-3.1219	-1.8452
28	C	-5.2621	-0.3191	0.6677
29	H	-4.5688	1.0643	-0.8356
30	C	-5.4640	-1.6662	1.0221
31	H	-5.2671	-3.7112	0.3611
32	H	-5.5338	0.4641	1.3698
33	C	-6.0568	-2.0127	2.3622
34	H	-5.5142	-1.5122	3.1717
35	H	-7.0988	-1.6759	2.4204
36	H	-6.0379	-3.0898	2.5469
37	Br	-1.8670	-1.9367	1.3895
38	Br	-1.7378	3.2888	-2.2438
39	Br	0.5774	-2.8468	-4.3879
40	Br	4.2945	-3.2027	-4.4072
41	Br	5.4335	-0.8234	-1.8087

42	Br	3.5833	-0.1001	2.5685
43	Br	0.6677	-1.7491	4.1535
44	Br	1.0671	5.6874	-1.6663
45	Br	3.8906	3.5323	-0.6258
46	H	-1.1604	0.6275	-4.1539
47	Si	-0.6947	0.9649	-5.5312
48	C	-1.2013	2.7395	-5.9189
49	C	-1.4892	-0.2449	-6.7353
50	C	1.1870	0.8124	-5.5448
51	H	-2.2824	2.8728	-5.7998
52	H	-0.6992	3.4531	-5.2577
53	H	-0.9398	2.9974	-6.9522
54	H	-1.2386	-1.2811	-6.4866
55	H	-2.5803	-0.1504	-6.6996
56	H	-1.1725	-0.0551	-7.7673
57	C	1.9679	1.5636	-4.6448
58	C	1.8609	-0.0386	-6.4392
59	C	3.3602	1.4653	-4.6320
60	H	1.4871	2.2357	-3.9400
61	C	3.2552	-0.1377	-6.4365
62	H	1.2945	-0.6374	-7.1477
63	C	4.0077	0.6121	-5.5302
64	H	3.9401	2.0517	-3.9245
65	H	3.7522	-0.8033	-7.1367
66	H	5.0910	0.5299	-5.5201

step_006_t_SP
Energy (POTENTIAL) = -26981.8783479 Eh

	Atom	X	Y	Z
1	Cu	-0.3949	-0.2768	-1.7825
2	N	1.4531	-1.0598	-2.4448
3	N	2.5836	-0.6047	-1.8350
4	N	0.4432	1.6493	-1.4819
5	N	1.7563	1.6874	-1.1183
6	N	0.3622	-0.7026	0.2195
7	N	1.6302	-0.2698	0.4758
8	B	2.4759	0.3898	-0.6524
9	C	1.8240	-1.9811	-3.3292
10	C	3.2225	-2.1428	-3.3208
11	C	3.6609	-1.2426	-2.3522
12	C	0.0560	2.9033	-1.7079
13	C	1.1301	3.7939	-1.5133
14	C	2.1905	2.9702	-1.1370
15	C	-0.1183	-1.2103	1.3488
16	C	0.8350	-1.1195	2.3818
17	C	1.9330	-0.5130	1.7744
18	H	3.5540	0.6549	-0.2475
19	N	-2.0327	-0.4839	-2.5322
20	S	-3.6293	-0.4737	-3.0122
21	O	-3.8040	-1.5454	-4.0050
22	O	-4.0197	0.9015	-3.3567
23	C	-4.3706	-0.9415	-1.4678
24	C	-4.5460	-2.2976	-1.1768
25	C	-4.7009	0.0575	-0.5414
26	C	-5.0760	-2.6527	0.0618
27	H	-4.2796	-3.0549	-1.9062
28	C	-5.2274	-0.3194	0.6872
29	H	-4.5546	1.1034	-0.7883
30	C	-5.4214	-1.6756	1.0101
31	H	-5.2266	-3.7026	0.2958
32	H	-5.4931	0.4450	1.4119
33	C	-5.9948	-2.0576	2.3492
34	H	-5.4430	-1.5753	3.1636
35	H	-7.0370	-1.7266	2.4307
36	H	-5.9694	-3.1389	2.5070
37	Br	-1.8679	-1.9117	1.4086
38	Br	-1.7136	3.2958	-2.2117
39	Br	0.5515	-2.8973	-4.3677
40	Br	4.2607	-3.2994	-4.3793
41	Br	5.4295	-0.8980	-1.8137

42	Br	3.5892	-0.0793	2.5580
43	Br	0.6652	-1.6841	4.1701
44	Br	1.1361	5.6643	-1.7242
45	Br	3.9572	3.4764	-0.7353
46	H	-1.2279	0.5468	-4.2010
47	Si	-0.7363	0.9334	-5.5630
48	C	-1.2835	2.7020	-5.9170
49	C	-1.4694	-0.2751	-6.8058
50	C	1.1474	0.8272	-5.5268
51	H	-2.3673	2.8061	-5.7950
52	H	-0.7992	3.4150	-5.2423
53	H	-1.0278	2.9844	-6.9453
54	H	-1.1790	-1.3062	-6.5794
55	H	-2.5638	-0.2273	-6.7783
56	H	-1.1510	-0.0469	-7.8294
57	C	1.8884	1.6478	-4.6541
58	C	1.8618	-0.0670	-6.3448
59	C	3.2806	1.5711	-4.5888
60	H	1.3765	2.3591	-4.0124
61	C	3.2565	-0.1413	-6.2933
62	H	1.3274	-0.7189	-7.0309
63	C	3.9687	0.6737	-5.4106
64	H	3.8278	2.2096	-3.9004
65	H	3.7855	-0.8391	-6.9361
66	H	5.0521	0.6096	-5.3617

step_007_t_SP
Energy (POTENTIAL) = -26981.8772155 Eh

	Atom	X	Y	Z
1	Cu	-0.3830	-0.2600	-1.7874
2	N	1.4648	-1.0570	-2.4369
3	N	2.5952	-0.6061	-1.8239
4	N	0.4567	1.6518	-1.4605
5	N	1.7716	1.6878	-1.1057
6	N	0.3702	-0.7046	0.2243
7	N	1.6372	-0.2706	0.4849
8	B	2.4877	0.3875	-0.6404
9	C	1.8342	-1.9841	-3.3157
10	C	3.2319	-2.1535	-3.3010
11	C	3.6711	-1.2521	-2.3340
12	C	0.0676	2.9061	-1.6803
13	C	1.1432	3.7958	-1.4908
14	C	2.2061	2.9704	-1.1243
15	C	-0.1165	-1.2037	1.3544
16	C	0.8310	-1.1066	2.3923
17	C	1.9324	-0.5047	1.7871
18	H	3.5658	0.6498	-0.2331
19	N	-2.0140	-0.4268	-2.5734
20	S	-3.6169	-0.4143	-3.0333
21	O	-3.8013	-1.4749	-4.0368
22	O	-4.0173	0.9632	-3.3583
23	C	-4.3470	-0.9023	-1.4887
24	C	-4.5160	-2.2622	-1.2129
25	C	-4.6855	0.0844	-0.5526
26	C	-5.0465	-2.6338	0.0209
27	H	-4.2449	-3.0099	-1.9504
28	C	-5.2120	-0.3087	0.6710
29	H	-4.5472	1.1339	-0.7882
30	C	-5.3989	-1.6691	0.9790
31	H	-5.1916	-3.6871	0.2431
32	H	-5.4837	0.4464	1.4032
33	C	-5.9725	-2.0689	2.3129
34	H	-5.4316	-1.5845	3.1333
35	H	-7.0200	-1.7542	2.3923
36	H	-5.9323	-3.1508	2.4632
37	Br	-1.8682	-1.9001	1.4087
38	Br	-1.7062	3.2922	-2.1732
39	Br	0.5607	-2.8990	-4.3542
40	Br	4.2681	-3.3177	-4.3530
41	Br	5.4396	-0.9135	-1.7909

42	Br	3.5842	-0.0659	2.5776
43	Br	0.6506	-1.6596	4.1834
44	Br	1.1477	5.6671	-1.6943
45	Br	3.9763	3.4734	-0.7342
46	H	-1.2594	0.4773	-4.2149
47	Si	-0.7642	0.8962	-5.5732
48	C	-1.3331	2.6641	-5.8903
49	C	-1.4792	-0.3032	-6.8342
50	C	1.1193	0.8066	-5.5231
51	H	-2.4157	2.7546	-5.7494
52	H	-0.8451	3.3704	-5.2114
53	H	-1.0951	2.9648	-6.9177
54	H	-1.1795	-1.3340	-6.6184
55	H	-2.5739	-0.2664	-6.8119
56	H	-1.1566	-0.0593	-7.8530
57	C	1.8482	1.6317	-4.6445
58	C	1.8453	-0.0843	-6.3348
59	C	3.2401	1.5620	-4.5669
60	H	1.3274	2.3403	-4.0072
61	C	3.2398	-0.1511	-6.2711
62	H	1.3204	-0.7393	-7.0252
63	C	3.9399	0.6678	-5.3822
64	H	3.7777	2.2033	-3.8737
65	H	3.7780	-0.8462	-6.9092
66	H	5.0231	0.6090	-5.3236

step_008_t_SP
Energy (POTENTIAL) = -26981.876638 Eh

	Atom	X	Y	Z
1	Cu	-0.3626	-0.4679	-1.9823
2	N	1.4875	-1.3257	-2.6052
3	N	2.6232	-0.8676	-2.0088
4	N	0.5126	1.3805	-1.5822
5	N	1.8046	1.3900	-1.1481
6	N	0.3727	-1.0106	0.0466
7	N	1.6672	-0.6666	0.3087
8	B	2.5172	0.0629	-0.7722
9	C	1.8482	-2.2679	-3.4696
10	C	3.2459	-2.4382	-3.4672
11	C	3.6945	-1.5238	-2.5163
12	C	0.1139	2.6468	-1.6751
13	C	1.1545	3.5201	-1.3098
14	C	2.2107	2.6716	-0.9824
15	C	-0.0946	-1.6278	1.1254
16	C	0.8938	-1.7065	2.1264
17	C	1.9996	-1.0797	1.5562
18	H	3.5961	0.2955	-0.3506
19	N	-1.9783	-0.6523	-2.8165
20	S	-3.6109	-0.4868	-3.1217
21	O	-4.0284	-1.6056	-3.9819
22	O	-3.8768	0.8933	-3.5591
23	C	-4.2538	-0.7184	-1.4803
24	C	-4.7375	-1.9728	-1.1017
25	C	-4.2532	0.3656	-0.5920
26	C	-5.2452	-2.1337	0.1868
27	H	-4.7299	-2.7975	-1.8060
28	C	-4.7602	0.1824	0.6879
29	H	-3.8751	1.3320	-0.9059
30	C	-5.2643	-1.0651	1.0979
31	H	-5.6399	-3.1002	0.4869
32	H	-4.7706	1.0173	1.3829
33	C	-5.7898	-1.2452	2.4977
34	H	-4.9670	-1.2244	3.2233
35	H	-6.4743	-0.4344	2.7685
36	H	-6.3157	-2.1966	2.6127
37	Br	-1.8764	-2.2395	1.1738
38	Br	-1.6244	3.0695	-2.2531
39	Br	0.5639	-3.1763	-4.5024
40	Br	4.2732	-3.5982	-4.5352
41	Br	5.4673	-1.1820	-1.9897

42	Br	3.6982	-0.8152	2.3255
43	Br	0.7529	-2.4806	3.8379
44	Br	1.1380	5.4013	-1.3095
45	Br	3.9410	3.1539	-0.4268
46	H	-1.3213	-0.2583	-4.5772
47	Si	-0.7894	0.4638	-5.8050
48	C	-1.9240	1.9263	-6.1349
49	C	-0.7833	-0.7460	-7.2481
50	C	0.9563	1.0339	-5.3763
51	H	-2.9431	1.5767	-6.3293
52	H	-1.9685	2.6021	-5.2764
53	H	-1.5832	2.4973	-7.0067
54	H	-0.1094	-1.5906	-7.0764
55	H	-1.7895	-1.1493	-7.4094
56	H	-0.4682	-0.2441	-8.1707
57	C	1.1876	2.2966	-4.7979
58	C	2.0717	0.2011	-5.5888
59	C	2.4712	2.7019	-4.4252
60	H	0.3569	2.9778	-4.6364
61	C	3.3572	0.5986	-5.2161
62	H	1.9400	-0.7733	-6.0515
63	C	3.5590	1.8482	-4.6243
64	H	2.6226	3.6824	-3.9831
65	H	4.2006	-0.0633	-5.3912
66	H	4.5580	2.1585	-4.3306

step_009_t_SP
Energy (POTENTIAL) = -26981.8752144 Eh

	Atom	X	Y	Z
1	Cu	-0.3625	-0.4457	-1.9598
2	N	1.4910	-1.3140	-2.6040
3	N	2.6317	-0.8563	-2.0174
4	N	0.5239	1.3953	-1.5633
5	N	1.8202	1.3998	-1.1423
6	N	0.3915	-1.0016	0.0534
7	N	1.6897	-0.6634	0.3058
8	B	2.5337	0.0699	-0.7770
9	C	1.8444	-2.2573	-3.4698
10	C	3.2420	-2.4291	-3.4785
11	C	3.6988	-1.5139	-2.5324
12	C	0.1298	2.6631	-1.6548
13	C	1.1776	3.5325	-1.3012
14	C	2.2334	2.6800	-0.9829
15	C	-0.0670	-1.6260	1.1319
16	C	0.9306	-1.7156	2.1226
17	C	2.0327	-1.0878	1.5467
18	H	3.6154	0.3000	-0.3607
19	N	-1.9540	-0.6173	-2.8561
20	S	-3.5935	-0.4534	-3.1219
21	O	-4.0243	-1.5596	-3.9927
22	O	-3.8744	0.9322	-3.5338
23	C	-4.2202	-0.7102	-1.4768
24	C	-4.6949	-1.9719	-1.1114
25	C	-4.2238	0.3626	-0.5760
26	C	-5.1962	-2.1516	0.1769
27	H	-4.6822	-2.7888	-1.8246
28	C	-4.7249	0.1611	0.7040
29	H	-3.8501	1.3343	-0.8788
30	C	-5.2208	-1.0937	1.1004
31	H	-5.5794	-3.1254	0.4685
32	H	-4.7339	0.9866	1.4101
33	C	-5.7802	-1.2863	2.4854
34	H	-5.1693	-0.7724	3.2344
35	H	-6.7923	-0.8674	2.5516
36	H	-5.8404	-2.3451	2.7519
37	Br	-1.8503	-2.2303	1.1924
38	Br	-1.6120	3.0907	-2.2190
39	Br	0.5502	-3.1663	-4.4907
40	Br	4.2599	-3.5916	-4.5533
41	Br	5.4760	-1.1714	-2.0209

42	Br	3.7399	-0.8364	2.3015
43	Br	0.8028	-2.5012	3.8298
44	Br	1.1686	5.4138	-1.3030
45	Br	3.9712	3.1562	-0.4452
46	H	-1.3541	-0.2460	-4.5339
47	Si	-0.8312	0.4670	-5.7887
48	C	-1.9685	1.9292	-6.1057
49	C	-0.8528	-0.7564	-7.2188
50	C	0.9187	1.0294	-5.3758
51	H	-2.9905	1.5808	-6.2850
52	H	-1.9996	2.6078	-5.2489
53	H	-1.6372	2.4958	-6.9842
54	H	-0.1760	-1.5995	-7.0530
55	H	-1.8622	-1.1594	-7.3591
56	H	-0.5539	-0.2604	-8.1501
57	C	1.1567	2.2894	-4.7938
58	C	2.0307	0.1960	-5.6043
59	C	2.4447	2.6924	-4.4349
60	H	0.3282	2.9699	-4.6196
61	C	3.3205	0.5916	-5.2450
62	H	1.8930	-0.7766	-6.0688
63	C	3.5296	1.8388	-4.6505
64	H	2.6021	3.6709	-3.9907
65	H	4.1616	-0.0699	-5.4316
66	H	4.5321	2.1473	-4.3675

step_010_t_SP
Energy (POTENTIAL) = -26981.8739238 Eh

	Atom	X	Y	Z
1	Cu	-0.3867	-0.4037	-1.9143
2	N	1.4843	-1.2766	-2.5914
3	N	2.6254	-0.8184	-2.0061
4	N	0.5058	1.4359	-1.5204
5	N	1.8067	1.4310	-1.1116
6	N	0.3825	-0.9811	0.0657
7	N	1.6817	-0.6458	0.3175
8	B	2.5235	0.0982	-0.7600
9	C	1.8369	-2.2253	-3.4502
10	C	3.2346	-2.4010	-3.4566
11	C	3.6923	-1.4819	-2.5153
12	C	0.1214	2.7074	-1.6098
13	C	1.1793	3.5687	-1.2666
14	C	2.2311	2.7081	-0.9570
15	C	-0.0730	-1.6165	1.1395
16	C	0.9278	-1.7168	2.1255
17	C	2.0284	-1.0836	1.5525
18	H	3.6040	0.3282	-0.3407
19	N	-1.9204	-0.5995	-2.9180
20	S	-3.5632	-0.4217	-3.1477
21	O	-4.0174	-1.5088	-4.0317
22	O	-3.8424	0.9731	-3.5322
23	C	-4.1852	-0.6982	-1.5021
24	C	-4.6673	-1.9611	-1.1527
25	C	-4.1936	0.3654	-0.5914
26	C	-5.1809	-2.1514	0.1295
27	H	-4.6529	-2.7710	-1.8739
28	C	-4.7079	0.1542	0.6823
29	H	-3.8153	1.3387	-0.8829
30	C	-5.2128	-1.1017	1.0620
31	H	-5.5701	-3.1266	0.4082
32	H	-4.7213	0.9737	1.3953
33	C	-5.7938	-1.3046	2.4370
34	H	-5.2068	-0.7801	3.1976
35	H	-6.8147	-0.9050	2.4840
36	H	-5.8398	-2.3645	2.7020
37	Br	-1.8565	-2.2176	1.2003
38	Br	-1.6210	3.1551	-2.1586
39	Br	0.5406	-3.1395	-4.4648
40	Br	4.2511	-3.5685	-4.5274
41	Br	5.4698	-1.1383	-2.0046

42	Br	3.7385	-0.8426	2.3036
43	Br	0.8045	-2.5187	3.8253
44	Br	1.1840	5.4502	-1.2644
45	Br	3.9763	3.1735	-0.4332
46	H	-1.3546	-0.2814	-4.5109
47	Si	-0.8334	0.4365	-5.7882
48	C	-1.9687	1.9061	-6.0707
49	C	-0.8832	-0.7837	-7.2186
50	C	0.9182	0.9843	-5.3764
51	H	-2.9891	1.5649	-6.2700
52	H	-2.0074	2.5562	-5.1927
53	H	-1.6274	2.4984	-6.9281
54	H	-0.2047	-1.6282	-7.0682
55	H	-1.8959	-1.1832	-7.3428
56	H	-0.5998	-0.2832	-8.1525
57	C	1.1557	2.2263	-4.7559
58	C	2.0307	0.1632	-5.6439
59	C	2.4459	2.6246	-4.4012
60	H	0.3254	2.8957	-4.5509
61	C	3.3226	0.5550	-5.2882
62	H	1.8921	-0.7963	-6.1344
63	C	3.5322	1.7845	-4.6582
64	H	2.6042	3.5894	-3.9288
65	H	4.1648	-0.0962	-5.5038
66	H	4.5366	2.0894	-4.3780

step_011_t_SP
Energy (POTENTIAL) = -26981.872936 Eh

	Atom	X	Y	Z
1	Cu	-0.4715	-0.3841	-1.8726
2	N	1.4379	-1.2431	-2.5649
3	N	2.5659	-0.7808	-1.9578
4	N	0.4104	1.4640	-1.4802
5	N	1.7131	1.4607	-1.0746
6	N	0.3126	-1.0132	0.0713
7	N	1.5907	-0.6217	0.3520
8	B	2.4352	0.1352	-0.7137
9	C	1.8101	-2.1963	-3.4087
10	C	3.2081	-2.3726	-3.3835
11	C	3.6447	-1.4477	-2.4384
12	C	0.0415	2.7371	-1.6145
13	C	1.1090	3.5979	-1.3025
14	C	2.1519	2.7372	-0.9655
15	C	-0.1162	-1.7208	1.1117
16	C	0.8775	-1.8041	2.1063
17	C	1.9474	-1.0894	1.5730
18	H	3.5071	0.3763	-0.2791
19	N	-1.9075	-0.5896	-3.0233
20	S	-3.5549	-0.4046	-3.1986
21	O	-4.0446	-1.4837	-4.0750
22	O	-3.8440	0.9930	-3.5698
23	C	-4.1379	-0.6859	-1.5369
24	C	-4.7372	-1.9037	-1.2154
25	C	-4.0098	0.3370	-0.5886
26	C	-5.2315	-2.0888	0.0766
27	H	-4.8301	-2.6812	-1.9657
28	C	-4.5015	0.1299	0.6940
29	H	-3.5463	1.2793	-0.8577
30	C	-5.1235	-1.0811	1.0464
31	H	-5.7165	-3.0271	0.3307
32	H	-4.4102	0.9192	1.4351
33	C	-5.6457	-1.2825	2.4452
34	H	-4.8243	-1.2703	3.1719
35	H	-6.3321	-0.4763	2.7271
36	H	-6.1740	-2.2342	2.5466
37	Br	-1.8473	-2.4615	1.1228
38	Br	-1.6907	3.1940	-2.1858
39	Br	0.5354	-3.1156	-4.4471
40	Br	4.2479	-3.5452	-4.4264
41	Br	5.4109	-1.0971	-1.8930

42	Br	3.6321	-0.7843	2.3572
43	Br	0.7789	-2.6725	3.7748
44	Br	1.1382	5.4779	-1.3709
45	Br	3.9039	3.2031	-0.4664
46	H	-1.3588	-0.2970	-4.5194
47	Si	-0.8121	0.4211	-5.8226
48	C	-1.9557	1.8825	-6.1069
49	C	-0.8489	-0.8119	-7.2405
50	C	0.9283	0.9677	-5.3774
51	H	-2.9695	1.5353	-6.3278
52	H	-2.0142	2.5216	-5.2221
53	H	-1.6036	2.4864	-6.9521
54	H	-0.1617	-1.6478	-7.0837
55	H	-1.8576	-1.2216	-7.3629
56	H	-0.5698	-0.3141	-8.1774
57	C	1.1516	2.2047	-4.7409
58	C	2.0465	0.1504	-5.6334
59	C	2.4352	2.6023	-4.3629
60	H	0.3164	2.8706	-4.5435
61	C	3.3311	0.5416	-5.2529
62	H	1.9173	-0.8059	-6.1326
63	C	3.5274	1.7664	-4.6096
64	H	2.5841	3.5629	-3.8794
65	H	4.1781	-0.1066	-5.4585
66	H	4.5261	2.0707	-4.3093

step_012_t_SP
Energy (POTENTIAL) = -26981.8728133 Eh

	Atom	X	Y	Z
1	Cu	-0.5067	-0.3800	-1.8045
2	N	1.4081	-1.2488	-2.5715
3	N	2.5467	-0.7846	-1.9852
4	N	0.4034	1.4740	-1.4694
5	N	1.7153	1.4638	-1.0944
6	N	0.3279	-0.9948	0.0974
7	N	1.6163	-0.6129	0.3425
8	B	2.4401	0.1370	-0.7441
9	C	1.7683	-2.1999	-3.4224
10	C	3.1670	-2.3739	-3.4227
11	C	3.6186	-1.4491	-2.4850
12	C	0.0443	2.7486	-1.6155
13	C	1.1265	3.6033	-1.3392
14	C	2.1684	2.7375	-1.0131
15	C	-0.0791	-1.6947	1.1528
16	C	0.9407	-1.7833	2.1195
17	C	2.0016	-1.0795	1.5548
18	H	3.5202	0.3777	-0.3303
19	N	-1.8887	-0.5713	-3.0378
20	S	-3.5373	-0.3760	-3.1742
21	O	-4.0429	-1.4425	-4.0590
22	O	-3.8378	1.0259	-3.5230
23	C	-4.1134	-0.6771	-1.5113
24	C	-4.6839	-1.9095	-1.1947
25	C	-4.0174	0.3468	-0.5608
26	C	-5.1789	-2.1105	0.0950
27	H	-4.7557	-2.6871	-1.9473
28	C	-4.5101	0.1246	0.7194
29	H	-3.5796	1.3021	-0.8271
30	C	-5.1019	-1.1025	1.0671
31	H	-5.6403	-3.0618	0.3450
32	H	-4.4437	0.9152	1.4620
33	C	-5.6256	-1.3208	2.4632
34	H	-4.8069	-1.3042	3.1930
35	H	-6.3231	-0.5258	2.7495
36	H	-6.1420	-2.2800	2.5558
37	Br	-1.8168	-2.4149	1.2201
38	Br	-1.6934	3.2131	-2.1630
39	Br	0.4798	-3.1236	-4.4411
40	Br	4.1907	-3.5429	-4.4857
41	Br	5.3939	-1.0990	-1.9682

42	Br	3.7083	-0.7858	2.2938
43	Br	0.8814	-2.6474	3.7918
44	Br	1.1730	5.4819	-1.4363
45	Br	3.9355	3.1942	-0.5611
46	H	-1.3702	-0.2845	-4.4377
47	Si	-0.8328	0.4227	-5.8018
48	C	-1.9873	1.8785	-6.0671
49	C	-0.9194	-0.8330	-7.1963
50	C	0.9130	0.9653	-5.3866
51	H	-3.0057	1.5263	-6.2559
52	H	-2.0228	2.5282	-5.1890
53	H	-1.6589	2.4719	-6.9293
54	H	-0.1646	-1.6183	-7.1030
55	H	-1.9055	-1.3095	-7.2191
56	H	-0.7676	-0.3280	-8.1587
57	C	1.1453	2.2033	-4.7546
58	C	2.0264	0.1453	-5.6559
59	C	2.4345	2.6010	-4.3976
60	H	0.3131	2.8692	-4.5446
61	C	3.3166	0.5381	-5.2973
62	H	1.8893	-0.8129	-6.1490
63	C	3.5228	1.7650	-4.6611
64	H	2.5912	3.5620	-3.9172
65	H	4.1602	-0.1109	-5.5142
66	H	4.5261	2.0701	-4.3776

step_013_t_SP
Energy (POTENTIAL) = -26981.873228 Eh

	Atom	X	Y	Z
1	Cu	-0.5690	-0.3154	-1.6409
2	N	1.4190	-1.0898	-2.4475
3	N	2.5251	-0.6011	-1.8205
4	N	0.2680	1.5781	-1.2947
5	N	1.5920	1.6091	-0.9671
6	N	0.2758	-0.8970	0.2334
7	N	1.5428	-0.4575	0.4944
8	B	2.3582	0.3103	-0.5834
9	C	1.8435	-1.9465	-3.3675
10	C	3.2510	-2.0319	-3.3686
11	C	3.6395	-1.1530	-2.3618
12	C	-0.1119	2.8350	-1.5270
13	C	0.9729	3.7172	-1.3681
14	C	2.0351	2.8880	-1.0106
15	C	-0.1336	-1.5714	1.3049
16	C	0.8646	-1.5876	2.2970
17	C	1.9134	-0.8656	1.7323
18	H	3.4190	0.5980	-0.1496
19	N	-1.8490	-0.4901	-2.9883
20	S	-3.5011	-0.4129	-3.1666
21	O	-3.8957	-1.5081	-4.0745
22	O	-3.9018	0.9635	-3.5214
23	C	-4.1193	-0.7708	-1.5288
24	C	-4.5723	-2.0556	-1.2314
25	C	-4.1774	0.2582	-0.5815
26	C	-5.1008	-2.3071	0.0361
27	H	-4.5263	-2.8380	-1.9811
28	C	-4.7028	-0.0129	0.6763
29	H	-3.8300	1.2535	-0.8338
30	C	-5.1772	-1.2953	1.0043
31	H	-5.4662	-3.3028	0.2715
32	H	-4.7523	0.7808	1.4170
33	C	-5.7504	-1.5637	2.3722
34	H	-5.0488	-1.2611	3.1578
35	H	-6.6715	-0.9896	2.5282
36	H	-5.9845	-2.6226	2.5110
37	Br	-1.8537	-2.3330	1.3625
38	Br	-1.8780	3.2459	-2.0255
39	Br	0.6257	-2.8927	-4.4507
40	Br	4.3521	-3.0796	-4.4778
41	Br	5.3873	-0.7356	-1.8021

42	Br	3.5884	-0.4837	2.5026
43	Br	0.7947	-2.3958	3.9964
44	Br	0.9892	5.5853	-1.5949
45	Br	3.8130	3.3915	-0.6576
46	H	-1.3252	-0.1211	-4.2557
47	Si	-0.8000	0.5759	-5.7032
48	C	-1.6952	2.2259	-5.7629
49	C	-1.3061	-0.6036	-7.0731
50	C	1.0530	0.7713	-5.5255
51	H	-2.7556	2.0960	-5.5292
52	H	-1.2745	2.9323	-5.0417
53	H	-1.6010	2.6655	-6.7639
54	H	-0.8189	-1.5775	-6.9679
55	H	-2.3894	-0.7638	-7.0492
56	H	-1.0460	-0.1941	-8.0571
57	C	1.5884	1.6674	-4.5784
58	C	1.9526	0.0253	-6.3116
59	C	2.9665	1.8114	-4.4201
60	H	0.9260	2.2582	-3.9531
61	C	3.3327	0.1770	-6.1651
62	H	1.5760	-0.6785	-7.0487
63	C	3.8421	1.0664	-5.2155
64	H	3.3582	2.5034	-3.6805
65	H	4.0097	-0.4021	-6.7861
66	H	4.9158	1.1777	-5.0943

step_014_t_SP
Energy (POTENTIAL) = -26981.8761449 Eh

	Atom	X	Y	Z
1	Cu	-0.5507	-0.3911	-1.7659
2	N	1.3767	-1.2980	-2.5789
3	N	2.5184	-0.8274	-2.0031
4	N	0.4016	1.4484	-1.4781
5	N	1.7092	1.4228	-1.0891
6	N	0.2973	-1.0169	0.0970
7	N	1.5966	-0.6641	0.3294
8	B	2.4224	0.0866	-0.7556
9	C	1.7364	-2.2419	-3.4379
10	C	3.1371	-2.4035	-3.4553
11	C	3.5910	-1.4794	-2.5184
12	C	0.0559	2.7276	-1.6191
13	C	1.1419	3.5694	-1.3200
14	C	2.1720	2.6917	-0.9895
15	C	-0.1086	-1.7278	1.1469
16	C	0.9231	-1.8512	2.0962
17	C	1.9895	-1.1589	1.5275
18	H	3.5056	0.3145	-0.3431
19	N	-1.8759	-0.5543	-3.0783
20	S	-3.5227	-0.3293	-3.1568
21	O	-4.0652	-1.3800	-4.0412
22	O	-3.8214	1.0782	-3.4889
23	C	-4.0875	-0.6349	-1.4887
24	C	-4.7000	-1.8498	-1.1861
25	C	-3.9609	0.3744	-0.5267
26	C	-5.2006	-2.0507	0.1020
27	H	-4.7983	-2.6153	-1.9480
28	C	-4.4598	0.1532	0.7517
29	H	-3.4946	1.3193	-0.7824
30	C	-5.0923	-1.0574	1.0854
31	H	-5.6923	-2.9898	0.3404
32	H	-4.3677	0.9328	1.5033
33	C	-5.6253	-1.2742	2.4786
34	H	-4.8118	-1.2595	3.2141
35	H	-6.3236	-0.4781	2.7603
36	H	-6.1449	-2.2320	2.5675
37	Br	-1.8575	-2.4157	1.2327
38	Br	-1.6663	3.2112	-2.1937
39	Br	0.4472	-3.1802	-4.4457
40	Br	4.1605	-3.5615	-4.5311
41	Br	5.3695	-1.1157	-2.0221

42	Br	3.7125	-0.9142	2.2455
43	Br	0.8708	-2.7419	3.7542
44	Br	1.2068	5.4479	-1.4002
45	Br	3.9352	3.1305	-0.5052
46	H	-1.4251	-0.3374	-4.3000
47	Si	-0.7948	0.3921	-5.8542
48	C	-2.0079	1.8011	-6.1289
49	C	-0.8021	-0.8798	-7.2381
50	C	0.9156	0.9920	-5.3892
51	H	-2.9888	1.4081	-6.4132
52	H	-2.1433	2.3930	-5.2204
53	H	-1.6540	2.4608	-6.9313
54	H	-0.0017	-1.6167	-7.1312
55	H	-1.7569	-1.4155	-7.2669
56	H	-0.6722	-0.3734	-8.2036
57	C	1.0946	2.2572	-4.7925
58	C	2.0582	0.1893	-5.5833
59	C	2.3597	2.6999	-4.4047
60	H	0.2391	2.9083	-4.6364
61	C	3.3232	0.6266	-5.1903
62	H	1.9625	-0.7888	-6.0463
63	C	3.4766	1.8818	-4.5950
64	H	2.4749	3.6824	-3.9571
65	H	4.1895	-0.0085	-5.3510
66	H	4.4611	2.2222	-4.2873

step_015_t_SP
Energy (POTENTIAL) = -26981.8813047 Eh

	Atom	X	Y	Z
1	Cu	-0.6862	-0.3576	-1.5029
2	N	1.2411	-1.1915	-2.4528
3	N	2.3898	-0.6950	-1.9140
4	N	0.2151	1.5349	-1.3517
5	N	1.5477	1.5565	-1.0597
6	N	0.2438	-0.8231	0.3323
7	N	1.5566	-0.4604	0.4579
8	B	2.3152	0.2517	-0.6996
9	C	1.6007	-2.0596	-3.3903
10	C	3.0048	-2.1491	-3.4873
11	C	3.4639	-1.2566	-2.5231
12	C	-0.1703	2.7966	-1.5456
13	C	0.9202	3.6738	-1.3956
14	C	1.9899	2.8366	-1.0822
15	C	-0.1246	-1.3621	1.4953
16	C	0.9495	-1.3720	2.4032
17	C	1.9967	-0.7866	1.6961
18	H	3.4050	0.5316	-0.3406
19	N	-1.8865	-0.5517	-2.9380
20	S	-3.5314	-0.3956	-3.1250
21	O	-3.9541	-1.4540	-4.0653
22	O	-3.9040	0.9965	-3.4486
23	C	-4.1669	-0.7811	-1.4990
24	C	-4.5219	-2.0965	-1.1976
25	C	-4.3283	0.2467	-0.5646
26	C	-5.0510	-2.3802	0.0612
27	H	-4.3983	-2.8793	-1.9383
28	C	-4.8557	-0.0565	0.6871
29	H	-4.0581	1.2647	-0.8211
30	C	-5.2302	-1.3695	1.0180
31	H	-5.3350	-3.4011	0.3011
32	H	-4.9838	0.7366	1.4187
33	C	-5.8256	-1.6769	2.3691
34	H	-5.3182	-1.1213	3.1645
35	H	-6.8837	-1.3884	2.3997
36	H	-5.7685	-2.7445	2.6002
37	Br	-1.8857	-1.9565	1.7915
38	Br	-1.9520	3.2203	-1.9654
39	Br	0.3136	-3.0000	-4.3982
40	Br	4.0260	-3.2215	-4.6485
41	Br	5.2464	-0.8397	-2.0887

42	Br	3.7532	-0.4802	2.2987
43	Br	0.9645	-2.0176	4.1714
44	Br	0.9319	5.5462	-1.5848
45	Br	3.7719	3.3346	-0.7420
46	H	-1.3927	-0.2786	-4.0198
47	Si	-0.6591	0.5929	-5.7117
48	C	-1.5703	2.2415	-5.6701
49	C	-1.1462	-0.4951	-7.1700
50	C	1.1931	0.7958	-5.5210
51	H	-2.6277	2.0949	-5.4317
52	H	-1.1473	2.9118	-4.9167
53	H	-1.4922	2.7368	-6.6470
54	H	-0.6343	-1.4617	-7.1404
55	H	-2.2250	-0.6847	-7.1562
56	H	-0.9006	-0.0062	-8.1218
57	C	1.7216	1.7289	-4.6033
58	C	2.1047	0.0210	-6.2676
59	C	3.0975	1.8885	-4.4465
60	H	1.0531	2.3398	-4.0039
61	C	3.4830	0.1847	-6.1173
62	H	1.7384	-0.7118	-6.9815
63	C	3.9832	1.1173	-5.2055
64	H	3.4809	2.6144	-3.7359
65	H	4.1663	-0.4177	-6.7084
66	H	5.0553	1.2428	-5.0856

step_016_t_SP
Energy (POTENTIAL) = -26981.8895993 Eh

	Atom	X	Y	Z
1	Cu	-0.7103	-0.3708	-1.5307
2	N	1.2473	-1.1661	-2.4608
3	N	2.3761	-0.6729	-1.8773
4	N	0.1589	1.5154	-1.3391
5	N	1.4834	1.5509	-1.0134
6	N	0.1922	-0.9014	0.3003
7	N	1.4861	-0.4930	0.4748
8	B	2.2588	0.2550	-0.6490
9	C	1.6410	-2.0198	-3.3978
10	C	3.0478	-2.1032	-3.4497
11	C	3.4714	-1.2228	-2.4587
12	C	-0.2265	2.7710	-1.5732
13	C	0.8554	3.6563	-1.4134
14	C	1.9202	2.8319	-1.0533
15	C	-0.1579	-1.5687	1.4016
16	C	0.9068	-1.6080	2.3199
17	C	1.9305	-0.9112	1.6834
18	H	3.3354	0.5447	-0.2595
19	N	-1.9317	-0.5956	-2.9447
20	S	-3.5836	-0.4569	-3.1040
21	O	-4.0209	-1.5513	-3.9934
22	O	-3.9635	0.9214	-3.4725
23	C	-4.1762	-0.7846	-1.4500
24	C	-4.6231	-2.0651	-1.1273
25	C	-4.2254	0.2578	-0.5183
26	C	-5.1279	-2.3008	0.1526
27	H	-4.5879	-2.8580	-1.8666
28	C	-4.7264	0.0022	0.7538
29	H	-3.8898	1.2517	-0.7908
30	C	-5.1904	-1.2764	1.1080
31	H	-5.4849	-3.2946	0.4086
32	H	-4.7660	0.8066	1.4835
33	C	-5.7425	-1.5280	2.4881
34	H	-5.0527	-1.1697	3.2602
35	H	-6.6887	-0.9927	2.6320
36	H	-5.9289	-2.5917	2.6601
37	Br	-1.8766	-2.3077	1.6106
38	Br	-1.9923	3.1778	-2.0650
39	Br	0.3921	-2.9536	-4.4582
40	Br	4.1099	-3.1562	-4.5911
41	Br	5.2370	-0.8063	-1.9603

42	Br	3.6628	-0.5754	2.3373
43	Br	0.9401	-2.4144	4.0202
44	Br	0.8645	5.5231	-1.6476
45	Br	3.6901	3.3464	-0.6790
46	H	-1.4990	-0.4294	-3.9645
47	Si	-0.6309	0.5795	-5.8380
48	C	-1.5942	2.2018	-5.7740
49	C	-1.0055	-0.4383	-7.3825
50	C	1.2065	0.8270	-5.5654
51	H	-2.6491	2.0221	-5.5460
52	H	-1.1973	2.8707	-5.0056
53	H	-1.5267	2.7176	-6.7412
54	H	-0.5157	-1.4165	-7.3508
55	H	-2.0842	-0.6067	-7.4735
56	H	-0.6679	0.0824	-8.2883
57	C	1.6805	1.7538	-4.6118
58	C	2.1636	0.0726	-6.2760
59	C	3.0456	1.9280	-4.3888
60	H	0.9779	2.3487	-4.0356
61	C	3.5310	0.2494	-6.0582
62	H	1.8404	-0.6557	-7.0150
63	C	3.9766	1.1769	-5.1132
64	H	3.3859	2.6509	-3.6538
65	H	4.2484	-0.3384	-6.6231
66	H	5.0402	1.3136	-4.9409

step_017_t_SP
Energy (POTENTIAL) = -26981.8928414 Eh

	Atom	X	Y	Z
1	Cu	-0.6312	-0.4042	-1.8023
2	N	1.4338	-1.1039	-2.5449
3	N	2.4784	-0.6356	-1.8028
4	N	0.1819	1.4585	-1.3799
5	N	1.4408	1.4865	-0.8550
6	N	0.1136	-1.1811	-0.0064
7	N	1.3171	-0.6983	0.4250
8	B	2.1977	0.1767	-0.5152
9	C	1.9406	-1.9456	-3.4354
10	C	3.3419	-2.0409	-3.3042
11	C	3.6366	-1.1875	-2.2462
12	C	-0.1934	2.7249	-1.5747
13	C	0.8249	3.6083	-1.1748
14	C	1.8459	2.7716	-0.7269
15	C	-0.2996	-2.0739	0.8921
16	C	0.6253	-2.1776	1.9483
17	C	1.6384	-1.2869	1.6019
18	H	3.2141	0.4403	0.0258
19	N	-1.9469	-0.6738	-3.1126
20	S	-3.6089	-0.4969	-3.1074
21	O	-4.1854	-1.6264	-3.8607
22	O	-3.9550	0.8700	-3.5442
23	C	-4.0300	-0.6815	-1.3800
24	C	-4.9135	-1.6944	-1.0075
25	C	-3.5296	0.2215	-0.4377
26	C	-5.2962	-1.7983	0.3304
27	H	-5.2966	-2.3832	-1.7522
28	C	-3.9118	0.0941	0.8944
29	H	-2.8550	1.0150	-0.7358
30	C	-4.8028	-0.9124	1.2994
31	H	-5.9910	-2.5802	0.6244
32	H	-3.5199	0.7919	1.6292
33	C	-5.1880	-1.0605	2.7487
34	H	-4.5100	-1.7594	3.2553
35	H	-5.1279	-0.1053	3.2786
36	H	-6.2027	-1.4554	2.8558
37	Br	-1.8981	-3.0378	0.6622
38	Br	-1.8609	3.1531	-2.3276
39	Br	0.8287	-2.8677	-4.6467
40	Br	4.5414	-3.0463	-4.3496
41	Br	5.3265	-0.7861	-1.5210

42	Br	3.2126	-0.9107	2.5610
43	Br	0.5182	-3.2696	3.4777
44	Br	0.8185	5.4883	-1.2459
45	Br	3.5353	3.2786	-0.0759
46	H	-1.6399	-0.6754	-4.1483
47	Si	-0.7629	0.3567	-6.2362
48	C	-1.9697	1.8091	-6.2830
49	C	-0.6950	-0.6037	-7.8611
50	C	0.9401	0.8701	-5.6453
51	H	-2.9557	1.4766	-6.6235
52	H	-2.0987	2.2564	-5.2937
53	H	-1.6132	2.5867	-6.9717
54	H	-0.0934	-1.5140	-7.7728
55	H	-1.7023	-0.8984	-8.1747
56	H	-0.2589	0.0117	-8.6592
57	C	1.1002	1.9217	-4.7184
58	C	2.1013	0.1829	-6.0588
59	C	2.3581	2.2676	-4.2243
60	H	0.2323	2.4827	-4.3851
61	C	3.3604	0.5262	-5.5656
62	H	2.0235	-0.6338	-6.7712
63	C	3.4931	1.5664	-4.6411
64	H	2.4534	3.0864	-3.5179
65	H	4.2379	-0.0205	-5.8987
66	H	4.4726	1.8323	-4.2539

step_000_s_SP
Energy (POTENTIAL) = -26981.8432989 Eh

	Atom	X	Y	Z
1	Cu	-0.5697	-0.4198	-1.8271
2	N	1.2803	-1.0765	-2.6005
3	N	2.4127	-0.6096	-2.0020
4	N	0.2098	1.5422	-1.6242
5	N	1.5186	1.6510	-1.2610
6	N	0.2823	-0.7859	0.1250
7	N	1.5375	-0.2997	0.3401
8	B	2.3125	0.3885	-0.8211
9	C	1.6544	-1.9649	-3.5166
10	C	3.0553	-2.1028	-3.5324
11	C	3.4927	-1.2162	-2.5504
12	C	-0.2461	2.7706	-1.8541
13	C	0.7770	3.7199	-1.6592
14	C	1.8804	2.9567	-1.2778
15	C	-0.1435	-1.3062	1.2713
16	C	0.8366	-1.1698	2.2733
17	C	1.8893	-0.5214	1.6292
18	H	3.3933	0.7002	-0.4590
19	N	-2.2475	-0.8022	-2.3864
20	S	-3.8473	-0.8820	-2.8363
21	O	-4.0121	-2.0641	-3.6956
22	O	-4.2403	0.4434	-3.3389
23	C	-4.5775	-1.1762	-1.2492
24	C	-4.7460	-2.4935	-0.8088
25	C	-4.8912	-0.0820	-0.4310
26	C	-5.2507	-2.7107	0.4707
27	H	-4.4912	-3.3257	-1.4560
28	C	-5.3938	-0.3224	0.8412
29	H	-4.7520	0.9308	-0.7929
30	C	-5.5805	-1.6348	1.3130
31	H	-5.3958	-3.7287	0.8206
32	H	-5.6476	0.5164	1.4830
33	C	-6.1466	-1.8717	2.6877
34	H	-5.6989	-1.1936	3.4214
35	H	-7.2274	-1.6823	2.6920
36	H	-5.9866	-2.9012	3.0188
37	Br	-1.8598	-2.0764	1.3898
38	Br	-2.0404	3.0500	-2.3395
39	Br	0.3829	-2.8506	-4.5835
40	Br	4.0946	-3.2357	-4.6145
41	Br	5.2617	-0.8689	-2.0179

42	Br	3.5480	-0.0095	2.3567
43	Br	0.7473	-1.7323	4.0680
44	Br	0.6781	5.5876	-1.8727
45	Br	3.6136	3.5613	-0.8668
46	H	-0.8867	0.6085	-4.2725
47	Si	-0.3594	1.0768	-5.5817
48	C	-0.8007	2.8985	-5.8003
49	C	-1.1222	0.0364	-6.9540
50	C	1.5212	0.9068	-5.5629
51	H	-1.8834	3.0568	-5.7491
52	H	-0.3340	3.5181	-5.0274
53	H	-0.4516	3.2625	-6.7742
54	H	-0.8525	-1.0203	-6.8586
55	H	-2.2159	0.1024	-6.9141
56	H	-0.8037	0.3798	-7.9450
57	C	2.2875	1.6720	-4.6624
58	C	2.2100	0.0506	-6.4404
59	C	3.6797	1.5792	-4.6291
60	H	1.7919	2.3567	-3.9797
61	C	3.6050	-0.0397	-6.4203
62	H	1.6564	-0.5555	-7.1529
63	C	4.3426	0.7212	-5.5114
64	H	4.2472	2.1752	-3.9195
65	H	4.1142	-0.7070	-7.1097
66	H	5.4262	0.6462	-5.4890

step_001_s_SP
Energy (POTENTIAL) = -26981.8424913 Eh

	Atom	X	Y	Z
1	Cu	-0.5387	-0.4010	-1.8062
2	N	1.3154	-1.0663	-2.5702
3	N	2.4459	-0.5986	-1.9689
4	N	0.2419	1.5583	-1.5908
5	N	1.5516	1.6620	-1.2290
6	N	0.3017	-0.7755	0.1471
7	N	1.5569	-0.2923	0.3691
8	B	2.3403	0.3967	-0.7864
9	C	1.6926	-1.9529	-3.4868
10	C	3.0939	-2.0879	-3.5006
11	C	3.5280	-1.2017	-2.5169
12	C	-0.2096	2.7887	-1.8198
13	C	0.8177	3.7338	-1.6269
14	C	1.9188	2.9662	-1.2472
15	C	-0.1304	-1.2984	1.2896
16	C	0.8454	-1.1671	2.2965
17	C	1.9023	-0.5188	1.6592
18	H	3.4194	0.7058	-0.4169
19	N	-2.2055	-0.7722	-2.4051
20	S	-3.8016	-0.8334	-2.8724
21	O	-3.9666	-2.0021	-3.7499
22	O	-4.1848	0.5008	-3.3585
23	C	-4.5437	-1.1461	-1.2937
24	C	-4.7119	-2.4685	-0.8685
25	C	-4.8681	-0.0617	-0.4667
26	C	-5.2274	-2.7007	0.4040
27	H	-4.4489	-3.2932	-1.5220
28	C	-5.3815	-0.3170	0.7983
29	H	-4.7290	0.9554	-0.8165
30	C	-5.5682	-1.6349	1.2547
31	H	-5.3724	-3.7228	0.7418
32	H	-5.6438	0.5141	1.4465
33	C	-6.1439	-1.8871	2.6226
34	H	-5.6863	-1.2308	3.3701
35	H	-7.2209	-1.6781	2.6274
36	H	-6.0030	-2.9251	2.9355
37	Br	-1.8490	-2.0654	1.3965
38	Br	-2.0037	3.0767	-2.3010
39	Br	0.4251	-2.8409	-4.5563
40	Br	4.1375	-3.2171	-4.5827
41	Br	5.2959	-0.8463	-1.9855

42	Br	3.5586	-0.0130	2.3964
43	Br	0.7466	-1.7349	4.0890
44	Br	0.7250	5.6020	-1.8387
45	Br	3.6548	3.5640	-0.8376
46	H	-0.9265	0.5977	-4.2360
47	Si	-0.4155	1.0517	-5.5572
48	C	-0.8619	2.8700	-5.7931
49	C	-1.1952	-0.0055	-6.9071
50	C	1.4655	0.8823	-5.5573
51	H	-1.9445	3.0266	-5.7331
52	H	-0.3890	3.5004	-5.0328
53	H	-0.5234	3.2225	-6.7749
54	H	-0.9299	-1.0621	-6.7988
55	H	-2.2881	0.0666	-6.8573
56	H	-0.8849	0.3216	-7.9061
57	C	2.2408	1.6407	-4.6588
58	C	2.1456	0.0310	-6.4462
59	C	3.6332	1.5468	-4.6389
60	H	1.7528	2.3204	-3.9659
61	C	3.5406	-0.0610	-6.4392
62	H	1.5847	-0.5706	-7.1568
63	C	4.2873	0.6938	-5.5325
64	H	4.2077	2.1379	-3.9309
65	H	4.0427	-0.7253	-7.1367
66	H	5.3709	0.6171	-5.5198

step_002_s_SP
Energy (POTENTIAL) = -26981.8414766 Eh

	Atom	X	Y	Z
1	Cu	-0.5093	-0.3916	-1.7864
2	N	1.3443	-1.0722	-2.5451
3	N	2.4751	-0.6106	-1.9400
4	N	0.2820	1.5638	-1.5583
5	N	1.5935	1.6530	-1.1988
6	N	0.3186	-0.7828	0.1666
7	N	1.5752	-0.3059	0.3955
8	B	2.3695	0.3802	-0.7541
9	C	1.7208	-1.9519	-3.4687
10	C	3.1221	-2.0868	-3.4844
11	C	3.5569	-1.2089	-2.4936
12	C	-0.1536	2.7991	-1.7938
13	C	0.8865	3.7316	-1.6086
14	C	1.9780	2.9519	-1.2261
15	C	-0.1224	-1.3033	1.3069
16	C	0.8484	-1.1767	2.3191
17	C	1.9122	-0.5340	1.6877
18	H	3.4487	0.6804	-0.3777
19	N	-2.1678	-0.7360	-2.4249
20	S	-3.7618	-0.7739	-2.9028
21	O	-3.9333	-1.9277	-3.7987
22	O	-4.1343	0.5702	-3.3693
23	C	-4.5106	-1.1041	-1.3300
24	C	-4.6860	-2.4309	-0.9221
25	C	-4.8335	-0.0289	-0.4904
26	C	-5.2078	-2.6769	0.3454
27	H	-4.4238	-3.2487	-1.5847
28	C	-5.3529	-0.2979	0.7692
29	H	-4.6885	0.9919	-0.8267
30	C	-5.5472	-1.6206	1.2082
31	H	-5.3584	-3.7026	0.6696
32	H	-5.6141	0.5262	1.4269
33	C	-6.1278	-1.8873	2.5714
34	H	-5.6655	-1.2459	3.3289
35	H	-7.2030	-1.6686	2.5776
36	H	-5.9966	-2.9308	2.8696
37	Br	-1.8456	-2.0616	1.4048
38	Br	-1.9438	3.1117	-2.2764
39	Br	0.4534	-2.8342	-4.5431
40	Br	4.1658	-3.2047	-4.5780
41	Br	5.3254	-0.8557	-1.9625

42	Br	3.5667	-0.0369	2.4347
43	Br	0.7368	-1.7435	4.1111
44	Br	0.8202	5.5992	-1.8335
45	Br	3.7232	3.5289	-0.8258
46	H	-0.9839	0.5680	-4.2274
47	Si	-0.4814	1.0128	-5.5562
48	C	-0.9644	2.8175	-5.8215
49	C	-1.2402	-0.0771	-6.8919
50	C	1.4022	0.8754	-5.5470
51	H	-2.0490	2.9543	-5.7483
52	H	-0.4928	3.4735	-5.0825
53	H	-0.6474	3.1546	-6.8159
54	H	-0.9420	-1.1244	-6.7798
55	H	-2.3342	-0.0376	-6.8334
56	H	-0.9480	0.2529	-7.8955
57	C	2.1592	1.6345	-4.6336
58	C	2.1015	0.0429	-6.4388
59	C	3.5526	1.5602	-4.6032
60	H	1.6563	2.2984	-3.9360
61	C	3.4976	-0.0297	-6.4208
62	H	1.5548	-0.5599	-7.1595
63	C	4.2261	0.7260	-5.5002
64	H	4.1128	2.1511	-3.8837
65	H	4.0148	-0.6801	-7.1206
66	H	5.3105	0.6645	-5.4792

step_003_s_SP
Energy (POTENTIAL) = -26981.8410647 Eh

	Atom	X	Y	Z
1	Cu	-0.4614	-0.3876	-1.8027
2	N	1.3964	-1.0611	-2.5426
3	N	2.5215	-0.5975	-1.9277
4	N	0.3125	1.5527	-1.4977
5	N	1.6225	1.6437	-1.1332
6	N	0.3546	-0.8221	0.1674
7	N	1.6101	-0.3496	0.4116
8	B	2.4059	0.3649	-0.7194
9	C	1.7824	-1.9349	-3.4681
10	C	3.1845	-2.0614	-3.4774
11	C	3.6093	-1.1868	-2.4796
12	C	-0.1376	2.7908	-1.6856
13	C	0.8898	3.7281	-1.4614
14	C	1.9902	2.9473	-1.1075
15	C	-0.1015	-1.3395	1.3025
16	C	0.8577	-1.2171	2.3266
17	C	1.9309	-0.5780	1.7080
18	H	3.4822	0.6623	-0.3326
19	N	-2.1165	-0.7417	-2.4494
20	S	-3.7060	-0.7609	-2.9443
21	O	-3.8878	-1.9189	-3.8328
22	O	-4.0598	0.5839	-3.4234
23	C	-4.4681	-1.0688	-1.3729
24	C	-4.6633	-2.3894	-0.9542
25	C	-4.7827	0.0178	-0.5449
26	C	-5.1967	-2.6173	0.3119
27	H	-4.4072	-3.2164	-1.6077
28	C	-5.3143	-0.2330	0.7133
29	H	-4.6228	1.0335	-0.8896
30	C	-5.5286	-1.5490	1.1628
31	H	-5.3625	-3.6380	0.6443
32	H	-5.5694	0.6004	1.3617
33	C	-6.1235	-1.7956	2.5236
34	H	-5.6645	-1.1479	3.2777
35	H	-7.1974	-1.5705	2.5167
36	H	-6.0018	-2.8362	2.8357
37	Br	-1.8325	-2.0832	1.3804
38	Br	-1.9302	3.0952	-2.1609
39	Br	0.5260	-2.8260	-4.5482
40	Br	4.2407	-3.1662	-4.5724
41	Br	5.3738	-0.8252	-1.9398

42	Br	3.5783	-0.0854	2.4745
43	Br	0.7224	-1.7835	4.1172
44	Br	0.7985	5.6022	-1.6124
45	Br	3.7239	3.5302	-0.6668
46	H	-1.0256	0.4518	-4.2500
47	Si	-0.5419	0.9375	-5.5728
48	C	-1.0923	2.7280	-5.7962
49	C	-1.2735	-0.1564	-6.9204
50	C	1.3448	0.8535	-5.5726
51	H	-2.1803	2.8187	-5.7027
52	H	-0.6354	3.3863	-5.0502
53	H	-0.8043	3.0968	-6.7881
54	H	-0.9428	-1.1956	-6.8240
55	H	-2.3679	-0.1508	-6.8565
56	H	-0.9957	0.1955	-7.9206
57	C	2.0877	1.6381	-4.6690
58	C	2.0595	0.0249	-6.4561
59	C	3.4825	1.5903	-4.6390
60	H	1.5731	2.3005	-3.9785
61	C	3.4565	-0.0209	-6.4388
62	H	1.5241	-0.5977	-7.1683
63	C	4.1710	0.7586	-5.5270
64	H	4.0326	2.2008	-3.9279
65	H	3.9856	-0.6690	-7.1316
66	H	5.2565	0.7178	-5.5061

step_004_s_SP
Energy (POTENTIAL) = -26981.8404514 Eh

	Atom	X	Y	Z
1	Cu	-0.3981	-0.3761	-1.8145
2	N	1.4618	-1.0757	-2.5283
3	N	2.5838	-0.6289	-1.8958
4	N	0.3892	1.5429	-1.4597
5	N	1.7009	1.6139	-1.0978
6	N	0.3928	-0.8570	0.1691
7	N	1.6461	-0.3897	0.4348
8	B	2.4635	0.3234	-0.6800
9	C	1.8514	-1.9395	-3.4621
10	C	3.2529	-2.0739	-3.4599
11	C	3.6733	-1.2170	-2.4453
12	C	-0.0394	2.7878	-1.6569
13	C	1.0050	3.7080	-1.4428
14	C	2.0924	2.9104	-1.0856
15	C	-0.0819	-1.3767	1.2951
16	C	0.8614	-1.2611	2.3348
17	C	1.9462	-0.6235	1.7356
18	H	3.5382	0.6068	-0.2781
19	N	-2.0578	-0.7011	-2.4682
20	S	-3.6476	-0.6929	-2.9663
21	O	-3.8398	-1.8335	-3.8751
22	O	-3.9929	0.6621	-3.4221
23	C	-4.4165	-1.0190	-1.4013
24	C	-4.6268	-2.3433	-1.0033
25	C	-4.7317	0.0587	-0.5617
26	C	-5.1766	-2.5844	0.2538
27	H	-4.3717	-3.1633	-1.6659
28	C	-5.2794	-0.2050	0.6867
29	H	-4.5619	1.0777	-0.8915
30	C	-5.5096	-1.5255	1.1153
31	H	-5.3561	-3.6082	0.5691
32	H	-5.5370	0.6215	1.3430
33	C	-6.1171	-1.7832	2.4684
34	H	-5.5752	-1.2413	3.2513
35	H	-7.1552	-1.4305	2.4957
36	H	-6.1143	-2.8475	2.7173
37	Br	-1.8159	-2.1159	1.3445
38	Br	-1.8232	3.1210	-2.1469
39	Br	0.6023	-2.8153	-4.5633
40	Br	4.3150	-3.1601	-4.5677
41	Br	5.4350	-0.8732	-1.8846

42	Br	3.5824	-0.1385	2.5308
43	Br	0.6957	-1.8335	4.1210
44	Br	0.9479	5.5817	-1.6118
45	Br	3.8400	3.4642	-0.6642
46	H	-1.1326	0.4271	-4.2721
47	Si	-0.6518	0.9077	-5.5993
48	C	-1.2548	2.6760	-5.8554
49	C	-1.3336	-0.2307	-6.9356
50	C	1.2356	0.8729	-5.5622
51	H	-2.3442	2.7357	-5.7536
52	H	-0.8114	3.3626	-5.1273
53	H	-0.9865	3.0312	-6.8577
54	H	-0.9699	-1.2568	-6.8191
55	H	-2.4281	-0.2596	-6.8826
56	H	-1.0568	0.1142	-7.9386
57	C	1.9359	1.6653	-4.6321
58	C	1.9921	0.0659	-6.4308
59	C	3.3297	1.6453	-4.5611
60	H	1.3884	2.3093	-3.9498
61	C	3.3886	0.0481	-6.3729
62	H	1.4901	-0.5631	-7.1614
63	C	4.0604	0.8343	-5.4343
64	H	3.8453	2.2602	-3.8284
65	H	3.9506	-0.5838	-7.0548
66	H	5.1454	0.8139	-5.3813

step_005_s_SP
Energy (POTENTIAL) = -26981.8360407 Eh

	Atom	X	Y	Z
1	Cu	-0.3925	-0.2761	-1.7747
2	N	1.4602	-1.0246	-2.4429
3	N	2.5846	-0.5624	-1.8278
4	N	0.4240	1.6606	-1.4816
5	N	1.7286	1.7127	-1.0882
6	N	0.3581	-0.6970	0.2223
7	N	1.6240	-0.2632	0.4852
8	B	2.4634	0.4189	-0.6343
9	C	1.8415	-1.9281	-3.3413
10	C	3.2421	-2.0709	-3.3365
11	C	3.6695	-1.1789	-2.3551
12	C	0.0254	2.9116	-1.7060
13	C	1.0822	3.8145	-1.4774
14	C	2.1446	3.0019	-1.0839
15	C	-0.1202	-1.2270	1.3422
16	C	0.8337	-1.1507	2.3759
17	C	1.9287	-0.5285	1.7789
18	H	3.5376	0.6922	-0.2246
19	N	-2.0318	-0.5549	-2.4891
20	S	-3.6166	-0.5687	-3.0034
21	O	-3.7650	-1.6675	-3.9703
22	O	-4.0031	0.7953	-3.3934
23	C	-4.3844	-0.9975	-1.4618
24	C	-4.5659	-2.3457	-1.1385
25	C	-4.7215	0.0254	-0.5640
26	C	-5.1104	-2.6680	0.1028
27	H	-4.2926	-3.1219	-1.8452
28	C	-5.2621	-0.3191	0.6677
29	H	-4.5688	1.0643	-0.8356
30	C	-5.4640	-1.6662	1.0221
31	H	-5.2671	-3.7112	0.3611
32	H	-5.5338	0.4641	1.3698
33	C	-6.0568	-2.0127	2.3622
34	H	-5.5142	-1.5122	3.1717
35	H	-7.0988	-1.6759	2.4204
36	H	-6.0379	-3.0898	2.5469
37	Br	-1.8670	-1.9367	1.3895
38	Br	-1.7378	3.2888	-2.2438
39	Br	0.5774	-2.8468	-4.3879
40	Br	4.2945	-3.2027	-4.4072
41	Br	5.4335	-0.8234	-1.8087

42	Br	3.5833	-0.1001	2.5685
43	Br	0.6677	-1.7491	4.1535
44	Br	1.0671	5.6874	-1.6663
45	Br	3.8906	3.5323	-0.6258
46	H	-1.1604	0.6275	-4.1539
47	Si	-0.6947	0.9649	-5.5312
48	C	-1.2013	2.7395	-5.9189
49	C	-1.4892	-0.2449	-6.7353
50	C	1.1870	0.8124	-5.5448
51	H	-2.2824	2.8728	-5.7998
52	H	-0.6992	3.4531	-5.2577
53	H	-0.9398	2.9974	-6.9522
54	H	-1.2386	-1.2811	-6.4866
55	H	-2.5803	-0.1504	-6.6996
56	H	-1.1725	-0.0551	-7.7673
57	C	1.9679	1.5636	-4.6448
58	C	1.8609	-0.0386	-6.4392
59	C	3.3602	1.4653	-4.6320
60	H	1.4871	2.2357	-3.9400
61	C	3.2552	-0.1377	-6.4365
62	H	1.2945	-0.6374	-7.1477
63	C	4.0077	0.6121	-5.5302
64	H	3.9401	2.0517	-3.9245
65	H	3.7522	-0.8033	-7.1367
66	H	5.0910	0.5299	-5.5201

step_006_s_SP
Energy (POTENTIAL) = -26981.8329407 Eh

	Atom	X	Y	Z
1	Cu	-0.3949	-0.2768	-1.7825
2	N	1.4531	-1.0598	-2.4448
3	N	2.5836	-0.6047	-1.8350
4	N	0.4432	1.6493	-1.4819
5	N	1.7563	1.6874	-1.1183
6	N	0.3622	-0.7026	0.2195
7	N	1.6302	-0.2698	0.4758
8	B	2.4759	0.3898	-0.6524
9	C	1.8240	-1.9811	-3.3292
10	C	3.2225	-2.1428	-3.3208
11	C	3.6609	-1.2426	-2.3522
12	C	0.0560	2.9033	-1.7079
13	C	1.1301	3.7939	-1.5133
14	C	2.1905	2.9702	-1.1370
15	C	-0.1183	-1.2103	1.3488
16	C	0.8350	-1.1195	2.3818
17	C	1.9330	-0.5130	1.7744
18	H	3.5540	0.6549	-0.2475
19	N	-2.0327	-0.4839	-2.5322
20	S	-3.6293	-0.4737	-3.0122
21	O	-3.8040	-1.5454	-4.0050
22	O	-4.0197	0.9015	-3.3567
23	C	-4.3706	-0.9415	-1.4678
24	C	-4.5460	-2.2976	-1.1768
25	C	-4.7009	0.0575	-0.5414
26	C	-5.0760	-2.6527	0.0618
27	H	-4.2796	-3.0549	-1.9062
28	C	-5.2274	-0.3194	0.6872
29	H	-4.5546	1.1034	-0.7883
30	C	-5.4214	-1.6756	1.0101
31	H	-5.2266	-3.7026	0.2958
32	H	-5.4931	0.4450	1.4119
33	C	-5.9948	-2.0576	2.3492
34	H	-5.4430	-1.5753	3.1636
35	H	-7.0370	-1.7266	2.4307
36	H	-5.9694	-3.1389	2.5070
37	Br	-1.8679	-1.9117	1.4086
38	Br	-1.7136	3.2958	-2.2117
39	Br	0.5515	-2.8973	-4.3677
40	Br	4.2607	-3.2994	-4.3793
41	Br	5.4295	-0.8980	-1.8137

42	Br	3.5892	-0.0793	2.5580
43	Br	0.6652	-1.6841	4.1701
44	Br	1.1361	5.6643	-1.7242
45	Br	3.9572	3.4764	-0.7353
46	H	-1.2279	0.5468	-4.2010
47	Si	-0.7363	0.9334	-5.5630
48	C	-1.2835	2.7020	-5.9170
49	C	-1.4694	-0.2751	-6.8058
50	C	1.1474	0.8272	-5.5268
51	H	-2.3673	2.8061	-5.7950
52	H	-0.7992	3.4150	-5.2423
53	H	-1.0278	2.9844	-6.9453
54	H	-1.1790	-1.3062	-6.5794
55	H	-2.5638	-0.2273	-6.7783
56	H	-1.1510	-0.0469	-7.8294
57	C	1.8884	1.6478	-4.6541
58	C	1.8618	-0.0670	-6.3448
59	C	3.2806	1.5711	-4.5888
60	H	1.3765	2.3591	-4.0124
61	C	3.2565	-0.1413	-6.2933
62	H	1.3274	-0.7189	-7.0309
63	C	3.9687	0.6737	-5.4106
64	H	3.8278	2.2096	-3.9004
65	H	3.7855	-0.8391	-6.9361
66	H	5.0521	0.6096	-5.3617

step_007_s_SP
Energy (POTENTIAL) = -26981.8427253 Eh

	Atom	X	Y	Z
1	Cu	-0.3830	-0.2600	-1.7874
2	N	1.4648	-1.0570	-2.4369
3	N	2.5952	-0.6061	-1.8239
4	N	0.4567	1.6518	-1.4605
5	N	1.7716	1.6878	-1.1057
6	N	0.3702	-0.7046	0.2243
7	N	1.6372	-0.2706	0.4849
8	B	2.4877	0.3875	-0.6404
9	C	1.8342	-1.9841	-3.3157
10	C	3.2319	-2.1535	-3.3010
11	C	3.6711	-1.2521	-2.3340
12	C	0.0676	2.9061	-1.6803
13	C	1.1432	3.7958	-1.4908
14	C	2.2061	2.9704	-1.1243
15	C	-0.1165	-1.2037	1.3544
16	C	0.8310	-1.1066	2.3923
17	C	1.9324	-0.5047	1.7871
18	H	3.5658	0.6498	-0.2331
19	N	-2.0140	-0.4268	-2.5734
20	S	-3.6169	-0.4143	-3.0333
21	O	-3.8013	-1.4749	-4.0368
22	O	-4.0173	0.9632	-3.3583
23	C	-4.3470	-0.9023	-1.4887
24	C	-4.5160	-2.2622	-1.2129
25	C	-4.6855	0.0844	-0.5526
26	C	-5.0465	-2.6338	0.0209
27	H	-4.2449	-3.0099	-1.9504
28	C	-5.2120	-0.3087	0.6710
29	H	-4.5472	1.1339	-0.7882
30	C	-5.3989	-1.6691	0.9790
31	H	-5.1916	-3.6871	0.2431
32	H	-5.4837	0.4464	1.4032
33	C	-5.9725	-2.0689	2.3129
34	H	-5.4316	-1.5845	3.1333
35	H	-7.0200	-1.7542	2.3923
36	H	-5.9323	-3.1508	2.4632
37	Br	-1.8682	-1.9001	1.4087
38	Br	-1.7062	3.2922	-2.1732
39	Br	0.5607	-2.8990	-4.3542
40	Br	4.2681	-3.3177	-4.3530
41	Br	5.4396	-0.9135	-1.7909

42	Br	3.5842	-0.0659	2.5776
43	Br	0.6506	-1.6596	4.1834
44	Br	1.1477	5.6671	-1.6943
45	Br	3.9763	3.4734	-0.7342
46	H	-1.2594	0.4773	-4.2149
47	Si	-0.7642	0.8962	-5.5732
48	C	-1.3331	2.6641	-5.8903
49	C	-1.4792	-0.3032	-6.8342
50	C	1.1193	0.8066	-5.5231
51	H	-2.4157	2.7546	-5.7494
52	H	-0.8451	3.3704	-5.2114
53	H	-1.0951	2.9648	-6.9177
54	H	-1.1795	-1.3340	-6.6184
55	H	-2.5739	-0.2664	-6.8119
56	H	-1.1566	-0.0593	-7.8530
57	C	1.8482	1.6317	-4.6445
58	C	1.8453	-0.0843	-6.3348
59	C	3.2401	1.5620	-4.5669
60	H	1.3274	2.3403	-4.0072
61	C	3.2398	-0.1511	-6.2711
62	H	1.3204	-0.7393	-7.0252
63	C	3.9399	0.6678	-5.3822
64	H	3.7777	2.2033	-3.8737
65	H	3.7780	-0.8462	-6.9092
66	H	5.0231	0.6090	-5.3236

step_008_s_SP
Energy (POTENTIAL) = -26981.8446451 Eh

	Atom	X	Y	Z
1	Cu	-0.3626	-0.4679	-1.9823
2	N	1.4875	-1.3257	-2.6052
3	N	2.6232	-0.8676	-2.0088
4	N	0.5126	1.3805	-1.5822
5	N	1.8046	1.3900	-1.1481
6	N	0.3727	-1.0106	0.0466
7	N	1.6672	-0.6666	0.3087
8	B	2.5172	0.0629	-0.7722
9	C	1.8482	-2.2679	-3.4696
10	C	3.2459	-2.4382	-3.4672
11	C	3.6945	-1.5238	-2.5163
12	C	0.1139	2.6468	-1.6751
13	C	1.1545	3.5201	-1.3098
14	C	2.2107	2.6716	-0.9824
15	C	-0.0946	-1.6278	1.1254
16	C	0.8938	-1.7065	2.1264
17	C	1.9996	-1.0797	1.5562
18	H	3.5961	0.2955	-0.3506
19	N	-1.9783	-0.6523	-2.8165
20	S	-3.6109	-0.4868	-3.1217
21	O	-4.0284	-1.6056	-3.9819
22	O	-3.8768	0.8933	-3.5591
23	C	-4.2538	-0.7184	-1.4803
24	C	-4.7375	-1.9728	-1.1017
25	C	-4.2532	0.3656	-0.5920
26	C	-5.2452	-2.1337	0.1868
27	H	-4.7299	-2.7975	-1.8060
28	C	-4.7602	0.1824	0.6879
29	H	-3.8751	1.3320	-0.9059
30	C	-5.2643	-1.0651	1.0979
31	H	-5.6399	-3.1002	0.4869
32	H	-4.7706	1.0173	1.3829
33	C	-5.7898	-1.2452	2.4977
34	H	-4.9670	-1.2244	3.2233
35	H	-6.4743	-0.4344	2.7685
36	H	-6.3157	-2.1966	2.6127
37	Br	-1.8764	-2.2395	1.1738
38	Br	-1.6244	3.0695	-2.2531
39	Br	0.5639	-3.1763	-4.5024
40	Br	4.2732	-3.5982	-4.5352
41	Br	5.4673	-1.1820	-1.9897

42	Br	3.6982	-0.8152	2.3255
43	Br	0.7529	-2.4806	3.8379
44	Br	1.1380	5.4013	-1.3095
45	Br	3.9410	3.1539	-0.4268
46	H	-1.3213	-0.2583	-4.5772
47	Si	-0.7894	0.4638	-5.8050
48	C	-1.9240	1.9263	-6.1349
49	C	-0.7833	-0.7460	-7.2481
50	C	0.9563	1.0339	-5.3763
51	H	-2.9431	1.5767	-6.3293
52	H	-1.9685	2.6021	-5.2764
53	H	-1.5832	2.4973	-7.0067
54	H	-0.1094	-1.5906	-7.0764
55	H	-1.7895	-1.1493	-7.4094
56	H	-0.4682	-0.2441	-8.1707
57	C	1.1876	2.2966	-4.7979
58	C	2.0717	0.2011	-5.5888
59	C	2.4712	2.7019	-4.4252
60	H	0.3569	2.9778	-4.6364
61	C	3.3572	0.5986	-5.2161
62	H	1.9400	-0.7733	-6.0515
63	C	3.5590	1.8482	-4.6243
64	H	2.6226	3.6824	-3.9831
65	H	4.2006	-0.0633	-5.3912
66	H	4.5580	2.1585	-4.3306

step_009_s_SP
Energy (POTENTIAL) = -26981.8466675 Eh

	Atom	X	Y	Z
1	Cu	-0.3625	-0.4457	-1.9598
2	N	1.4910	-1.3140	-2.6040
3	N	2.6317	-0.8563	-2.0174
4	N	0.5239	1.3953	-1.5633
5	N	1.8202	1.3998	-1.1423
6	N	0.3915	-1.0016	0.0534
7	N	1.6897	-0.6634	0.3058
8	B	2.5337	0.0699	-0.7770
9	C	1.8444	-2.2573	-3.4698
10	C	3.2420	-2.4291	-3.4785
11	C	3.6988	-1.5139	-2.5324
12	C	0.1298	2.6631	-1.6548
13	C	1.1776	3.5325	-1.3012
14	C	2.2334	2.6800	-0.9829
15	C	-0.0670	-1.6260	1.1319
16	C	0.9306	-1.7156	2.1226
17	C	2.0327	-1.0878	1.5467
18	H	3.6154	0.3000	-0.3607
19	N	-1.9540	-0.6173	-2.8561
20	S	-3.5935	-0.4534	-3.1219
21	O	-4.0243	-1.5596	-3.9927
22	O	-3.8744	0.9322	-3.5338
23	C	-4.2202	-0.7102	-1.4768
24	C	-4.6949	-1.9719	-1.1114
25	C	-4.2238	0.3626	-0.5760
26	C	-5.1962	-2.1516	0.1769
27	H	-4.6822	-2.7888	-1.8246
28	C	-4.7249	0.1611	0.7040
29	H	-3.8501	1.3343	-0.8788
30	C	-5.2208	-1.0937	1.1004
31	H	-5.5794	-3.1254	0.4685
32	H	-4.7339	0.9866	1.4101
33	C	-5.7802	-1.2863	2.4854
34	H	-5.1693	-0.7724	3.2344
35	H	-6.7923	-0.8674	2.5516
36	H	-5.8404	-2.3451	2.7519
37	Br	-1.8503	-2.2303	1.1924
38	Br	-1.6120	3.0907	-2.2190
39	Br	0.5502	-3.1663	-4.4907
40	Br	4.2599	-3.5916	-4.5533
41	Br	5.4760	-1.1714	-2.0209

42	Br	3.7399	-0.8364	2.3015
43	Br	0.8028	-2.5012	3.8298
44	Br	1.1686	5.4138	-1.3030
45	Br	3.9712	3.1562	-0.4452
46	H	-1.3541	-0.2460	-4.5339
47	Si	-0.8312	0.4670	-5.7887
48	C	-1.9685	1.9292	-6.1057
49	C	-0.8528	-0.7564	-7.2188
50	C	0.9187	1.0294	-5.3758
51	H	-2.9905	1.5808	-6.2850
52	H	-1.9996	2.6078	-5.2489
53	H	-1.6372	2.4958	-6.9842
54	H	-0.1760	-1.5995	-7.0530
55	H	-1.8622	-1.1594	-7.3591
56	H	-0.5539	-0.2604	-8.1501
57	C	1.1567	2.2894	-4.7938
58	C	2.0307	0.1960	-5.6043
59	C	2.4447	2.6924	-4.4349
60	H	0.3282	2.9699	-4.6196
61	C	3.3205	0.5916	-5.2450
62	H	1.8930	-0.7766	-6.0688
63	C	3.5296	1.8388	-4.6505
64	H	2.6021	3.6709	-3.9907
65	H	4.1616	-0.0699	-5.4316
66	H	4.5321	2.1473	-4.3675

step_010_s_SP
Energy (POTENTIAL) = -26981.8500531 Eh

	Atom	X	Y	Z
1	Cu	-0.3867	-0.4037	-1.9143
2	N	1.4843	-1.2766	-2.5914
3	N	2.6254	-0.8184	-2.0061
4	N	0.5058	1.4359	-1.5204
5	N	1.8067	1.4310	-1.1116
6	N	0.3825	-0.9811	0.0657
7	N	1.6817	-0.6458	0.3175
8	B	2.5235	0.0982	-0.7600
9	C	1.8369	-2.2253	-3.4502
10	C	3.2346	-2.4010	-3.4566
11	C	3.6923	-1.4819	-2.5153
12	C	0.1214	2.7074	-1.6098
13	C	1.1793	3.5687	-1.2666
14	C	2.2311	2.7081	-0.9570
15	C	-0.0730	-1.6165	1.1395
16	C	0.9278	-1.7168	2.1255
17	C	2.0284	-1.0836	1.5525
18	H	3.6040	0.3282	-0.3407
19	N	-1.9204	-0.5995	-2.9180
20	S	-3.5632	-0.4217	-3.1477
21	O	-4.0174	-1.5088	-4.0317
22	O	-3.8424	0.9731	-3.5322
23	C	-4.1852	-0.6982	-1.5021
24	C	-4.6673	-1.9611	-1.1527
25	C	-4.1936	0.3654	-0.5914
26	C	-5.1809	-2.1514	0.1295
27	H	-4.6529	-2.7710	-1.8739
28	C	-4.7079	0.1542	0.6823
29	H	-3.8153	1.3387	-0.8829
30	C	-5.2128	-1.1017	1.0620
31	H	-5.5701	-3.1266	0.4082
32	H	-4.7213	0.9737	1.3953
33	C	-5.7938	-1.3046	2.4370
34	H	-5.2068	-0.7801	3.1976
35	H	-6.8147	-0.9050	2.4840
36	H	-5.8398	-2.3645	2.7020
37	Br	-1.8565	-2.2176	1.2003
38	Br	-1.6210	3.1551	-2.1586
39	Br	0.5406	-3.1395	-4.4648
40	Br	4.2511	-3.5685	-4.5274
41	Br	5.4698	-1.1383	-2.0046

42	Br	3.7385	-0.8426	2.3036
43	Br	0.8045	-2.5187	3.8253
44	Br	1.1840	5.4502	-1.2644
45	Br	3.9763	3.1735	-0.4332
46	H	-1.3546	-0.2814	-4.5109
47	Si	-0.8334	0.4365	-5.7882
48	C	-1.9687	1.9061	-6.0707
49	C	-0.8832	-0.7837	-7.2186
50	C	0.9182	0.9843	-5.3764
51	H	-2.9891	1.5649	-6.2700
52	H	-2.0074	2.5562	-5.1927
53	H	-1.6274	2.4984	-6.9281
54	H	-0.2047	-1.6282	-7.0682
55	H	-1.8959	-1.1832	-7.3428
56	H	-0.5998	-0.2832	-8.1525
57	C	1.1557	2.2263	-4.7559
58	C	2.0307	0.1632	-5.6439
59	C	2.4459	2.6246	-4.4012
60	H	0.3254	2.8957	-4.5509
61	C	3.3226	0.5550	-5.2882
62	H	1.8921	-0.7963	-6.1344
63	C	3.5322	1.7845	-4.6582
64	H	2.6042	3.5894	-3.9288
65	H	4.1648	-0.0962	-5.5038
66	H	4.5366	2.0894	-4.3780

step_011_s_SP
Energy (POTENTIAL) = -26981.8537413 Eh

	Atom	X	Y	Z
1	Cu	-0.4715	-0.3841	-1.8726
2	N	1.4379	-1.2431	-2.5649
3	N	2.5659	-0.7808	-1.9578
4	N	0.4104	1.4640	-1.4802
5	N	1.7131	1.4607	-1.0746
6	N	0.3126	-1.0132	0.0713
7	N	1.5907	-0.6217	0.3520
8	B	2.4352	0.1352	-0.7137
9	C	1.8101	-2.1963	-3.4087
10	C	3.2081	-2.3726	-3.3835
11	C	3.6447	-1.4477	-2.4384
12	C	0.0415	2.7371	-1.6145
13	C	1.1090	3.5979	-1.3025
14	C	2.1519	2.7372	-0.9655
15	C	-0.1162	-1.7208	1.1117
16	C	0.8775	-1.8041	2.1063
17	C	1.9474	-1.0894	1.5730
18	H	3.5071	0.3763	-0.2791
19	N	-1.9075	-0.5896	-3.0233
20	S	-3.5549	-0.4046	-3.1986
21	O	-4.0446	-1.4837	-4.0750
22	O	-3.8440	0.9930	-3.5698
23	C	-4.1379	-0.6859	-1.5369
24	C	-4.7372	-1.9037	-1.2154
25	C	-4.0098	0.3370	-0.5886
26	C	-5.2315	-2.0888	0.0766
27	H	-4.8301	-2.6812	-1.9657
28	C	-4.5015	0.1299	0.6940
29	H	-3.5463	1.2793	-0.8577
30	C	-5.1235	-1.0811	1.0464
31	H	-5.7165	-3.0271	0.3307
32	H	-4.4102	0.9192	1.4351
33	C	-5.6457	-1.2825	2.4452
34	H	-4.8243	-1.2703	3.1719
35	H	-6.3321	-0.4763	2.7271
36	H	-6.1740	-2.2342	2.5466
37	Br	-1.8473	-2.4615	1.1228
38	Br	-1.6907	3.1940	-2.1858
39	Br	0.5354	-3.1156	-4.4471
40	Br	4.2479	-3.5452	-4.4264
41	Br	5.4109	-1.0971	-1.8930

42	Br	3.6321	-0.7843	2.3572
43	Br	0.7789	-2.6725	3.7748
44	Br	1.1382	5.4779	-1.3709
45	Br	3.9039	3.2031	-0.4664
46	H	-1.3588	-0.2970	-4.5194
47	Si	-0.8121	0.4211	-5.8226
48	C	-1.9557	1.8825	-6.1069
49	C	-0.8489	-0.8119	-7.2405
50	C	0.9283	0.9677	-5.3774
51	H	-2.9695	1.5353	-6.3278
52	H	-2.0142	2.5216	-5.2221
53	H	-1.6036	2.4864	-6.9521
54	H	-0.1617	-1.6478	-7.0837
55	H	-1.8576	-1.2216	-7.3629
56	H	-0.5698	-0.3141	-8.1774
57	C	1.1516	2.2047	-4.7409
58	C	2.0465	0.1504	-5.6334
59	C	2.4352	2.6023	-4.3629
60	H	0.3164	2.8706	-4.5435
61	C	3.3311	0.5416	-5.2529
62	H	1.9173	-0.8059	-6.1326
63	C	3.5274	1.7664	-4.6096
64	H	2.5841	3.5629	-3.8794
65	H	4.1781	-0.1066	-5.4585
66	H	4.5261	2.0707	-4.3093

step_012_s_SP
Energy (POTENTIAL) = -26981.8587996 Eh

	Atom	X	Y	Z
1	Cu	-0.5067	-0.3800	-1.8045
2	N	1.4081	-1.2488	-2.5715
3	N	2.5467	-0.7846	-1.9852
4	N	0.4034	1.4740	-1.4694
5	N	1.7153	1.4638	-1.0944
6	N	0.3279	-0.9948	0.0974
7	N	1.6163	-0.6129	0.3425
8	B	2.4401	0.1370	-0.7441
9	C	1.7683	-2.1999	-3.4224
10	C	3.1670	-2.3739	-3.4227
11	C	3.6186	-1.4491	-2.4850
12	C	0.0443	2.7486	-1.6155
13	C	1.1265	3.6033	-1.3392
14	C	2.1684	2.7375	-1.0131
15	C	-0.0791	-1.6947	1.1528
16	C	0.9407	-1.7833	2.1195
17	C	2.0016	-1.0795	1.5548
18	H	3.5202	0.3777	-0.3303
19	N	-1.8887	-0.5713	-3.0378
20	S	-3.5373	-0.3760	-3.1742
21	O	-4.0429	-1.4425	-4.0590
22	O	-3.8378	1.0259	-3.5230
23	C	-4.1134	-0.6771	-1.5113
24	C	-4.6839	-1.9095	-1.1947
25	C	-4.0174	0.3468	-0.5608
26	C	-5.1789	-2.1105	0.0950
27	H	-4.7557	-2.6871	-1.9473
28	C	-4.5101	0.1246	0.7194
29	H	-3.5796	1.3021	-0.8271
30	C	-5.1019	-1.1025	1.0671
31	H	-5.6403	-3.0618	0.3450
32	H	-4.4437	0.9152	1.4620
33	C	-5.6256	-1.3208	2.4632
34	H	-4.8069	-1.3042	3.1930
35	H	-6.3231	-0.5258	2.7495
36	H	-6.1420	-2.2800	2.5558
37	Br	-1.8168	-2.4149	1.2201
38	Br	-1.6934	3.2131	-2.1630
39	Br	0.4798	-3.1236	-4.4411
40	Br	4.1907	-3.5429	-4.4857
41	Br	5.3939	-1.0990	-1.9682

42	Br	3.7083	-0.7858	2.2938
43	Br	0.8814	-2.6474	3.7918
44	Br	1.1730	5.4819	-1.4363
45	Br	3.9355	3.1942	-0.5611
46	H	-1.3702	-0.2845	-4.4377
47	Si	-0.8328	0.4227	-5.8018
48	C	-1.9873	1.8785	-6.0671
49	C	-0.9194	-0.8330	-7.1963
50	C	0.9130	0.9653	-5.3866
51	H	-3.0057	1.5263	-6.2559
52	H	-2.0228	2.5282	-5.1890
53	H	-1.6589	2.4719	-6.9293
54	H	-0.1646	-1.6183	-7.1030
55	H	-1.9055	-1.3095	-7.2191
56	H	-0.7676	-0.3280	-8.1587
57	C	1.1453	2.2033	-4.7546
58	C	2.0264	0.1453	-5.6559
59	C	2.4345	2.6010	-4.3976
60	H	0.3131	2.8692	-4.5446
61	C	3.3166	0.5381	-5.2973
62	H	1.8893	-0.8129	-6.1490
63	C	3.5228	1.7650	-4.6611
64	H	2.5912	3.5620	-3.9172
65	H	4.1602	-0.1109	-5.5142
66	H	4.5261	2.0701	-4.3776

step_013_s_SP
Energy (POTENTIAL) = -26981.864252 Eh

	Atom	X	Y	Z
1	Cu	-0.5690	-0.3154	-1.6409
2	N	1.4190	-1.0898	-2.4475
3	N	2.5251	-0.6011	-1.8205
4	N	0.2680	1.5781	-1.2947
5	N	1.5920	1.6091	-0.9671
6	N	0.2758	-0.8970	0.2334
7	N	1.5428	-0.4575	0.4944
8	B	2.3582	0.3103	-0.5834
9	C	1.8435	-1.9465	-3.3675
10	C	3.2510	-2.0319	-3.3686
11	C	3.6395	-1.1530	-2.3618
12	C	-0.1119	2.8350	-1.5270
13	C	0.9729	3.7172	-1.3681
14	C	2.0351	2.8880	-1.0106
15	C	-0.1336	-1.5714	1.3049
16	C	0.8646	-1.5876	2.2970
17	C	1.9134	-0.8656	1.7323
18	H	3.4190	0.5980	-0.1496
19	N	-1.8490	-0.4901	-2.9883
20	S	-3.5011	-0.4129	-3.1666
21	O	-3.8957	-1.5081	-4.0745
22	O	-3.9018	0.9635	-3.5214
23	C	-4.1193	-0.7708	-1.5288
24	C	-4.5723	-2.0556	-1.2314
25	C	-4.1774	0.2582	-0.5815
26	C	-5.1008	-2.3071	0.0361
27	H	-4.5263	-2.8380	-1.9811
28	C	-4.7028	-0.0129	0.6763
29	H	-3.8300	1.2535	-0.8338
30	C	-5.1772	-1.2953	1.0043
31	H	-5.4662	-3.3028	0.2715
32	H	-4.7523	0.7808	1.4170
33	C	-5.7504	-1.5637	2.3722
34	H	-5.0488	-1.2611	3.1578
35	H	-6.6715	-0.9896	2.5282
36	H	-5.9845	-2.6226	2.5110
37	Br	-1.8537	-2.3330	1.3625
38	Br	-1.8780	3.2459	-2.0255
39	Br	0.6257	-2.8927	-4.4507
40	Br	4.3521	-3.0796	-4.4778
41	Br	5.3873	-0.7356	-1.8021

42	Br	3.5884	-0.4837	2.5026
43	Br	0.7947	-2.3958	3.9964
44	Br	0.9892	5.5853	-1.5949
45	Br	3.8130	3.3915	-0.6576
46	H	-1.3252	-0.1211	-4.2557
47	Si	-0.8000	0.5759	-5.7032
48	C	-1.6952	2.2259	-5.7629
49	C	-1.3061	-0.6036	-7.0731
50	C	1.0530	0.7713	-5.5255
51	H	-2.7556	2.0960	-5.5292
52	H	-1.2745	2.9323	-5.0417
53	H	-1.6010	2.6655	-6.7639
54	H	-0.8189	-1.5775	-6.9679
55	H	-2.3894	-0.7638	-7.0492
56	H	-1.0460	-0.1941	-8.0571
57	C	1.5884	1.6674	-4.5784
58	C	1.9526	0.0253	-6.3116
59	C	2.9665	1.8114	-4.4201
60	H	0.9260	2.2582	-3.9531
61	C	3.3327	0.1770	-6.1651
62	H	1.5760	-0.6785	-7.0487
63	C	3.8421	1.0664	-5.2155
64	H	3.3582	2.5034	-3.6805
65	H	4.0097	-0.4021	-6.7861
66	H	4.9158	1.1777	-5.0943

step_014_s_SP
Energy (POTENTIAL) = -26981.8723553 Eh

	Atom	X	Y	Z
1	Cu	-0.5507	-0.3911	-1.7659
2	N	1.3767	-1.2980	-2.5789
3	N	2.5184	-0.8274	-2.0031
4	N	0.4016	1.4484	-1.4781
5	N	1.7092	1.4228	-1.0891
6	N	0.2973	-1.0169	0.0970
7	N	1.5966	-0.6641	0.3294
8	B	2.4224	0.0866	-0.7556
9	C	1.7364	-2.2419	-3.4379
10	C	3.1371	-2.4035	-3.4553
11	C	3.5910	-1.4794	-2.5184
12	C	0.0559	2.7276	-1.6191
13	C	1.1419	3.5694	-1.3200
14	C	2.1720	2.6917	-0.9895
15	C	-0.1086	-1.7278	1.1469
16	C	0.9231	-1.8512	2.0962
17	C	1.9895	-1.1589	1.5275
18	H	3.5056	0.3145	-0.3431
19	N	-1.8759	-0.5543	-3.0783
20	S	-3.5227	-0.3293	-3.1568
21	O	-4.0652	-1.3800	-4.0412
22	O	-3.8214	1.0782	-3.4889
23	C	-4.0875	-0.6349	-1.4887
24	C	-4.7000	-1.8498	-1.1861
25	C	-3.9609	0.3744	-0.5267
26	C	-5.2006	-2.0507	0.1020
27	H	-4.7983	-2.6153	-1.9480
28	C	-4.4598	0.1532	0.7517
29	H	-3.4946	1.3193	-0.7824
30	C	-5.0923	-1.0574	1.0854
31	H	-5.6923	-2.9898	0.3404
32	H	-4.3677	0.9328	1.5033
33	C	-5.6253	-1.2742	2.4786
34	H	-4.8118	-1.2595	3.2141
35	H	-6.3236	-0.4781	2.7603
36	H	-6.1449	-2.2320	2.5675
37	Br	-1.8575	-2.4157	1.2327
38	Br	-1.6663	3.2112	-2.1937
39	Br	0.4472	-3.1802	-4.4457
40	Br	4.1605	-3.5615	-4.5311
41	Br	5.3695	-1.1157	-2.0221

42	Br	3.7125	-0.9142	2.2455
43	Br	0.8708	-2.7419	3.7542
44	Br	1.2068	5.4479	-1.4002
45	Br	3.9352	3.1305	-0.5052
46	H	-1.4251	-0.3374	-4.3000
47	Si	-0.7948	0.3921	-5.8542
48	C	-2.0079	1.8011	-6.1289
49	C	-0.8021	-0.8798	-7.2381
50	C	0.9156	0.9920	-5.3892
51	H	-2.9888	1.4081	-6.4132
52	H	-2.1433	2.3930	-5.2204
53	H	-1.6540	2.4608	-6.9313
54	H	-0.0017	-1.6167	-7.1312
55	H	-1.7569	-1.4155	-7.2669
56	H	-0.6722	-0.3734	-8.2036
57	C	1.0946	2.2572	-4.7925
58	C	2.0582	0.1893	-5.5833
59	C	2.3597	2.6999	-4.4047
60	H	0.2391	2.9083	-4.6364
61	C	3.3232	0.6266	-5.1903
62	H	1.9625	-0.7888	-6.0463
63	C	3.4766	1.8818	-4.5950
64	H	2.4749	3.6824	-3.9571
65	H	4.1895	-0.0085	-5.3510
66	H	4.4611	2.2222	-4.2873

step_015_s_SP
Energy (POTENTIAL) = -26981.878527 Eh

	Atom	X	Y	Z
1	Cu	-0.6862	-0.3576	-1.5029
2	N	1.2411	-1.1915	-2.4528
3	N	2.3898	-0.6950	-1.9140
4	N	0.2151	1.5349	-1.3517
5	N	1.5477	1.5565	-1.0597
6	N	0.2438	-0.8231	0.3323
7	N	1.5566	-0.4604	0.4579
8	B	2.3152	0.2517	-0.6996
9	C	1.6007	-2.0596	-3.3903
10	C	3.0048	-2.1491	-3.4873
11	C	3.4639	-1.2566	-2.5231
12	C	-0.1703	2.7966	-1.5456
13	C	0.9202	3.6738	-1.3956
14	C	1.9899	2.8366	-1.0822
15	C	-0.1246	-1.3621	1.4953
16	C	0.9495	-1.3720	2.4032
17	C	1.9967	-0.7866	1.6961
18	H	3.4050	0.5316	-0.3406
19	N	-1.8865	-0.5517	-2.9380
20	S	-3.5314	-0.3956	-3.1250
21	O	-3.9541	-1.4540	-4.0653
22	O	-3.9040	0.9965	-3.4486
23	C	-4.1669	-0.7811	-1.4990
24	C	-4.5219	-2.0965	-1.1976
25	C	-4.3283	0.2467	-0.5646
26	C	-5.0510	-2.3802	0.0612
27	H	-4.3983	-2.8793	-1.9383
28	C	-4.8557	-0.0565	0.6871
29	H	-4.0581	1.2647	-0.8211
30	C	-5.2302	-1.3695	1.0180
31	H	-5.3350	-3.4011	0.3011
32	H	-4.9838	0.7366	1.4187
33	C	-5.8256	-1.6769	2.3691
34	H	-5.3182	-1.1213	3.1645
35	H	-6.8837	-1.3884	2.3997
36	H	-5.7685	-2.7445	2.6002
37	Br	-1.8857	-1.9565	1.7915
38	Br	-1.9520	3.2203	-1.9654
39	Br	0.3136	-3.0000	-4.3982
40	Br	4.0260	-3.2215	-4.6485
41	Br	5.2464	-0.8397	-2.0887

42	Br	3.7532	-0.4802	2.2987
43	Br	0.9645	-2.0176	4.1714
44	Br	0.9319	5.5462	-1.5848
45	Br	3.7719	3.3346	-0.7420
46	H	-1.3927	-0.2786	-4.0198
47	Si	-0.6591	0.5929	-5.7117
48	C	-1.5703	2.2415	-5.6701
49	C	-1.1462	-0.4951	-7.1700
50	C	1.1931	0.7958	-5.5210
51	H	-2.6277	2.0949	-5.4317
52	H	-1.1473	2.9118	-4.9167
53	H	-1.4922	2.7368	-6.6470
54	H	-0.6343	-1.4617	-7.1404
55	H	-2.2250	-0.6847	-7.1562
56	H	-0.9006	-0.0062	-8.1218
57	C	1.7216	1.7289	-4.6033
58	C	2.1047	0.0210	-6.2676
59	C	3.0975	1.8885	-4.4465
60	H	1.0531	2.3398	-4.0039
61	C	3.4830	0.1847	-6.1173
62	H	1.7384	-0.7118	-6.9815
63	C	3.9832	1.1173	-5.2055
64	H	3.4809	2.6144	-3.7359
65	H	4.1663	-0.4177	-6.7084
66	H	5.0553	1.2428	-5.0856

step_016_s_SP
Energy (POTENTIAL) = -26981.8852933 Eh

	Atom	X	Y	Z
1	Cu	-0.7103	-0.3708	-1.5307
2	N	1.2473	-1.1661	-2.4608
3	N	2.3761	-0.6729	-1.8773
4	N	0.1589	1.5154	-1.3391
5	N	1.4834	1.5509	-1.0134
6	N	0.1922	-0.9014	0.3003
7	N	1.4861	-0.4930	0.4748
8	B	2.2588	0.2550	-0.6490
9	C	1.6410	-2.0198	-3.3978
10	C	3.0478	-2.1032	-3.4497
11	C	3.4714	-1.2228	-2.4587
12	C	-0.2265	2.7710	-1.5732
13	C	0.8554	3.6563	-1.4134
14	C	1.9202	2.8319	-1.0533
15	C	-0.1579	-1.5687	1.4016
16	C	0.9068	-1.6080	2.3199
17	C	1.9305	-0.9112	1.6834
18	H	3.3354	0.5447	-0.2595
19	N	-1.9317	-0.5956	-2.9447
20	S	-3.5836	-0.4569	-3.1040
21	O	-4.0209	-1.5513	-3.9934
22	O	-3.9635	0.9214	-3.4725
23	C	-4.1762	-0.7846	-1.4500
24	C	-4.6231	-2.0651	-1.1273
25	C	-4.2254	0.2578	-0.5183
26	C	-5.1279	-2.3008	0.1526
27	H	-4.5879	-2.8580	-1.8666
28	C	-4.7264	0.0022	0.7538
29	H	-3.8898	1.2517	-0.7908
30	C	-5.1904	-1.2764	1.1080
31	H	-5.4849	-3.2946	0.4086
32	H	-4.7660	0.8066	1.4835
33	C	-5.7425	-1.5280	2.4881
34	H	-5.0527	-1.1697	3.2602
35	H	-6.6887	-0.9927	2.6320
36	H	-5.9289	-2.5917	2.6601
37	Br	-1.8766	-2.3077	1.6106
38	Br	-1.9923	3.1778	-2.0650
39	Br	0.3921	-2.9536	-4.4582
40	Br	4.1099	-3.1562	-4.5911
41	Br	5.2370	-0.8063	-1.9603

42	Br	3.6628	-0.5754	2.3373
43	Br	0.9401	-2.4144	4.0202
44	Br	0.8645	5.5231	-1.6476
45	Br	3.6901	3.3464	-0.6790
46	H	-1.4990	-0.4294	-3.9645
47	Si	-0.6309	0.5795	-5.8380
48	C	-1.5942	2.2018	-5.7740
49	C	-1.0055	-0.4383	-7.3825
50	C	1.2065	0.8270	-5.5654
51	H	-2.6491	2.0221	-5.5460
52	H	-1.1973	2.8707	-5.0056
53	H	-1.5267	2.7176	-6.7412
54	H	-0.5157	-1.4165	-7.3508
55	H	-2.0842	-0.6067	-7.4735
56	H	-0.6679	0.0824	-8.2883
57	C	1.6805	1.7538	-4.6118
58	C	2.1636	0.0726	-6.2760
59	C	3.0456	1.9280	-4.3888
60	H	0.9779	2.3487	-4.0356
61	C	3.5310	0.2494	-6.0582
62	H	1.8404	-0.6557	-7.0150
63	C	3.9766	1.1769	-5.1132
64	H	3.3859	2.6509	-3.6538
65	H	4.2484	-0.3384	-6.6231
66	H	5.0402	1.3136	-4.9409

step_017_s_SP
Energy (POTENTIAL) = -26981.8869224 Eh

	Atom	X	Y	Z
1	Cu	-0.6312	-0.4042	-1.8023
2	N	1.4338	-1.1039	-2.5449
3	N	2.4784	-0.6356	-1.8028
4	N	0.1819	1.4585	-1.3799
5	N	1.4408	1.4865	-0.8550
6	N	0.1136	-1.1811	-0.0064
7	N	1.3171	-0.6983	0.4250
8	B	2.1977	0.1767	-0.5152
9	C	1.9406	-1.9456	-3.4354
10	C	3.3419	-2.0409	-3.3042
11	C	3.6366	-1.1875	-2.2462
12	C	-0.1934	2.7249	-1.5747
13	C	0.8249	3.6083	-1.1748
14	C	1.8459	2.7716	-0.7269
15	C	-0.2996	-2.0739	0.8921
16	C	0.6253	-2.1776	1.9483
17	C	1.6384	-1.2869	1.6019
18	H	3.2141	0.4403	0.0258
19	N	-1.9469	-0.6738	-3.1126
20	S	-3.6089	-0.4969	-3.1074
21	O	-4.1854	-1.6264	-3.8607
22	O	-3.9550	0.8700	-3.5442
23	C	-4.0300	-0.6815	-1.3800
24	C	-4.9135	-1.6944	-1.0075
25	C	-3.5296	0.2215	-0.4377
26	C	-5.2962	-1.7983	0.3304
27	H	-5.2966	-2.3832	-1.7522
28	C	-3.9118	0.0941	0.8944
29	H	-2.8550	1.0150	-0.7358
30	C	-4.8028	-0.9124	1.2994
31	H	-5.9910	-2.5802	0.6244
32	H	-3.5199	0.7919	1.6292
33	C	-5.1880	-1.0605	2.7487
34	H	-4.5100	-1.7594	3.2553
35	H	-5.1279	-0.1053	3.2786
36	H	-6.2027	-1.4554	2.8558
37	Br	-1.8981	-3.0378	0.6622
38	Br	-1.8609	3.1531	-2.3276
39	Br	0.8287	-2.8677	-4.6467
40	Br	4.5414	-3.0463	-4.3496
41	Br	5.3265	-0.7861	-1.5210

42	Br	3.2126	-0.9107	2.5610
43	Br	0.5182	-3.2696	3.4777
44	Br	0.8185	5.4883	-1.2459
45	Br	3.5353	3.2786	-0.0759
46	H	-1.6399	-0.6754	-4.1483
47	Si	-0.7629	0.3567	-6.2362
48	C	-1.9697	1.8091	-6.2830
49	C	-0.6950	-0.6037	-7.8611
50	C	0.9401	0.8701	-5.6453
51	H	-2.9557	1.4766	-6.6235
52	H	-2.0987	2.2564	-5.2937
53	H	-1.6132	2.5867	-6.9717
54	H	-0.0934	-1.5140	-7.7728
55	H	-1.7023	-0.8984	-8.1747
56	H	-0.2589	0.0117	-8.6592
57	C	1.1002	1.9217	-4.7184
58	C	2.1013	0.1829	-6.0588
59	C	2.3581	2.2676	-4.2243
60	H	0.2323	2.4827	-4.3851
61	C	3.3604	0.5262	-5.5656
62	H	2.0235	-0.6338	-6.7712
63	C	3.4931	1.5664	-4.6411
64	H	2.4534	3.0864	-3.5179
65	H	4.2379	-0.0205	-5.8987
66	H	4.4726	1.8323	-4.2539

step_000_t
Energy (POTENTIAL) = -2487.66577180 Eh

	Atom	X	Y	Z
1	Cu	-0.5697	-0.4198	-1.8271
2	N	1.2803	-1.0765	-2.6005
3	N	2.4127	-0.6096	-2.0020
4	N	0.2098	1.5422	-1.6242
5	N	1.5186	1.6510	-1.2610
6	N	0.2823	-0.7859	0.1250
7	N	1.5375	-0.2997	0.3401
8	B	2.3125	0.3885	-0.8211
9	C	1.6544	-1.9649	-3.5166
10	C	3.0553	-2.1028	-3.5324
11	C	3.4927	-1.2162	-2.5504
12	C	-0.2461	2.7706	-1.8541
13	C	0.7770	3.7199	-1.6592
14	C	1.8804	2.9567	-1.2778
15	C	-0.1435	-1.3062	1.2713
16	C	0.8366	-1.1698	2.2733
17	C	1.8893	-0.5214	1.6292
18	H	3.3933	0.7002	-0.4590
19	N	-2.2475	-0.8022	-2.3864
20	S	-3.8473	-0.8820	-2.8363
21	O	-4.0121	-2.0641	-3.6956
22	O	-4.2403	0.4434	-3.3389
23	C	-4.5775	-1.1762	-1.2492
24	C	-4.7460	-2.4935	-0.8088
25	C	-4.8912	-0.0820	-0.4310
26	C	-5.2507	-2.7107	0.4707
27	H	-4.4912	-3.3257	-1.4560
28	C	-5.3938	-0.3224	0.8412
29	H	-4.7520	0.9308	-0.7929
30	C	-5.5805	-1.6348	1.3130
31	H	-5.3958	-3.7287	0.8206
32	H	-5.6476	0.5164	1.4830
33	C	-6.1466	-1.8717	2.6877
34	H	-5.6989	-1.1936	3.4214
35	H	-7.2274	-1.6823	2.6920
36	H	-5.9866	-2.9012	3.0188
37	Br	-1.8598	-2.0764	1.3898
38	Br	-2.0404	3.0500	-2.3395
39	Br	0.3829	-2.8506	-4.5835
40	Br	4.0946	-3.2357	-4.6145
41	Br	5.2617	-0.8689	-2.0179

42	Br	3.5480	-0.0095	2.3567
43	Br	0.7473	-1.7323	4.0680
44	Br	0.6781	5.5876	-1.8727
45	Br	3.6136	3.5613	-0.8668
46	H	-0.8867	0.6085	-4.2725
47	Si	-0.3594	1.0768	-5.5817
48	C	-0.8007	2.8985	-5.8003
49	C	-1.1222	0.0364	-6.9540
50	C	1.5212	0.9068	-5.5629
51	H	-1.8834	3.0568	-5.7491
52	H	-0.3340	3.5181	-5.0274
53	H	-0.4516	3.2625	-6.7742
54	H	-0.8525	-1.0203	-6.8586
55	H	-2.2159	0.1024	-6.9141
56	H	-0.8037	0.3798	-7.9450
57	C	2.2875	1.6720	-4.6624
58	C	2.2100	0.0506	-6.4404
59	C	3.6797	1.5792	-4.6291
60	H	1.7919	2.3567	-3.9797
61	C	3.6050	-0.0397	-6.4203
62	H	1.6564	-0.5555	-7.1529
63	C	4.3426	0.7212	-5.5114
64	H	4.2472	2.1752	-3.9195
65	H	4.1142	-0.7070	-7.1097
66	H	5.4262	0.6462	-5.4890

step_001_t
Energy (POTENTIAL) = -2487.66569935 Eh

	Atom	X	Y	Z
1	Cu	-0.5387	-0.4010	-1.8062
2	N	1.3154	-1.0663	-2.5702
3	N	2.4459	-0.5986	-1.9689
4	N	0.2419	1.5583	-1.5908
5	N	1.5516	1.6620	-1.2290
6	N	0.3017	-0.7755	0.1471
7	N	1.5569	-0.2923	0.3691
8	B	2.3403	0.3967	-0.7864
9	C	1.6926	-1.9529	-3.4868
10	C	3.0939	-2.0879	-3.5006
11	C	3.5280	-1.2017	-2.5169
12	C	-0.2096	2.7887	-1.8198
13	C	0.8177	3.7338	-1.6269
14	C	1.9188	2.9662	-1.2472
15	C	-0.1304	-1.2984	1.2896
16	C	0.8454	-1.1671	2.2965
17	C	1.9023	-0.5188	1.6592
18	H	3.4194	0.7058	-0.4169
19	N	-2.2055	-0.7722	-2.4051
20	S	-3.8016	-0.8334	-2.8724
21	O	-3.9666	-2.0021	-3.7499
22	O	-4.1848	0.5008	-3.3585
23	C	-4.5437	-1.1461	-1.2937
24	C	-4.7119	-2.4685	-0.8685
25	C	-4.8681	-0.0617	-0.4667
26	C	-5.2274	-2.7007	0.4040
27	H	-4.4489	-3.2932	-1.5220
28	C	-5.3815	-0.3170	0.7983
29	H	-4.7290	0.9554	-0.8165
30	C	-5.5682	-1.6349	1.2547
31	H	-5.3724	-3.7228	0.7418
32	H	-5.6438	0.5141	1.4465
33	C	-6.1439	-1.8871	2.6226
34	H	-5.6863	-1.2308	3.3701
35	H	-7.2209	-1.6781	2.6274
36	H	-6.0030	-2.9251	2.9355
37	Br	-1.8490	-2.0654	1.3965
38	Br	-2.0037	3.0767	-2.3010
39	Br	0.4251	-2.8409	-4.5563
40	Br	4.1375	-3.2171	-4.5827
41	Br	5.2959	-0.8463	-1.9855

42	Br	3.5586	-0.0130	2.3964
43	Br	0.7466	-1.7349	4.0890
44	Br	0.7250	5.6020	-1.8387
45	Br	3.6548	3.5640	-0.8376
46	H	-0.9265	0.5977	-4.2360
47	Si	-0.4155	1.0517	-5.5572
48	C	-0.8619	2.8700	-5.7931
49	C	-1.1952	-0.0055	-6.9071
50	C	1.4655	0.8823	-5.5573
51	H	-1.9445	3.0266	-5.7331
52	H	-0.3890	3.5004	-5.0328
53	H	-0.5234	3.2225	-6.7749
54	H	-0.9299	-1.0621	-6.7988
55	H	-2.2881	0.0666	-6.8573
56	H	-0.8849	0.3216	-7.9061
57	C	2.2408	1.6407	-4.6588
58	C	2.1456	0.0310	-6.4462
59	C	3.6332	1.5468	-4.6389
60	H	1.7528	2.3204	-3.9659
61	C	3.5406	-0.0610	-6.4392
62	H	1.5847	-0.5706	-7.1568
63	C	4.2873	0.6938	-5.5325
64	H	4.2077	2.1379	-3.9309
65	H	4.0427	-0.7253	-7.1367
66	H	5.3709	0.6171	-5.5198

step_002_t
Energy (POTENTIAL) = -2487.66553947 Eh

	Atom	X	Y	Z
1	Cu	-0.5093	-0.3916	-1.7864
2	N	1.3443	-1.0722	-2.5451
3	N	2.4751	-0.6106	-1.9400
4	N	0.2820	1.5638	-1.5583
5	N	1.5935	1.6530	-1.1988
6	N	0.3186	-0.7828	0.1666
7	N	1.5752	-0.3059	0.3955
8	B	2.3695	0.3802	-0.7541
9	C	1.7208	-1.9519	-3.4687
10	C	3.1221	-2.0868	-3.4844
11	C	3.5569	-1.2089	-2.4936
12	C	-0.1536	2.7991	-1.7938
13	C	0.8865	3.7316	-1.6086
14	C	1.9780	2.9519	-1.2261
15	C	-0.1224	-1.3033	1.3069
16	C	0.8484	-1.1767	2.3191
17	C	1.9122	-0.5340	1.6877
18	H	3.4487	0.6804	-0.3777
19	N	-2.1678	-0.7360	-2.4249
20	S	-3.7618	-0.7739	-2.9028
21	O	-3.9333	-1.9277	-3.7987
22	O	-4.1343	0.5702	-3.3693
23	C	-4.5106	-1.1041	-1.3300
24	C	-4.6860	-2.4309	-0.9221
25	C	-4.8335	-0.0289	-0.4904
26	C	-5.2078	-2.6769	0.3454
27	H	-4.4238	-3.2487	-1.5847
28	C	-5.3529	-0.2979	0.7692
29	H	-4.6885	0.9919	-0.8267
30	C	-5.5472	-1.6206	1.2082
31	H	-5.3584	-3.7026	0.6696
32	H	-5.6141	0.5262	1.4269
33	C	-6.1278	-1.8873	2.5714
34	H	-5.6655	-1.2459	3.3289
35	H	-7.2030	-1.6686	2.5776
36	H	-5.9966	-2.9308	2.8696
37	Br	-1.8456	-2.0616	1.4048
38	Br	-1.9438	3.1117	-2.2764
39	Br	0.4534	-2.8342	-4.5431
40	Br	4.1658	-3.2047	-4.5780
41	Br	5.3254	-0.8557	-1.9625

42	Br	3.5667	-0.0369	2.4347
43	Br	0.7368	-1.7435	4.1111
44	Br	0.8202	5.5992	-1.8335
45	Br	3.7232	3.5289	-0.8258
46	H	-0.9839	0.5680	-4.2274
47	Si	-0.4814	1.0128	-5.5562
48	C	-0.9644	2.8175	-5.8215
49	C	-1.2402	-0.0771	-6.8919
50	C	1.4022	0.8754	-5.5470
51	H	-2.0490	2.9543	-5.7483
52	H	-0.4928	3.4735	-5.0825
53	H	-0.6474	3.1546	-6.8159
54	H	-0.9420	-1.1244	-6.7798
55	H	-2.3342	-0.0376	-6.8334
56	H	-0.9480	0.2529	-7.8955
57	C	2.1592	1.6345	-4.6336
58	C	2.1015	0.0429	-6.4388
59	C	3.5526	1.5602	-4.6032
60	H	1.6563	2.2984	-3.9360
61	C	3.4976	-0.0297	-6.4208
62	H	1.5548	-0.5599	-7.1595
63	C	4.2261	0.7260	-5.5002
64	H	4.1128	2.1511	-3.8837
65	H	4.0148	-0.6801	-7.1206
66	H	5.3105	0.6645	-5.4792

step_003_t
Energy (POTENTIAL) = -2487.66528131 Eh

	Atom	X	Y	Z
1	Cu	-0.4614	-0.3876	-1.8027
2	N	1.3964	-1.0611	-2.5426
3	N	2.5215	-0.5975	-1.9277
4	N	0.3125	1.5527	-1.4977
5	N	1.6225	1.6437	-1.1332
6	N	0.3546	-0.8221	0.1674
7	N	1.6101	-0.3496	0.4116
8	B	2.4059	0.3649	-0.7194
9	C	1.7824	-1.9349	-3.4681
10	C	3.1845	-2.0614	-3.4774
11	C	3.6093	-1.1868	-2.4796
12	C	-0.1376	2.7908	-1.6856
13	C	0.8898	3.7281	-1.4614
14	C	1.9902	2.9473	-1.1075
15	C	-0.1015	-1.3395	1.3025
16	C	0.8577	-1.2171	2.3266
17	C	1.9309	-0.5780	1.7080
18	H	3.4822	0.6623	-0.3326
19	N	-2.1165	-0.7417	-2.4494
20	S	-3.7060	-0.7609	-2.9443
21	O	-3.8878	-1.9189	-3.8328
22	O	-4.0598	0.5839	-3.4234
23	C	-4.4681	-1.0688	-1.3729
24	C	-4.6633	-2.3894	-0.9542
25	C	-4.7827	0.0178	-0.5449
26	C	-5.1967	-2.6173	0.3119
27	H	-4.4072	-3.2164	-1.6077
28	C	-5.3143	-0.2330	0.7133
29	H	-4.6228	1.0335	-0.8896
30	C	-5.5286	-1.5490	1.1628
31	H	-5.3625	-3.6380	0.6443
32	H	-5.5694	0.6004	1.3617
33	C	-6.1235	-1.7956	2.5236
34	H	-5.6645	-1.1479	3.2777
35	H	-7.1973	-1.5705	2.5167
36	H	-6.0018	-2.8362	2.8357
37	Br	-1.8325	-2.0832	1.3804
38	Br	-1.9302	3.0952	-2.1609
39	Br	0.5260	-2.8260	-4.5482
40	Br	4.2407	-3.1662	-4.5724
41	Br	5.3738	-0.8252	-1.9398

42	Br	3.5783	-0.0854	2.4745
43	Br	0.7224	-1.7835	4.1173
44	Br	0.7985	5.6022	-1.6124
45	Br	3.7239	3.5302	-0.6668
46	H	-1.0256	0.4518	-4.2500
47	Si	-0.5419	0.9375	-5.5728
48	C	-1.0923	2.7280	-5.7962
49	C	-1.2735	-0.1564	-6.9204
50	C	1.3448	0.8535	-5.5726
51	H	-2.1803	2.8187	-5.7027
52	H	-0.6354	3.3863	-5.0502
53	H	-0.8043	3.0968	-6.7881
54	H	-0.9428	-1.1956	-6.8240
55	H	-2.3679	-0.1508	-6.8565
56	H	-0.9957	0.1955	-7.9206
57	C	2.0877	1.6381	-4.6690
58	C	2.0595	0.0249	-6.4561
59	C	3.4825	1.5903	-4.6390
60	H	1.5731	2.3005	-3.9785
61	C	3.4565	-0.0209	-6.4388
62	H	1.5241	-0.5977	-7.1683
63	C	4.1710	0.7586	-5.5270
64	H	4.0326	2.2008	-3.9279
65	H	3.9856	-0.6690	-7.1316
66	H	5.2565	0.7178	-5.5061

step_004_t
Energy (POTENTIAL) = -2487.66500772 Eh

	Atom	X	Y	Z
1	Cu	-0.3981	-0.3761	-1.8145
2	N	1.4618	-1.0757	-2.5283
3	N	2.5838	-0.6289	-1.8958
4	N	0.3892	1.5429	-1.4597
5	N	1.7009	1.6139	-1.0978
6	N	0.3928	-0.8570	0.1691
7	N	1.6461	-0.3897	0.4348
8	B	2.4635	0.3234	-0.6800
9	C	1.8514	-1.9395	-3.4621
10	C	3.2529	-2.0739	-3.4599
11	C	3.6733	-1.2170	-2.4453
12	C	-0.0394	2.7878	-1.6569
13	C	1.0050	3.7080	-1.4428
14	C	2.0924	2.9104	-1.0856
15	C	-0.0819	-1.3767	1.2951
16	C	0.8614	-1.2611	2.3348
17	C	1.9461	-0.6235	1.7356
18	H	3.5382	0.6068	-0.2781
19	N	-2.0578	-0.7011	-2.4682
20	S	-3.6476	-0.6929	-2.9663
21	O	-3.8398	-1.8335	-3.8751
22	O	-3.9929	0.6621	-3.4221
23	C	-4.4165	-1.0190	-1.4013
24	C	-4.6268	-2.3433	-1.0033
25	C	-4.7317	0.0587	-0.5617
26	C	-5.1766	-2.5844	0.2538
27	H	-4.3717	-3.1633	-1.6659
28	C	-5.2794	-0.2050	0.6867
29	H	-4.5619	1.0777	-0.8915
30	C	-5.5096	-1.5255	1.1153
31	H	-5.3561	-3.6082	0.5691
32	H	-5.5370	0.6215	1.3430
33	C	-6.1171	-1.7832	2.4684
34	H	-5.5752	-1.2413	3.2513
35	H	-7.1552	-1.4305	2.4957
36	H	-6.1143	-2.8475	2.7173
37	Br	-1.8159	-2.1159	1.3445
38	Br	-1.8232	3.1210	-2.1469
39	Br	0.6023	-2.8153	-4.5633
40	Br	4.3150	-3.1601	-4.5677
41	Br	5.4350	-0.8732	-1.8846

42	Br	3.5824	-0.1385	2.5308
43	Br	0.6957	-1.8335	4.1210
44	Br	0.9479	5.5817	-1.6118
45	Br	3.8400	3.4642	-0.6642
46	H	-1.1326	0.4271	-4.2721
47	Si	-0.6518	0.9077	-5.5993
48	C	-1.2548	2.6760	-5.8554
49	C	-1.3336	-0.2307	-6.9356
50	C	1.2356	0.8729	-5.5622
51	H	-2.3442	2.7357	-5.7536
52	H	-0.8114	3.3626	-5.1273
53	H	-0.9865	3.0312	-6.8577
54	H	-0.9699	-1.2568	-6.8191
55	H	-2.4281	-0.2596	-6.8826
56	H	-1.0568	0.1142	-7.9386
57	C	1.9359	1.6653	-4.6321
58	C	1.9921	0.0659	-6.4308
59	C	3.3297	1.6453	-4.5611
60	H	1.3884	2.3093	-3.9498
61	C	3.3886	0.0481	-6.3729
62	H	1.4901	-0.5631	-7.1614
63	C	4.0604	0.8343	-5.4343
64	H	3.8453	2.2602	-3.8284
65	H	3.9506	-0.5838	-7.0548
66	H	5.1454	0.8139	-5.3813

step_005_t
Energy (POTENTIAL) = -2487.66458227 Eh

	Atom	X	Y	Z
1	Cu	-0.3925	-0.2761	-1.7747
2	N	1.4602	-1.0246	-2.4429
3	N	2.5846	-0.5624	-1.8278
4	N	0.4240	1.6606	-1.4816
5	N	1.7286	1.7127	-1.0882
6	N	0.3581	-0.6970	0.2223
7	N	1.6240	-0.2632	0.4852
8	B	2.4634	0.4189	-0.6343
9	C	1.8415	-1.9281	-3.3413
10	C	3.2421	-2.0709	-3.3365
11	C	3.6695	-1.1789	-2.3551
12	C	0.0254	2.9116	-1.7060
13	C	1.0822	3.8145	-1.4774
14	C	2.1446	3.0019	-1.0839
15	C	-0.1202	-1.2270	1.3422
16	C	0.8337	-1.1507	2.3759
17	C	1.9287	-0.5285	1.7789
18	H	3.5376	0.6922	-0.2246
19	N	-2.0318	-0.5549	-2.4891
20	S	-3.6166	-0.5687	-3.0034
21	O	-3.7650	-1.6675	-3.9703
22	O	-4.0031	0.7953	-3.3934
23	C	-4.3844	-0.9975	-1.4618
24	C	-4.5659	-2.3457	-1.1385
25	C	-4.7215	0.0254	-0.5640
26	C	-5.1104	-2.6680	0.1027
27	H	-4.2926	-3.1219	-1.8452
28	C	-5.2621	-0.3191	0.6677
29	H	-4.5688	1.0643	-0.8356
30	C	-5.4640	-1.6662	1.0221
31	H	-5.2671	-3.7112	0.3611
32	H	-5.5338	0.4641	1.3698
33	C	-6.0568	-2.0127	2.3622
34	H	-5.5142	-1.5122	3.1717
35	H	-7.0988	-1.6759	2.4204
36	H	-6.0379	-3.0898	2.5469
37	Br	-1.8670	-1.9367	1.3895
38	Br	-1.7378	3.2888	-2.2438
39	Br	0.5774	-2.8468	-4.3879
40	Br	4.2945	-3.2027	-4.4072
41	Br	5.4335	-0.8234	-1.8087

42	Br	3.5833	-0.1001	2.5685
43	Br	0.6677	-1.7491	4.1535
44	Br	1.0671	5.6874	-1.6663
45	Br	3.8906	3.5323	-0.6258
46	H	-1.1604	0.6275	-4.1539
47	Si	-0.6947	0.9649	-5.5312
48	C	-1.2013	2.7395	-5.9189
49	C	-1.4892	-0.2449	-6.7353
50	C	1.1870	0.8124	-5.5448
51	H	-2.2824	2.8728	-5.7998
52	H	-0.6992	3.4531	-5.2577
53	H	-0.9398	2.9974	-6.9522
54	H	-1.2385	-1.2811	-6.4866
55	H	-2.5803	-0.1504	-6.6996
56	H	-1.1725	-0.0551	-7.7673
57	C	1.9679	1.5636	-4.6448
58	C	1.8609	-0.0386	-6.4392
59	C	3.3602	1.4653	-4.6320
60	H	1.4871	2.2357	-3.9400
61	C	3.2552	-0.1377	-6.4365
62	H	1.2945	-0.6374	-7.1477
63	C	4.0077	0.6121	-5.5303
64	H	3.9401	2.0517	-3.9245
65	H	3.7522	-0.8033	-7.1367
66	H	5.0910	0.5299	-5.5201

step_006_t

Energy (POTENTIAL) = -2487.66396743 Eh

	Atom	X	Y	Z
1	Cu	-0.3949	-0.2768	-1.7825
2	N	1.4531	-1.0598	-2.4448
3	N	2.5836	-0.6047	-1.8350
4	N	0.4432	1.6493	-1.4819
5	N	1.7563	1.6874	-1.1183
6	N	0.3622	-0.7026	0.2195
7	N	1.6302	-0.2698	0.4758
8	B	2.4759	0.3898	-0.6524
9	C	1.8240	-1.9811	-3.3292
10	C	3.2225	-2.1428	-3.3208
11	C	3.6609	-1.2426	-2.3522
12	C	0.0560	2.9033	-1.7079
13	C	1.1301	3.7939	-1.5133
14	C	2.1905	2.9702	-1.1370
15	C	-0.1183	-1.2103	1.3488
16	C	0.8350	-1.1195	2.3818
17	C	1.9330	-0.5130	1.7744
18	H	3.5540	0.6549	-0.2475
19	N	-2.0327	-0.4839	-2.5322
20	S	-3.6293	-0.4737	-3.0122
21	O	-3.8040	-1.5454	-4.0050
22	O	-4.0197	0.9015	-3.3567
23	C	-4.3706	-0.9415	-1.4678
24	C	-4.5460	-2.2976	-1.1768
25	C	-4.7009	0.0575	-0.5414
26	C	-5.0760	-2.6527	0.0618
27	H	-4.2796	-3.0549	-1.9062
28	C	-5.2274	-0.3194	0.6872
29	H	-4.5546	1.1034	-0.7883
30	C	-5.4214	-1.6756	1.0101
31	H	-5.2266	-3.7026	0.2958
32	H	-5.4931	0.4450	1.4119
33	C	-5.9948	-2.0576	2.3492
34	H	-5.4430	-1.5753	3.1636
35	H	-7.0370	-1.7266	2.4307
36	H	-5.9694	-3.1389	2.5070
37	Br	-1.8679	-1.9117	1.4086
38	Br	-1.7136	3.2958	-2.2117
39	Br	0.5515	-2.8973	-4.3677
40	Br	4.2607	-3.2994	-4.3793
41	Br	5.4295	-0.8980	-1.8137

42	Br	3.5892	-0.0793	2.5580
43	Br	0.6652	-1.6841	4.1701
44	Br	1.1361	5.6643	-1.7242
45	Br	3.9572	3.4764	-0.7353
46	H	-1.2279	0.5468	-4.2010
47	Si	-0.7363	0.9334	-5.5630
48	C	-1.2835	2.7020	-5.9170
49	C	-1.4694	-0.2751	-6.8058
50	C	1.1474	0.8272	-5.5268
51	H	-2.3673	2.8061	-5.7950
52	H	-0.7992	3.4150	-5.2423
53	H	-1.0278	2.9844	-6.9453
54	H	-1.1790	-1.3062	-6.5794
55	H	-2.5638	-0.2273	-6.7783
56	H	-1.1510	-0.0469	-7.8294
57	C	1.8884	1.6478	-4.6541
58	C	1.8618	-0.0670	-6.3448
59	C	3.2806	1.5711	-4.5888
60	H	1.3765	2.3591	-4.0124
61	C	3.2565	-0.1413	-6.2933
62	H	1.3274	-0.7189	-7.0309
63	C	3.9687	0.6737	-5.4106
64	H	3.8278	2.2096	-3.9004
65	H	3.7855	-0.8391	-6.9361
66	H	5.0521	0.6096	-5.3617

step_007_t
Energy (POTENTIAL) = -2487.66323766 Eh

	Atom	X	Y	Z
1	Cu	-0.3830	-0.2600	-1.7874
2	N	1.4648	-1.0570	-2.4369
3	N	2.5952	-0.6061	-1.8239
4	N	0.4567	1.6518	-1.4605
5	N	1.7716	1.6878	-1.1057
6	N	0.3702	-0.7046	0.2243
7	N	1.6372	-0.2706	0.4849
8	B	2.4877	0.3875	-0.6404
9	C	1.8342	-1.9841	-3.3157
10	C	3.2319	-2.1535	-3.3010
11	C	3.6711	-1.2521	-2.3340
12	C	0.0676	2.9061	-1.6803
13	C	1.1432	3.7958	-1.4908
14	C	2.2061	2.9704	-1.1243
15	C	-0.1165	-1.2037	1.3544
16	C	0.8310	-1.1066	2.3923
17	C	1.9324	-0.5047	1.7871
18	H	3.5658	0.6498	-0.2331
19	N	-2.0140	-0.4268	-2.5734
20	S	-3.6169	-0.4143	-3.0333
21	O	-3.8013	-1.4749	-4.0368
22	O	-4.0173	0.9632	-3.3583
23	C	-4.3470	-0.9023	-1.4887
24	C	-4.5160	-2.2622	-1.2129
25	C	-4.6855	0.0844	-0.5526
26	C	-5.0465	-2.6338	0.0209
27	H	-4.2449	-3.0099	-1.9504
28	C	-5.2120	-0.3087	0.6710
29	H	-4.5472	1.1339	-0.7882
30	C	-5.3989	-1.6691	0.9790
31	H	-5.1916	-3.6871	0.2431
32	H	-5.4837	0.4464	1.4032
33	C	-5.9725	-2.0689	2.3129
34	H	-5.4316	-1.5845	3.1333
35	H	-7.0200	-1.7542	2.3923
36	H	-5.9323	-3.1508	2.4632
37	Br	-1.8682	-1.9001	1.4087
38	Br	-1.7062	3.2922	-2.1732
39	Br	0.5607	-2.8990	-4.3542
40	Br	4.2681	-3.3177	-4.3530
41	Br	5.4396	-0.9135	-1.7909

42	Br	3.5842	-0.0659	2.5776
43	Br	0.6506	-1.6596	4.1834
44	Br	1.1477	5.6671	-1.6943
45	Br	3.9763	3.4734	-0.7342
46	H	-1.2594	0.4773	-4.2149
47	Si	-0.7642	0.8962	-5.5732
48	C	-1.3331	2.6641	-5.8903
49	C	-1.4792	-0.3032	-6.8342
50	C	1.1193	0.8066	-5.5231
51	H	-2.4157	2.7546	-5.7494
52	H	-0.8451	3.3704	-5.2114
53	H	-1.0951	2.9648	-6.9177
54	H	-1.1795	-1.3340	-6.6184
55	H	-2.5739	-0.2664	-6.8119
56	H	-1.1566	-0.0593	-7.8530
57	C	1.8482	1.6317	-4.6445
58	C	1.8453	-0.0843	-6.3348
59	C	3.2401	1.5620	-4.5669
60	H	1.3274	2.3403	-4.0072
61	C	3.2398	-0.1511	-6.2711
62	H	1.3204	-0.7393	-7.0252
63	C	3.9399	0.6678	-5.3822
64	H	3.7777	2.2033	-3.8737
65	H	3.7780	-0.8462	-6.9092
66	H	5.0231	0.6090	-5.3236

step_008_t
Energy (POTENTIAL) = -2487.66353570 Eh

	Atom	X	Y	Z
1	Cu	-0.3626	-0.4679	-1.9823
2	N	1.4875	-1.3257	-2.6052
3	N	2.6232	-0.8676	-2.0088
4	N	0.5126	1.3805	-1.5822
5	N	1.8046	1.3900	-1.1481
6	N	0.3727	-1.0106	0.0466
7	N	1.6672	-0.6666	0.3087
8	B	2.5172	0.0629	-0.7722
9	C	1.8482	-2.2679	-3.4696
10	C	3.2459	-2.4382	-3.4672
11	C	3.6945	-1.5238	-2.5163
12	C	0.1139	2.6468	-1.6751
13	C	1.1545	3.5201	-1.3098
14	C	2.2107	2.6716	-0.9824
15	C	-0.0946	-1.6278	1.1254
16	C	0.8938	-1.7065	2.1264
17	C	1.9996	-1.0797	1.5562
18	H	3.5961	0.2955	-0.3506
19	N	-1.9783	-0.6523	-2.8165
20	S	-3.6109	-0.4868	-3.1217
21	O	-4.0284	-1.6056	-3.9819
22	O	-3.8768	0.8933	-3.5591
23	C	-4.2538	-0.7184	-1.4803
24	C	-4.7375	-1.9728	-1.1017
25	C	-4.2532	0.3656	-0.5920
26	C	-5.2452	-2.1337	0.1868
27	H	-4.7299	-2.7975	-1.8060
28	C	-4.7602	0.1824	0.6879
29	H	-3.8751	1.3320	-0.9059
30	C	-5.2643	-1.0651	1.0979
31	H	-5.6399	-3.1002	0.4869
32	H	-4.7706	1.0173	1.3829
33	C	-5.7898	-1.2452	2.4977
34	H	-4.9670	-1.2244	3.2233
35	H	-6.4743	-0.4344	2.7685
36	H	-6.3157	-2.1966	2.6127
37	Br	-1.8764	-2.2395	1.1738
38	Br	-1.6244	3.0695	-2.2531
39	Br	0.5639	-3.1763	-4.5024
40	Br	4.2732	-3.5982	-4.5352
41	Br	5.4673	-1.1820	-1.9897

42	Br	3.6982	-0.8152	2.3255
43	Br	0.7529	-2.4806	3.8379
44	Br	1.1380	5.4013	-1.3095
45	Br	3.9410	3.1539	-0.4268
46	H	-1.3213	-0.2583	-4.5772
47	Si	-0.7894	0.4638	-5.8050
48	C	-1.9240	1.9263	-6.1349
49	C	-0.7833	-0.7460	-7.2481
50	C	0.9563	1.0339	-5.3763
51	H	-2.9431	1.5767	-6.3293
52	H	-1.9685	2.6021	-5.2764
53	H	-1.5832	2.4973	-7.0067
54	H	-0.1094	-1.5906	-7.0764
55	H	-1.7895	-1.1493	-7.4094
56	H	-0.4682	-0.2441	-8.1707
57	C	1.1876	2.2966	-4.7979
58	C	2.0717	0.2011	-5.5888
59	C	2.4712	2.7019	-4.4252
60	H	0.3569	2.9778	-4.6364
61	C	3.3572	0.5986	-5.2161
62	H	1.9400	-0.7733	-6.0515
63	C	3.5590	1.8482	-4.6243
64	H	2.6226	3.6824	-3.9831
65	H	4.2006	-0.0633	-5.3912
66	H	4.5580	2.1585	-4.3306

step_009_t
Energy (POTENTIAL) = -2487.66261518 Eh

	Atom	X	Y	Z
1	Cu	-0.3625	-0.4457	-1.9598
2	N	1.4910	-1.3140	-2.6040
3	N	2.6317	-0.8563	-2.0174
4	N	0.5239	1.3953	-1.5633
5	N	1.8202	1.3998	-1.1423
6	N	0.3915	-1.0016	0.0534
7	N	1.6897	-0.6634	0.3059
8	B	2.5337	0.0699	-0.7770
9	C	1.8444	-2.2573	-3.4698
10	C	3.2420	-2.4291	-3.4785
11	C	3.6988	-1.5139	-2.5324
12	C	0.1298	2.6631	-1.6548
13	C	1.1776	3.5325	-1.3012
14	C	2.2334	2.6800	-0.9829
15	C	-0.0670	-1.6260	1.1319
16	C	0.9306	-1.7156	2.1226
17	C	2.0327	-1.0878	1.5467
18	H	3.6154	0.3000	-0.3607
19	N	-1.9540	-0.6173	-2.8561
20	S	-3.5935	-0.4534	-3.1219
21	O	-4.0243	-1.5596	-3.9927
22	O	-3.8744	0.9322	-3.5338
23	C	-4.2202	-0.7102	-1.4768
24	C	-4.6949	-1.9719	-1.1114
25	C	-4.2238	0.3626	-0.5760
26	C	-5.1962	-2.1516	0.1769
27	H	-4.6822	-2.7888	-1.8246
28	C	-4.7249	0.1611	0.7040
29	H	-3.8501	1.3343	-0.8788
30	C	-5.2208	-1.0937	1.1004
31	H	-5.5794	-3.1254	0.4685
32	H	-4.7339	0.9866	1.4101
33	C	-5.7802	-1.2863	2.4854
34	H	-5.1693	-0.7724	3.2344
35	H	-6.7923	-0.8674	2.5516
36	H	-5.8404	-2.3451	2.7519
37	Br	-1.8503	-2.2303	1.1924
38	Br	-1.6120	3.0907	-2.2190
39	Br	0.5502	-3.1663	-4.4907
40	Br	4.2599	-3.5916	-4.5533
41	Br	5.4760	-1.1714	-2.0209

42	Br	3.7399	-0.8364	2.3015
43	Br	0.8028	-2.5012	3.8298
44	Br	1.1686	5.4137	-1.3030
45	Br	3.9712	3.1562	-0.4452
46	H	-1.3541	-0.2460	-4.5339
47	Si	-0.8312	0.4670	-5.7887
48	C	-1.9685	1.9292	-6.1057
49	C	-0.8528	-0.7564	-7.2188
50	C	0.9187	1.0294	-5.3758
51	H	-2.9905	1.5808	-6.2850
52	H	-1.9996	2.6078	-5.2489
53	H	-1.6372	2.4958	-6.9842
54	H	-0.1760	-1.5995	-7.0530
55	H	-1.8622	-1.1594	-7.3591
56	H	-0.5539	-0.2604	-8.1501
57	C	1.1567	2.2894	-4.7938
58	C	2.0307	0.1960	-5.6043
59	C	2.4447	2.6924	-4.4349
60	H	0.3282	2.9699	-4.6196
61	C	3.3205	0.5916	-5.2450
62	H	1.8930	-0.7766	-6.0688
63	C	3.5296	1.8388	-4.6505
64	H	2.6021	3.6709	-3.9907
65	H	4.1616	-0.0699	-5.4316
66	H	4.5321	2.1473	-4.3675

step_010_t
Energy (POTENTIAL) = -2487.66190373 Eh

	Atom	X	Y	Z
1	Cu	-0.3867	-0.4037	-1.9143
2	N	1.4843	-1.2766	-2.5914
3	N	2.6254	-0.8184	-2.0061
4	N	0.5058	1.4359	-1.5204
5	N	1.8067	1.4310	-1.1116
6	N	0.3825	-0.9811	0.0657
7	N	1.6817	-0.6458	0.3175
8	B	2.5235	0.0982	-0.7600
9	C	1.8369	-2.2253	-3.4502
10	C	3.2346	-2.4010	-3.4566
11	C	3.6923	-1.4819	-2.5153
12	C	0.1214	2.7074	-1.6098
13	C	1.1793	3.5687	-1.2666
14	C	2.2311	2.7081	-0.9570
15	C	-0.0730	-1.6165	1.1395
16	C	0.9278	-1.7168	2.1255
17	C	2.0284	-1.0836	1.5525
18	H	3.6040	0.3282	-0.3407
19	N	-1.9204	-0.5995	-2.9180
20	S	-3.5632	-0.4217	-3.1477
21	O	-4.0174	-1.5088	-4.0317
22	O	-3.8424	0.9731	-3.5322
23	C	-4.1852	-0.6982	-1.5021
24	C	-4.6673	-1.9611	-1.1527
25	C	-4.1936	0.3654	-0.5914
26	C	-5.1809	-2.1514	0.1295
27	H	-4.6529	-2.7710	-1.8739
28	C	-4.7079	0.1542	0.6823
29	H	-3.8153	1.3387	-0.8829
30	C	-5.2128	-1.1017	1.0620
31	H	-5.5701	-3.1266	0.4082
32	H	-4.7213	0.9737	1.3953
33	C	-5.7938	-1.3046	2.4370
34	H	-5.2068	-0.7801	3.1976
35	H	-6.8147	-0.9050	2.4840
36	H	-5.8398	-2.3645	2.7020
37	Br	-1.8565	-2.2176	1.2003
38	Br	-1.6210	3.1551	-2.1586
39	Br	0.5406	-3.1395	-4.4648
40	Br	4.2511	-3.5685	-4.5274
41	Br	5.4698	-1.1383	-2.0046

42	Br	3.7385	-0.8426	2.3036
43	Br	0.8045	-2.5187	3.8253
44	Br	1.1840	5.4502	-1.2644
45	Br	3.9763	3.1735	-0.4332
46	H	-1.3546	-0.2814	-4.5109
47	Si	-0.8334	0.4365	-5.7882
48	C	-1.9687	1.9061	-6.0707
49	C	-0.8832	-0.7837	-7.2186
50	C	0.9182	0.9843	-5.3764
51	H	-2.9891	1.5649	-6.2700
52	H	-2.0074	2.5562	-5.1927
53	H	-1.6274	2.4984	-6.9281
54	H	-0.2047	-1.6282	-7.0682
55	H	-1.8959	-1.1832	-7.3428
56	H	-0.5998	-0.2832	-8.1525
57	C	1.1557	2.2263	-4.7559
58	C	2.0307	0.1632	-5.6439
59	C	2.4459	2.6246	-4.4012
60	H	0.3254	2.8957	-4.5509
61	C	3.3226	0.5550	-5.2882
62	H	1.8921	-0.7963	-6.1344
63	C	3.5322	1.7845	-4.6582
64	H	2.6042	3.5894	-3.9288
65	H	4.1648	-0.0962	-5.5038
66	H	4.5366	2.0894	-4.3780

step_011_t
Energy (POTENTIAL) = -2487.66164566 Eh

	Atom	X	Y	Z
1	Cu	-0.4715	-0.3841	-1.8726
2	N	1.4379	-1.2431	-2.5649
3	N	2.5659	-0.7808	-1.9578
4	N	0.4104	1.4640	-1.4802
5	N	1.7131	1.4607	-1.0746
6	N	0.3126	-1.0132	0.0713
7	N	1.5907	-0.6217	0.3520
8	B	2.4352	0.1352	-0.7137
9	C	1.8101	-2.1963	-3.4087
10	C	3.2081	-2.3726	-3.3835
11	C	3.6447	-1.4477	-2.4384
12	C	0.0415	2.7371	-1.6145
13	C	1.1090	3.5979	-1.3025
14	C	2.1519	2.7372	-0.9655
15	C	-0.1162	-1.7208	1.1117
16	C	0.8775	-1.8041	2.1063
17	C	1.9474	-1.0894	1.5730
18	H	3.5071	0.3763	-0.2791
19	N	-1.9075	-0.5896	-3.0233
20	S	-3.5549	-0.4046	-3.1986
21	O	-4.0446	-1.4837	-4.0750
22	O	-3.8440	0.9930	-3.5698
23	C	-4.1379	-0.6859	-1.5369
24	C	-4.7372	-1.9037	-1.2154
25	C	-4.0098	0.3370	-0.5886
26	C	-5.2315	-2.0888	0.0766
27	H	-4.8301	-2.6812	-1.9657
28	C	-4.5015	0.1299	0.6940
29	H	-3.5463	1.2793	-0.8577
30	C	-5.1235	-1.0811	1.0464
31	H	-5.7165	-3.0271	0.3307
32	H	-4.4102	0.9192	1.4351
33	C	-5.6457	-1.2825	2.4452
34	H	-4.8243	-1.2703	3.1719
35	H	-6.3321	-0.4763	2.7271
36	H	-6.1740	-2.2342	2.5466
37	Br	-1.8473	-2.4615	1.1228
38	Br	-1.6907	3.1940	-2.1858
39	Br	0.5354	-3.1156	-4.4471
40	Br	4.2479	-3.5452	-4.4264
41	Br	5.4109	-1.0971	-1.8930

42	Br	3.6321	-0.7843	2.3572
43	Br	0.7789	-2.6725	3.7748
44	Br	1.1382	5.4779	-1.3709
45	Br	3.9039	3.2031	-0.4664
46	H	-1.3588	-0.2970	-4.5194
47	Si	-0.8121	0.4211	-5.8226
48	C	-1.9557	1.8825	-6.1069
49	C	-0.8489	-0.8119	-7.2405
50	C	0.9283	0.9677	-5.3774
51	H	-2.9695	1.5353	-6.3278
52	H	-2.0142	2.5216	-5.2221
53	H	-1.6036	2.4864	-6.9521
54	H	-0.1617	-1.6478	-7.0837
55	H	-1.8576	-1.2216	-7.3629
56	H	-0.5698	-0.3141	-8.1774
57	C	1.1516	2.2047	-4.7409
58	C	2.0465	0.1504	-5.6334
59	C	2.4352	2.6023	-4.3629
60	H	0.3164	2.8705	-4.5435
61	C	3.3311	0.5416	-5.2529
62	H	1.9173	-0.8059	-6.1326
63	C	3.5274	1.7664	-4.6096
64	H	2.5841	3.5629	-3.8794
65	H	4.1781	-0.1066	-5.4585
66	H	4.5261	2.0707	-4.3093

step_012_t
Energy (POTENTIAL) = -2487.66199702 Eh

	Atom	X	Y	Z
1	Cu	-0.5067	-0.3800	-1.8045
2	N	1.4081	-1.2488	-2.5715
3	N	2.5467	-0.7846	-1.9852
4	N	0.4034	1.4740	-1.4694
5	N	1.7153	1.4638	-1.0944
6	N	0.3279	-0.9948	0.0974
7	N	1.6163	-0.6129	0.3425
8	B	2.4401	0.1370	-0.7441
9	C	1.7683	-2.1999	-3.4224
10	C	3.1670	-2.3739	-3.4227
11	C	3.6186	-1.4491	-2.4850
12	C	0.0443	2.7486	-1.6155
13	C	1.1265	3.6033	-1.3392
14	C	2.1684	2.7375	-1.0131
15	C	-0.0791	-1.6947	1.1528
16	C	0.9407	-1.7833	2.1195
17	C	2.0016	-1.0795	1.5548
18	H	3.5202	0.3777	-0.3303
19	N	-1.8887	-0.5713	-3.0378
20	S	-3.5373	-0.3760	-3.1742
21	O	-4.0429	-1.4425	-4.0590
22	O	-3.8378	1.0259	-3.5230
23	C	-4.1134	-0.6771	-1.5113
24	C	-4.6839	-1.9095	-1.1947
25	C	-4.0174	0.3468	-0.5608
26	C	-5.1789	-2.1105	0.0950
27	H	-4.7557	-2.6871	-1.9473
28	C	-4.5101	0.1246	0.7194
29	H	-3.5796	1.3021	-0.8271
30	C	-5.1019	-1.1025	1.0671
31	H	-5.6403	-3.0618	0.3450
32	H	-4.4437	0.9152	1.4620
33	C	-5.6256	-1.3208	2.4632
34	H	-4.8069	-1.3042	3.1930
35	H	-6.3231	-0.5258	2.7495
36	H	-6.1420	-2.2799	2.5558
37	Br	-1.8168	-2.4149	1.2201
38	Br	-1.6934	3.2131	-2.1630
39	Br	0.4798	-3.1236	-4.4411
40	Br	4.1907	-3.5429	-4.4857
41	Br	5.3939	-1.0991	-1.9682

42	Br	3.7083	-0.7858	2.2938
43	Br	0.8814	-2.6474	3.7918
44	Br	1.1730	5.4819	-1.4363
45	Br	3.9355	3.1942	-0.5611
46	H	-1.3702	-0.2845	-4.4377
47	Si	-0.8328	0.4227	-5.8018
48	C	-1.9873	1.8785	-6.0671
49	C	-0.9194	-0.8330	-7.1963
50	C	0.9130	0.9653	-5.3866
51	H	-3.0057	1.5263	-6.2559
52	H	-2.0228	2.5282	-5.1890
53	H	-1.6589	2.4719	-6.9293
54	H	-0.1646	-1.6183	-7.1030
55	H	-1.9055	-1.3095	-7.2191
56	H	-0.7676	-0.3280	-8.1587
57	C	1.1453	2.2033	-4.7546
58	C	2.0264	0.1453	-5.6559
59	C	2.4345	2.6010	-4.3976
60	H	0.3131	2.8692	-4.5446
61	C	3.3166	0.5381	-5.2973
62	H	1.8893	-0.8129	-6.1490
63	C	3.5228	1.7650	-4.6611
64	H	2.5912	3.5620	-3.9172
65	H	4.1602	-0.1109	-5.5142
66	H	4.5261	2.0701	-4.3776

step_013_t
Energy (POTENTIAL) = -2487.66255280 Eh

	Atom	X	Y	Z
1	Cu	-0.5690	-0.3154	-1.6409
2	N	1.4190	-1.0898	-2.4475
3	N	2.5251	-0.6011	-1.8205
4	N	0.2680	1.5781	-1.2947
5	N	1.5920	1.6091	-0.9671
6	N	0.2758	-0.8970	0.2334
7	N	1.5428	-0.4575	0.4944
8	B	2.3582	0.3103	-0.5834
9	C	1.8435	-1.9465	-3.3675
10	C	3.2510	-2.0319	-3.3686
11	C	3.6395	-1.1530	-2.3618
12	C	-0.1119	2.8350	-1.5270
13	C	0.9729	3.7172	-1.3681
14	C	2.0351	2.8880	-1.0106
15	C	-0.1336	-1.5714	1.3049
16	C	0.8646	-1.5876	2.2970
17	C	1.9134	-0.8656	1.7323
18	H	3.4190	0.5980	-0.1496
19	N	-1.8490	-0.4901	-2.9883
20	S	-3.5011	-0.4129	-3.1666
21	O	-3.8957	-1.5081	-4.0745
22	O	-3.9018	0.9635	-3.5214
23	C	-4.1193	-0.7708	-1.5288
24	C	-4.5723	-2.0556	-1.2314
25	C	-4.1774	0.2582	-0.5815
26	C	-5.1008	-2.3071	0.0361
27	H	-4.5263	-2.8380	-1.9811
28	C	-4.7028	-0.0129	0.6763
29	H	-3.8300	1.2535	-0.8338
30	C	-5.1772	-1.2953	1.0043
31	H	-5.4662	-3.3028	0.2715
32	H	-4.7523	0.7808	1.4170
33	C	-5.7504	-1.5637	2.3722
34	H	-5.0488	-1.2611	3.1577
35	H	-6.6715	-0.9896	2.5282
36	H	-5.9845	-2.6226	2.5110
37	Br	-1.8537	-2.3330	1.3625
38	Br	-1.8780	3.2459	-2.0255
39	Br	0.6257	-2.8927	-4.4507
40	Br	4.3521	-3.0796	-4.4778
41	Br	5.3873	-0.7356	-1.8021

42	Br	3.5884	-0.4837	2.5026
43	Br	0.7947	-2.3958	3.9964
44	Br	0.9892	5.5853	-1.5949
45	Br	3.8130	3.3915	-0.6576
46	H	-1.3252	-0.1211	-4.2557
47	Si	-0.8000	0.5759	-5.7032
48	C	-1.6952	2.2259	-5.7629
49	C	-1.3061	-0.6036	-7.0731
50	C	1.0530	0.7713	-5.5255
51	H	-2.7556	2.0960	-5.5292
52	H	-1.2745	2.9323	-5.0417
53	H	-1.6010	2.6655	-6.7639
54	H	-0.8189	-1.5775	-6.9679
55	H	-2.3894	-0.7638	-7.0492
56	H	-1.0460	-0.1941	-8.0571
57	C	1.5884	1.6674	-4.5784
58	C	1.9526	0.0253	-6.3116
59	C	2.9665	1.8114	-4.4201
60	H	0.9260	2.2582	-3.9531
61	C	3.3327	0.1770	-6.1651
62	H	1.5760	-0.6785	-7.0487
63	C	3.8421	1.0664	-5.2155
64	H	3.3582	2.5034	-3.6805
65	H	4.0097	-0.4021	-6.7861
66	H	4.9158	1.1777	-5.0943

step_014_t
Energy (POTENTIAL) = -2487.66549688 Eh

	Atom	X	Y	Z
1	Cu	-0.5507	-0.3911	-1.7659
2	N	1.3767	-1.2980	-2.5789
3	N	2.5184	-0.8274	-2.0031
4	N	0.4016	1.4484	-1.4781
5	N	1.7092	1.4228	-1.0891
6	N	0.2973	-1.0169	0.0970
7	N	1.5966	-0.6641	0.3294
8	B	2.4224	0.0866	-0.7556
9	C	1.7364	-2.2419	-3.4379
10	C	3.1371	-2.4035	-3.4553
11	C	3.5910	-1.4794	-2.5184
12	C	0.0559	2.7276	-1.6191
13	C	1.1419	3.5694	-1.3200
14	C	2.1720	2.6917	-0.9895
15	C	-0.1086	-1.7278	1.1469
16	C	0.9231	-1.8512	2.0962
17	C	1.9895	-1.1589	1.5275
18	H	3.5056	0.3145	-0.3431
19	N	-1.8759	-0.5543	-3.0783
20	S	-3.5227	-0.3293	-3.1568
21	O	-4.0652	-1.3800	-4.0412
22	O	-3.8214	1.0782	-3.4889
23	C	-4.0875	-0.6349	-1.4887
24	C	-4.7000	-1.8498	-1.1861
25	C	-3.9609	0.3744	-0.5267
26	C	-5.2006	-2.0507	0.1020
27	H	-4.7983	-2.6153	-1.9480
28	C	-4.4598	0.1532	0.7517
29	H	-3.4946	1.3193	-0.7824
30	C	-5.0923	-1.0574	1.0854
31	H	-5.6923	-2.9898	0.3404
32	H	-4.3677	0.9328	1.5033
33	C	-5.6253	-1.2742	2.4786
34	H	-4.8118	-1.2595	3.2141
35	H	-6.3236	-0.4781	2.7603
36	H	-6.1449	-2.2320	2.5675
37	Br	-1.8575	-2.4157	1.2327
38	Br	-1.6663	3.2112	-2.1937
39	Br	0.4472	-3.1802	-4.4457
40	Br	4.1605	-3.5615	-4.5311
41	Br	5.3695	-1.1157	-2.0221

42	Br	3.7125	-0.9142	2.2455
43	Br	0.8708	-2.7419	3.7542
44	Br	1.2068	5.4479	-1.4002
45	Br	3.9352	3.1305	-0.5052
46	H	-1.4251	-0.3374	-4.3000
47	Si	-0.7948	0.3921	-5.8542
48	C	-2.0079	1.8011	-6.1289
49	C	-0.8021	-0.8798	-7.2381
50	C	0.9156	0.9920	-5.3892
51	H	-2.9888	1.4081	-6.4132
52	H	-2.1433	2.3930	-5.2204
53	H	-1.6540	2.4608	-6.9313
54	H	-0.0017	-1.6167	-7.1312
55	H	-1.7569	-1.4155	-7.2669
56	H	-0.6722	-0.3734	-8.2036
57	C	1.0946	2.2572	-4.7925
58	C	2.0582	0.1893	-5.5833
59	C	2.3597	2.6999	-4.4047
60	H	0.2391	2.9083	-4.6364
61	C	3.3232	0.6266	-5.1903
62	H	1.9625	-0.7888	-6.0463
63	C	3.4766	1.8818	-4.5950
64	H	2.4749	3.6824	-3.9571
65	H	4.1895	-0.0085	-5.3510
66	H	4.4611	2.2222	-4.2873

step_015_t
Energy (POTENTIAL) = -2487.66982023 Eh

	Atom	X	Y	Z
1	Cu	-0.6862	-0.3576	-1.5029
2	N	1.2411	-1.1915	-2.4528
3	N	2.3898	-0.6950	-1.9140
4	N	0.2151	1.5349	-1.3517
5	N	1.5477	1.5565	-1.0597
6	N	0.2438	-0.8231	0.3323
7	N	1.5566	-0.4604	0.4579
8	B	2.3152	0.2517	-0.6996
9	C	1.6007	-2.0596	-3.3903
10	C	3.0048	-2.1491	-3.4873
11	C	3.4639	-1.2566	-2.5231
12	C	-0.1703	2.7966	-1.5456
13	C	0.9202	3.6738	-1.3956
14	C	1.9899	2.8366	-1.0822
15	C	-0.1246	-1.3621	1.4953
16	C	0.9495	-1.3720	2.4032
17	C	1.9967	-0.7866	1.6961
18	H	3.4050	0.5316	-0.3406
19	N	-1.8865	-0.5517	-2.9380
20	S	-3.5314	-0.3956	-3.1250
21	O	-3.9541	-1.4540	-4.0653
22	O	-3.9040	0.9965	-3.4486
23	C	-4.1669	-0.7811	-1.4990
24	C	-4.5219	-2.0965	-1.1976
25	C	-4.3283	0.2467	-0.5646
26	C	-5.0510	-2.3802	0.0612
27	H	-4.3983	-2.8793	-1.9383
28	C	-4.8557	-0.0565	0.6871
29	H	-4.0581	1.2647	-0.8211
30	C	-5.2302	-1.3695	1.0180
31	H	-5.3350	-3.4011	0.3011
32	H	-4.9838	0.7366	1.4187
33	C	-5.8256	-1.6769	2.3691
34	H	-5.3182	-1.1213	3.1645
35	H	-6.8837	-1.3884	2.3997
36	H	-5.7685	-2.7445	2.6002
37	Br	-1.8857	-1.9565	1.7915
38	Br	-1.9520	3.2203	-1.9654
39	Br	0.3136	-3.0000	-4.3982
40	Br	4.0260	-3.2215	-4.6485
41	Br	5.2464	-0.8397	-2.0887

42	Br	3.7532	-0.4802	2.2987
43	Br	0.9645	-2.0176	4.1714
44	Br	0.9319	5.5462	-1.5848
45	Br	3.7719	3.3346	-0.7420
46	H	-1.3927	-0.2786	-4.0198
47	Si	-0.6591	0.5929	-5.7117
48	C	-1.5703	2.2415	-5.6701
49	C	-1.1462	-0.4951	-7.1700
50	C	1.1931	0.7958	-5.5210
51	H	-2.6277	2.0949	-5.4317
52	H	-1.1473	2.9118	-4.9167
53	H	-1.4922	2.7368	-6.6470
54	H	-0.6343	-1.4617	-7.1404
55	H	-2.2250	-0.6847	-7.1562
56	H	-0.9006	-0.0062	-8.1218
57	C	1.7216	1.7289	-4.6033
58	C	2.1047	0.0210	-6.2676
59	C	3.0975	1.8885	-4.4465
60	H	1.0531	2.3398	-4.0039
61	C	3.4830	0.1847	-6.1173
62	H	1.7384	-0.7118	-6.9815
63	C	3.9832	1.1173	-5.2055
64	H	3.4809	2.6144	-3.7359
65	H	4.1663	-0.4177	-6.7084
66	H	5.0553	1.2428	-5.0856

step_016_t
Energy (POTENTIAL) = -2487.67742084 Eh

	Atom	X	Y	Z
1	Cu	-0.7103	-0.3708	-1.5307
2	N	1.2473	-1.1661	-2.4608
3	N	2.3761	-0.6729	-1.8773
4	N	0.1589	1.5154	-1.3391
5	N	1.4834	1.5509	-1.0134
6	N	0.1922	-0.9014	0.3003
7	N	1.4861	-0.4930	0.4748
8	B	2.2588	0.2550	-0.6490
9	C	1.6410	-2.0198	-3.3978
10	C	3.0478	-2.1032	-3.4497
11	C	3.4714	-1.2228	-2.4587
12	C	-0.2265	2.7710	-1.5732
13	C	0.8554	3.6563	-1.4134
14	C	1.9202	2.8319	-1.0533
15	C	-0.1579	-1.5687	1.4016
16	C	0.9068	-1.6080	2.3199
17	C	1.9305	-0.9112	1.6834
18	H	3.3354	0.5447	-0.2595
19	N	-1.9317	-0.5956	-2.9447
20	S	-3.5836	-0.4569	-3.1040
21	O	-4.0209	-1.5513	-3.9934
22	O	-3.9635	0.9214	-3.4725
23	C	-4.1762	-0.7846	-1.4500
24	C	-4.6231	-2.0651	-1.1273
25	C	-4.2254	0.2578	-0.5183
26	C	-5.1279	-2.3008	0.1526
27	H	-4.5879	-2.8580	-1.8666
28	C	-4.7264	0.0022	0.7538
29	H	-3.8898	1.2517	-0.7908
30	C	-5.1904	-1.2764	1.1080
31	H	-5.4849	-3.2946	0.4086
32	H	-4.7660	0.8066	1.4835
33	C	-5.7425	-1.5280	2.4881
34	H	-5.0527	-1.1697	3.2602
35	H	-6.6887	-0.9927	2.6320
36	H	-5.9289	-2.5917	2.6601
37	Br	-1.8766	-2.3077	1.6106
38	Br	-1.9923	3.1778	-2.0650
39	Br	0.3921	-2.9536	-4.4582
40	Br	4.1099	-3.1562	-4.5911
41	Br	5.2370	-0.8063	-1.9603

42	Br	3.6628	-0.5754	2.3373
43	Br	0.9401	-2.4144	4.0202
44	Br	0.8645	5.5231	-1.6476
45	Br	3.6901	3.3464	-0.6790
46	H	-1.4990	-0.4294	-3.9645
47	Si	-0.6309	0.5795	-5.8380
48	C	-1.5942	2.2018	-5.7740
49	C	-1.0055	-0.4383	-7.3825
50	C	1.2065	0.8270	-5.5654
51	H	-2.6491	2.0221	-5.5460
52	H	-1.1973	2.8707	-5.0056
53	H	-1.5267	2.7176	-6.7412
54	H	-0.5157	-1.4165	-7.3508
55	H	-2.0842	-0.6067	-7.4735
56	H	-0.6679	0.0824	-8.2883
57	C	1.6805	1.7538	-4.6118
58	C	2.1636	0.0726	-6.2760
59	C	3.0456	1.9280	-4.3888
60	H	0.9779	2.3487	-4.0356
61	C	3.5310	0.2494	-6.0582
62	H	1.8404	-0.6557	-7.0150
63	C	3.9766	1.1769	-5.1132
64	H	3.3859	2.6509	-3.6538
65	H	4.2484	-0.3384	-6.6231
66	H	5.0402	1.3136	-4.9409

step_017_t
Energy (POTENTIAL) = -2487.68084537 Eh

	Atom	X	Y	Z
1	Cu	-0.6312	-0.4042	-1.8023
2	N	1.4338	-1.1039	-2.5449
3	N	2.4784	-0.6356	-1.8028
4	N	0.1819	1.4585	-1.3799
5	N	1.4408	1.4865	-0.8550
6	N	0.1136	-1.1811	-0.0064
7	N	1.3171	-0.6983	0.4250
8	B	2.1977	0.1767	-0.5152
9	C	1.9406	-1.9456	-3.4354
10	C	3.3419	-2.0409	-3.3042
11	C	3.6366	-1.1875	-2.2462
12	C	-0.1934	2.7249	-1.5747
13	C	0.8249	3.6083	-1.1748
14	C	1.8459	2.7716	-0.7269
15	C	-0.2996	-2.0739	0.8921
16	C	0.6253	-2.1776	1.9483
17	C	1.6384	-1.2869	1.6019
18	H	3.2141	0.4403	0.0258
19	N	-1.9469	-0.6737	-3.1126
20	S	-3.6089	-0.4969	-3.1074
21	O	-4.1854	-1.6264	-3.8607
22	O	-3.9550	0.8700	-3.5442
23	C	-4.0300	-0.6815	-1.3800
24	C	-4.9135	-1.6944	-1.0075
25	C	-3.5296	0.2215	-0.4377
26	C	-5.2962	-1.7983	0.3304
27	H	-5.2966	-2.3832	-1.7522
28	C	-3.9118	0.0941	0.8944
29	H	-2.8550	1.0150	-0.7358
30	C	-4.8028	-0.9124	1.2994
31	H	-5.9910	-2.5802	0.6244
32	H	-3.5199	0.7919	1.6292
33	C	-5.1880	-1.0605	2.7487
34	H	-4.5100	-1.7594	3.2553
35	H	-5.1279	-0.1053	3.2786
36	H	-6.2027	-1.4554	2.8558
37	Br	-1.8981	-3.0378	0.6622
38	Br	-1.8609	3.1531	-2.3276
39	Br	0.8287	-2.8677	-4.6467
40	Br	4.5414	-3.0463	-4.3496
41	Br	5.3265	-0.7861	-1.5210
42	Br	3.2126	-0.9107	2.5610
43	Br	0.5182	-3.2696	3.4777
44	Br	0.8185	5.4883	-1.2459
45	Br	3.5353	3.2786	-0.0759
46	H	-1.6399	-0.6754	-4.1483
47	Si	-0.7629	0.3567	-6.2362

48	C	-1.9697	1.8091	-6.2830
49	C	-0.6950	-0.6037	-7.8611
50	C	0.9401	0.8701	-5.6453
51	H	-2.9557	1.4766	-6.6235
52	H	-2.0987	2.2564	-5.2937
53	H	-1.6132	2.5867	-6.9717
54	H	-0.0934	-1.5140	-7.7728
55	H	-1.7023	-0.8984	-8.1747
56	H	-0.2589	0.0117	-8.6592
57	C	1.1002	1.9217	-4.7184
58	C	2.1013	0.1829	-6.0588
59	C	2.3581	2.2676	-4.2243
60	H	0.2323	2.4827	-4.3851
61	C	3.3604	0.5262	-5.5656
62	H	2.0235	-0.6338	-6.7712
63	C	3.4931	1.5664	-4.6411
64	H	2.4534	3.0864	-3.5179
65	H	4.2379	-0.0205	-5.8987
66	H	4.4726	1.8323	-4.2539

8. References for computational data

(1) Frisch, M. J.; Trucks, G. W; Schlegel, H. B.; Scuseria, G. E; Robb, M. A.; Cheeseman, J. R.; Scalmani, G.; Barone, V.; Mennucci, B; Petersson, G. A.; Nakatsuji, H.; Caricato, M.; Li, X.; Hratchian, H. P.; Izmaylov, A. F.; Bloino, J.; Zheng, G.; Sonnenberg, J. L.; Hada, M.; Ehara, M.; Toyota, K.; Fukuda, R.; Hasegawa, J.; Ishida, M.; Nakajima, T.; Honda, Y.; Kitao, O.; Nakai, H.; Vreven, T.; Montgomery, J. A. Jr.; Peralta, J. E.; Ogliaro, F.; Bearpark, M.; Heyd, J. J.; Brothers, E.; Kudin, K. N.; Staroverov, V. N.; Keith, T.; Kobayashi, R.; Normand, J.; Raghavachari, K.; Rendell, A.; Burant, J. C.; Iyengar, S. S.; Tomasi, J.; Cossi, M.; Rega, N.; Millam, J. M.; Klene, M.; Knox, J. E.; Cross, J. B.; Bakken, V.; Adamo, C.; Jaramillo, J.; Gomperts, R.; Stratmann, R. E.; Yazyev, O.; Austin, A. J.; Cammi, R.; Pomelli, C.; Ochterski, J. W.; Martin, R. L.; Morokuma, K.; Zakrzewski, V. G.; Voth, G. A.; Salvador, P.; Dannenberg, J. J.; Dapprich, S.; Daniels, A. D.; Farkas, O.; Foresman, J. B.; Ortiz, J. V.; Cioslowski, J.; Fox, D. J. Gaussian 09, Revision D.01, Gaussian, Inc., Wallingford CT, **2013**.

(2) (a) Stephens, P. J.; Devlin, F. J.; Chabalowski, C. F.; Frisch, M. J. Ab Initio Calculation of Vibrational Absorption and Circular Dichroism Spectra Using Density Functional Force Fields *J. Phys. Chem.* **1994**, *98*, 11623-11627. (b) Lee, C.; Yang, W.; Parr, R. G. Development of the Colle-Salvetti correlation-energy formula into a functional of the electron density. *Phys. Rev. B* **1988**, *37*, 785-789. (c) Becke, A. D. Density-functional thermochemistry. III. The role of exact exchange. *J. Chem. Phys.* **1993**, *98*, 5648-5652.

(3) Grimme, S. Semiempirical GGA-type density functional constructed with a long-range dispersion correction. *J. Comput. Chem.* **2006**, *27*, 1787-1799.

(4) Marenich, A. V.; Cramer, C. J.; Truhlar, D.G. Universal Solvation Model Based on Solute Electron Density and on a Continuum Model of the Solvent Defined by the Bulk Dielectric Constant and Atomic Surface Tensions. *J. Phys. Chem. B* **2009**, *113*, 6378-6396.

(5) Hay, P. J.; Wadt, W. R. Ab initio effective core potentials for molecular calculations. Potentials for the transition metal atoms Sc to Hg. *J. Phys. Chem.* **1985**, *82*, 270-283.

(6) Rodríguez, M. R.; Beltrán, Á.; Mudarra, Á. L.; Álvarez, E.; Maseras, F.; Díaz-Requejo, M. M.; Pérez, P. J. Catalytic Nitrene Transfer To Alkynes: A Novel and Versatile Route for the Synthesis of Sulfinamides and Isothiazoles. *Angew. Chem. Int. Ed.* **2017**, *56*, 12842-12847.

(7) Francl, M. M.; Pietro, W. J.; Hehre, W. J.; Binkley, J. S.; Gordon, M. S.; DeFrees, D. J. Self-consistent molecular orbital methods. XXIII. A polarization-type basis set for second-row elements. *J. Phys. Chem. Chem. Phys.* **1982**, **77**, 3654-3665.
(8) (a) Weigend, F. Accurate Coulomb-fitting basis sets for H to Rn. *Phys. Chem.* **2006**, *8*, 1057-1065 10.1039/B515623H. (b) Weigend, F.; Ahlrichs, R. Balanced basis sets of split valence, triple zeta valence and quadruple zeta valence quality for H to Rn: Design and assessment of accuracy. *Phys. Chem. Chem. Phys.* **2005**, *7*, 3297-3305.
(9) (a) Luchini, G.; Alegre-Requena, J.; Funes-Ardoiz, I.; Rodríguez-Guerra, J.; Chen, J. R. **2019**. GoodVibes v3.0.0. Zenodo. 10.5281/zenodo.3346166. (b) Luchini, G.; Alegre-Requena, J. V.; Funes-Ardoiz, I.; Paton R. S. GoodVibes: automated thermochemistry for heterogeneous computational chemistry data [version 1 Paton, peer review: 2 approved with reservations]. F1000Research **2020**, 9 Chem. Inf. Sci., 291.
(10) Grimme, S. Supramolecular Binding Thermodynamics by Dispersion-Corrected Density Functional Theory. *Chem. Eur. J.* **2012**, *18*, 9955-9964.
(11) Ribeiro; R. F.; Marenich, A. V.; Cramer, C. J.; Truhlar, D. G. J. Use of Solution-Phase Vibrational Frequencies in Continuum Models for the Free Energy of Solvation. *J. Phys. Chem. B* **2011**, *115*, 14556-14562.
(12) Pérez-Soto, R.; Besora, M.; Maseras, F. pyssian v1.0.2, **2021**. maserasgroup-repo/pyssian: 1.0.2 (v1.0.2). Zenodo.
(13) Rodríguez-Guerra., J. easymecp v0.3.2, **2020**. jaimergp/easymecp: v0.3.2 (v0.3.2). Zenodo.
(14) Harvey, J. N.; Aschi, M.; Schwarz, H.; Koch, W. The Singlet and Triplet States of Phenyl Cation. A Hybrid Approach for Locationg Minimum Energy Crossing Points Between Non-Interacting Potential Energy Surfaces. *Theor. Chem. Acc.* **1998**, *99*, 95-99.
